# A catalytically polymerized solid electrolyte enables 450 Wh kg^−1^ lithium–metal batteries with thermal–mechanical abuse tolerance

**DOI:** 10.1038/s41467-026-76381-y

**Published:** 2026-08-08

**Authors:** Jiawen Tang, Junyu Zhang, Jiacheng Liu, Yunsong Li, Ahu Shao, Zhiqiao Wang, Xin Wang, Qiurong Jia, Ting Liu, Zhe Liu, Jian-Gan Wang, Zhaohui Wang, Fei Xu, Yue Ma

**Affiliations:** 1 State Key Laboratory of Solidification Processing, Center for Nano Energy Materials, School of Materials Science and Engineering, Northwestern Polytechnical University, Xi’an, PR China; 2 Shaanxi Raisight Energy Tech Co. Ltd, Xi’an, China; 3 Zhengzhou BAK Battery Co. Ltd, Zhengzhou, PR China; 4 College of Materials Science and Engineering, Hunan University, Changsha, China

**Keywords:** Batteries, Batteries, Energy, Batteries

## Abstract

Practical implementation of solid polymer electrolytes is constrained by interfacial instability and manufacturing scalability. Here, we report a roll-to-roll compatible, 9.6-μm-thick solid polymer electrolyte membrane synthesized via in situ 1,3-dioxolane polymerization catalyzed by Lewis-acidic Li_1.3_Al_0.3_Ti_1.7_(PO_4_)_3_ on a polyethylene matrix, achieving a 99.1% conversion rate. The resulting membrane demonstrates 191.7 MPa mechanical strength and 418.7 mS ionic conductance at 25 °C. To resolve multiscale interfacial incompatibilities, a dual-additive strategy is employed: tris(4-fluorophenyl) phosphine constructs a fluorine-rich interphase extending positive electrode tolerance to 4.8 V, while Mg(TFSI)_2_ forms a Li–Mg alloy lowering the negative electrode Li⁺ diffusion barrier to 0.127 eV. Validated in 1.2 Ah pouch cells, this system attains specific energy and energy density of 456.7 Wh kg⁻^1^ and 911.1 Wh L⁻^1^ (based on the total mass and volume of the pouch cell, respectively), stable wide-temperature cycling (−20 to 55 °C), and prevents thermal propagation under abuse conditions.

## Abstract (web summary)

Practical solid-state batteries are limited by scalable manufacturing and thermal safety. Here, authors develop a roll-to-roll compatible, in-situ polymerized solid electrolyte that stabilizes electrode interfaces, delivering robust 1.2 Ah pouch cells that pass stringent abuse safety tests.

## Introduction

The electrification wave has driven the soaring growth in electric vehicles, unmanned aerial systems, and high-end consumer electronics, intensifying the demand for electrochemical cells that surpass the energy-density ceiling of conventional lithium-ion batteries^1,2^. Solid-state lithium metal batteries (SLMBs) integrate the non-flammable, solid-state ionic conductor with high-capacity Li-metal negative electrodes and nickel-rich oxide positive electrodes^3^ (LiNi_x_Co_y_Mn_1₋x₋y_O_2_, NCM, *x* ≥ 0.8), representing a battery paradigm that delivers high energy density while mitigating the safety risks posed by liquid electrolyte leakage and flammability^4^. Among various candidates, solid polymer electrolytes (SPEs) are particularly attractive due to the compelling lightweight nature, molecular tailoring ability and straightforward processability^5^. However, practical deployment of such systems faces two formidable obstacles. First, cell prototyping that simultaneously achieves high specific energy, specific power and durable cycling remains unresolved. More critically, systematic safety validations, particularly protocols to suppress thermal runaway under realistic conditions such as low N/P ratios, high-capacity formats, and wide operating temperatures, have yet to be thoroughly established. These gaps leave the reliability of SLMBs under practical constraints unverified.

**Fig. 1:**
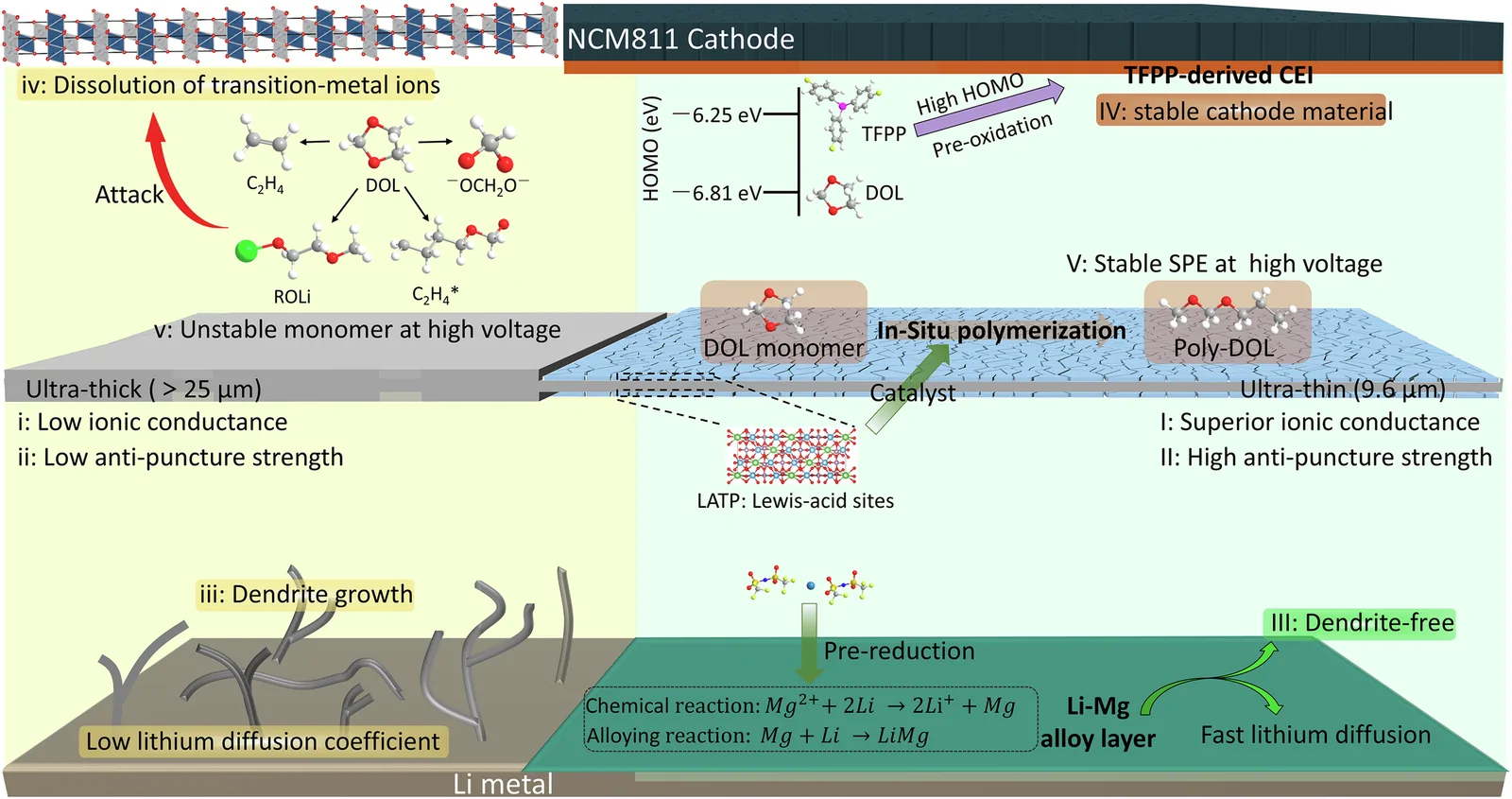
Schematic illustration of Li||NCM811 cell model with the catalytic in-situ polymerization and dual-additive strategies. Left side: the performance fading origins of Li|PDOL-PE|NCM811 prototype, which include: (i) Low ionic conductance; (ii) Low anti-puncture mechanical strength; (iii) Inhomogeneous Li^+^ influx and dendrite growth; (iv) Transition metal (TM) dissolution from the NCM811 positive electrode; (v) DOL monomer oxidization at high voltage. Right side: The features of the as-proposed Li|PDOL-PLMT (PDOL-PE/LATP-Mg(TFSI)_2_-TFPP)|NCM811 system, which include: (I) Superior ionic conductance; (II) High anti-puncture strength; (III) Accelerated lithium diffusion of intermediate Li-Mg alloy that suppresses the dendrite growth; (IV) The suppressed TM cross-talk effect; (V) High monomer conversion efficiency that tolerates the high voltage charging.

Developing Ah-scale pouch-format cells with polymer electrolytes requires navigating an intrinsic trade-off among room-temperature ionic conductivity, dendrite**-**penetration resistance, and tensile strength for lamination and layer-stacking^6^. In such a polymeric matrix, Li⁺ conduction occurs primarily through segmental motion within amorphous regions, while the mechanical strength of the polymer arises from long-chain entanglement and cross-linking density^7,8^. Consequently, the chain stacking and ordering adversely suppressed segmental motion and severely restricted ion transport^9^ (Fig. 1i). To suppress polymeric crystalline domains, various types of ionic liquids^10^, succinonitrile plasticizer^11^ or inorganic nanofillers (e.g., TiO_2_^12^, Al_2_O_3_^13^, and CeO_2_^14^) have been incorporated into polyether matrices. Alternatively, the integration of continuous ceramic conductors (e.g., LAGP^15^, LLZO^16^, LGPS^17^) into thick-layer composite membranes (>30 μm) affords supplementary Li⁺ pathways, but their intrinsic brittleness undermines mechanical robustness at the penalty. While strategies like cross-linking or copolymerization with rigid monomers (e.g., vinylene carbonate^18^, vinyl ethylene carbonate^19^) enhance the tensile strength, the composite polymers were still not robust enough for layer-stacking cell assembly. Furthermore, energy-dense cell designs require re-optimized positive electrode slurries with Li⁺-conductive additives (e.g., sulfides^20^, polymeric salts^21^, halides^22^, or mixed ionic–electronic oxides^23^) to ensure efficient ionic percolation across the high-areal-capacity loadings (>3 mAh cm^−2^). Accordingly, in situ polymerization has been proposed as a promising approach to guarantee the continuous ion-conducting networks, while streamlining roll-to-roll manufacturing. Nevertheless, reducing the thickness of SPE membranes to enhance ionic conductance inevitably compromises their safety characteristics (Fig. 1ii), as thinner membranes are more vulnerable to short circuits caused by dendrite penetration or external puncture^24^ (Fig. 1iii). Notably, SPE thickness dictates cell-level Li^+^ diffusion kinetics and internal direct current resistance (DCR), yet these critical parameters are frequently overlooked. Conventional SPE formation often sacrifices cell-level energy and power density due to its excessive thickness and mass compared to commercial separator/liquid electrolyte systems. To this end, advanced SPE design must synergize high ionic conductivity, cation transference and robust tensile strength compatible with roll-to-roll manufacturing, while attaining the thinness and low areal density comparable to polyolefin separators (9–25 μm, 0.6–1.2 mg cm^−2^) and liquid electrolytes (1.3–1.5 g Ah^−1^). The core challenge lies in the synergistic molecular design of both the scaffold and monomer components, which must reconcile mechanical integrity with optimized polymerization kinetics, cross-linking density, and scaffold conformation.

The cycling endurance and safety metrics of the SLMB also hinge on the multiscale interfacial stability, where the SPE may undergo parasitic oxidation in contact with high-voltage NCM positive electrodes, leading to thick positive electrode electrolyte interphase (CEI) formation and rapid capacity fading^25,26^ (Fig. 1iv). Our previous mechanistic study attributed this interfacial decomposition partially to the catalytic effect of transition metal ions^27^ (e.g., Ni^3^⁺, Co^2^⁺, Mn^3^⁺), which promote the deprotonation of residual 1,3-dioxolane (DOL) oligomeric fragments and unreacted monomers (Fig. 1v). Strategies involving salt-derived binding sites or Lewis acid fillers (e.g., tris(pentafluorophenyl)borane^28^, Sn(OTf)_2_^29^, and yttria-stabilized zirconia (YSZ)^30^) have demonstrated improved polymerization conversion and enhanced Li⁺ transference numbers via anion adsorption. On the negative electrode side, in situ-formed SPEs tend to develop ionically resistive interphases. These interphases comprise species with low Li⁺ diffusivity, such as Li_2_O (~10^−9^ S cm^−1^), lithium alkyl carbonates (ROCO_2_Li, ~10^−10^ S cm^−1^), and Li_2_CO_3_ (ranging from 10^−11^ to 10^−10^ S cm^−1^)^31,32^. During long-term operation, this elevated interfacial resistance promotes irregular lithium deposition and dendrite growth under high current densities (Fig. 1iii). Accordingly, inorganic salts including MoO_3_^33^, SnCl_4_^34^, InCl_3_^35^, and ZnF_2_^36^ have been employed to induce lithiophilic interphases (e.g., Li–Mo, Li–Sn, Li-In/LiCl, Li–Zn/LiF), aiming to mitigate the Li^+^ diffusion barrier^37^. However, a predictive understanding of how the improved multiscale interfacial properties dictate cell-level safety features remains elusive. This is partly because prevailing validation methods are typically limited to component-level tests, such as SPE flammability or mechanical abuse of single-layer pouch cells^38^. More crucially, operation reliability for Ah-scale, or even larger-format (>5 Ah) SLMB models, particularly regarding the thermal runaway suppression under thermal abuse or mechanical damage, has rarely been reported. Therefore, the advancing SLMB prototyping requires a dual focus: developing a scalable, holistic interfacial strategy that ensures multiscale stability under practical cell operation and extreme abuse scenarios, and fundamentally elucidating how these stabilized interfaces inhibit thermal runaway propagation.

Our work proposes a Lewis acid-catalyzed polymerization strategy for constructing Ah-level SLMBs, achieving specific energy, specific power, cycling endurance, and operational safety. Specifically, a mechanically reinforced LATP-functionalized polyethylene substrate (155.9 MPa tensile strength, <5° contact angle) catalyzed 99.1% PDOL polymerization. The resulting SPE membrane thus breaks the performance trade-off, delivers a high ionic conductance of 418.7 mS and a tensile strength of 191.7 MPa at a thickness of 9.6 μm. The dual-additive approach stabilized both electrode interfaces during the formation cycle. The tris(4-fluorophenyl) phosphine (TFPP) experienced sacrificial oxidation to form a robust, electron-blocking CEI, which inhibited transition metal cross-over and extended the SPE’s electrochemical window to 4.8 V. Meanwhile, the Mg(TFSI)_2_ additive was reduced to generate a lithiophilic Li–Mg alloy interphase on the negative electrode, mitigating the lithium diffusion barrier to 0.127 eV. When integrated into a 1.2 Ah Li||NCM811 pouch cell configuration, the prototype delivers a specific energy of 456.7 Wh kg^−1^, an energy density of 911.1 Wh L^−1^ (based on the total mass and volume of the pouch cell, respectively), and a high specific power of 1976.0 W kg^−1^, alongside a capacity retention of 85.5% after 600 cycles. Additionally, the system exhibits operational resilience across a wide temperature range from −20 to 55 °C. More importantly, the composite membrane acts as a thermally stable barrier that effectively mitigates exothermic reactions between the electrodes. This safety profile was validated through industry-standard tests, including nail penetration, thermal chamber exposure, and accelerating rate calorimetry (ARC). These tests confirmed the absence of thermal runaway and an elevated onset temperature for self-heating propagation. By integrating the catalytic in-situ polymerization with dual interfacial stabilization during the formation cycle, this work establishes a methodology for practical SLMBs that balances high specific energy and power, wide-temperature resilience, and operational safety.

## Results

**Fig. 2:**
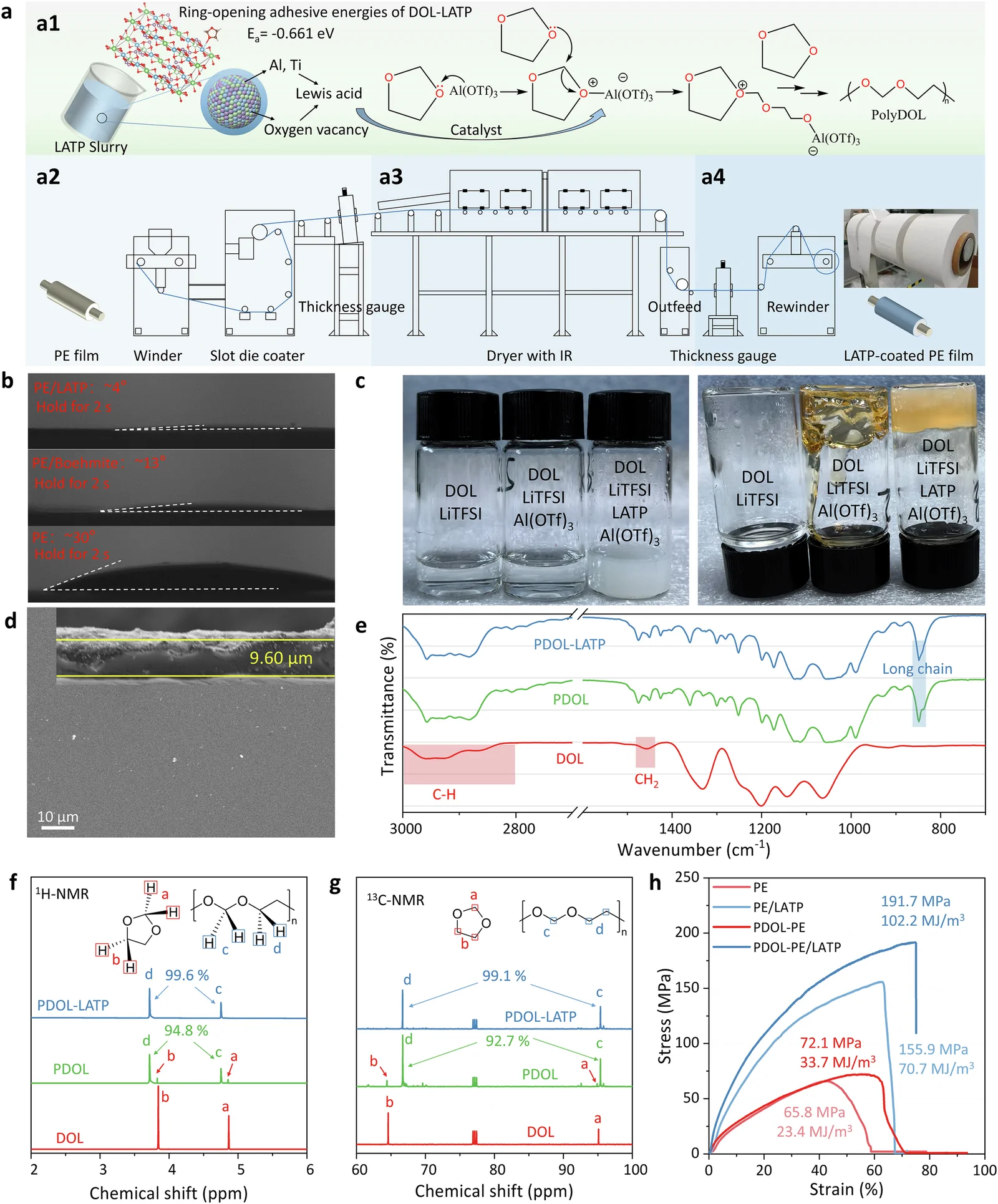
Key characteristics of the PDOL-PE/LATP electrolyte. **a1** Mechanism of the LATP-catalyzed ring-opening polymerization of DOL. **a2-4** Industrial-scale microgroove coating of the LATP particles on the 5 μm porous polyethylene (PE) substrate. **b** Contact angles of DOL monomer droplets on PE/LATP, PE/Boehmite and PE membranes after 2 s contact. **c** Optical images of DOL-LiTFSI, DOL-LiTFSI with Al(OTf)_3_ and DOL-LiTFSI with Al(OTf)_3_-LATP before (left) and after (right) polymerization. **d** Top-view and cross-sectional SEM images of PDOL-PE/LATP SPE. **e** FT-IR spectra of DOL, PDOL and PDOL-LATP. **f** ^1^H and **g** ^13^C NMR spectra of DOL, PDOL and the PDOL-LATP. **h** Strain-stress curves of PE, PE/LATP, PDOL-PE, and PDOL-PE/LATP.

Figure 2a1 illustrates the key procedures of ring-opening polymerization of 1,3-dioxolane (DOL), in which Al(OTf)_3_ initiates the coordination with the oxygen atoms of the cyclic ether structure. The polarization of the C-O bond significantly reduces the five-membered ring’s thermodynamic stability, thus generating an oxonium ion intermediate. The polymerization then proceeds via a cationic chain-growth mechanism, where the activated monomer attacks additional liquid DOL molecules to form solid-state long chains of poly-DOL (PDOL)^39^. Simultaneously, the LATP particle fillers, rich in Lewis acid sites (e.g., Ti^4^⁺, Al^3^⁺) and oxygen vacancies, synergistically promote the polymerization of residual monomers, thereby enhancing the monomer conversion degree and reducing volatile DOL content. Density functional theory (DFT) calculations were employed to evaluate the ring-opening adsorption energies of DOL. The LATP-DOL interaction yielded an adsorption energy of −0.661 eV, which is lower than that of conventional lithium salts (LiPF_6_: −0.537 eV; LiDFOB: −0.547 eV) and is comparable to the strong Lewis acid initiator Al(OTf)_3_ (−0.730 eV) (Supplementary Fig. 1). Such a high binding affinity confirms that LATP serves as an effective catalytic site, actively activating the DOL monomers and promoting the ring-opening polymerization. This enhanced interaction confirms the catalytic role of LATP in facilitating the ring-opening polymerization and improved monomer conversion efficiency^40,41^, which is expected to enhance the cycling stability of SPE when paired with a high-voltage positive electrode. Subsequently, the 1.5-μm-thick coating of LATP nanoparticles (Supplementary Fig. 2) was applied to both sides of a 5-μm-thick polyethylene (PE) substrate via a microgroove coating. Scalable processing involves the stepwise slitting, slot-die coating (Fig. 2a2), roller pressing with thickness control, drying (Fig. 2a3), and rewinding into separator reels (500 m length, inset image in Fig. 2a4). Systematic coating optimization was conducted to meet commercial standards (Supplementary Table 1), yielding a PE/LATP separator with a puncture resistance of 447 gf and a breakdown voltage of 0.8 kV. Furthermore, the separator exhibits thermal stability, maintaining a low shrinkage of 0.5–0.7% after 1 h at 150 °C. Scanning electron microscopy (SEM) confirmed that the PE/LATP separator maintained a uniform and smooth surface, with a total thickness of 8.2 μm (Supplementary Fig. 3). Moreover, contact angle measurements of DOL monomer droplets after 2 s revealed values of 4° on PE/LATP, 13° on PE/Boehmite, and 30° on bare PE separators (Fig. 2b). These results indicate that LATP coating markedly enhanced the affinity and wettability toward the DOL precursor. The DOL-LiTFSI system with Al(OTf)_3_ initiator was transformed into a quasi-solid-state yet with perceptible fluidity. In stark contrast, the LATP-incorporated sample exhibited no fluidity, demonstrating a superior polymerization degree with the aid of the Lewis-acid catalytic effect (Fig. 2c). Therefore, the PE/LATP scaffold intimately integrated within PDOL upon the in-situ polymerization process, generating a smooth PDOL-PE/LATP SPE membrane with the uniform thickness of 9.6 μm (Fig. 2d). By comparison, the use of a 9 μm PE scaffold alone yielded a much thicker PDOL-PE membrane (~15–16 μm, Supplementary Fig. 4).

Fourier transform infrared spectroscopy (FT-IR) was employed to track functional group evolution during the polymerization and assess the influence of LATP addition on PDOL segment structure (Fig. 2e). Compared with the DOL, the PDOL and PDOL-LATP systems exhibited a characteristic long-chain vibration at 848.6 cm⁻^1^. Furthermore, the shift in C–O–C vibration and absence of C–H vibration modes suggest successful ring-opening DOL polymerization into long-chain PDOL after initiator introduction. Notably, LATP incorporation did not alter the position or intensity of PDOL characteristic vibrations, indicating that the PE/LATP scaffold does not disrupt the chain structure of PDOL. Nuclear magnetic resonance (NMR) analysis (Fig. 2f, g) further confirms the complete PDOL conversion. New ^1^H and ^13^C peaks emerged after polymerization, consistent with the PDOL structure, where ^1^H and ^13^C sites (a, b in DOL monomer) shift to c, d upon polymerization. Therefore, the relative content of PDOL to DOL monomer can be quantified by the peak area ratio of c/a or d/b. Calculation from Fig. 2f and g reveals that the PDOL/DOL content ratio reaches 92.7–94.8% in the electrolyte with initiator alone, whereas this value increases to 99.1–99.6% when both initiator and LATP particles are present, demonstrating the prominent catalytic effect of LATP in promoting DOL polymerization. Tensile testing (Fig. 2h) further revealed that the PDOL-PE/LATP achieves an elongation at break of 75% and a tensile strength of 191.7 MPa. This strength is nearly triple that of the pristine PDOL-PE (72.1 MPa, Fig. 2h). Moreover, the fracture toughness derived from the integral area under the stress-strain curve was substantially higher for PDOL-PE/LATP (102.2 MJ m^−3^) compared to PE/LATP (70.7 MJ m^−3^) or PDOL-PE (33.7 MJ m^−3^). Collectively, these results indicate that the PE/LATP scaffold provides crucial mechanical reinforcement, while the PDOL incorporation markedly increases plasticity and ultimate mechanical strength. Electrochemically, the PDOL-PE/LATP electrolyte demonstrated an ionic conductivity of 2.0 × 10^−4^ S cm^−1^ at 25 °C, markedly higher than PDOL-PE (8.2 × 10^−5^ S cm^−1^, Supplementary Fig. 5). Benefiting from the reduced thickness and LATP reinforcement, the 9.6-μm-thick PDOL-PE/LATP membrane achieves an ionic conductance of 418.7 mS at 25 °C, which is higher than that of the thicker PDOL-PE electrolyte (16.7 μm, 98.7 mS), directly correlating with a minimized direct-current internal resistance (DCIR). Arrhenius fitting further revealed a lower activation energy for Li⁺ migration in PDOL-PE/LATP (0.137 eV) compared to PDOL-PE (0.261 eV, Supplementary Figs. 6 and 7). In addition, the Li⁺ transference number (t_Li⁺_) of PDOL-PE/LATP reached 0.62 (measured by the Bruce–Vincent–Evans method), which is higher than the value of 0.28 for PDOL-PE (Supplementary Fig. 8). Therefore, the PDOL-PE/LATP electrolyte achieves a favorable balance between ionic transport and mechanical properties at a thickness of 9.6 μm.

**Fig. 3:**
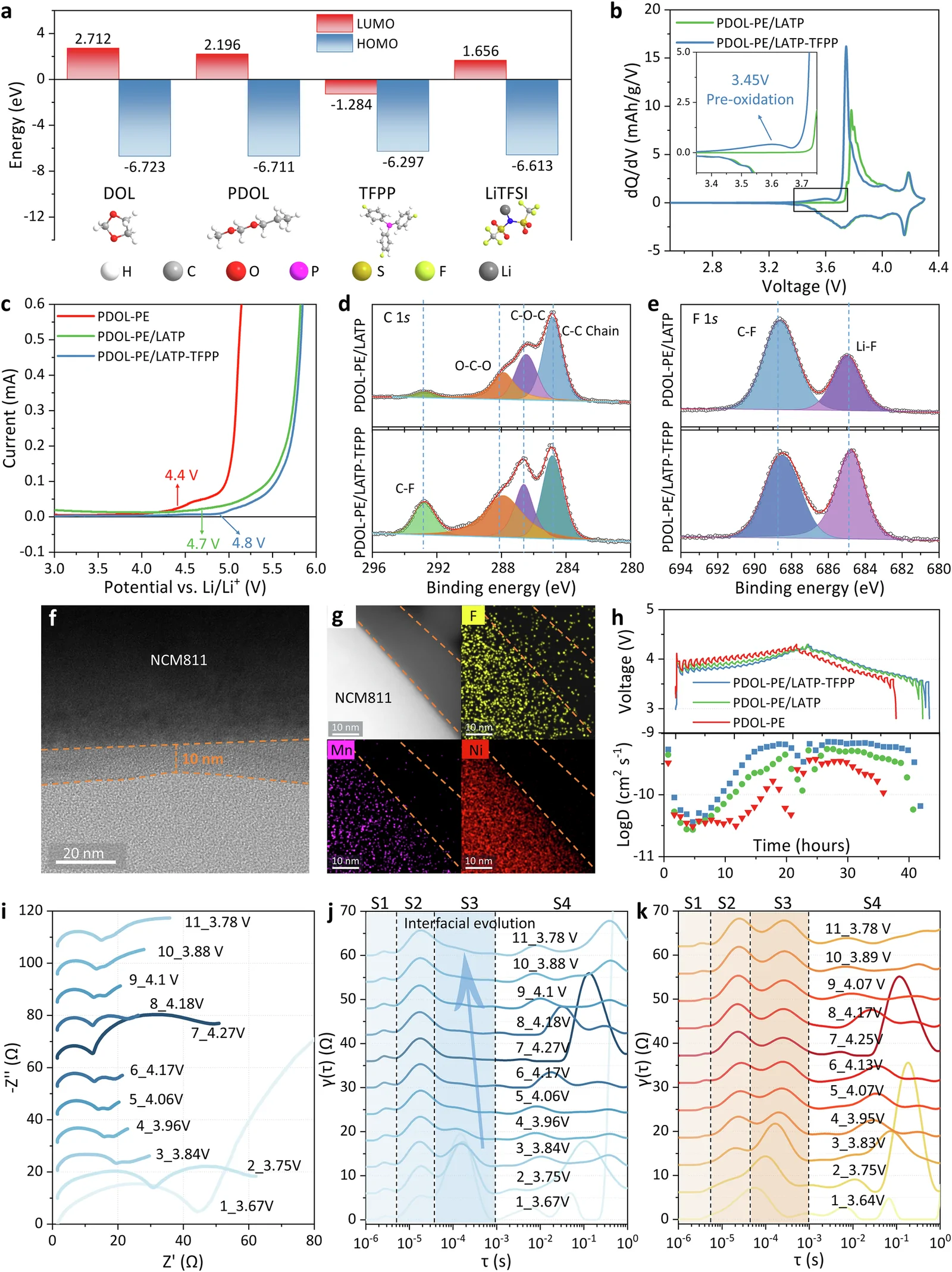
Characterization of the TFPP-derived CEI and its effect on NCM811 interfacial stability. **a** DFT-calculated HOMO/LUMO energies of DOL, PDOL, TFPP and LiTFSI salt. **b** dQ/dV curves of Li|PDOL-PE/LATP|NCM811 and Li|PDOL-PE/LATP-TFPP|NCM811 cells during the initial cycle. **c** LSV curves of PDOL, PDOL-PE/LATP and PDOL-PE/LATP-TFPP SPEs after the formation cycle. **d,** **e** C 1*s* and F 1*s* core-level XPS spectra of NCM811 positive electrodes after cycling with the PDOL-PE/LATP and PDOL-PE/LATP-TFPP SPEs. **f,** **g** TEM image, high-angle annular dark-field image and F, Mn, Ni elemental mappings of a cycled NCM811 positive electrode particle in the Li|PDOL-PE/LATP-TFPP|NCM811 cell. **h** GITT plots and corresponding diffusion coefficients of Li||NCM811 cells as paired with PDOL-PE, PDOL-PE/LATP, and PDOL-PE/LATP-TFPP. **i** Galvanostatic electrochemical impedance spectra (GEIS) collected during initial charge/discharge of the Li|PDOL-PE/LATP-TFPP|NCM811 cell, and **j** corresponding DRT analysis. **k** DRT analysis derived from the initial charge/discharge GEIS data of the Li|PDOL-PE/LATP|NCM811 cell.

Reversible capacity decay stems from transition metal (TM) dissolution from the positive electrode and thick, non-uniform CEI formation during high-voltage cycling. The highest occupied molecular orbital (HOMO) energies and electrostatic potential (ESP) of key species were determined by DFT calculations to screen potential additives (Supplementary Figs. 9, 10 and Supplementary Data 1–5), with the results for the primary components illustrated in Fig. 3a. The results identified TFPP (−6.297 eV) as the most susceptible to oxidation, followed by LiTFSI (−6.613 eV), PDOL (−6.711 eV), and DOL (−6.723 eV). Thermodynamic and electrostatic potential analyses demonstrate that TFPP undergoes targeted, preferential sacrificial oxidation over the polymer matrix while preserving its perfluoroalkyl groups, uniquely enabling the construction of a robust, F-rich protective interphase unlike overly inert or non-fluorinated benchmarks. Accordingly, dQ/dV analysis (Fig. 3b) revealed that the NCM811 positive electrode paired with the PDOL-PE/LATP-TFPP SPE exhibited a sacrificial oxidation peak at 3.45 V, the potential of which is distinctly lower than that observed with TFPP-free PDOL-PE/LATP (3.69 V). Subsequent cycles (Supplementary Fig. 11) showed no emerging redox peaks, indicating complete TFPP oxidation during the initial cycle and the formation of a stable, passivating CEI. Linear sweep voltammetry (LSV) revealed the stability of the PDOL-PE/LATP-TFPP electrolyte reached 4.8 V, notably higher than that of PDOL-PE/LATP (4.7 V) and PDOL-PE (4.4 V) (Fig. 3c). To verify the oxidative stability limit beyond the dynamic LSV scans, potentiostatic floating tests were performed (Supplementary Fig. 12). While the PDOL-PE and PDOL-PE/LATP electrolytes exhibited sharp current surges-indicative of rapid decomposition—at 4.5 and 4.7 V, respectively, the optimized PDOL-PE/LATP-TFPP system maintained a negligible and stable leakage current even at 4.8 V, confirming its superior high-voltage tolerance. Compared to the TFPP-free system, the NCM811 positive electrode cycled with PDOL-PE/LATP-TFPP showed an additional 43 mAh g^−1^ of formation charge capacity (Supplementary Fig. 13), confirming the electrochemical decomposition of TFPP and its contribution to a robust CEI. To further characterize the CEI composition, X-ray photoelectron spectroscopy (XPS) analysis was conducted to examine the CEI formation on NCM811 positive electrodes cycled with different SPEs. After 20 cycles, the C 1*s* spectra (Fig. 3d) revealed four characteristic peaks: those situated at 284.8, 286.6, and 287.9 eV correspond to the long-chain PDOL segments, while the peak at 292.8 eV is attributed to C−F from TFSI^−^^42^. In the F 1*s* spectra (Fig. 3e), signals at 685.0 and 688.6 eV are assignable to LiF and C−F species, respectively^43^. The increased LiF content after cycling further corroborates the robust CEI formation. High-resolution transmission electron microscopy (HRTEM) exhibits a uniform amorphous CEI layer of ~10 nm thickness on positive electrode particles cycled in the PDOL-PE/LATP-TFPP electrolyte (Fig. 3f). Corresponding EDS mapping (Fig. 3g) clearly showed homogeneous distributions of F, Mn, and Ni signals, confirming the incorporation of TFPP-derived species within the CEI. In contrast, an irregular and amorphous CEI layer with non-uniform thickness (0–4.5 nm) was observed on the positive electrode cycled in PDOL-PE/LATP (Supplementary Fig. 14), resulting in accelerated structural degradation of the positive electrode.

Galvanostatic intermittent titration technique (GITT) measurements revealed a Li^+^ diffusion coefficient (D_Li+_) of approximately 10^−9.2^ cm^2^ s^−1^ for the Li | |NCM811 cell utilizing the PDOL-PE/LATP-TFPP electrolyte. This value is 2–3 times higher than that of the cell employing the PDOL-PE/LATP electrolyte (Fig. 3h). Further insight was provided by galvanostatic electrochemical impedance spectroscopy (GEIS, Fig. 3i and Supplementary Fig. 15), which elucidated the role of TFPP in facilitating the charge transfer kinetics. Upon initial charging of the Li|PDOL-PE/LATP-TFPP|NCM811 cell, the high-frequency region (∼1 MHz) of the EIS spectrum corresponds to the bulk electrolyte resistance (*R*_bulk_, see fitting circuit in Supplementary Fig. 16). The intermediate-frequency range (500 kHz–50 Hz) exhibits a semicircle attributable to interfacial resistance (*R*_int_), while the low-frequency tail (50–0.1 Hz) reflects bulk Li⁺ diffusion within the electrode (Warburg behavior). As the state of charge (SOC) increases, the low-frequency Warburg tail gradually diminishes, and a depressed semicircle associated with charge-transfer resistance (*R*_ct_) emerges. Throughout cycling, *R*_bulk_ remains constant, while *R*_int_ and *R*_ct_ decrease in tandem during charging and increase concurrently during discharging. Distribution of relaxation times (DRT) analysis (Fig. 3j) further deconvolutes the impedance into four dominant processes: bulk electrolyte response (S1), interfacial resistance (S2), ion transport through the CEI (S3), and charge transfer (S4)^44^. During the formation cycle, peaks in regions S1 and S2 remain stable in both PDOL-PE/LATP and PDOL-PE/LATP-TFPP, confirming the high voltage stability of these SPEs. The fading of the S3 peak demonstrates CEI evolution (Fig. 3j), as the TFPP-derived CEI develops a higher proportion of ionically conductive inorganic species, substantially reducing internal resistance. In stark contrast, the Li|PDOL-PE/LATP|NCM811 cell shows minimal attenuation in the S3 peak intensity (Fig. 3k and Supplementary Fig. 17), suggesting a less dense and ionically insulating CEI. This comparison confirms that the TFPP-induced CEI effectively enhances ionic conductivity and suppresses parasitic reactions.

**Fig. 4:**
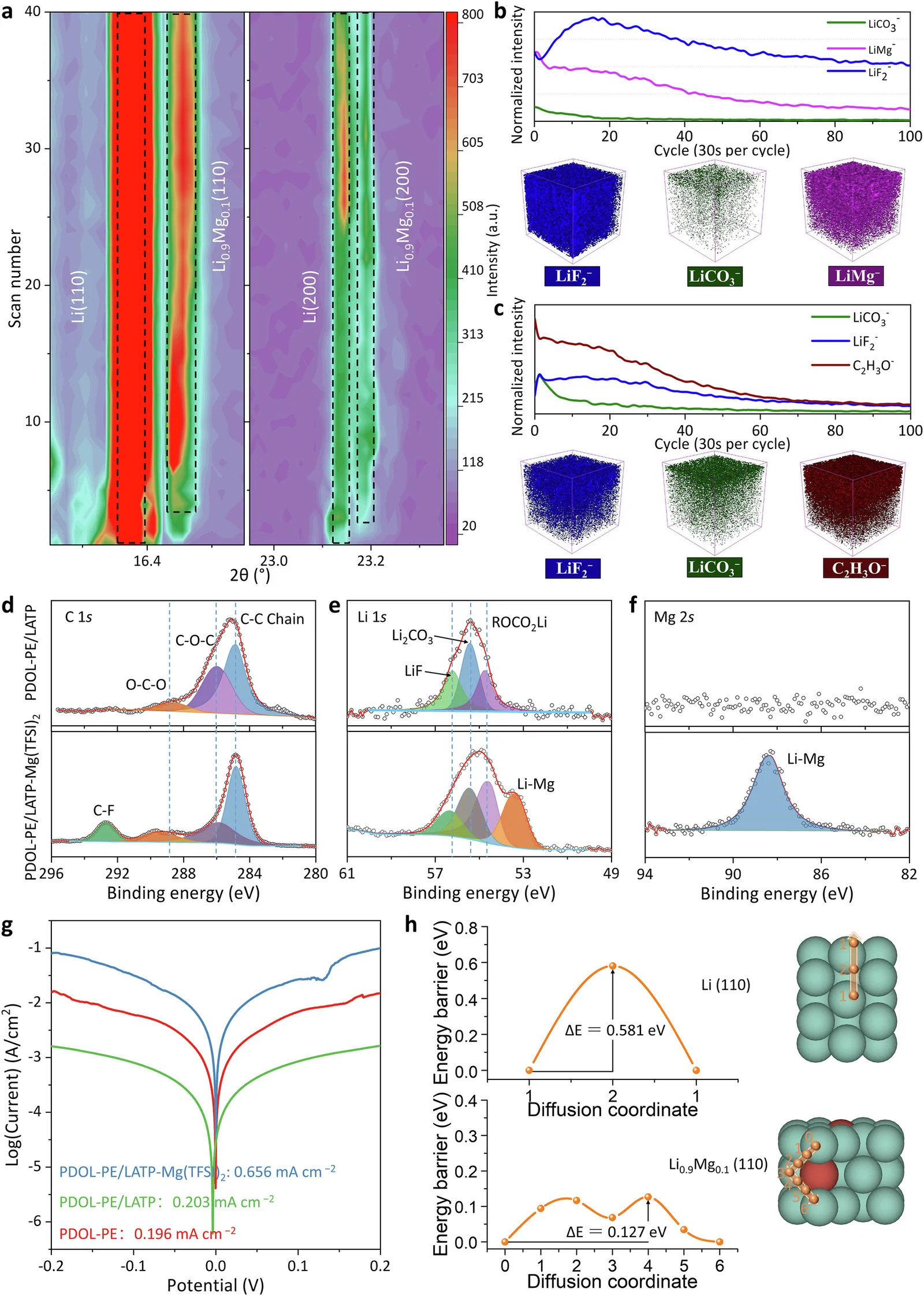
The compositional characterization and spatial arrangement of Mg(TFSI)_2_-dervied SEI. **a** The real-time phase evolution of the Li|PDOL-PE/LATP-Mg(TFSI)_2_|Li cell during the formation process (Mo K-alpha radiation). **b** TOF-SIMS depth profiling and 3D spatial distribution of LiF_2_⁻, LiCO_3_^−^, and LiMg^−^ fragments on the surface of lithium foil cycled for 100 h (0.3 mA cm^−^^1^, 0.3 mAh cm^−^^1^) in the Li|PDOL-PE/LATP-Mg(TFSI)_2_|Li cell. **c** TOF-SIMS depth profiles and 3D spatial distribution of LiF_2_⁻, LiCO_3_^−^, and C_2_H_3_O^−^ fragments on the surface of lithium foil cycled for 100 h (0.3 mA cm^−^^1^, 0.3 mAh cm^−^^1^) in the Li|PDOL-PE/LATP|Li cell. **d**–**f** C 1*s*, Li 1*s*, and Mg 2*s* core-level XPS spectra of Li foil disassembled from the Li|PDOL-PE/LATP-Mg(TFSI)_2_|Li and Li|PDOL-PE/LATP|Li cells after 100 h cycling (0.3 mA cm^−^^1^, 0.3 mAh cm^−^^1^). **g** Tafel plots of the Li symmetric cells utilizing PDOL-PE, PDOL-PE/LATP and PDOL-PE/LATP-Mg(TFSI)_2_. **h** Calculated diffusion pathways and corresponding energy barriers for Li atom diffusion on the surface of Li foil and Li–Mg alloy.

Additionally, the Mg(TFSI)_2_ additive is designed to modulate the SEI composition on the lithium negative electrode via a controlled reduction process. Driven by the higher standard reduction potential of Mg²⁺/Mg (−2.36 V vs. SHE) relative to Li^+^/Li (−3.04 V), Mg^2+^ undergoes a spontaneous displacement reaction with lithium metal during the initial formation cycle: Mg^2+^ + 2Li → 2Li⁺ + Mg^45^. This metallic Mg subsequently alloys with lithium, forming a lithiophilic interphase. To probe the dynamic evolution of Li–Mg alloys, operando transmission X-ray diffraction (XRD) was conducted to scrutinize the formation of Li–Mg alloys as the Mg(TFSI)_2_ additive interacts with the lithium negative electrode. As shown in Fig. 4a, the initial diffraction peak at 16.35° corresponds to metallic lithium in its initial state. Upon the Li symmetric cell cycling, new diffraction peaks that are assignable to Li_0.9_Mg_0.1_ (110) and Li_0.9_Mg_0.1_ (200) intermediate alloy emerged at 16.45° and 23.2°, confirming the in-situ Mg reduction and subsequent intermediate alloy formation at the interface. As illustrated in Fig. 4b, time-of-flight secondary ion mass spectrometry (ToF-SIMS) analysis identified ionic fragments including LiF_2_^−^, LiCO_3_^−^, and LiMg^−^ within the Mg(TFSI)_2_-derived SEI, corresponding to LiF, Li_2_CO_3_, and Li–Mg alloy phases, respectively. Normalized depth profiles revealed a gradient distribution of these species: signals from LiCO_3_^−^ and LiMg^−^ progressively decrease and stabilize with sputtering depth, whereas the LiF_2_⁻ intensity increases and eventually plateaus. In comparison with the PDOL-PE/LATP-derived SEI (Fig. 4c), the Mg(TFSI)_2_-containing SPE yields a weaker LiCO_3_^−^ signal, suggesting suppressed parasitic reactions due to preferential Mg^2^⁺ reduction. Concurrently, the stronger LiF_2_^−^ signal suggested a more inorganic-rich interphase. These findings indicate that the lithiophilic Li–Mg alloy, uniformly distributed in the SEI outer layer, facilitates homogeneous Li⁺ flux and regulates Li deposition. In sharp contrast, the PDOL-PE/LATP SEI exhibits pronounced C_2_H_3_O^−^ and LiCO_3_^−^ signals (Fig. 4c). The accumulation of C_2_H_3_O⁻ species in the outer SEI likely compromises mechanical integrity, while the underlying inorganic layer, rich in Li_2_CO_3_ and LiF, impedes Li⁺ diffusivity, ultimately restricting high-rate cycling performance.

XPS analysis provided further insights into the interfacial composition of the Li negative electrode (Fig. 4d–f). In the C 1*s* spectrum (Fig. 4d), peaks at 284.8, 286.0, and 288.1 eV are assigned to PDOL-derived carbon species, while the signal at 292.7 eV corresponds to C–F bonds from TFSI^−^. Deconvolution of the Li 1*s* spectrum (Fig. 4e) identified components at 56.4 eV (LiF), 55.4 eV (Li_2_CO_3_), and 54.6 eV (Li–O_2_COR). Notably, the spectrum for Mg(TFSI)_2_-containing SPE reveals an additional peak at 53.4 eV, attributed to a Li–Mg alloy, which is corroborated by the Mg 2*s* signal at 88.4 eV (Fig. 4f). To assess Li⁺ transfer kinetics, exchange current density (j_0_) was determined from Tafel plots. The PDOL-PE/LATP-Mg(TFSI)_2_ electrolyte delivers an i₀ of 0.656 mA cm^−2^, over three times higher than PDOL-PE (0.196 mA cm^−2^) and PDOL-PE/LATP (0.203 mA cm^−2^) systems (Fig. 4g), indicating significantly enhanced interfacial charge transfer. Complementing these findings, DFT calculations revealed a substantially lower Li diffusion barrier on the Li_0.9_Mg_0.1_ alloy surface (0.127 eV) than on bare Li metal (0.581 eV, Fig. 4h). This reduced energy barrier promotes rapid and homogeneous interfacial Li⁺ diffusion on the substrate, mitigating localized Li aggregation and favoring uniform deposition.

**Fig. 5:**
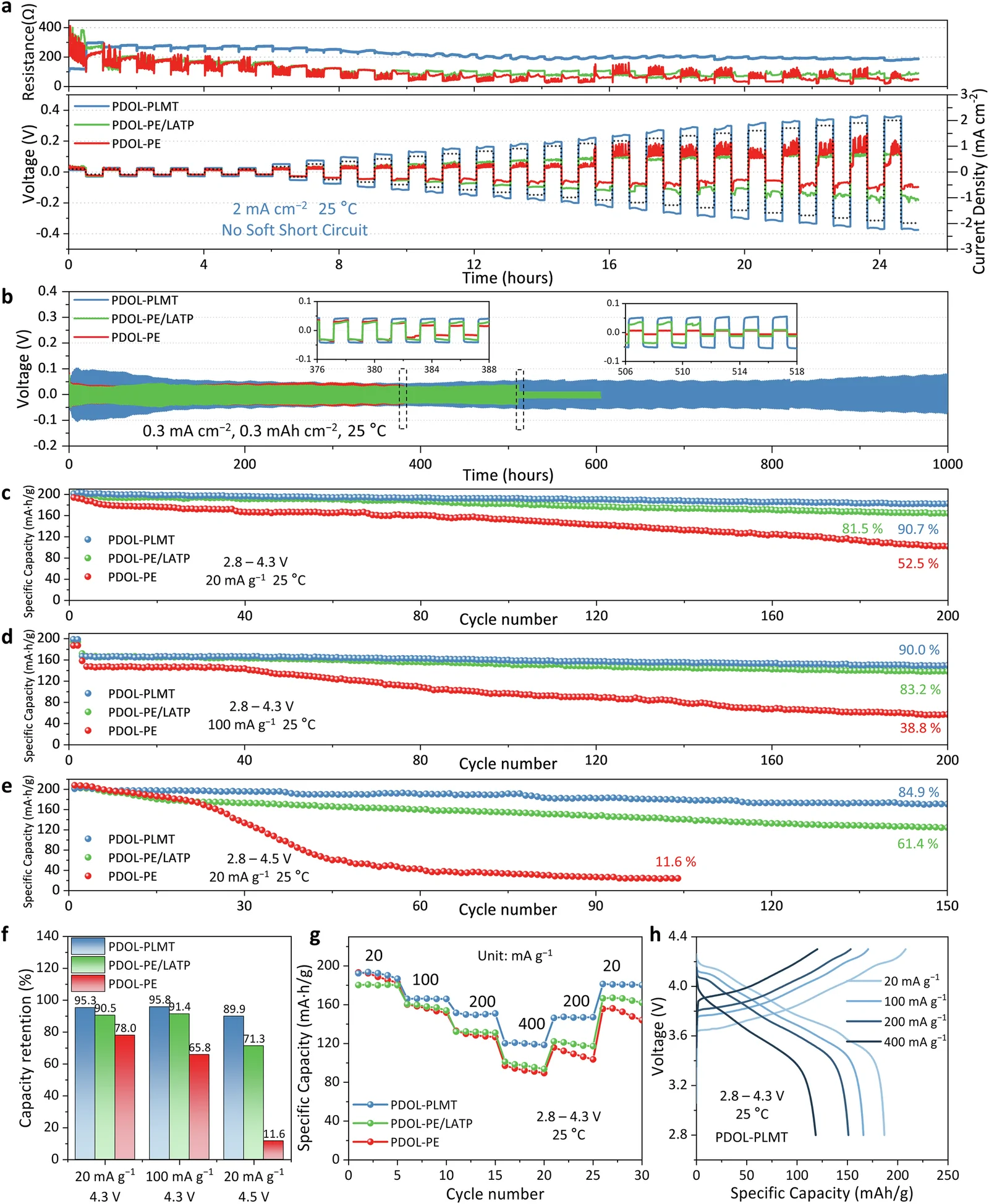
Electrochemical performance of Li symmetric cells and Li||NCM811 (1C = 200 mA g^−1^) coin-cell evaluations using PDOL-PE, PDOL-PE/LATP, and PDOL-PLMT SPEs. **a** Galvanostatic cycling of Li symmetric cells at step-increased current densities. **b** Long-term galvanostatic cycling of Li symmetric cells using PDOL-PE, PDOL-PE/LATP, and PDOL-PLMT SPEs. **c,** **d** Cycling performance of Li||NCM811 cells with PDOL-PE, PDOL-PE/LATP, and PDOL-PLMT SPEs at 20 and 100 mA g^−1^ with the cutoff voltage of 4.3 V. **e** Cycling performance of Li||NCM811 cells with PDOL-PE, PDOL-PE/LATP, and PDOL-PLMT SPEs within the voltage range of 2.8–4.5 V. **f** Discharge capacity retention values after 100 cycles of the full cells under various cutoff voltages and currents. **g** Specific current performance of Li||NCM811 cells with PDOL-PE, PDOL-PE/LATP and PDOL-PLMT SPEs. **h** Charge–discharge curves of the Li||NCM811 with PDOL-PLMT SPE at various currents.

Following the formation cycle, the Li symmetric cell employing the PDOL-PE exhibited an area-specific resistance (ASR) of 96 Ω cm^−2^, whereas the cell with PDOL-PLMT (PDOL-PE/LATP- Mg(TFSI)_2_-TFPP) showed a significantly lower ASR of 67 Ω cm^−2^ (Supplementary Fig. 18), owing to a more ionically conductive SEI. Molecular dynamics simulations demonstrate that the additives provide interfacial stabilization without disrupting bulk Li⁺ transport pathways or inducing Li⁺ trapping (Supplementary Fig. 19; Supplementary Data 6 and 7). Moreover, the cell with PDOL-PLMT achieved a critical current density (CCD) of 2 mA cm^−2^ without soft short-circuiting (Fig. 5a), more than double the 0.8 mA cm^−2^ of the cell with PDOL-PE. As the current density increased, the Li symmetric cell with PDOL-PE exhibited pronounced voltage fluctuations, indicative of interfacial failure from dead Li accumulation and dendritic growth. In contrast, the PDOL-PE/LATP system without a dual additive strategy showed a lower CCD value of 1.2 mA cm^−2^ after experiencing a soft short-circuit. At 25 °C, the Li symmetric cell with PDOL-PLMT rendered robust cycling for over 1000 h with a steady polarization of only 66 mV (0.3 mA cm^−2^, Fig. 5b). Notably, the cell demonstrated the initial polarization increase, which is attributed to the temporary hindrance of Li⁺ transport by Mg(TFSI)_2_ salt at the SPE/Li interface. As cycling continued, polarization voltage decreased and stabilized, consistent with the formation of an interfacial intermediate layer resulting from alloy reactions between Mg(TFSI)_2_ and Li. Crucially, the formation of this lithiophilic Li–Mg interphase lowers the Li^+^ diffusion barrier and enhances the exchange current density (j_0_), fundamentally driving the elevated CCD and fast-kinetic rate performance. By comparison, Li symmetric cells with PDOL-PE/LATP and PDOL-PE experienced short circuits after only 511 h and 382 h, respectively. To rigorously evaluate negative electrode reversibility under deep cycling conditions (0.5 mA cm⁻^2^, 5 mAh cm⁻^2^), Li|Cu cells were assembled. The PDOL-PLMT system demonstrated exceptional stability, achieving a high average coulombic efficiency (ACE) of 99.03%. Notably, the PDOL-PE/LATP-Mg(TFSI)_2_ exhibited nearly identical reversibility to the full system, whereas the bare PDOL-PE control failed rapidly with erratic voltage oscillations and sharp polarization buildup (Supplementary Fig. 20). This comparative performance definitively identifies Mg(TFSI)_2_ as the dominant factor in governing negative electrode stability, confirming that the in situ formed lithiophilic Li-Mg alloy interface is essential for regulating uniform deposition under high areal capacities.

EIS curves of Li||NCM811 cells (Supplementary Fig. 21) revealed that the charge-transfer resistance (*R*_ct_, represented by intermediate-frequency semicircles) was markedly lower in cell systems with PDOL-PE/LATP and PDOL-PLMT than in that with PDOL-PE. Full coin cells assembled with high-mass-loading NCM811 positive electrodes (5 ~ 6 mg cm^−2^) further highlighted the performance merits of PDOL-PLMT SPE. The cell using PDOL-PE exhibited rapid performance degradation, retaining only 52.5% of its initial capacity after 200 cycles at 20 mA g^−1^ (Fig. 5c and Supplementary Fig. 22). In contrast, the PDOL-PE/LATP cell showed significantly enhanced cycling stability, achieving 81.5% capacity retention (164.5 mAh g^−1^). The cell with PDOL-PLMT SPE rendered the best performance, maintaining 90.7% retention after 200 cycles. At 100 mA g^−1^ (Fig. 5d and Supplementary Fig. 23), the Li|PDOL-PLMT|NCM811 cell still retained 90.0% of its initial capacity after 200 cycles, far exceeding the PDOL-PE-based cell (38.8%). Under an elevated cut-off voltage of 4.5 V (Fig. 5e and Supplementary Fig. 24), the PDOL-PLMT-based cell delivered a reversible capacity of 201.3 mAh g^−1^ at 20 mA g^−1^ and retained 84.9% after 150 cycles. By comparison, the cell with PDOL-PE/LATP showed rapid capacity decay, declining from 202.7 to 143.9 mAh g^−1^ within 100 cycles. The PDOL-PE-based cell performed worst, with capacity dropping from 208.1 to 53.2 mAh g^−1^ in only 50 cycles. The distinct disparities in cycling stability, summarized in Fig. 5f, stem from a fundamental improvement in polymerization degree and interfacial stabilization of the SPE. While the baseline PDOL-PE system rapidly degrades due to high-voltage oxidation of residual monomers, LATP-catalyzed polymerization eliminates the vulnerability of unreacted DOL. In addition, the incorporation of TFPP and Mg(TFSI)_2_ additives further protects the NCM811 positive electrode and accelerates Li⁺ kinetics at the negative electrode surface, collectively promoting the cycling performance even under high-voltage and high-rate conditions.

Post-mortem morphological characterization showed that NCM811 particles cycled with PDOL-PE/LATP SPE (2.8–4.5 V) exhibited numerous microcracks due to repeated structural expansion and shrinkage (Supplementary Fig. 25a, b), whereas those NCM particles cycled with PDOL-PLMT remained smooth and intact (Supplementary Fig. 25c, d), indicating effective structural preservation. This enhanced high-voltage stability is ascribed to the protective TFPP-derived CEI layer, which mitigates PDOL oxidation and TM dissolution. Benefiting from its high voltage tolerance and facile interfacial kinetics, the Li|PDOL-PLMT|NCM811 cell demonstrated impressive rate capability (Fig. 5g). It delivered discharge capacities of 186.8, 166.0, 151.0, and 118.5 mAh g^−1^ at 20, 100, 200, and 400 mA g^−1^, respectively, significantly outperforming the PDOL-PE/LATP and PDOL-PE cells (Supplementary Fig. 26). The corresponding charge-discharge voltage profiles of the batteries at different rates, as seen in Fig. 5h, demonstrate low electrochemical polarization in PDOL-PLMT batteries. When the C-rate was restored to 20 mA g^−1^, the PDOL-PLMT-based cell still exhibited the discharge capacity of 181.3 mAh g^−1^, compared to only 155.7 mAh g^−1^ for the PDOL-PE cell. These results confirm that the PDOL-PLMT electrolyte enhances reaction kinetics and cycling stability in high-voltage LMBs by effectively suppressing interfacial side reactions. Further systematic control studies (Supplementary Figs. 27–31) explicitly decouple the dual-additive functions, verifying that Mg(TFSI)_2_ regulates the anodic fast-kinetics while the TFPP-derived CEI is indispensable for long-term, high-voltage positive electrode preservation.

The suppression of TM crossover was systematically evaluated across quantitative, spectroscopic, and spatial dimensions. Quantitatively, ICP analysis reveals a substantial ~66% reduction in TM deposition on the cycled negative electrodes for TFPP-containing systems (Supplementary Fig. 32). This macroscopic mitigation is further corroborated by high-resolution XPS (Supplementary Fig. 33), which demonstrates a step-wise elimination of surface Ni species: from pronounced Ni^3^⁺ and Ni^2^⁺ peaks in the PDOL-PE, to a weak Ni^3^⁺ signal in the PDOL-PE/LATP, to complete absence in the PDOL-PLMT. Spatially, ToF-SIMS depth profiling reveals that while TMs penetrate deep into the bulk of the control negative electrode, driving continuous parasitic reactions, they are strictly confined to the outermost surface and rapidly vanish upon sputtering in the PDOL-PLMT system (Supplementary Fig. 34). Collectively, this multi-scale evidence unambiguously confirms that the robust TFPP-derived CEI shuts down NCM811 dissolution at its source, effectively blocking deep TM diffusion and preserving both positive electrode structural integrity and negative electrode SEI stability.

**Fig. 6:**
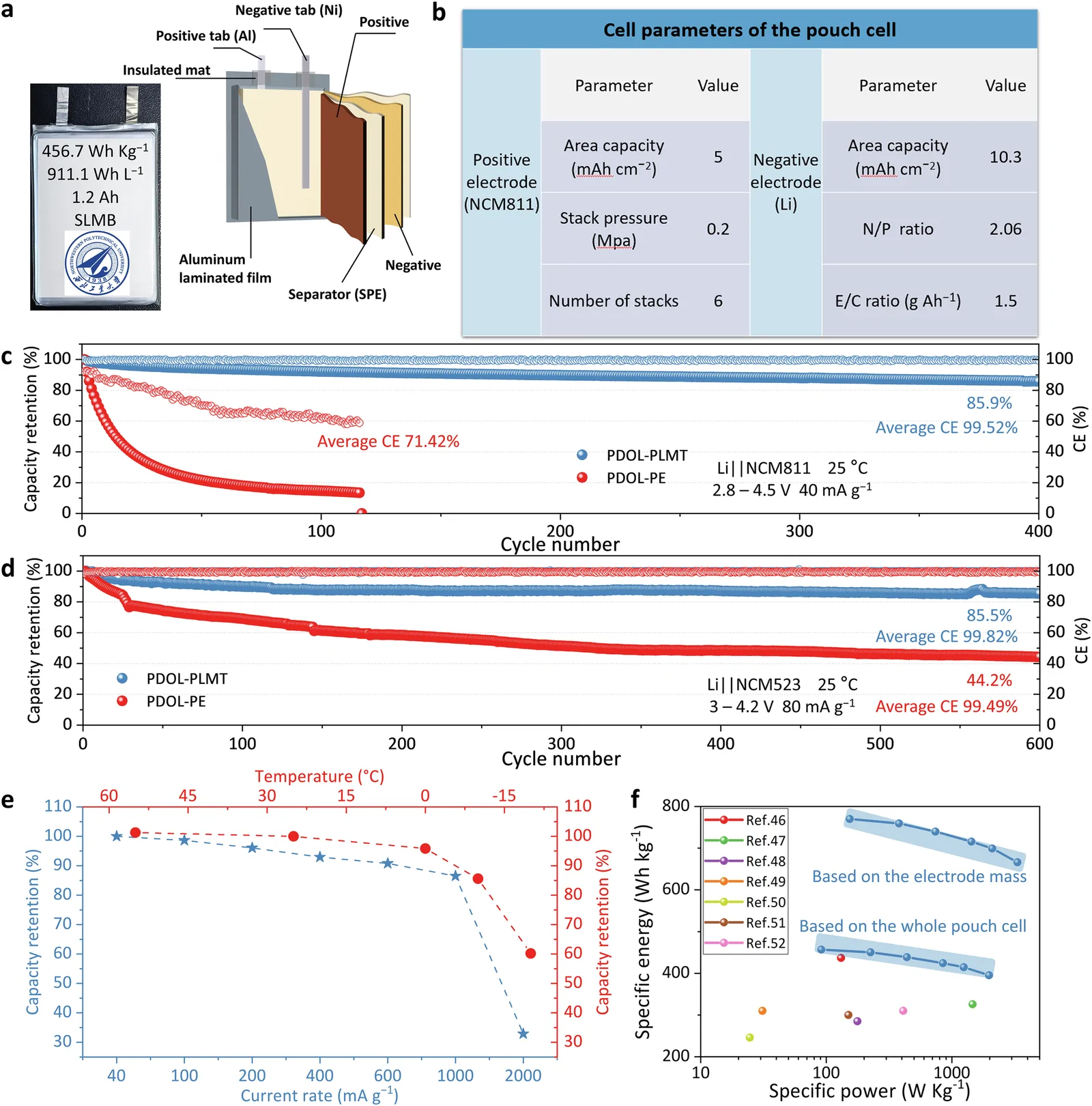
The electrochemical performance of various pouch-format cell prototypes. **a** Optical image of the 1.2 Ah pouch cell and corresponding schematic illustration of the assembled components. **b** Specific parameters of the 1.2 Ah pouch cell. **c** Cycling stability of Li||NCM811 pouch cells with PDOL-PE and PDOL-PLMT SPEs within 2.8–4.5 V (25 °C, 1C = 200 mA g^−1^). **d** Cycling performance of Li||NCM523 pouch cells with PDOL-PE and PDOL-PLMT SPEs within 3.0–4.2 V (25 °C, 1C = 160 mA g^−1^). **e** Specific current capability and wide-temperature-range performance of the Li|PDOL-PLMT|NCM811 pouch cell. **f** Ragone plot of the Li|PDOL-PLMT|NCM811 cell prototype and the key performance metrics as compared to other NCM811-based pouch cells reported in the literature (All gravimetric energy densities are calculated based on the total mass of the pouch cell).

To validate the practical viability of the solid-state battery prototypes (Supplementary Table 2 and 3), we assembled a series of 1.2 Ah pouch cells by pairing the 100 μm Li negative electrode with a high-mass-loading NCM811 positive electrode (25 mg cm^−2^, double-sided, Fig. 6a). Systematically validated using batch-produced cells (Supplementary Figs. 35 and 36), the Li|PDOL-PLMT|NCM811 configuration exhibited durable cycling attributable to the ultrathin SPE and stabilized interfaces across the scales. Upon galvanostatic cycling within 2.8–4.5 V, the cell prototype maintained 85.9% capacity retention for 400 cycles (Fig. 6c and Supplementary Fig. 37), while simultaneously achieving specific energy/energy densities of 456.7 Wh kg^−1^/911.1 Wh L^−1^ (based on the total mass and volume of the pouch cell, respectively). In contrast, the Li|PDOL-PE|NCM811 pouch cell showed rapid capacity deterioration and suffered from short-circuiting after only 117 cycles. To prove the generic applicability of PDOL-PLMT-based cell configuration, we assembled a series of Ah-scale pouch cells pairing various LiCoO_2_ (LCO), LiFePO_4_ (LFP), and LiNi_0.5_Co_0.2_Mn_0.3_O_2_ (NCM523, Supplementary Fig. 38) positive electrode chemistries with the PDOL-PLMT SPE and thin-layer Li foil. The Li|PDOL-PLMT|LFP prototype maintained 98.9% capacity retention after 500 cycles (Supplementary Fig. 39). Additionally, the Li|PDOL-PLMT|LCO and Li|PDOL-PLMT|NCM523 prototypes demonstrated excellent long-term stability, retaining 99.2% capacity after 300 cycles (Supplementary Fig. 40) and 85.5% after 600 cycles (Fig. 6d), respectively. The Li|PDOL-PLMT|NCM811 pouch cell also delivered stable specific current capability, maintaining 98.6%, 96.1%, 93.0%, 90.8%, 86.5%, and 32.9% of its 40 mA g^−1^ capacity at 100, 200, 400, 600, 1000, and 2000 mA g^−1^, respectively. Furthermore, the cell exhibited stable operation across a broad temperature range, with capacity retentions of 101.3% at 55 °C, 95.8% at 0 °C, 85.6% at −10 °C, and 60.2% at −20 °C, all relative to its capacity at 25 °C (Fig. 6e). As summarized in the Ragone plot^46–52^ (Fig. 6f), the Li|PDOL-PLMT|NCM811 pouch cell achieved a specific energy of 456.7 Wh kg⁻^1^ and an energy density of 911.1 Wh L⁻^1^ (based on the total mass and volume of the pouch cell, Supplementary Table 4), alongside a specific power of 1976.0 W kg⁻^1^ at the device level (Supplementary Table 5).

**Fig. 7:**
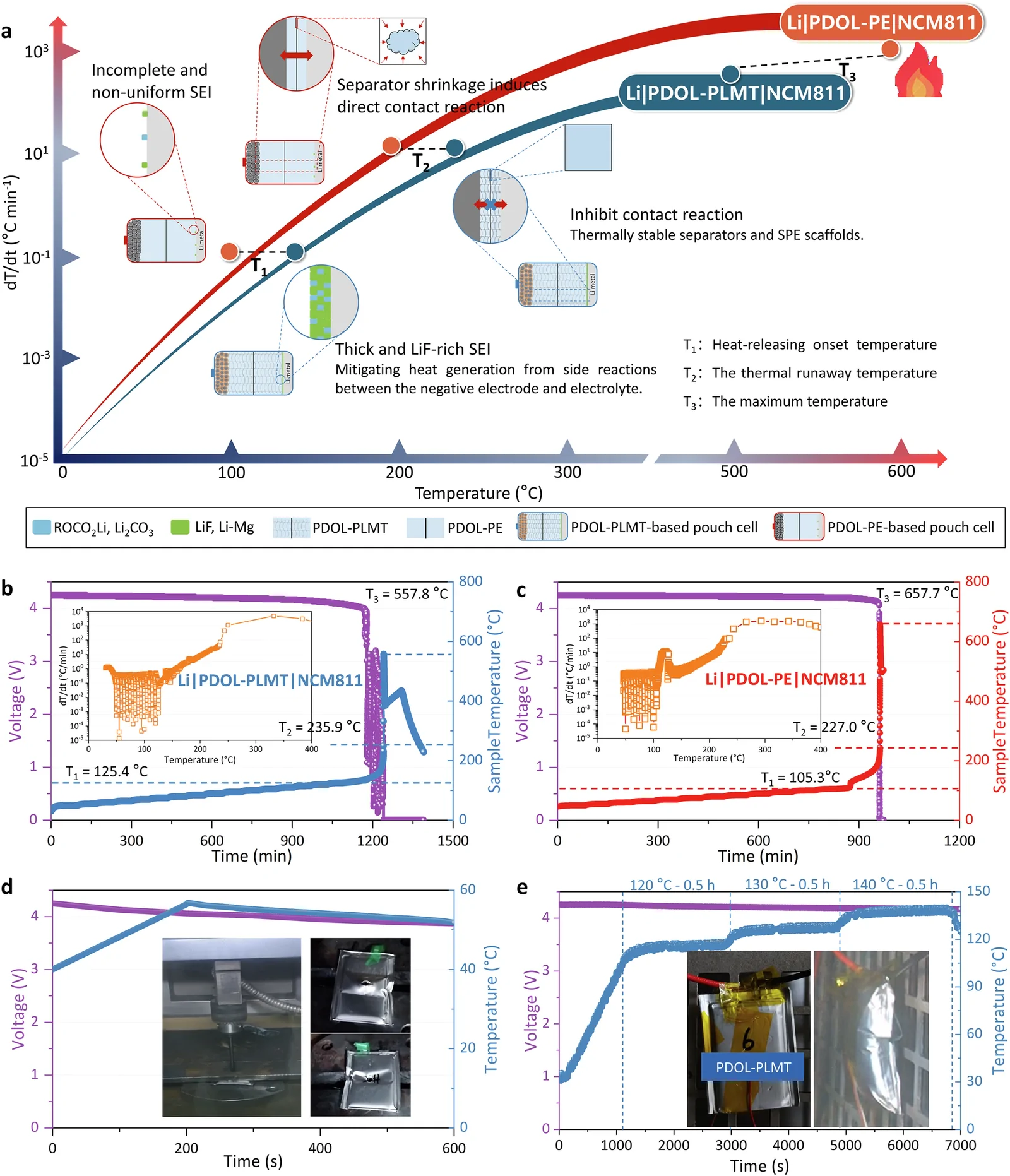
Quantification and in-depth analysis of thermal runaway process for pouch-format cells. **a** Schematic illustrating the safety protocol of the catalytic in-situ polymerization and dual-additive strategies for the PDOL-PLMT equipped SLMB. ARC tests of fully-charged 1.2 Ah Li||NCM811 prototypes with **b** PDOL-PLMT and **c** PDOL-PE and corresponding thermal runaway analysis. **d** Nail penetration test and **e** thermal chamber test of 1.2 Ah Li|PDOL-PLMT|NCM811 pouch cells according to the Chinese national standard GB 31241-2022 and GB 38031-2020.

Critically, the safety of the SLMB configuration was evaluated using a comprehensive suite of industry-standard tests, which elucidate the crucial effect of the PDOL-PLMT on the thermal runaway inhibition even under extreme abuse conditions. ARC was employed to characterize thermal propagation, tracking the onset (T_1_), trigger (T_2_), and maximum temperatures (T_3_), as well as the self-heating rate (dT/dt) during the transition from self-heating to thermal runaway (Fig. 7a). The Li|PDOL-PLMT|NCM811 pouch cell exhibited significantly higher T_1_ (125.4 °C) and T_2_ (235.9 °C) than the Li|PDOL-PE|NCM811 reference (105.3 °C and 227.0 °C, respectively; Fig. 7b, c). This improvement originates from the LATP-catalyzed in-situ polymerization and the stable interphase obtained during formation cycling, which collectively reduce the content of residual reactive monomers and suppress parasitic reactions at the Li surface. The hybrid PDOL-PLMT also enhances thermal and mechanical integrity, delaying internal short circuits caused by thermal shrinkage. Consequently, the thermal runaway trigger point is substantially delayed. Upon further heating, the polymer network eventually melts, compromising the SPE integrity and leading to thermal runaway. Post-mortem analysis confirms this thermal barrier effect: the PDOL-PE-based cell suffered complete degradation and severe deformation (Supplementary Fig. 41a, b), whereas its PDOL-PLMT counterpart retained notable structural integrity (Supplementary Fig. 41c, d). This aligns with ARC results (Fig. 7b, c), which show a significantly lower T_3_ for the Li|PDOL-PLMT|NCM811 (557.8 °C) than for that of Li|PDOL-PE|NCM811 (657.7 °C). Collectively, this rigorous thermal abuse testing confirms the PDOL-PLMT framework as a robust thermal barrier, delaying ~20 °C threshold onset temperature and suppressing peak temperature by ~100 °C. This integrity-preserving strategy mitigates the risk of catastrophic failure, providing a critical pathway to intrinsically safe, high-energy batteries.

Finally, the safety protocol of the 1.2 Ah Li|PDOL-PLMT|NCM811 pouch cell with the industry-standard benchmarks (GB 31241-2022 and GB 38031-2020) was conducted. In the nail penetration test, a fully charged pouch cell (100% SOC) was punctured by a 5 mm steel nail at 25 mm s^−1^, while the temperature at the puncture site was monitored in real-time. The Li|PDOL-PLMT|NCM811 cell passed the test without thermal runaway, smoke, explosion, or post-test venting/leakage. Upon the nail penetration, the cell’s voltage only slightly dropped from 4.25 to 3.86 V, and the temperature peaked at 56.8 °C after 202 s (Fig. 7d). The PDOL-PLMT effectively suppressed the internal short circuit, thereby preventing a sudden temperature surge. In stark contrast, the Li|PDOL-PE|NCM811 pouch cell ignited during the nail penetration, releasing substantial smoke and flames. Within 20 s of nail penetration, the cell’s voltage plummeted to 0 V, and its temperature soared to 474.7 °C (Supplementary Fig. 42). Furthermore, pouch cells based on PDOL-PE exhibited pronounced swelling when charged to 4.3 V during the formation cycle (Supplementary Fig. 43), indicative of severe interfacial instability and gas generation from SPE decomposition. Under thermal abuse at 140 °C (GB 31241-2022, Fig. 7e), the Li|PDOL-PE|NCM811 prototype experienced a rapid voltage drop from 4.25 to 0.63 V (Supplementary Fig. 44), accompanied by severe swelling and package rupture. In contrast, the Li|PDOL-PLMT|NCM811 prototype maintained voltage stability with only minor, localized swelling. Additionally, the mechanical abuse tolerance of the fully charged (100% SOC) prototype was evaluated (in accordance with Chinese national standard GB 38031-2020). The 1.2 Ah Li|PDOL-PLMT|NCM811 prototype retained its structural integrity after being impacted by a 9.1 kg mass dropped from a height of 610 mm (Supplementary Fig. 45). Conversely, the Li|PDOL-PE|NCM811 pouch cell swelled upon impact (Supplementary Fig. 46), signaling an internal short circuit that generated substantial Joule heat and initiated electrolyte decomposition. These results confirm that the PDOL-PLMT SPE effectively mitigates both thermal and mechanical abuse risks, substantially enhancing overall cell safety.

## Discussion

In summary, this work develops a scalable in-situ polymerization strategy for lithium-metal battery prototypes, balancing high specific energy and power with cycling endurance. Concurrently, the fundamental role of interfacial stabilization in suppressing thermal runaway is elucidated. The 9.6-μm-thick SPE membrane features an in situ polymerized PDOL matrix confined within a PE/LATP scaffold, delivering a mechanical strength of 191.7 MPa and an ionic conductance of 418.7 mS at 25 °C. Within this architecture, LATP nanoparticles function as a Lewis acid catalyst to achieve a DOL-to-PDOL conversion rate of 99.1%. At the positive electrode interface, the TFPP-derived CEI mitigates electrolyte decomposition and the TM cation crossover effect, thereby supporting stable cell operation up to 4.8 V. At the negative electrode, a lithiophilic Li–Mg alloy derived from the Mg(TFSI)_2_ additive lowers the Li⁺ diffusion barrier and guides homogeneous Li deposition. When integrated into a 1.2 Ah Li||NCM811 pouch cell, the system achieves a specific energy of 456.7 Wh kg⁻^1^ and an energy density of 911.1 Wh L⁻^1^ (based on the total mass and volume of the pouch cell, respectively), alongside a high specific power of 1976.0 W kg⁻^1^ and 85.5% capacity retention after 600 cycles. Notably, the PDOL-PLMT SPE physically suppresses detrimental negative electrode reactions, mitigating thermal runaway risks and enabling the cell to pass industry-standard thermal and mechanical abuse tests. Beyond detailing the mechanisms of interfacial stabilization, this work presents a methodology for cell prototyping: utilizing engineered in-situ polymerized electrolytes to suppress exothermic reactions, thereby charting a clear pathway toward safe, high-energy SLMBs.

## Methods

### Materials

1,3-Dioxolane (DOL), containing approximately 75 ppm of butylated hydroxytoluene as an inhibitor and with a purity of 99.8%, was acquired from Suzhou DuoDuo Chemical Technology. Lithium bis (trifluoromethanesulfonyl)imide (LiTFSI) (99% purity), TFPP (98% purity), LiOH·H_2_O (analytical reagent grade, 98% purity), γ-Al_2_O_3_ (99.9% metal basis, y-phase, 10 nm particle size), NH_4_H_2_PO_4_ (analytical reagent grade, 99% purity), and TiO_2_ (≥99.0% purity) were obtained from Aladdin. Al(OTf)_3_ (99.9% trace metal basis), isopropanol (IPA) (analytical reagent grade, ≥99.5% purity), and Mg(TFSI)_2_ (>97.0% purity) were sourced from Macklin. Polycrystalline NCM811, acetylene black, and PVDF (average molecular weight Mw ≈ 1,000,000 g mol⁻¹) were purchased from Guangdong Canrd New Energy Technology Co., Ltd., China. Both the lithium metal chips (15.8 mm in diameter, 1 mm in thickness) and the lithium foil (100 mm in width, 100 μm in thickness) were procured from China Energy Lithium Co., Ltd.

### Preparation of nano LATP particles

Precursors including γ-Al_2_O_3_, TiO_2_, NH_4_H_2_PO_4_, and LiOH·H_2_O were dispersed in IPA to synthesize stoichiometric Li_1.3_Al_0.3_Ti_1.7_(PO_4_)_3_ (LATP). The suspension was mechanically alloyed in a planetary ball mill (MSK-SFM-1, Shenzhen Kejing) at 400 rpm for 6 h, employing a dual-size combination of YSZ beads (100 g of 10-mm diameter and 350 g of 5-mm diameter) to ensure efficient pulverization. Subsequently, the dried powders were calcined at 950 °C for 6 h within a saggar containing approximately 80 g of the LATP precursor powder to yield pure rhombic-phase LATP powders. The powders were then sieved through an 80-mesh screen and subjected to an additional ball-milling process at 500 rpm for 24 h to refine the powder properties.

### Preparation of the SPE membrane

Preparation of DOL precursor: First, 5 mM Al(OTf)_3_ was dissolved in a 1 M LiTFSI/DOL solution under vigorous stirring for 20 min. Subsequently, the designated additives–TFPP (1 wt%) and/or Mg(TFSI)_2_ (3 wt%) were incorporated under continuous stirring. The resulting precursor mixture underwent in-situ polymerization at 25 °C, yielding a cross-linked PDOL polymer electrolyte. All synthesis procedures were conducted within an argon-filled glovebox with water and oxygen levels maintained below 0.1 ppm.

### Battery assembly and electrochemical measurements

Standard CR2016 coin cells were assembled utilizing lithium foil negative electrodes and PE or PE/LATP separators, which regulated the thickness of the in situ polymerized SPEs and prevented internal short circuits during the liquid phase. Each cell was injected with 40 μL of liquid precursor and aged at 25 °C for 24 h to ensure complete polymerization. The NCM811 positive electrode was fabricated by casting a slurry of polycrystalline NCM811, acetylene black, and PVDF (80:10:10 weight ratio) onto aluminum foil, yielding an active material mass loading of ~5–6 mg cm^−2^. The positive electrodes were punched into 12 mm discs and stored in an argon-filled glovebox prior to use. Unless otherwise noted, all electrochemical measurements were conducted at 25 °C using a NEWARE CT-4008 battery testing system.

### Assembly and formation protocol of the pouch cells

The detailed design and assembly parameters of the pouch cells, including the specific volume of the injected precursor, electrode dimensions, and the exact number of layers, are summarized in Supplementary Tables 2 and 3.

The protocol is designed to ensure complete wetting, controlled polymerization, and gas removal: After electrolyte injection and pre-sealing, the pouch cells were kept at 45 °C for 48 h. This step ensures the complete infiltration of the precursor into the electrode pores and the completion of the in situ polymerization reaction prior to electrochemical charging. We adopted a multi-step formation strategy. During this stage, a uniform stack pressure of 0.5 MPa was applied to the fixture to ensure dense interface formation.

### Li||NCM811 pouch cells

Constant current (CC) charge at 0.05 C to 3.8 V, rest for 10 min.

CC charge at 0.1 C to 4.5 V, followed by constant voltage (CV) holding until current drops below 0.02 C.

Rest for 10 min, then CC discharge at 0.1 C to 2.8 V.

### Li||NCM523 pouch cells

Similar protocol with a cutoff voltage of 4.2 V (0.05 C to 3.8 V → 0.1 C/CV to 4.2 V → Discharge to 3.0 V).

### Li||LFP pouch cells

Protocol adapted for LFP (0.05 C to 3.3 V → 0.1 C/CV to 4.0 V → Discharge to 2.5 V).

### Li||LCO pouch cells

Protocol adapted for high-voltage LCO (0.05 C to 3.8 V → 0.1 C/CV to 4.4 V → Discharge to 3.0 V).

Critically, after the formation cycles, the cells were transferred to an Ar glovebox for degassing (gas bag removal) and secondary sealing. This step effectively removes any gaseous byproducts generated from the sacrificial oxidation of TFPP, preventing cell swelling and ensuring compact assembly. Subsequent long-term cycling tests were conducted under a controlled stack pressure of 0.2 MPa to accommodate volume expansion while maintaining interface contact.

### Materials characterization

FTIR transmittance spectra of the PDOL polymer electrolyte were acquired using a Nicolet Nexus 5700 spectrometer (Thermo Electron Corporation, USA). The morphologies of PE/LATP and the in-situ polymerized SPEs were examined using a ZEISS GeminiSEM 500 SEM. Tensile stress-strain measurements of the SPEs were conducted on an Instron 3342 tensile tester, with an elongation rate of 10 mm min^−1^. The PDOL and PDOL-LATP samples were dissolved in chloroform-d and subsequently analyzed using ^1^H NMR and ^13^C NMR spectroscopy on a Bruker Avance NEO 600 system. An ex-situ XRD analysis was conducted using a Bruker D8 DISCOVER system with Cu Kα radiation to identify the phases of LATP. The scan range was set from 10° to 60° with a step size of 0.02°. For operando XRD measurements, a transmission-mode X-ray diffractometer (STADIP STOE) equipped with a Mo Kα1 radiation source (*λ* = 0.70930 Å, operated at 50 kV and 40 mA) was employed. The SPE and Li foil were configured within a single-layer pouch cell, mounted on a specialized specimen holder (HT-SPB-XRD, Beijing Scistar Technology Co. Ltd.). The cell terminals were connected to a battery testing system to perform GCD procedures at a current density of 0.2 mA cm^−2^. Moreover, operando XRD measurements were taken with a step scan of 1.05° and variable step times, ensuring that the selected range was scanned every 7–8 min. XPS spectra of the NCM811 positive electrodes and lithium foils, as well as the surface of the SPE membrane before and after cycling, were acquired using a PHI 5000 VersaProbe III instrument. The CEI on the cycled NMC811 positive electrodes was investigated with a TEM (FEI Talos F200X). Elemental analysis and depth profiles of the SEI were determined by TOF-SIMS (PHI nano TOF II). The sputtered area for TOF-SIMS analysis was 100 × 100 μm^2^. Ion etching was conducted using an Ar^+^ ion beam with a current of approximately ~2 nA for 30 s per cycle. The depth profiles were processed and analyzed with ION-TOF software.

### Electrochemical measurements

The ionic conductivities of PDOL-PE and PDOL-PE/LATP electrolytes were measured using an electrochemical impedance spectroscopy (EIS) method. Potentiostatic EIS measurements were conducted using a Gamry Reference 600. To ensure a quasi-stationary state, the cells were rested at open-circuit voltage for 10 s prior to the test. The impedance spectra were recorded in the frequency range from 1 MHz to 1 Hz with an AC voltage amplitude of 10 mV, collecting 10 data points per decade. The stainless-steel (SS)|SPE|SS cells were employed to obtain the total resistance of various electrolytes at temperatures ranging from 22 to 60 °C. And the corresponding ionic conductivity (*σ*) can be calculated by using the following Eq. (1):

$${{{\rm{\sigma }}}}=\frac{L}{R\times S}$$

where *L* is the thickness of the electrolyte membrane, *S* represents the area of the SS electrode, and *R* is the measured resistance.

The activation energies (*E*_a_) of PDOL-PE and PDOL-PE/LATP electrolytes were calculated by fitting ionic conductivity using the Arrhenius equation (2):

$$K=A{e}^{-\frac{{E}_{a}}{{RT}}}$$

The Li^+^ migration number (t_Li_^+^) of SPE was obtained by the chronoamperometric method and the EIS method. Before measurement, the SPE was assembled into a Li symmetric cell and rested for 12 h. Then, the obtained Li|SPE|Li cells were used to perform a potentiostatic polarization test at an applied voltage of 10 mV. The t_Li_^+^ was calculated from BruceVincent–Evans equation (3):

$${t}_{{{{{\rm{Li}}}}}^{+}}=\frac{{I}_{{ss}}}{{I}_{0}}\times \frac{\Delta V-{I}_{0}\times {R}_{0}}{\Delta V-{I}_{{ss}}\times {R}_{{ss}}}$$

Where *I*_0_ and *I*_SS_ are initial and steady-state current, respectively. Δ*V* is the applied polarization voltage. *R*_0_ and *R*_SS_ are the interfacial resistance before and after the polarization process, respectively.

The electrochemical stability windows of PDOL-PE, PDOL-PE/LATP and PDOL-PE/LATP-TFPP electrolytes were evaluated via LSV using a SS working electrode and a Li counter/reference electrode at a scan rate of 0.1 mV s^–1^. Electrochemical floating tests were performed on Li||NCM811 cells using various SPEs. The cells were initially charged to 4.3 V, followed by a series of potentiostatic steps from 4.3 to 4.8 V, with each voltage held for 10 h to monitor the resultant leakage current. To investigate ion transport kinetics, Galvanostatic Intermittent Titration Technique (GITT) measurements were conducted using a Neware battery testing system. The GITT protocol consisted of a series of current pulses at 0.1 °C for 30 min, each followed by a 30-min relaxation period; this sequence was applied continuously during charging to 4.3 V and subsequent discharging to 2.8 V (vs. Li/Li⁺). The Li^+^ diffusion coefficient (D_Li_^+^) can be calculated according to the following Eq. (4):

$${D}_{{Li}^{+}}=\frac{4}{\pi \tau }{\left(\frac{{m}_{{NCM}811}{V}_{{NCM}811}}{{M}_{{NCM}811}S}\right)}^{2}{\left(\frac{\Delta {E}_{s}}{\Delta {E}_{\tau }}\right)}^{2}$$

in which *m*_NCM811_ is the mass of active substance on electrode (g); *M*_NCM811_ is molar mass (g mol^−1^); *V*_NCM811_ is molar volume (cm^3^ mol^−1^); S is area of electrode plate (cm^2^); *τ* stands for the relaxation time (s), Δ*E*_s_ represents the steady-state potential change via the current pulse, and Δ*E*_t_ is the potential change in current pulse after a 30 min charge/discharge process.

### Distribution of relaxation time (DRT) analysis

DRT technology is a technique used to decompose complex impedance spectra into a series of individual relaxation processes. This method allows for the representation of the electrochemical process in the time domain. It is particularly useful for interpreting the *τ* = RC (time constant) of a parallel Randles circuit, where *τ* is the specific relaxation time for each RC parallel circuit. The DRT analysis transitions the impedance data into a DRTs, which can be derived from the following formula (5):

$$Z\left(\omega \right)={R}_{\infty }+{\int }_{0}^{\infty }\frac{\gamma \left(\tau \right)}{1+j\omega \tau }d\tau$$

$Z\left(\omega \right)$ denotes the total impedance in the frequency domain. ${R}_{\infty }$ signifies the fundamental Ohmic resistance within the electrochemical system, while $\gamma \left(\tau \right)$ characterizes the distribution function of relaxation times associated with the impedance of a series of RC parallel circuits. The DRT analysis is performed using the DRT Tools, a MATLAB-based software suite developed by Ciucci. Group (https://ciucci.org/project/drt/).

### Computational details

The molecular geometries of DOL, PDOL, TFPP, LiTFSI, Al(OTf)_3_, H^+^(PF_5_OH)⁻, H^+^(BF_3_OH)⁻, and LATP were optimized at the B3LYP/6-31G(d) level of theory and verified as local energy minima on the potential energy surface. All of these theoretical calculations were performed using the Gaussian 16 software package. The ring-opening adsorption energies (*E*_(ad)_) of DOL on various initiators was calculated using the following Eq. (6):

$${{{{\rm{E}}}}}_{({{{\rm{ad}}}})}={{{{\rm{E}}}}}_{({{{\rm{DOL}}}}+{{{\rm{initiator}}}})}-{{{{\rm{E}}}}}_{({{{\rm{DOL}}}})}$$

Furthermore, the ESP distributions and frontier molecular orbital energy levels, specifically the HOMO and LUMO, were computed to comprehensively evaluate the thermodynamic oxidation limits and spatially resolve the preferential reaction sites of the additives.

Calculations of Li^+^ diffusion pathways and corresponding energy barriers were performed using the Vienna Ab initio Simulation Package with the Perdew-Burke-Ernzerhof functional within the generalized gradient approximation. The projector augmented wave method was employed to describe ionic cores, utilizing a plane-wave cutoff energy of 450 eV. Partial occupancies of Kohn−Sham orbitals were handled using Gaussian smearing with a width of 0.05 eV. The energy convergence criterion was set to 10^–5^ eV. Dispersion interactions were accounted for using Grimme’s DFT-D3 correction. For Brillouin zone integration, Monkhorst-Pack K-point sampling was applied to the Li (110) and Li_0.9_Mg_0.1_ (110) surfaces. Finally, the climbing-image nudged elastic band (CI-NEB) method was employed to determine diffusion barriers, with a force convergence threshold of 0.05 eV/Å.

Molecular dynamics (MD) simulations were systematically performed utilizing the GROMACS^53,54^ software package (version 2024.2) equipped with the all-atom OPLS (OPLS-AA) force field^55^. The initial atomic coordinates and topological parameters for the organic components—specifically the TFPP additive, the TFSI⁻ anion and the PDOL polymer chain (explicitly constructed with a degree of polymerization of 20 1,3-dioxolane monomers)-were generated through the LigParGen web server^56^. Standard Lennard-Jones parameters for the inorganic Li⁺ and Mg^2^⁺ cations were adopted from established OPLS-AA literature libraries. To accurately capture the electrostatic environment and molecular polarization, partial atomic charges were rigorously derived based on DFT geometry optimizations at the B3LYP/6-31G(d) level using the Gaussian 16 suite. Subsequently, the Multiwfn 3.8 code^57^ was utilized to assign Restricted Electrostatic Potential charges for the TFSI⁻ anions, while a scaled 1.2 × CM5 charge model was implemented for the organic species (PDOL and TFPP).

During the simulation trajectories, the equations of motion were integrated using a standard time step of 2 fs. To permit this time step, all bonds involving hydrogen atoms were constrained employing the LINCS algorithm. The cut-off radii for both van der Waals and short-range real-space electrostatic interactions were rationally set to 1.2 nm. Long-range electrostatic interactions were computed using the Particle Mesh Ewald method^58^. Throughout the equilibration and production runs, the system temperature and pressure were rigorously maintained using the velocity-rescaling (V-rescale) thermostat and the C-rescale barostat, respectively, ensuring a stable isothermal-isobaric ensemble.

## Supplementary information

Supplementary Information

Description of Additional Supplementary Files

Supplementary Data 1–7

Transparent Peer Review File

## Source data

Source Data file

## Data Availability

The source data generated in this study are provided in the Source Data file, and the Figshare database https://doi.org/10.6084/m9.figshare.31927650. Source data are provided with this paper.
