# Supplementary material for: A catalytically polymerized solid electrolyte enables 450 Wh kg−1 lithium–metal batteries with thermal–mechanical abuse tolerance
Source: Nat Commun. 2026 Aug 8;17:9545. doi: 10.1038/s41467-026-76381-y (PMC13547571; doi:10.1038/s41467-026-76381-y)
Supplement: Supplementary file 1 [file 41467_2026_76381_MOESM1_ESM.pdf]

Supplementary Information

A Catalytically Polymerized Solid Electrolyte Enables 450 Wh kg^{-1} Lithium–Metal Batteries with Thermal–Mechanical Abuse Tolerance

Jiawen Tang [1], Junyu Zhang [1], Jiacheng Liu [1], Yunsong Li [1], Ahu Shao [1], Zhiqiao Wang [1], Xin Wang [2], Qiurong Jia [3], Ting Liu [1], Zhe Liu [1], Jian-Gan Wang [1], Zhaohui Wang [4], Fei Xu [1], Yue Ma* [1]

[1] State Key Laboratory of Solidification Processing, Center for Nano Energy Materials, School of Materials Science and Engineering, Northwestern Polytechnical University, Xi'an 710072, P. R. China.

[2] Shaanxi Raisight Energy Tech Co., Ltd., Xi'an 710086, China

[3] Zhengzhou BAK Battery Co. Ltd., Zhengzhou, P. R. China.

[4] College of Materials Science and Engineering, Hunan University, Changsha, 410082 China

Corresponding author: mayue04@nwpu.edu.cn (Y. Ma)

Supplementary Figures

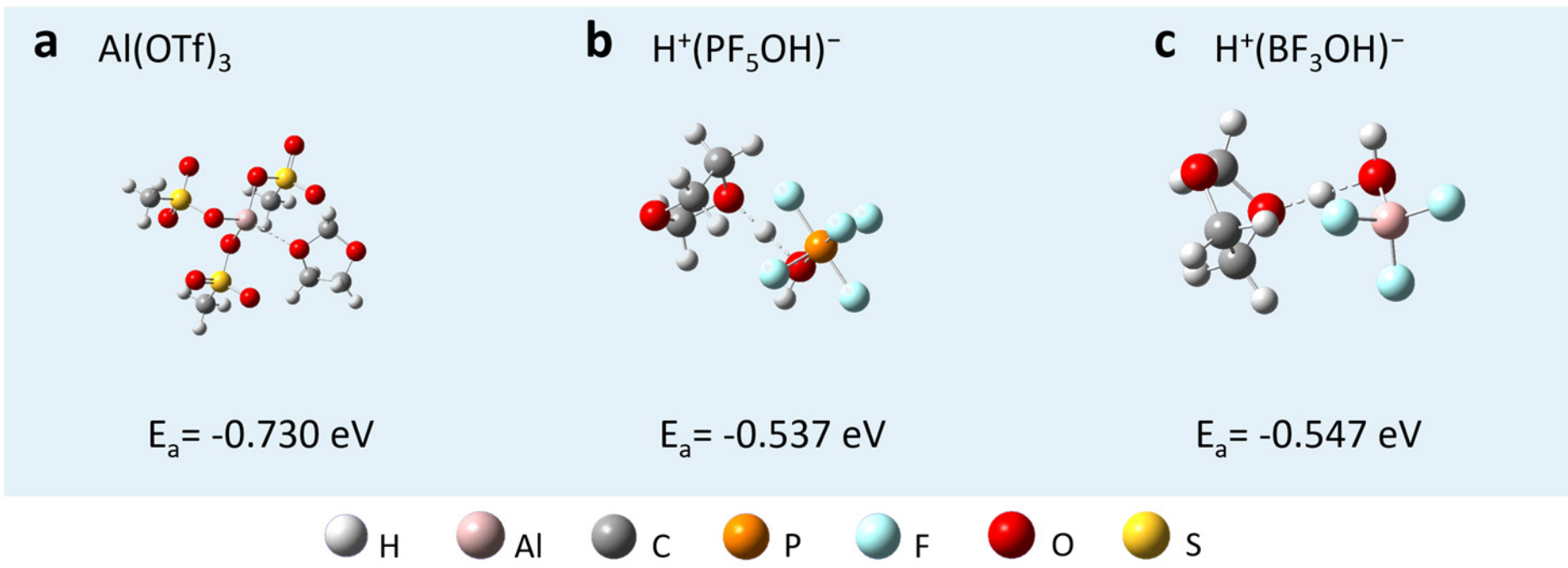

Supplementary Fig. 1 | Theoretical investigation of the DOL ring-opening mechanism catalyzed by different initiators. DFT calculated binding energies for the ring-opening process of DOL with (**a**) $Al(OTf)_3$, (**b**) $H^+(PF_5OH)^-$ and (**c**) $H^+(BF_3OH)^-$ initiators.

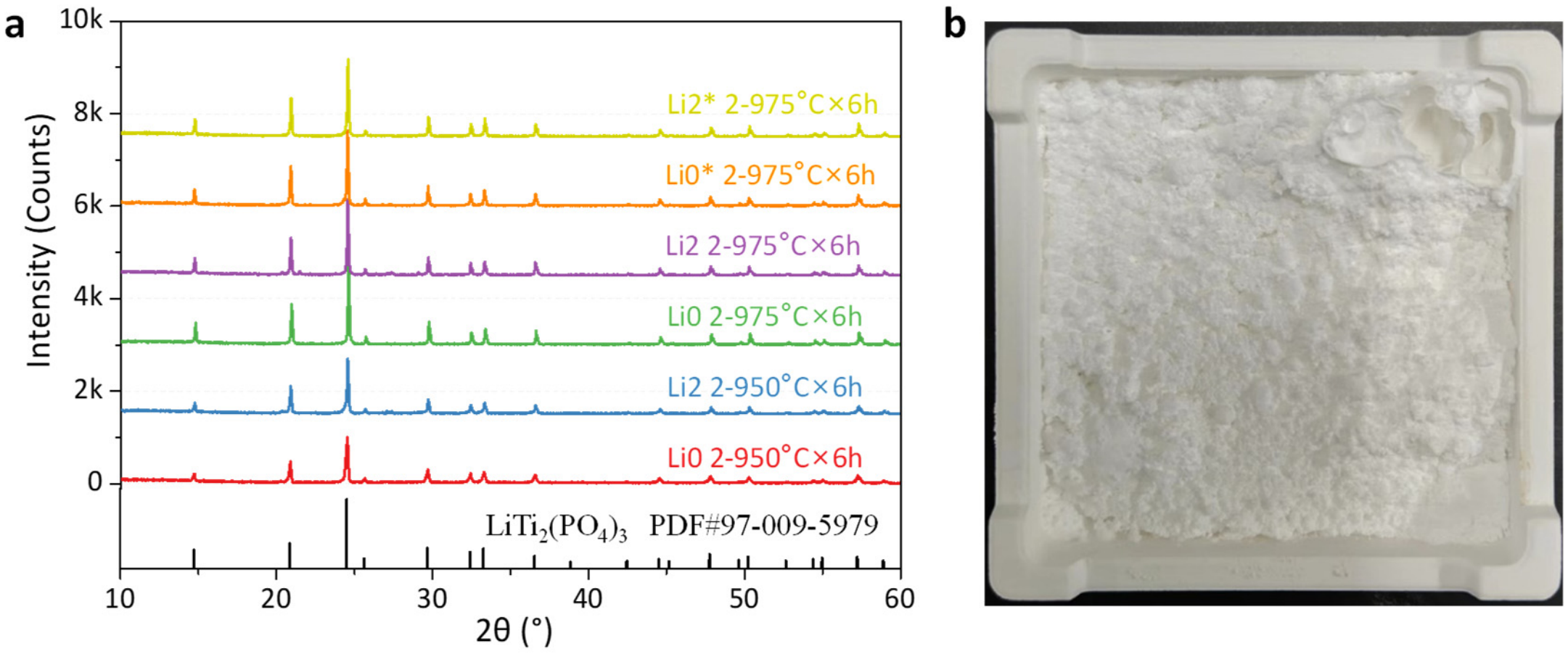

Supplementary Fig. 2 | Structural characterization and phase purity of the batch-synthesized LATP powders. a XRD patterns of LATP powders prepared in batches after calcination. The labels Li0 and Li2 denote stoichiometric and 2% excess lithium in the precursor, respectively. **b** Optical photographs of the calcined LATP powders.

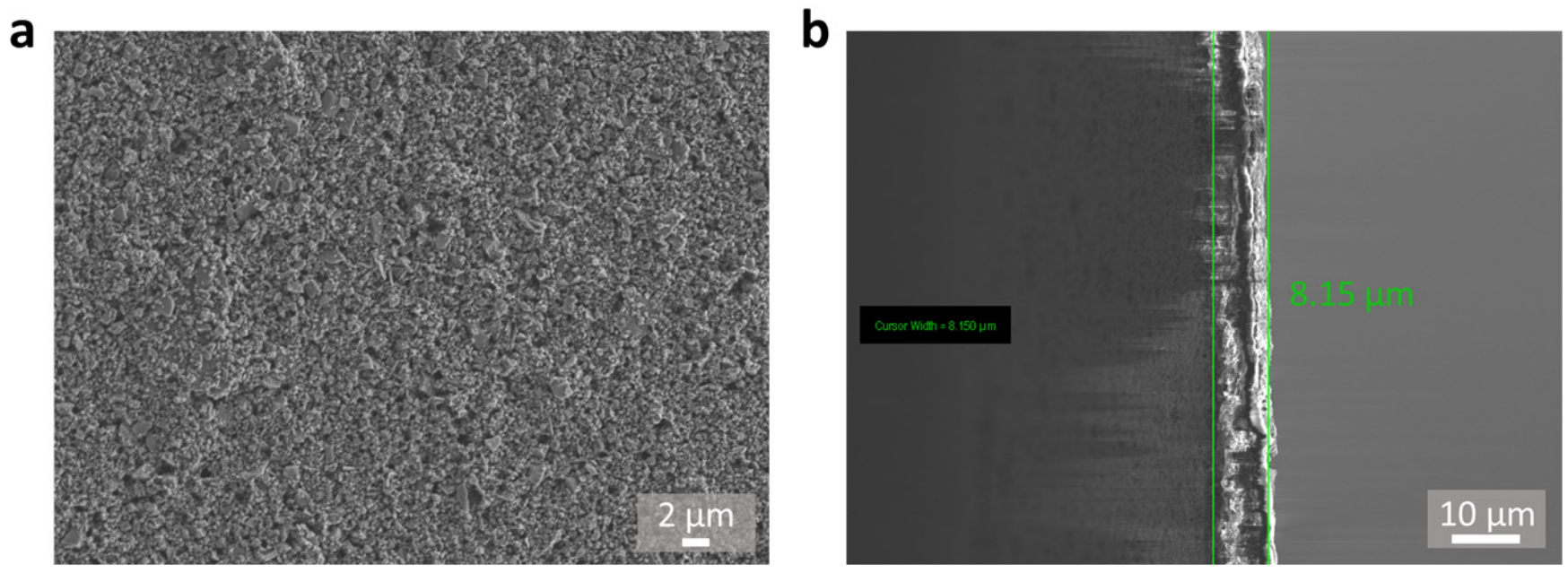

Supplementary Fig. 3 | Microstructural characterization of the PE/LATP composite membrane.

a Top-view and (**b**) cross-sectional SEM images of PE/LATP membrane.

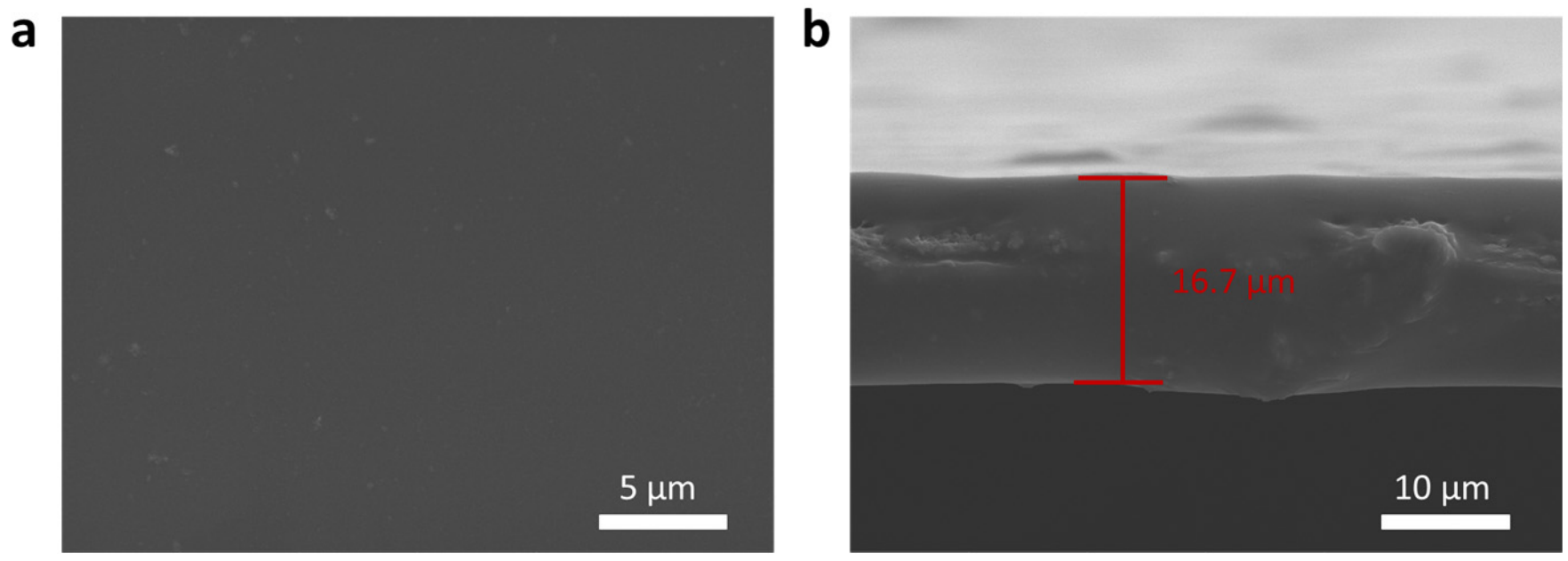

Supplementary Fig. 4 | Microstructural characterization of the PDOL-PE SPE. a Top-view and (**b**) cross-sectional SEM images of PDOL-PE electrolyte.

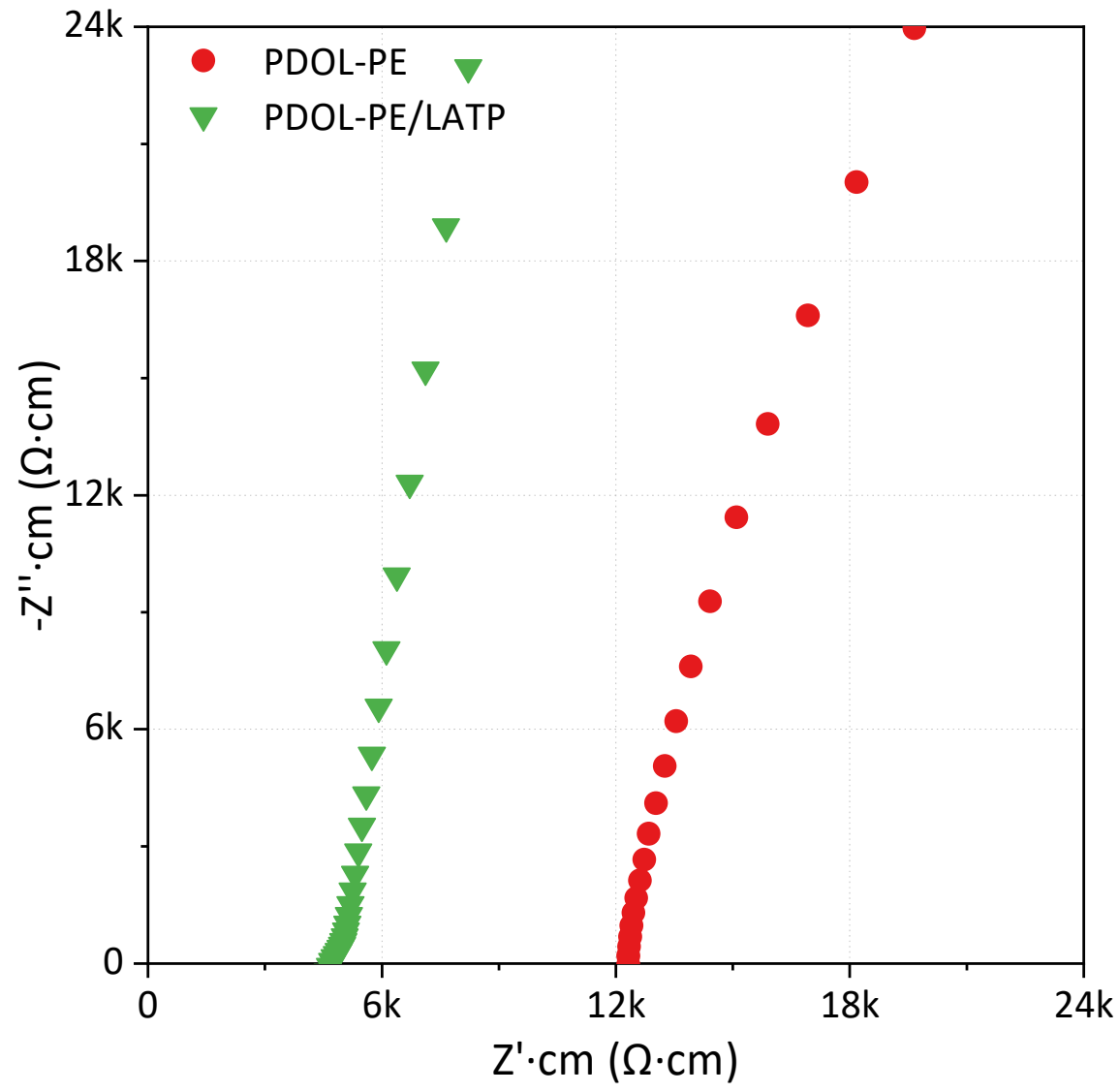

Supplementary Fig. 5 | Room-temperature ionic conductivity evaluation of the SPEs. Typical Nyquist plots of SS|PDOL-PE|SS and SS|PDOL-PE/LATP|SS symmetric cells at room temperature. The impedance data are normalized by the electrode area (8 mm × 8 mm × π) and the respective electrolyte thicknesses (16.7 μm for PDOL-PE and 9.6 μm for PDOL-PE/LATP). SS denotes stainless steel.

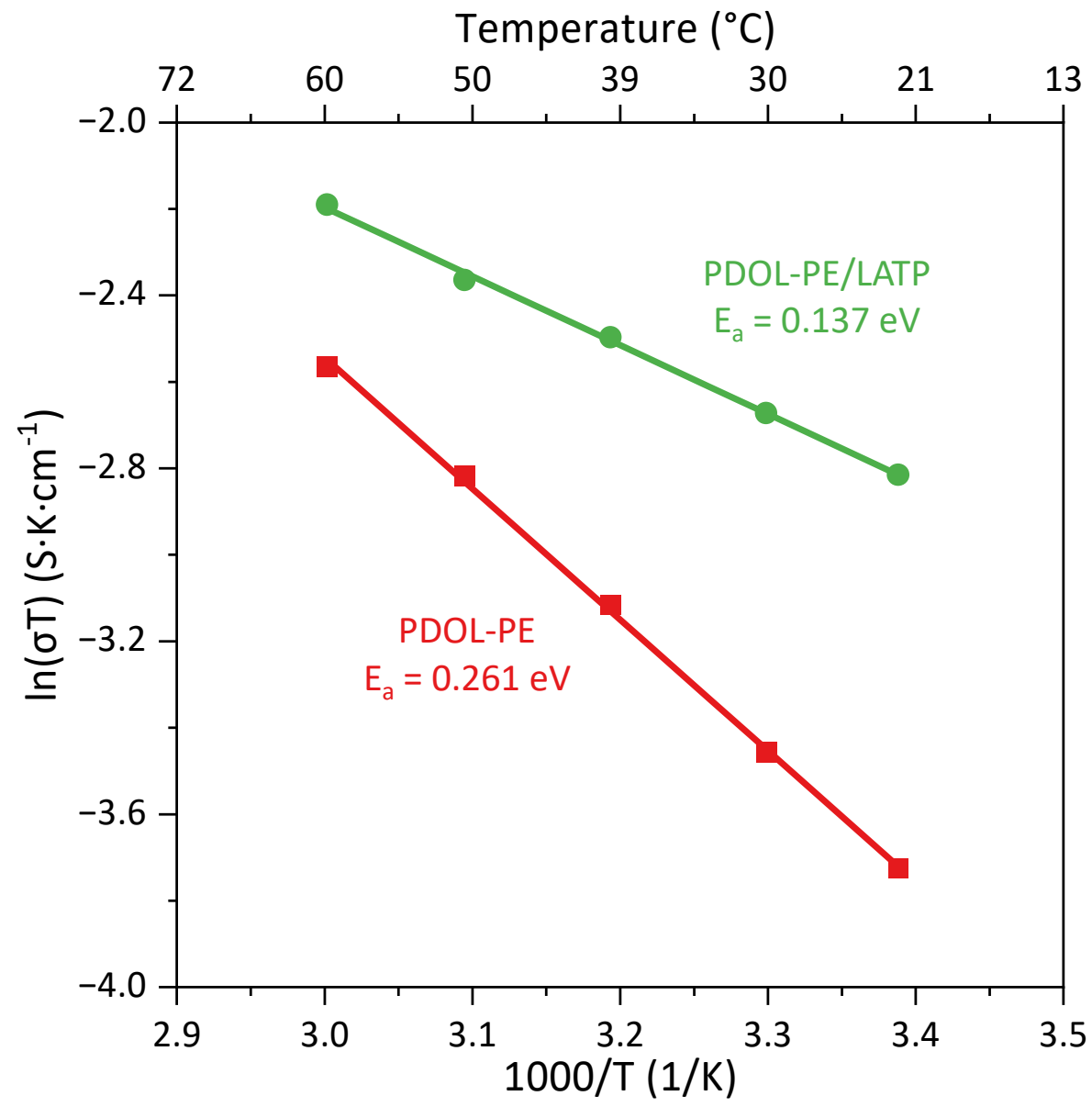

Supplementary Fig. 6 | Ion transport kinetics of the SPEs. Temperature-dependent ionic conductivities and the corresponding activation energies (E_a) of the PDOL-PE and PDOL-PE/LATP electrolytes.

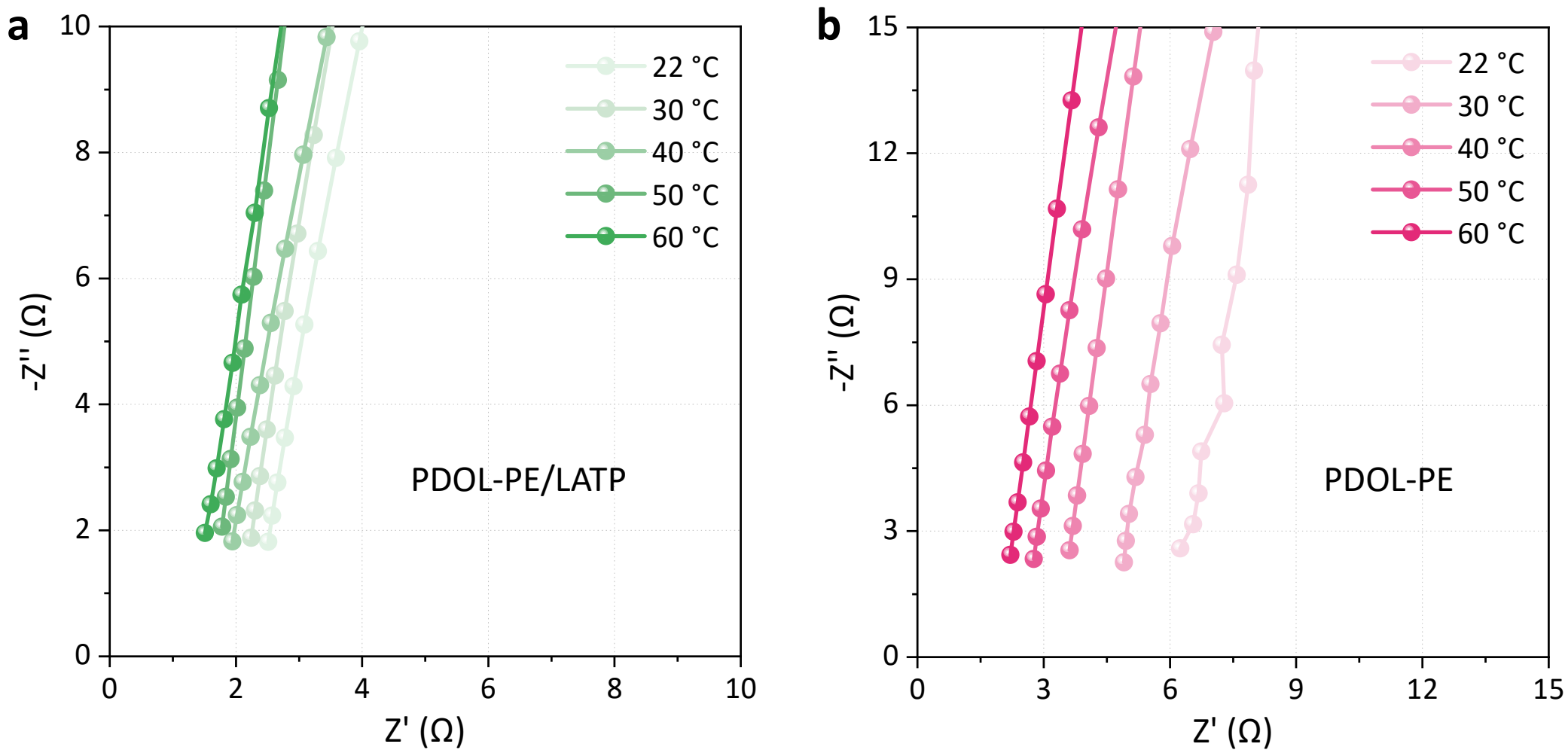

Supplementary Fig. 7 | Temperature-dependent EIS of the SPEs. The EIS curves of the (**a**) SS|PDOL-PE/LATP|SS and (**b**) SS|PDOL-PE|SS symmetric cells at different temperatures.

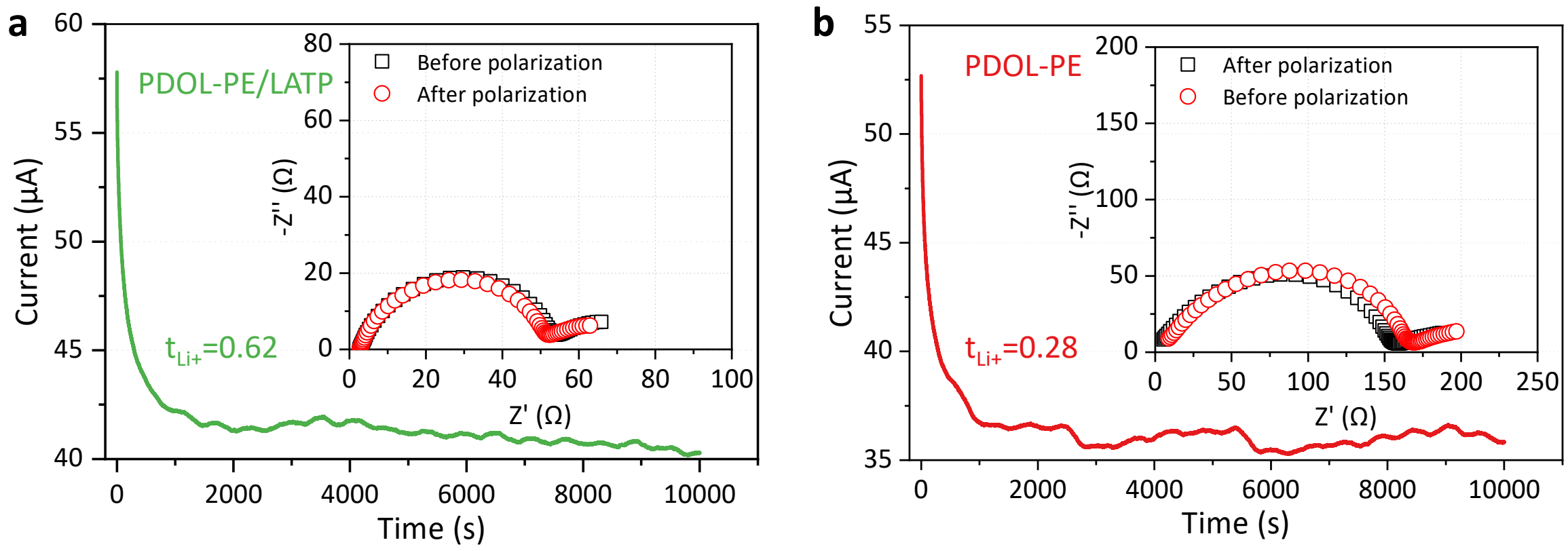

Supplementary Fig. 8 | Determination of the lithium-ion transference numbers (t_{Li+}). Potentiostatic polarization curves of the Li symmetric cells assembled with the (**a**) PDOL-PE/LATP and (**b**) PDOL-PE electrolytes. The insets display the corresponding electrochemical impedance spectra (Nyquist plots) measured before and after the DC polarization.

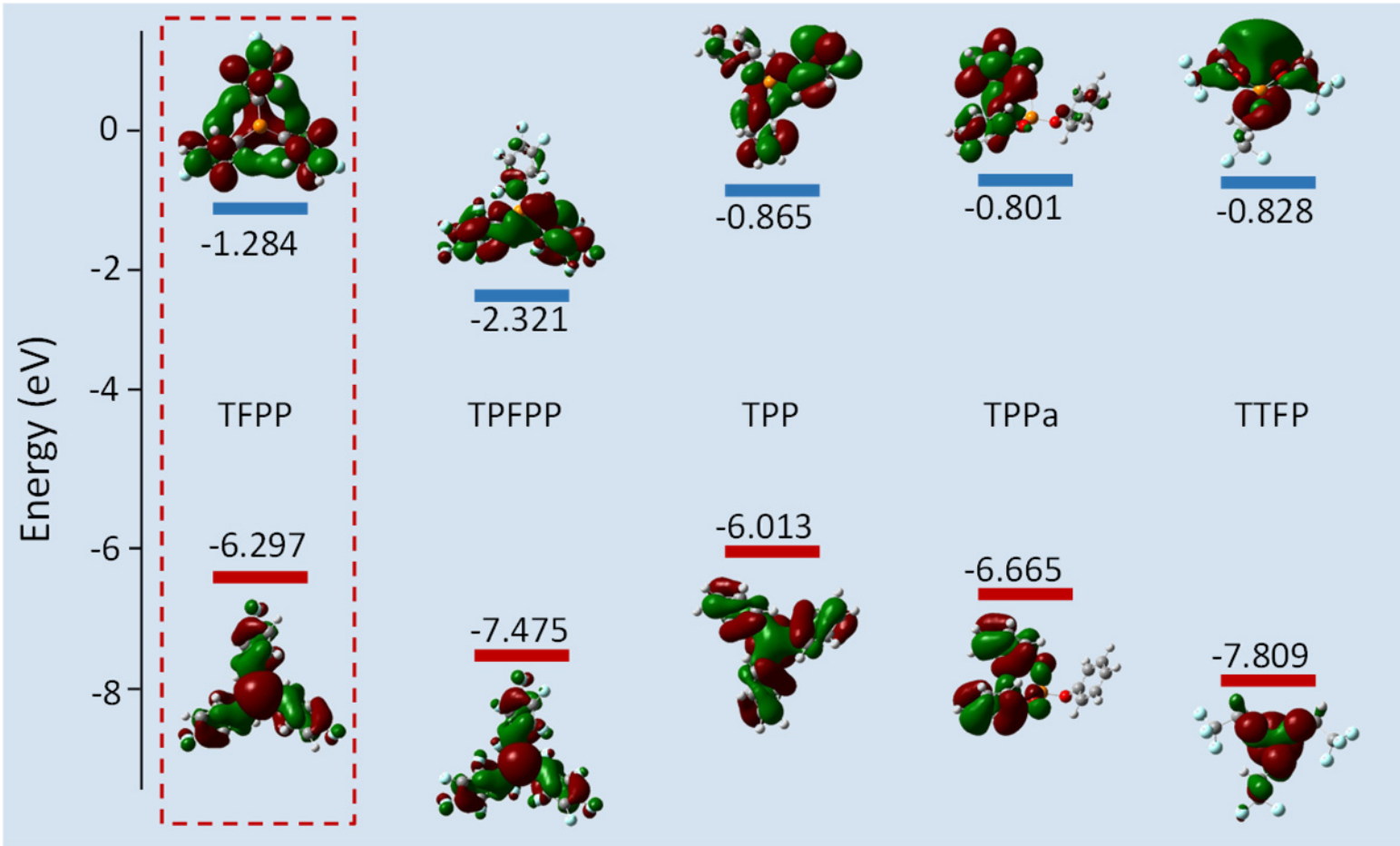

Supplementary Fig. 9 | Frontier molecular orbital energies of the investigated additives. Comparison of the HOMO and LUMO energy levels for the organophosphorus and fluorinated electrolyte additives.

Density Functional Theory (DFT) calculations were conducted to evaluate the thermodynamic suitability of TFPP against various organophosphorus and fluorinated benchmarks (Supplementary Fig. 9). The oxidative stability threshold of the electrolyte is dictated by the PDOL backbone (−6.711 eV) and LiTFSI salt (−6.613 eV). Effective positive electrode protection requires an additive to sacrificially oxidize before these baseline components. Benchmarks with deep HOMOs, such as TPFPP (−7.475 eV) and TTFP (−7.809 eV), are thermodynamically precluded from providing prior protection. Meanwhile, although TPP (−6.013 eV) fulfills the oxidation requirement, its lack of fluorine atoms results in a structurally deficient, purely organic CEI. In contrast, TFPP (−6.297 eV) strategically bridges these thermodynamic and structural requirements. It strikes a perfect balance by ensuring preferential oxidation over the polymer matrix while supplying the critical perfluoroalkyl groups necessary to construct a robust, F-rich CEI for high-voltage stabilization.

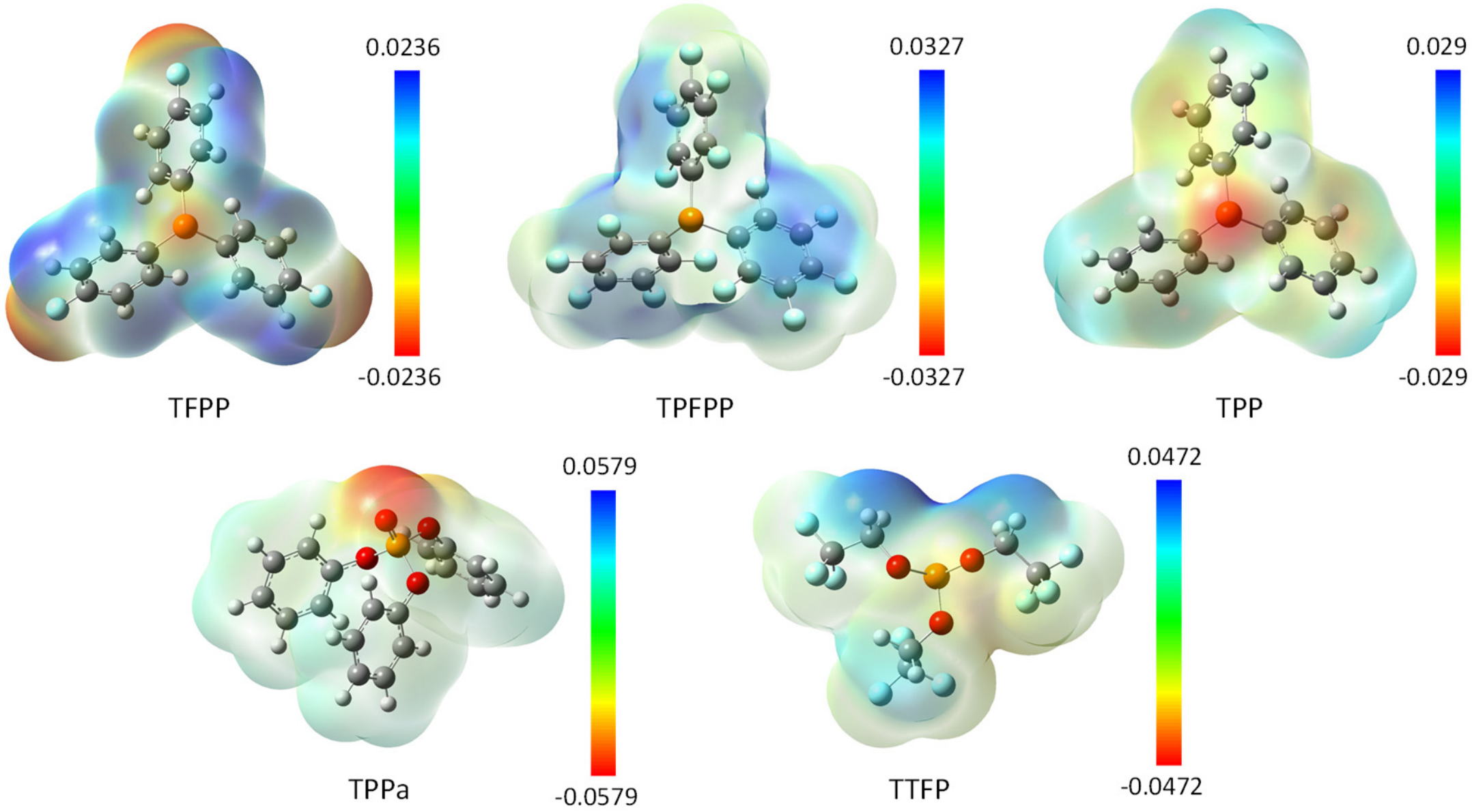

Supplementary Fig. 10 | Molecular charge distribution and identification of active interaction sites. Electrostatic potential (ESP) maps for the investigated additive molecules: TFPP, TPFPP, TPP, TPPa, and TTFP.

To further elucidate the spatial reactivity and initial decomposition mechanisms of the additives at the high-voltage positive electrode, Electrostatic Potential (ESP) surfaces were analyzed (Supplementary Fig. 10. The ESP map of TFPP reveals distinct nucleophilic (red) hot-spots on the central phosphorus atom and specific phenyl edges, dictating preferential and targeted oxidation. Crucially, the para-fluorine substituents exhibit neutral-to-electron-poor character, ensuring they survive the initial oxidative attack to subsequently build a structurally robust, inorganic-rich CEI. Conversely, non-fluorinated benchmarks (TPP, TPPa) show diffuse electron density across their organic backbones, which typically results in the formation of mechanically fragile, purely organic interphases incapable of suppressing transition metal crossover. On the other extreme, highly fluorinated analogs (TPFPP, TTFP) present heavily electron-deficient surfaces, structurally confirming their excessive inertness and inability to sacrificially oxidize prior to the degradation of

the polymer backbone.

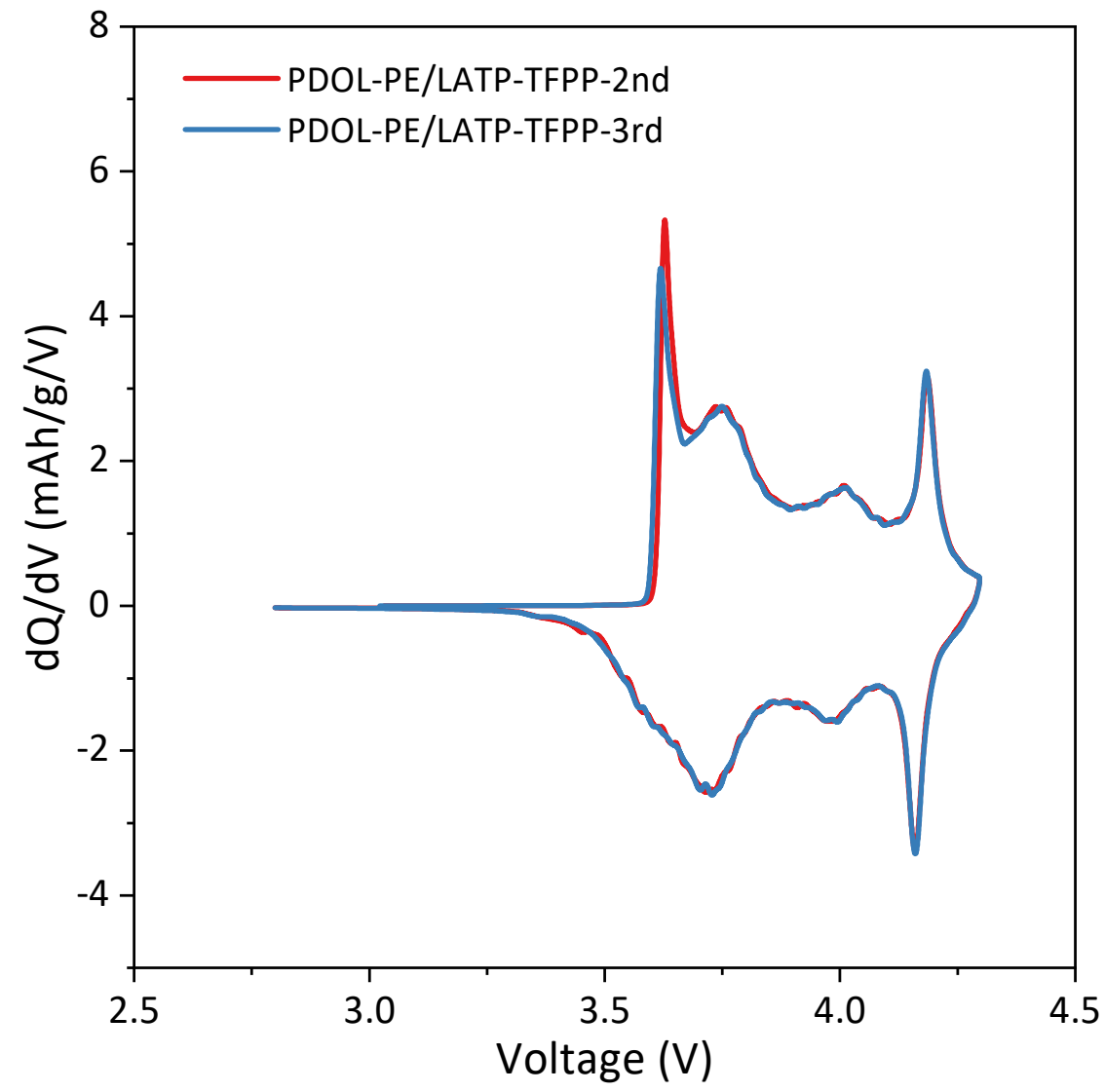

Supplementary Fig. 11 | Electrochemical redox signatures and phase transition behavior of the NCM811 positive electrode. The dQ/dV curves of Li|PDOL-PE/LATP-TFPP|NCM811 cell in the 2nd and 3rd cycles.

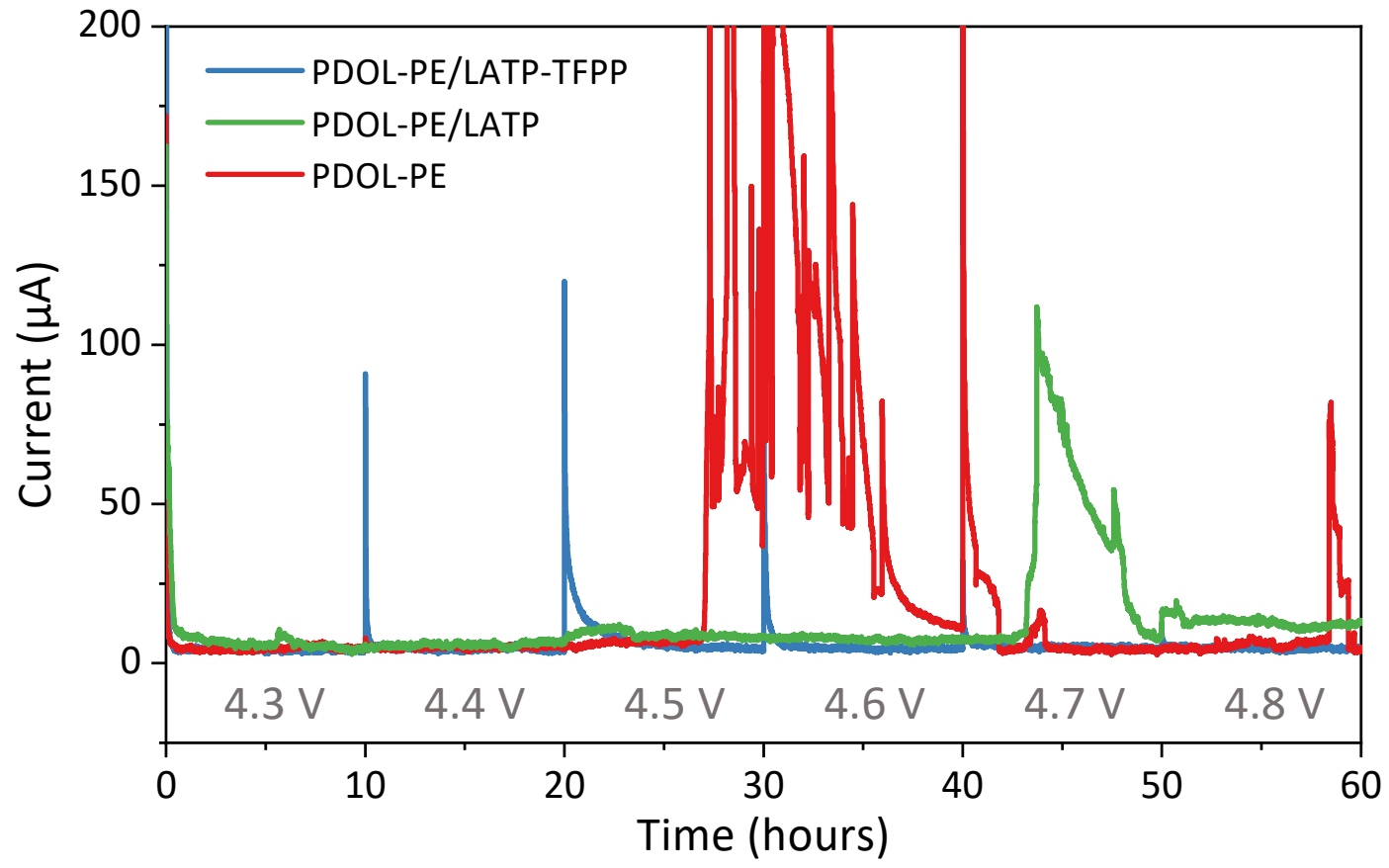

Supplementary Fig. 12 | High-voltage stability of the SPEs. Electrochemical floating tests of Li||NCM811 cells assembled with PDOL-PE, PDOL-PE/LATP and PDOL-PE/LATP/TFPP SPEs.

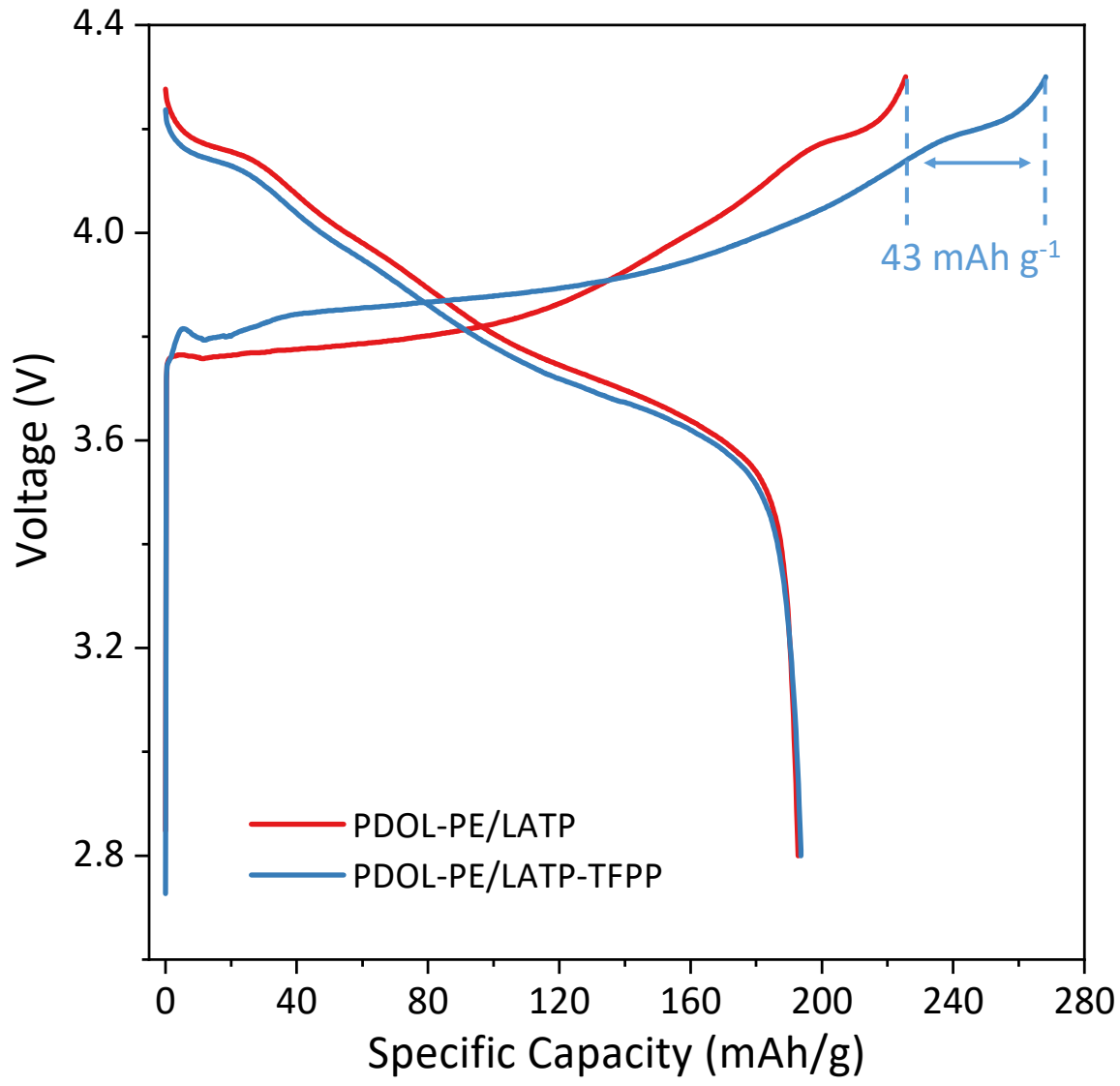

Supplementary Fig. 13 | Electrochemical activation and initial capacity behavior. Initial charge–discharge profiles of the Li|PDOL-PE/LATP|NCM811 and Li|PDOL-PE/LATP-TFPP|NCM811 cells at the formation cycle.

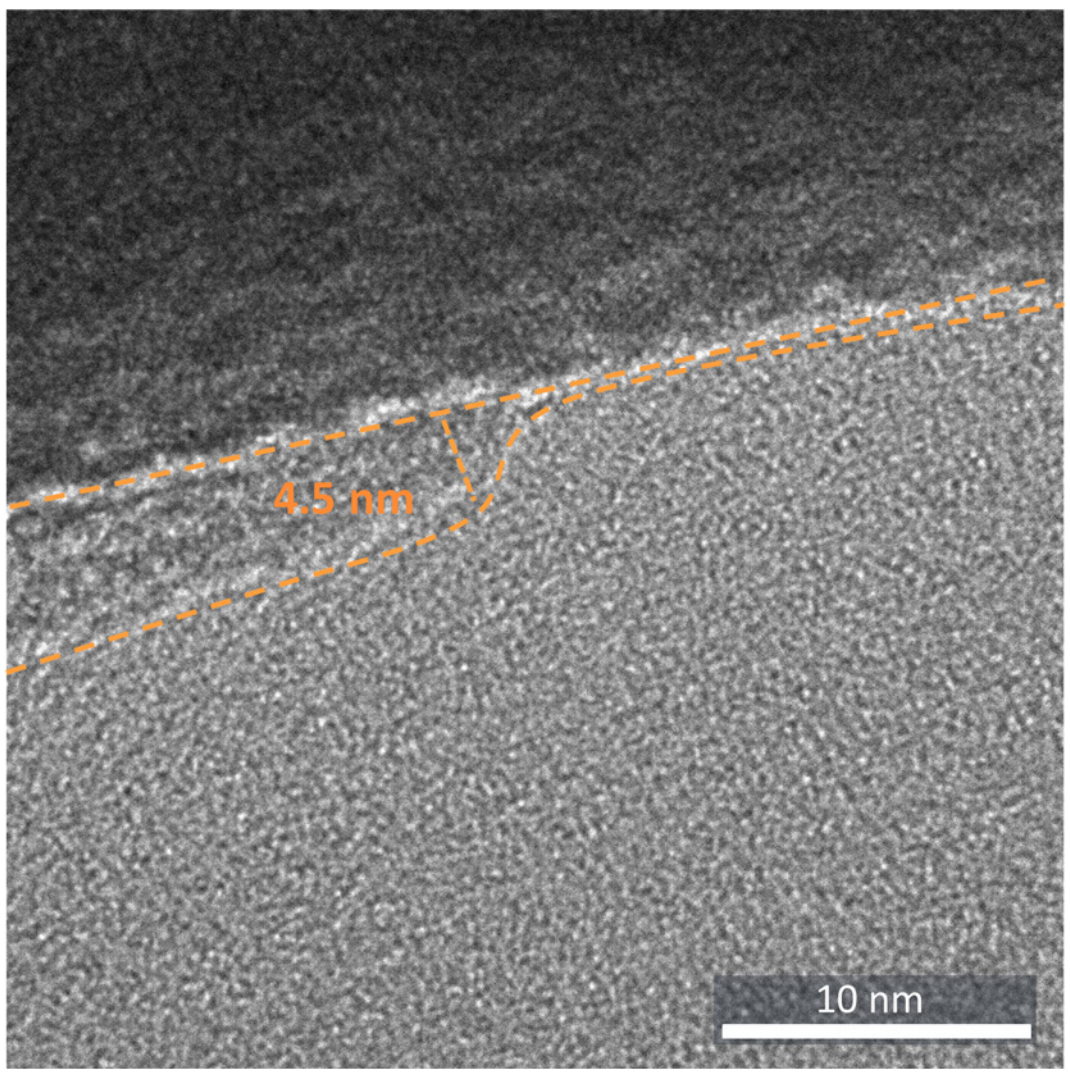

Supplementary Fig. 14 | Microstructural evolution and surface stability of the NCM811 positive electrode. TEM image of the NCM811 positive electrode retrieved from the cycled Li|PDOL-PE/LATP|NCM811 cell.

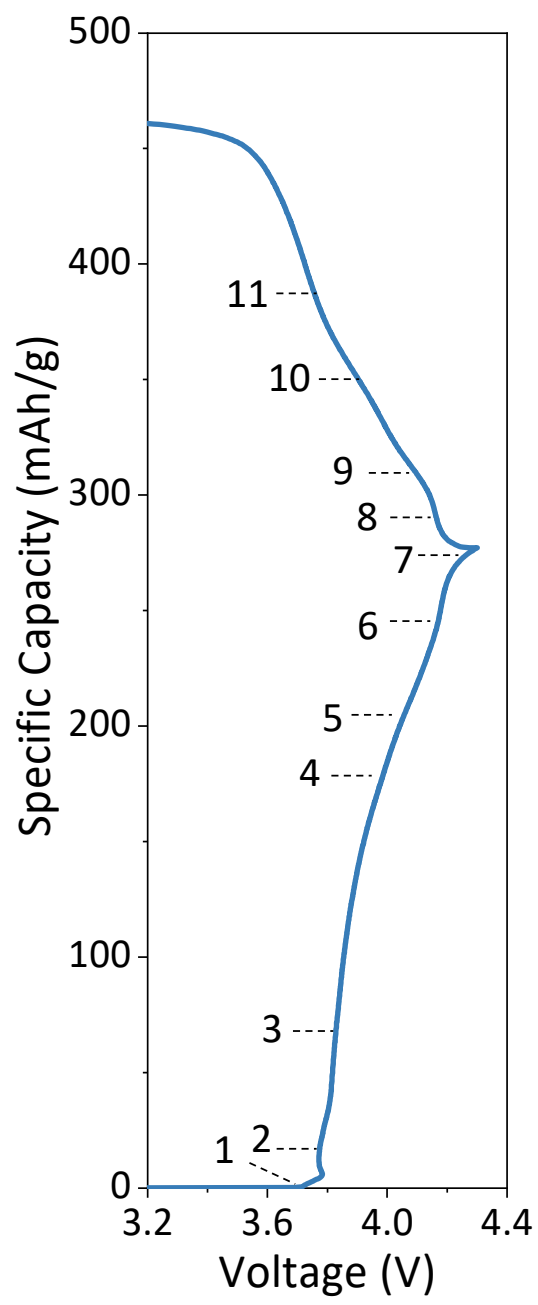

Supplementary Fig. 15 | Initial electrochemical activation and corresponding voltage response. The corresponding voltage profiles were recorded during the initial charge and discharge cycles for the Li|PDOL-PE/LATP-TFPP|NCM811 cell.

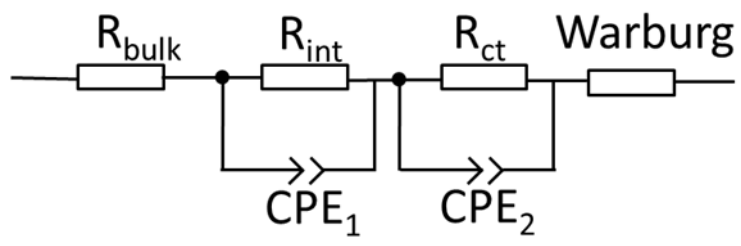

Supplementary Fig. 16 | The equivalent circuit model used for fitting the experimental EIS data.

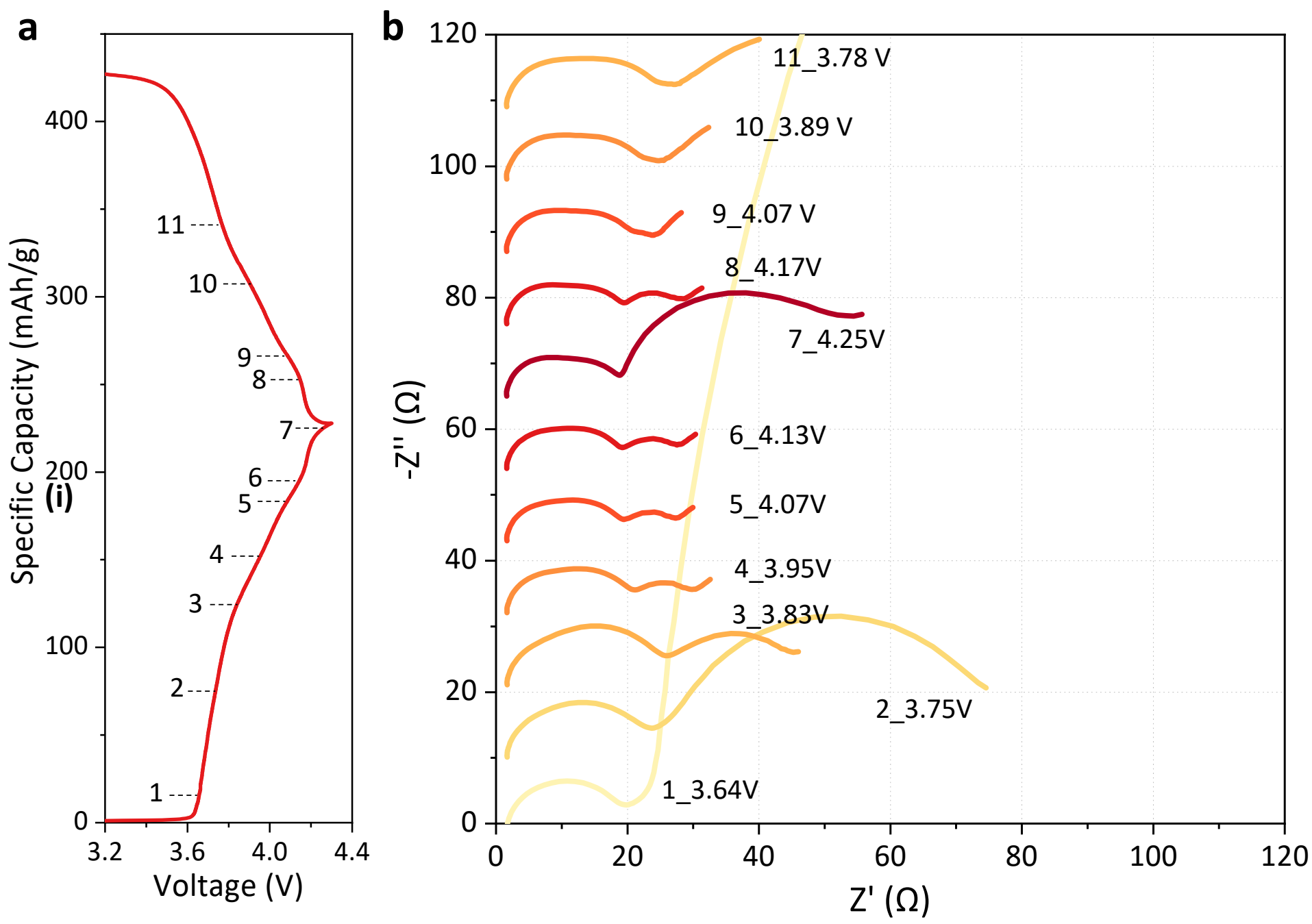

Supplementary Fig. 17 | Dynamic evolution of the interfacial impedance during the electrochemical formation process. a Voltage profiles and the (**b**) corresponding GEIS results of the Li|PDOL-PE/LATP|NCM811 cell, collected during the initial formation cycle.

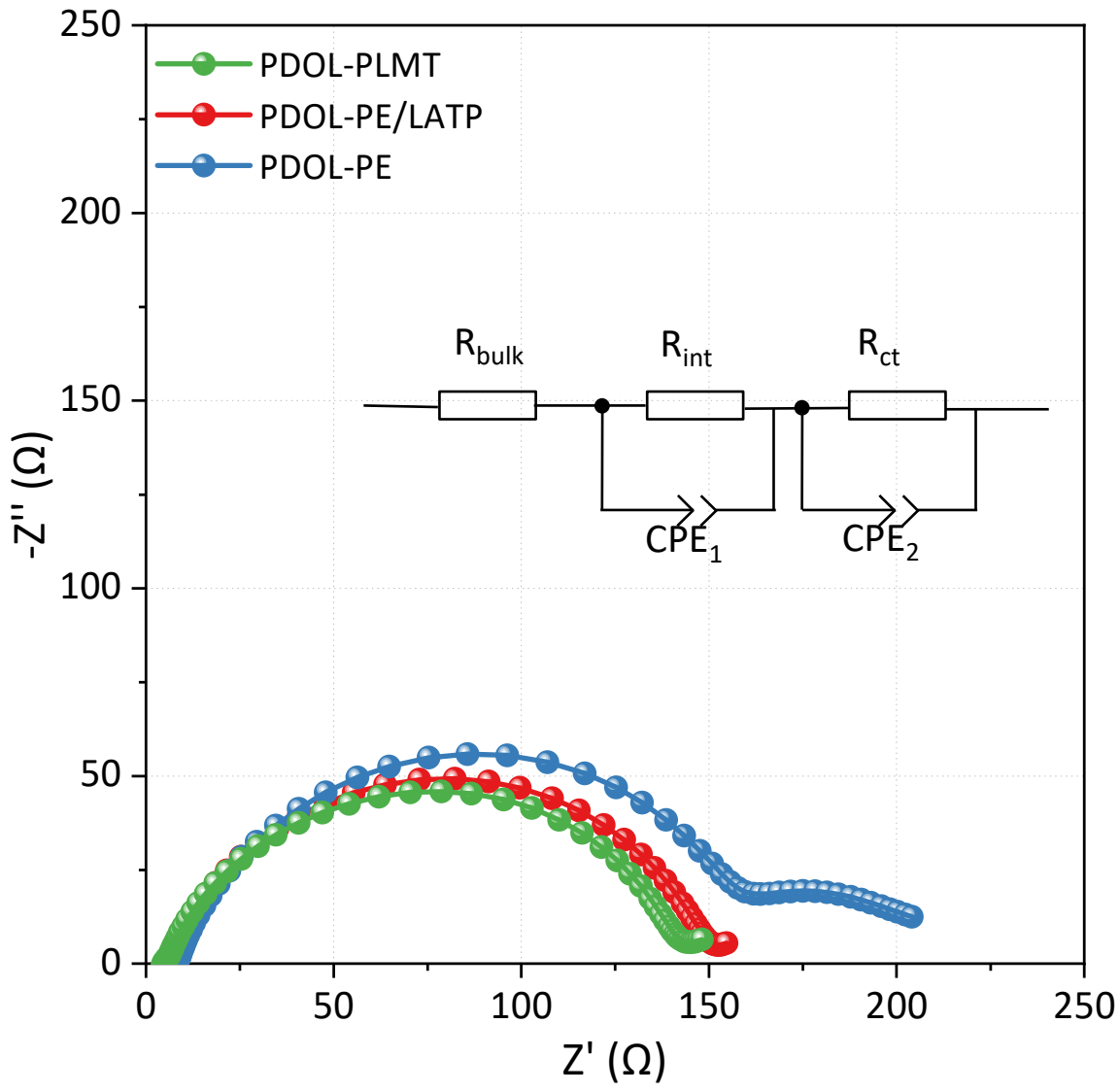

Supplementary Fig. 18 | Interfacial impedance and SEI stability of the lithium metal negative electrodes. EIS plots of the Li|PDOL-PLMT|Li, Li|PDOL-PE/LATP|Li, and Li|PDOL-PE|Li symmetric cells after formation cycling.

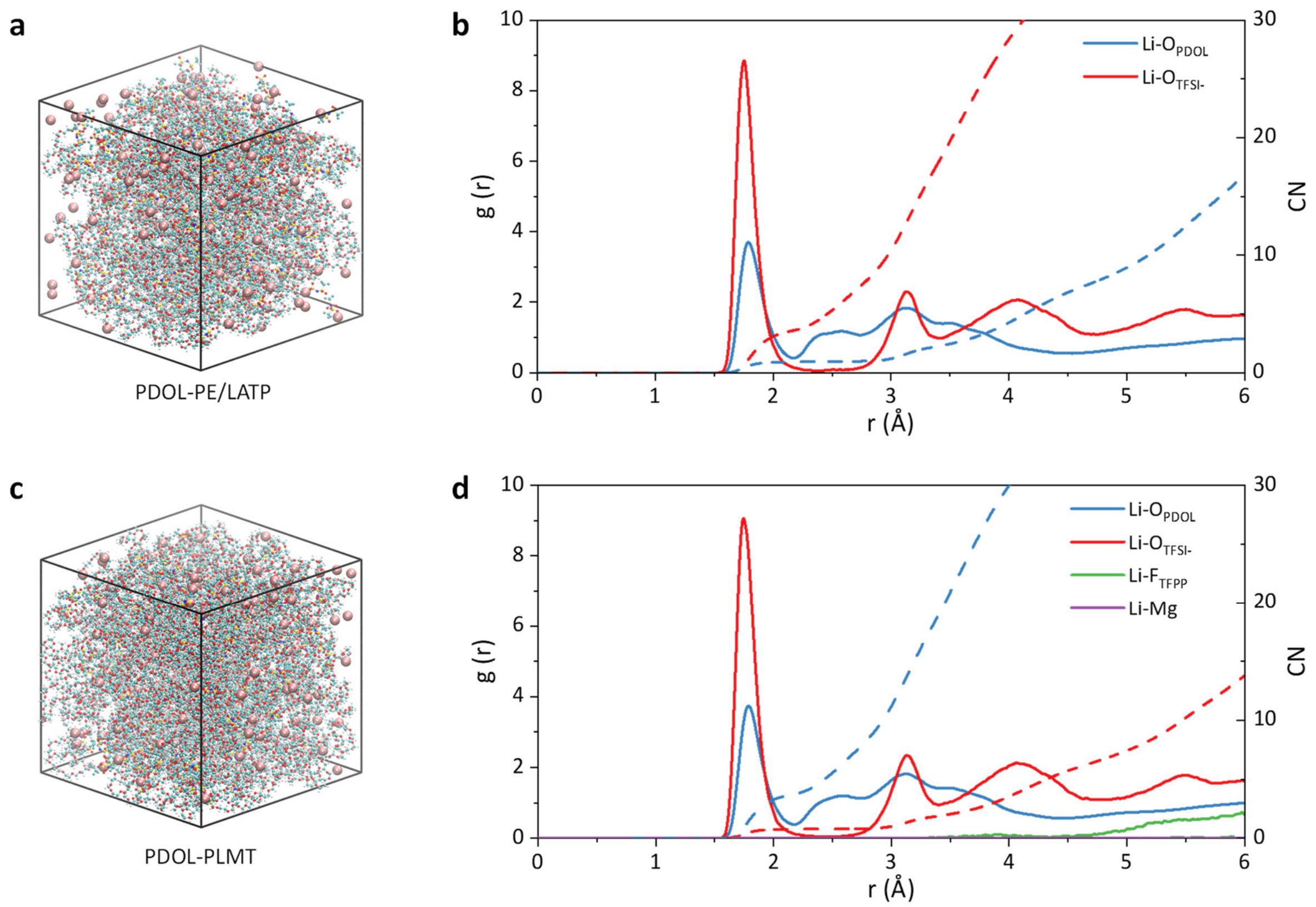

Supplementary Fig. 19 | Molecular dynamics (MD) simulations of the Li^+ coordination environments. a MD snapshots (**b**) RDF and CN of PDOL-PE/LATP electrolyte. **c** MD snapshots (**d**) RDF and CN of PDOL-PLMT electrolyte.

MD simulations were performed to verify that the additives do not negatively alter the bulk Li^+ conduction mechanism (Supplementary Fig. 19). Structural analysis reveals effectively zero coordination between Li^+ and either TFPP or Mg^{2+}. This lack of coordination is thermodynamically expected: the Mg^{2+} cation naturally repels Li^+ due to strong electrostatic forces, while the bulky, fluorinated TFPP molecule exhibits much weaker nucleophilicity toward Li^+ compared to the abundant ether oxygens in the PDOL backbone. Most importantly, the primary Li^+-PDOL and Li^+-$TFSI^-$ transport pathways remain nearly identical to those in the baseline system. This confirms that TFPP and $Mg(TFSI)_2$ do not induce Li^+ trapping within the bulk polymer matrix. Their protective

roles are strictly limited to forming the CEI and Li-Mg alloy at the respective interfaces, thereby ensuring that the unhindered Li^+ transport required for excellent rate capability is fully maintained.

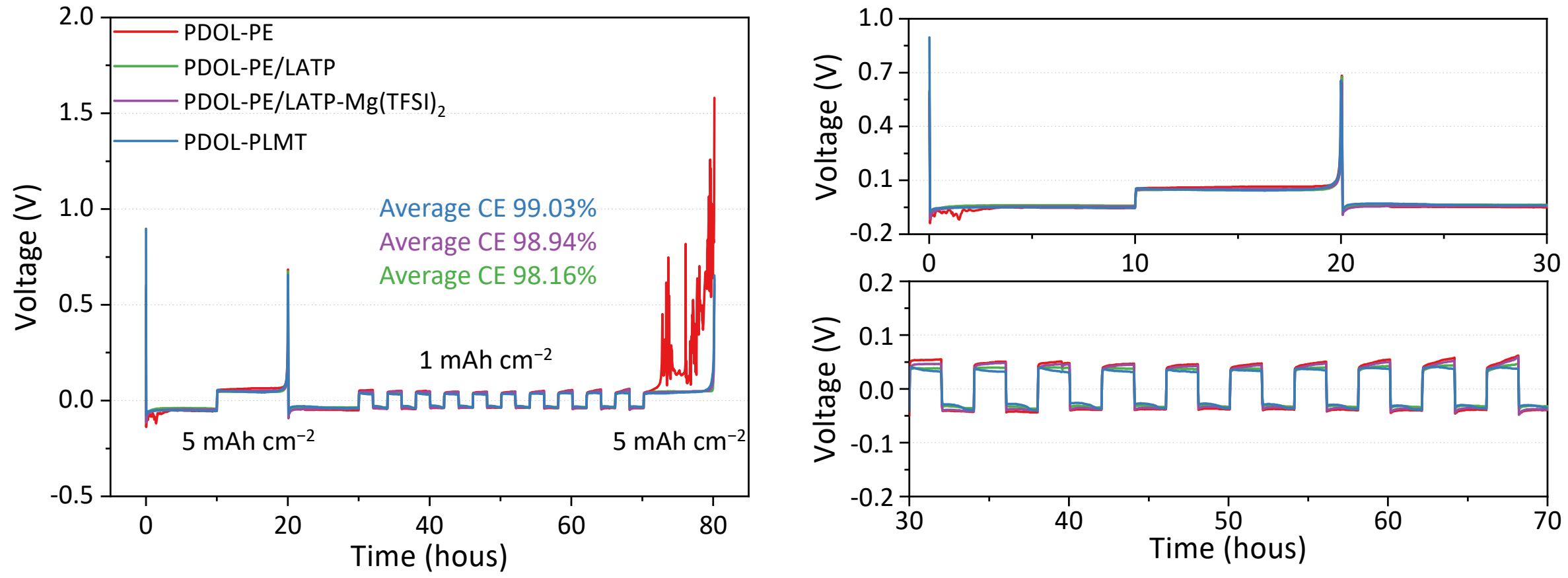

Supplementary Fig. 20 | Electrochemical reversibility of lithium plating and stripping. Comparative Li plating/stripping stability of Li|Cu cells with the four indicated SPEs (PDOL-PE, PDOL-PE/LATP, PDOL-PE/LATP-$Mg(TFSI)_2$, and PDOL-PLMT).

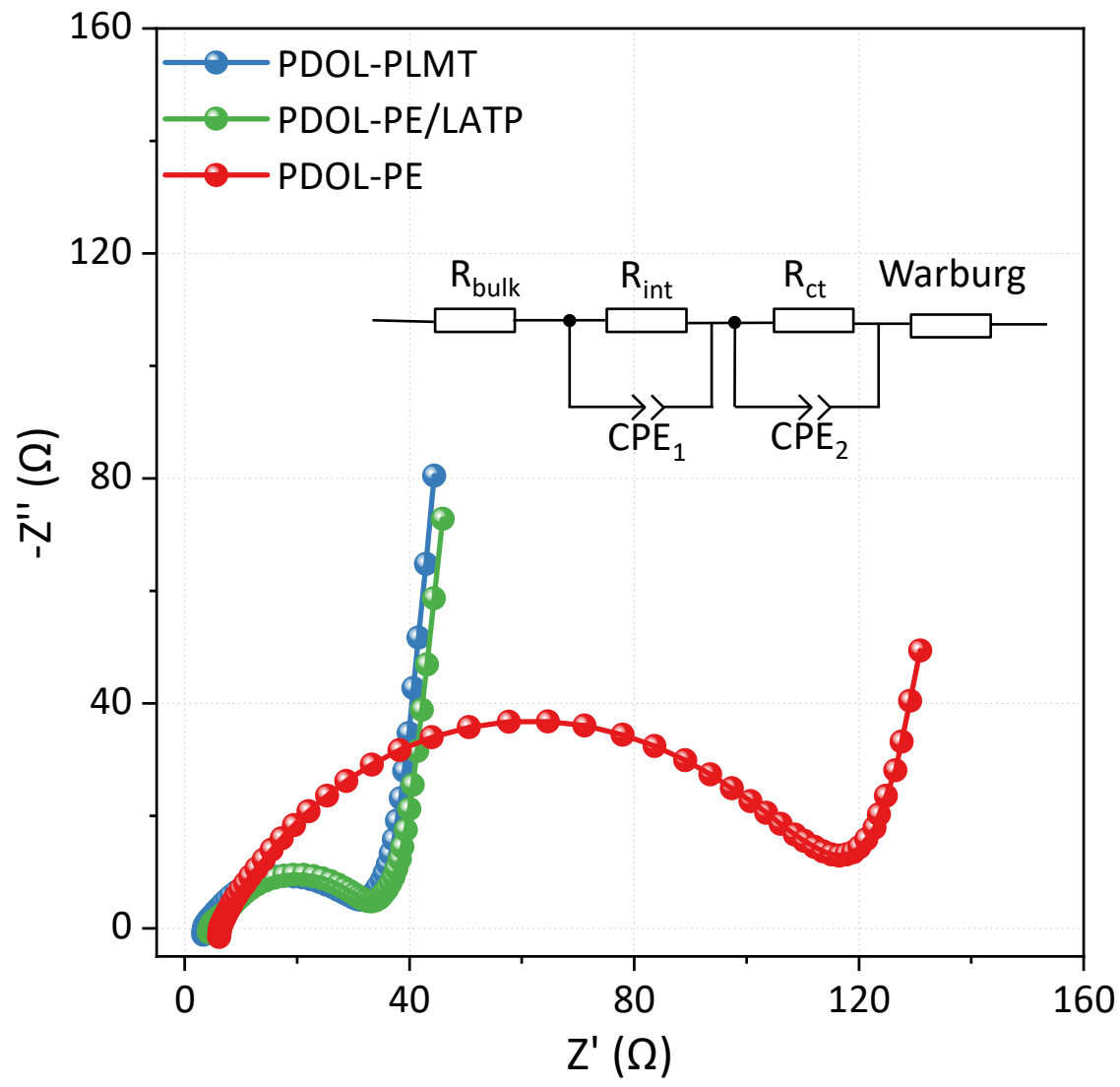

Supplementary Fig. 21 | Electrochemical kinetics and interfacial impedance of the assembled cells. EIS curves of Li|PDOL-PE|NCM811, Li|PDOL-PE/LATP|NCM811 and Li|PDOL-PLMT|NCM811 cells.

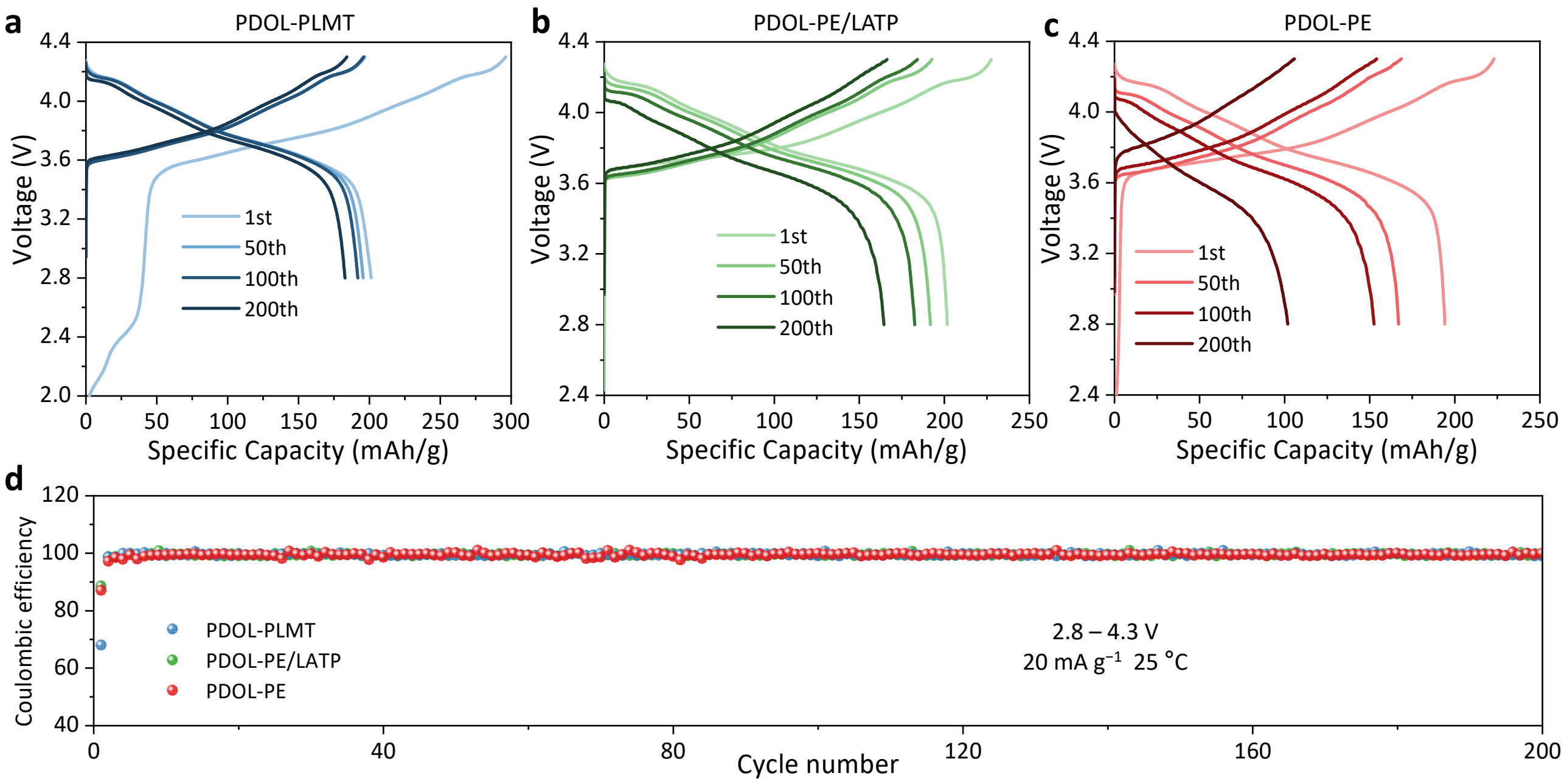

Supplementary Fig. 22 | Comparative electrochemical performance and voltage polarization of the assembled cells. Charge–discharge profiles of (**a**) Li|PDOL-PLMT|NCM811, (**b**) Li|PDOL-PE/LATP|NCM811 and (**c**) Li|PDOL-PE|NCM811 cells at 20 mA g^{-1} (2.8–4.3 V, 25 °C). (**d**) Comparative Coulombic efficiencies of Li||NCM811 cells integrated with PDOL-PLMT, PDOL-PE/LATP, and PDOL-PE electrolytes over continuous cycling.

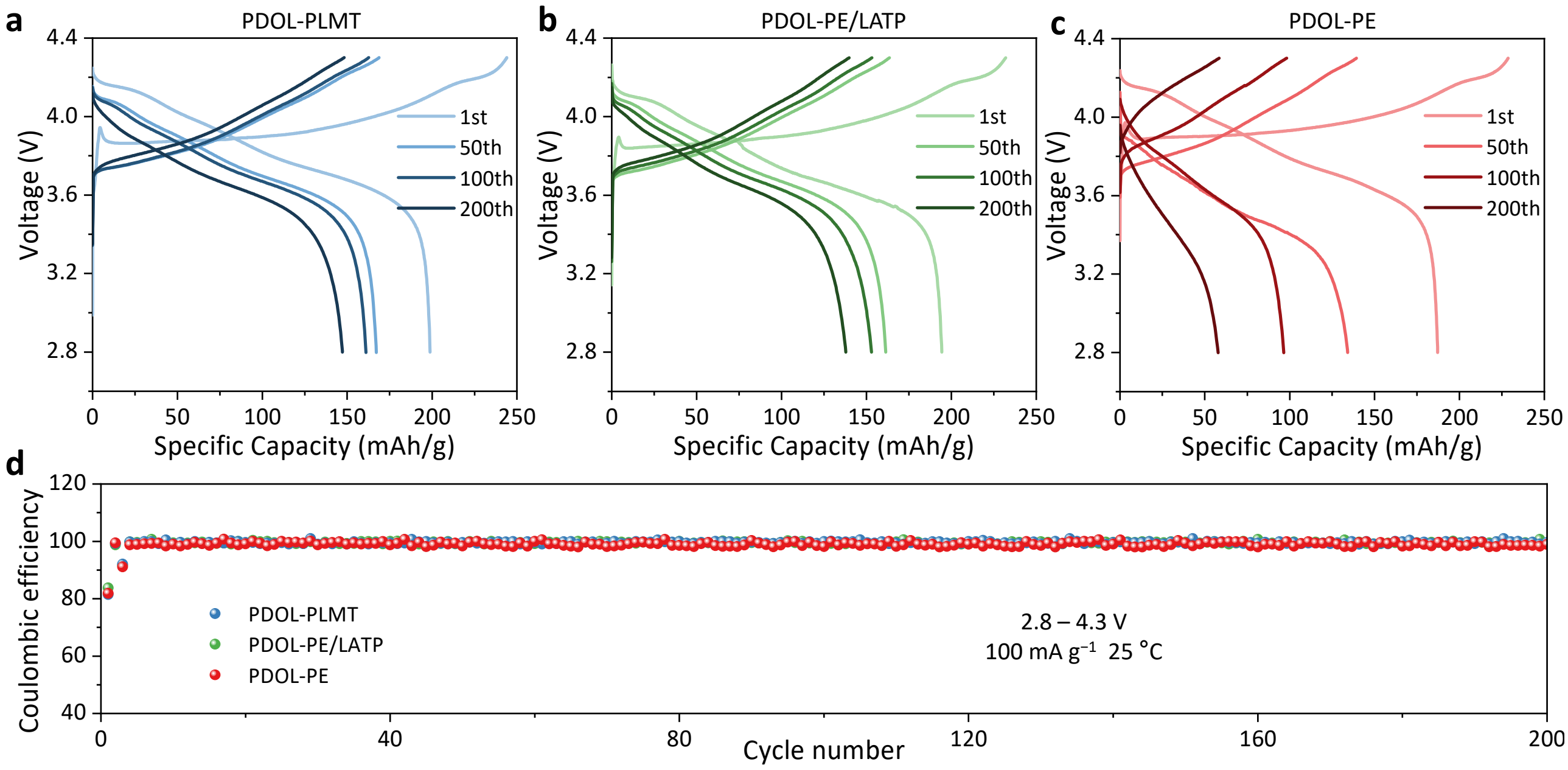

Supplementary Fig. 23 | Electrochemical kinetics and voltage polarization at an elevated rate. Charge–discharge profiles of (**a**) Li|PDOL-PLMT|NCM811, (**b**) Li|PDOL-PE/LATP|NCM811 and (**c**) Li|PDOL-PE|NCM811 cells at 100 mA g^{-1} (2.8–4.3 V, 25 °C). (**d**) Comparative Coulombic efficiencies of Li||NCM811 cells integrated with PDOL-PLMT, PDOL-PE/LATP, and PDOL-PE electrolytes over continuous cycling.

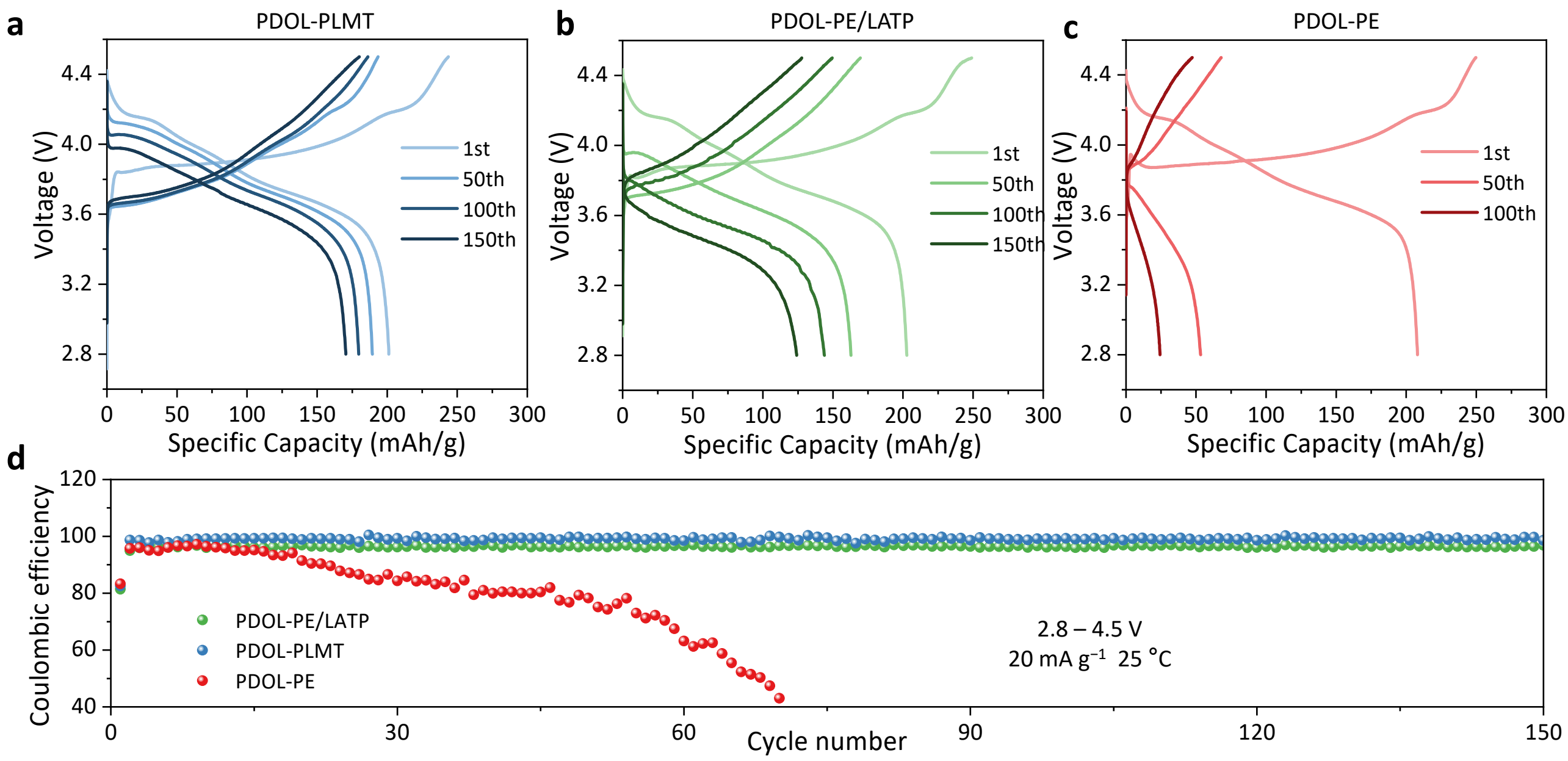

Supplementary Fig. 24 | Extreme high-voltage tolerance and electrochemical reversibility. Charge–discharge profiles of (**a**) Li|PDOL-PLMT|NCM811, (**b**) Li|PDOL-PE/LATP|NCM811 and (**c**) Li|PDOL-PE|NCM811 cells at 20 mA g^{-1} (2.8–4.5 V, 25 °C). (**d**) Comparative Coulombic efficiencies of Li||NCM811 cells integrated with PDOL-PLMT, PDOL-PE/LATP, and PDOL-PE electrolytes over continuous cycling.

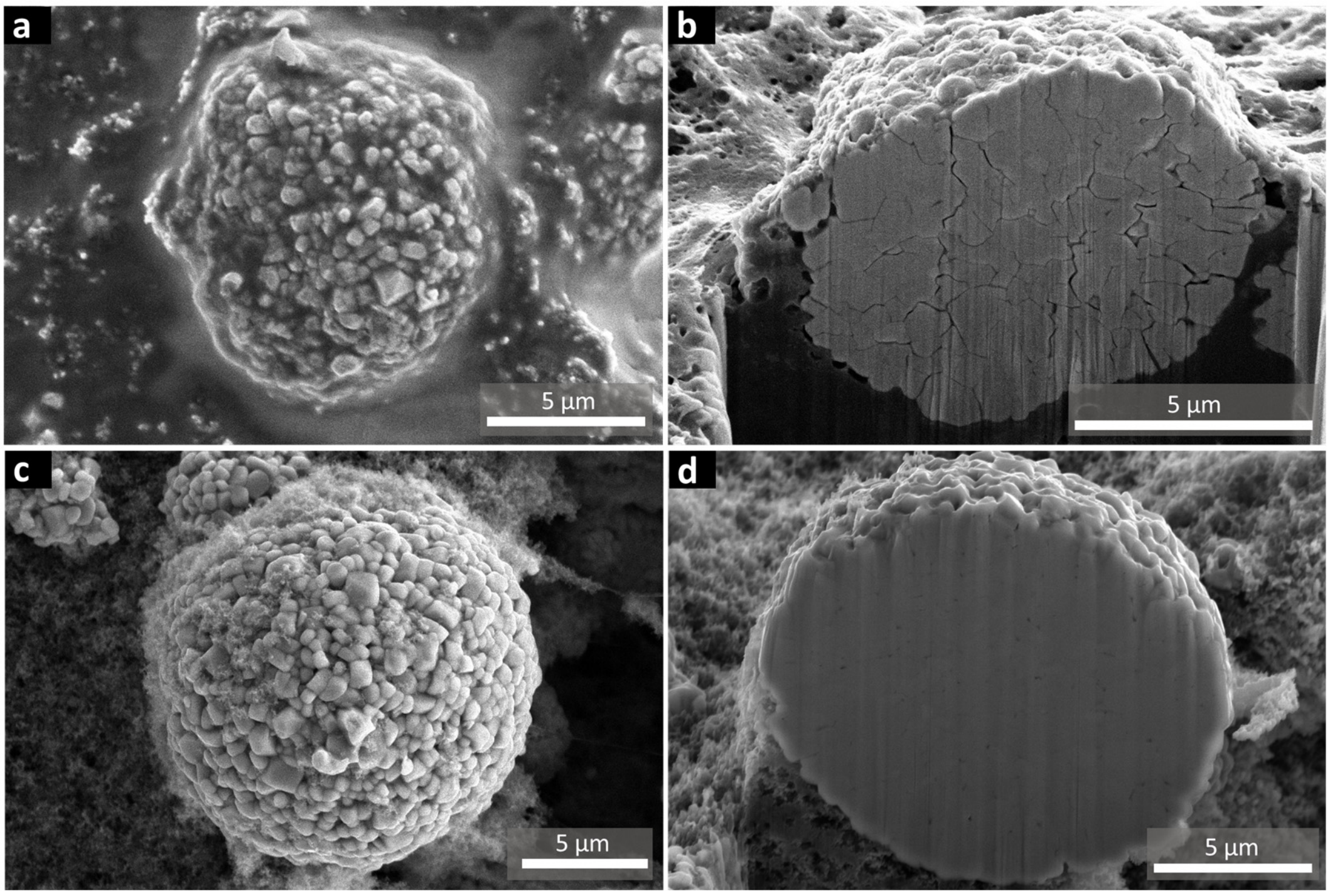

Supplementary Fig. 25 | Morphological stability and suppression of internal microcracking in the NCM811 positive electrodes. Surface and FIB-prepared cross-sectional SEM images of the NCM811 positive electrodes after 150 cycles (2.8–4.5 V, 20 mA g^{-1}, 25 °C) retrieved from the (**a, b**) Li|PDOL-PE/LATP|NCM811 and (**c, d**) Li|PDOL-PLMT|NCM811 cells.

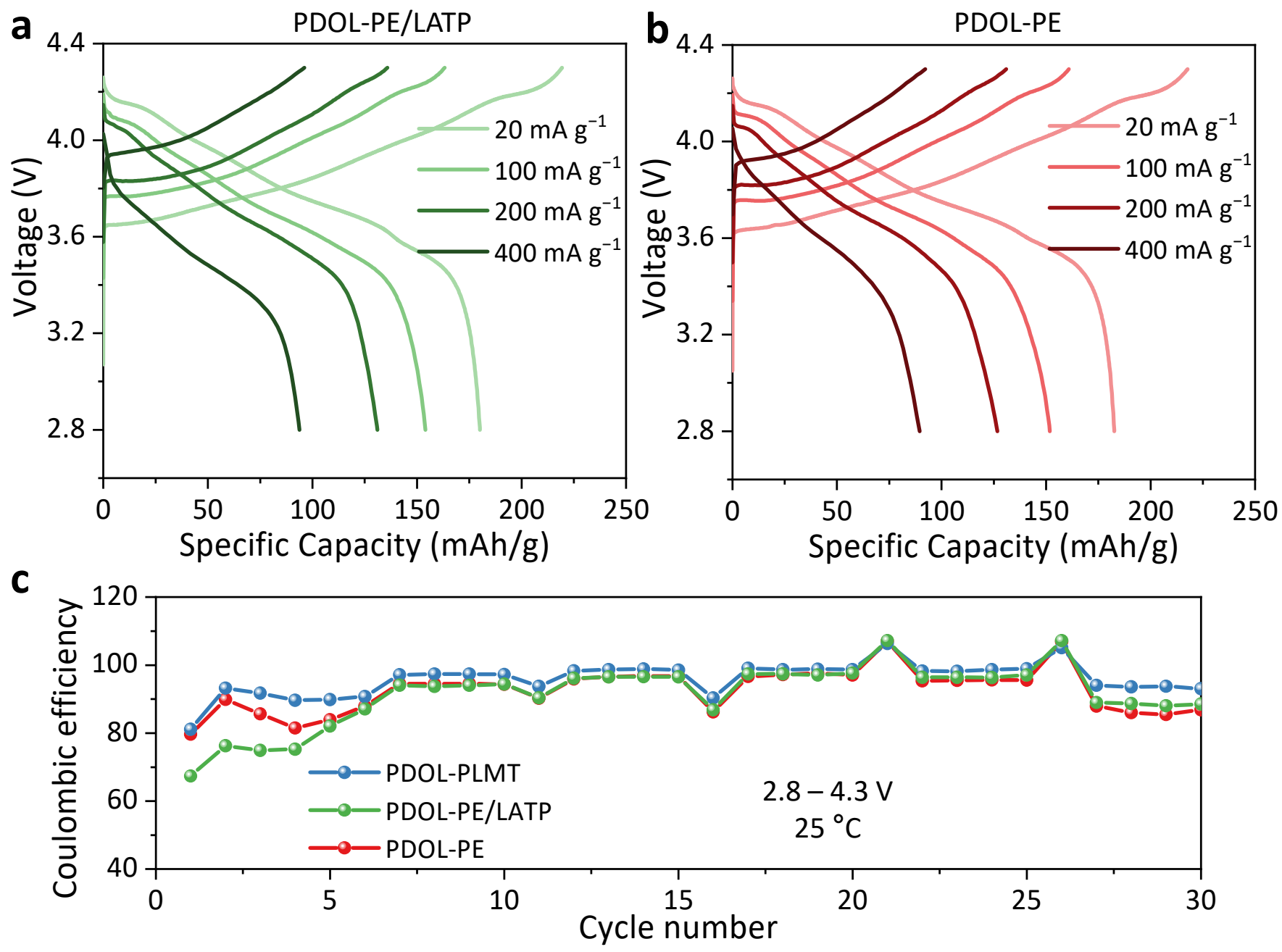

Supplementary Fig. 26 | Rate-dependent electrochemical behaviors and kinetic response. Charge–discharge profiles of the (**a**) Li|PDOL-PE/LATP|NCM811, and (**b**) Li|PDOL-PE|NCM811 cells evaluated at various current densities from 20 to 400 mA g^{-1} (2.8–4.3 V, 25 °C). (**c**) Rate-dependent coulombic efficiencies of Li||NCM811 cells utilizing the three respective electrolytes, showing the interfacial reversibility under varying current densities.

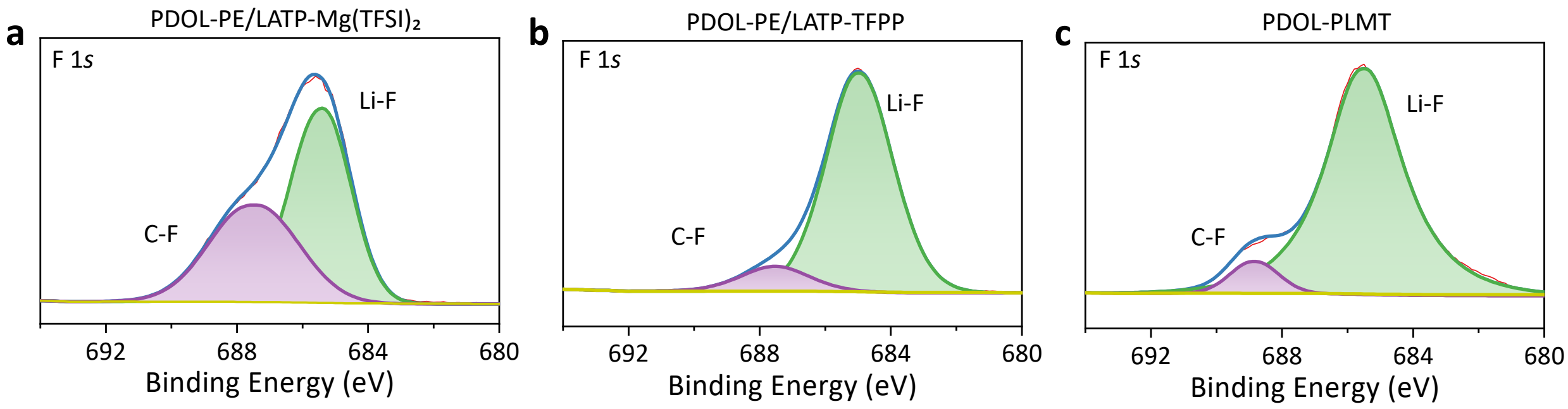

Supplementary Fig. 27 | Surface chemistry and SEI composition analysis on the Li metal negative electrodes. High-resolution F 1s XPS spectra of the Li metal negative electrode surfaces harvested from Li||NCM811 coin cells after 100 cycles (2.8–4.5 V, 20 mA g^{-1}, 25 °C), employing (**a**) PDOL-PE/LATP-$Mg(TFSI)_2$, (**b**) PDOL-PE/LATP-TFPP, and (**c**) PDOL-PLMT.

Comparative F 1s XPS analysis of the cycled Li negative electrodes (Supplementary Fig. 27) reveals uniformly high LiF intensities across the single- and dual-additive systems. This confirms that the LiF-rich SEI is primarily dictated by the decomposition of the $TFSI^-$ anions, independent of TFPP. Consequently, this explicitly decouples the distinct additive functions, validating that the superior high-voltage stability conferred by TFPP strictly originates from its robust construction of the CEI.

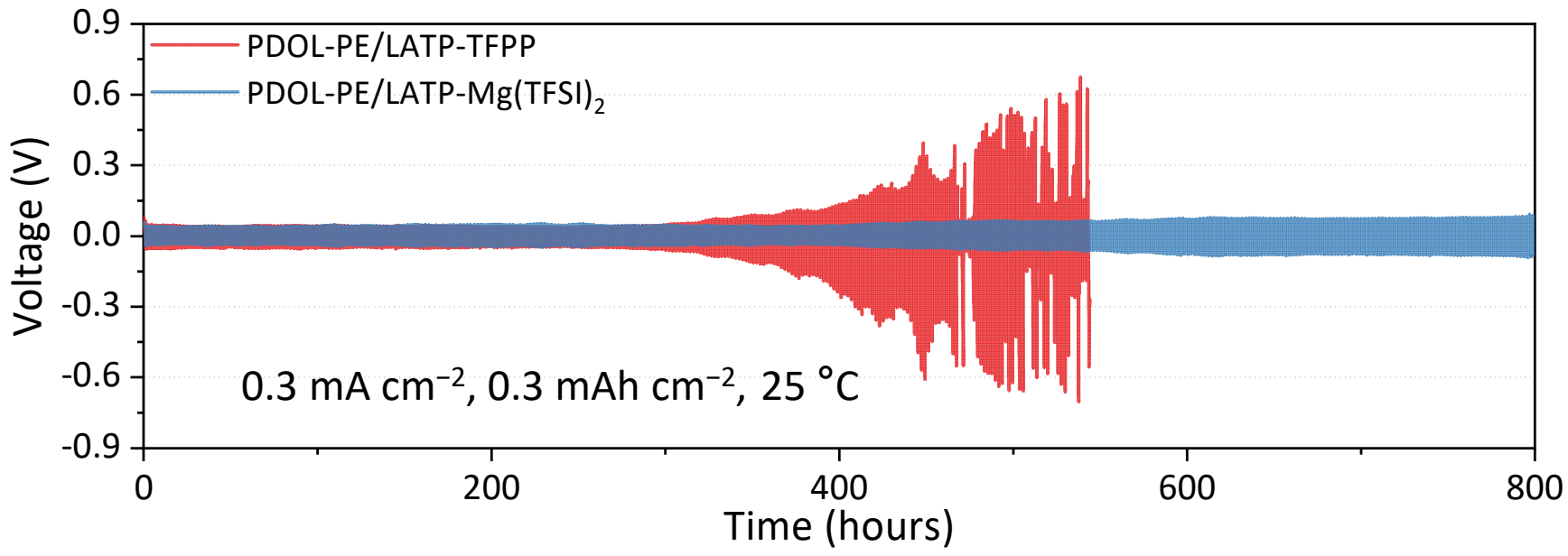

Supplementary Fig. 28 | Long-term interfacial stability and lithium cycling endurance. Galvanostatic Li plating/stripping profiles of the Li|Li symmetric cells employing the PDOL-PE/LATP-TFPP and PDOL-PE/LATP-$Mg(TFSI)_2$ electrolytes.

To explicitly decouple the interfacial roles of the additives and evaluate lithium plating/stripping stability, Li|Li symmetric cells were systematically evaluated (Supplementary Fig. 28). The control electrolyte formulated exclusively with the PDOL-PE/LATP-TFPP succumbed to soft short-circuits within 500 hours, indicative of uncontrolled dendritic penetration. In stark contrast, the incorporation of $Mg(TFSI)_2$ enabled remarkably stable voltage profiles for over 800 hours. This comparative electrochemical evidence unambiguously dictates that the in situ formed Li–Mg alloy interphase is the dominant factor in regulating homogeneous Li deposition and fundamentally preventing dendrite-induced failure.

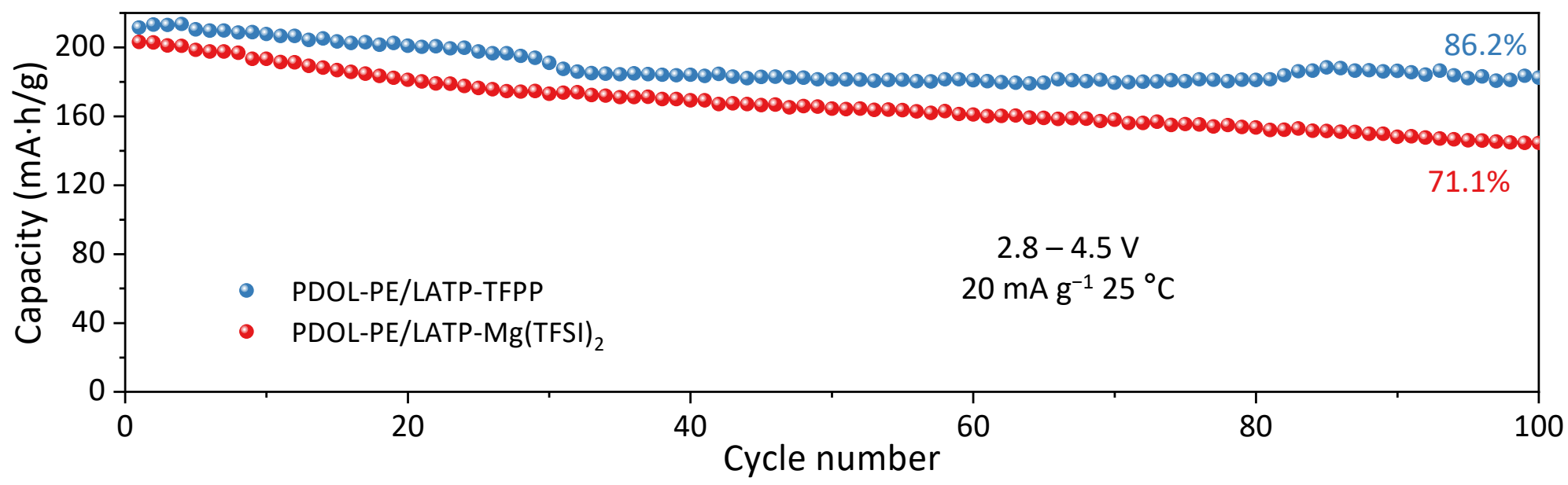

Supplementary Fig. 29 | High-voltage cycling endurance and capacity retention. Long-term cycling performance of Li||NCM811 cells using PDOL-PE/LATP-TFPP and PDOL-PE/LATP-$Mg(TFSI)_2$ electrolytes at 4.5V (25 °C).

Evaluating full-cell performance under an aggressive 4.5 V cut-off explicitly isolates the positive electrode-stabilizing efficacy of TFPP (Supplementary Fig. 29). The $Mg(TFSI)_2$-only control exhibited severe degradation, with capacity retention plummeting to 71.1% after 100 cycles. In sharp contrast, the isolated addition of TFPP effectively mitigated this decay, delivering an 86.2% retention that closely approaches the fully integrated dual-additive system. This comparative evaluation conclusively establishes that the TFPP-derived CEI is essential for protecting the highly reactive NCM811 positive electrode under severe high-voltage stress.

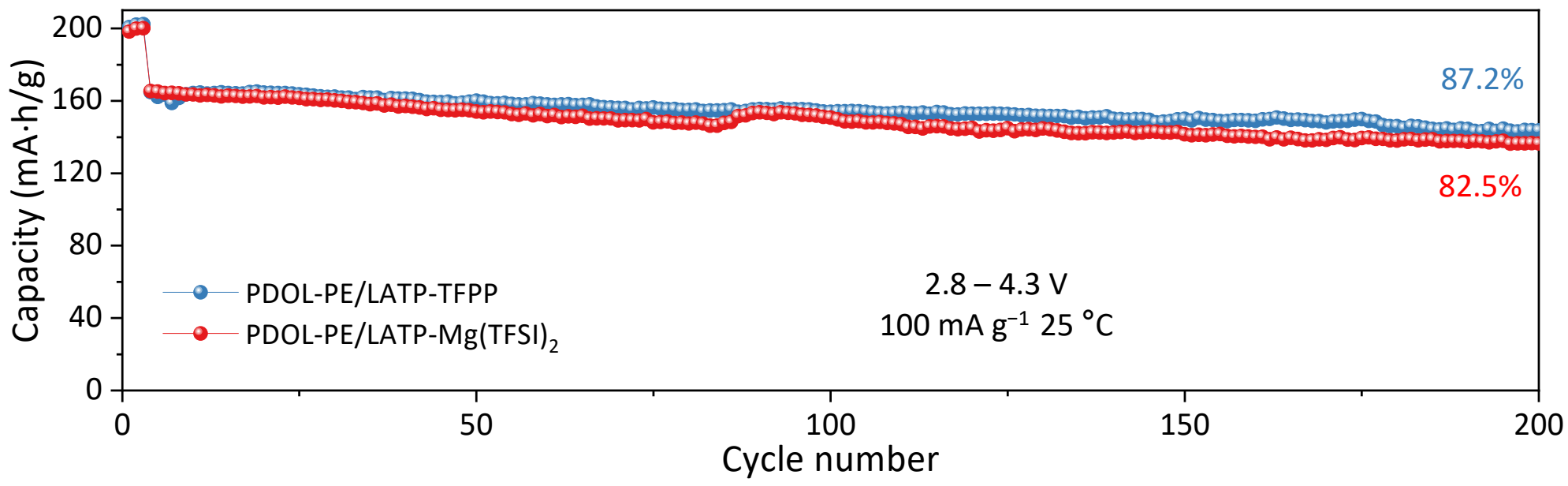

Supplementary Fig. 30 | Comparative evaluation of the long-term cycling endurance. Long-term cycling performance of Li||NCM811 coin-cells using PDOL-PE/LATP-TFPP and PDOL-PE/LATP-$Mg(TFSI)_2$ electrolytes at 100 mA g^{-1} (2.8–4.3 V, 25 °C).

To further delineate the distinct interfacial contributions under continuous electrochemical stress, long-term cycling was evaluated at 100 mA g^{-1} (Supplementary Fig. 30). This comparative analysis highlights the indispensable role of TFPP in securing long-term reversibility. Specifically, the control system relying solely on the $Mg(TFSI)_2$ anodic regulator exhibited cumulative capacity fade, yielding an 82.5% retention after 200 cycles. In distinct contrast, the isolated addition of TFPP effectively suppressed this degradation, achieving a significantly superior retention of 87.2%. This quantitative performance gap unambiguously corroborates that the TFPP-derived CEI provides essential shielding for the positive electrode, sustaining its structural integrity throughout extended operation.

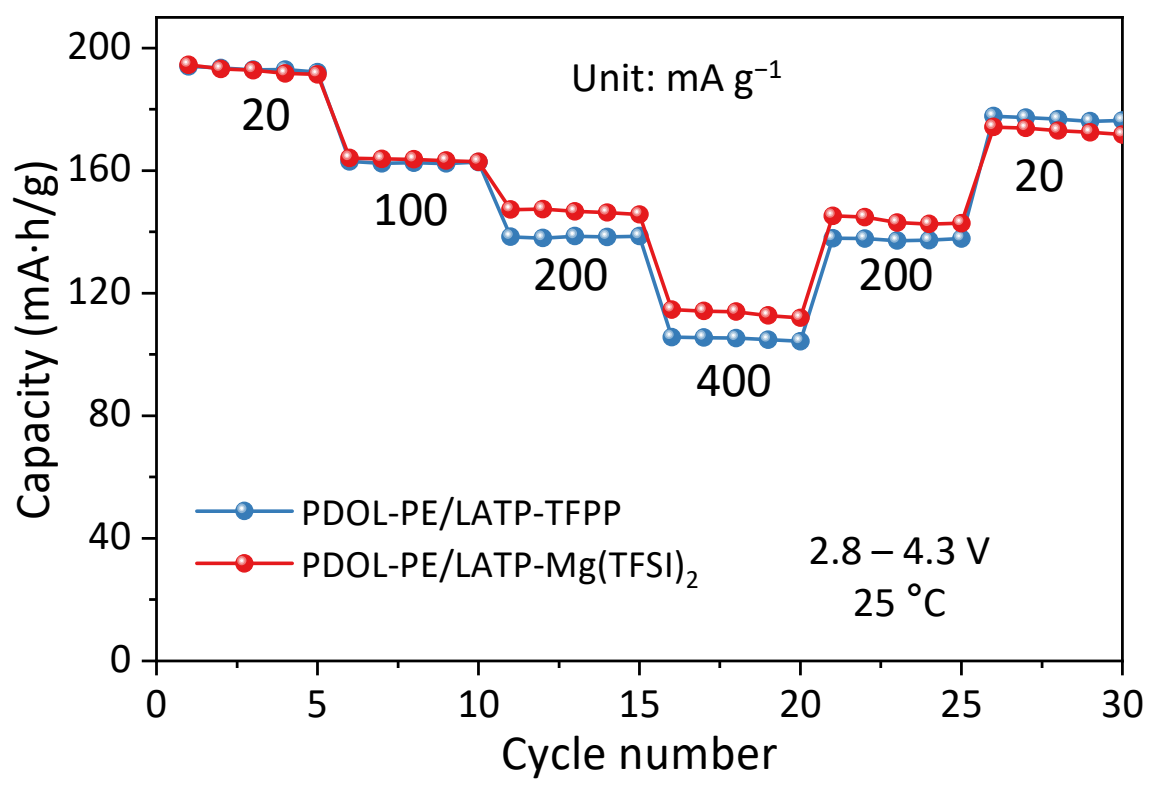

Supplementary Fig. 31 | Rate capability and electrochemical kinetic response at 25 °C. Comparative rate performance of the Li||NCM811 cells employing PDOL-PE/LATP-TFPP and PDOL-PE/LATP-Mg(TFSI)$_2$ electrolytes from 20 to 400 mA g^{-1}.

Complementary rate capability tests (Supplementary Fig. 31) precisely isolate the kinetic advantages imparted by the anodic regulator. As the applied current density incrementally increases, systems formulated with Mg(TFSI)$_2$ demonstrate profoundly superior high-rate kinetics compared to their Mg-free counterparts. This exceptional rate performance stems directly from the in situ construction of the lithiophilic Li–Mg alloy-rich SEI. By establishing a fast-ion conducting interfacial network that drastically minimizes the Li^+ diffusion barrier, the Mg-regulated interphase fundamentally facilitates rapid charge transfer, ensuring robust capacity delivery even under stringent high-rate conditions.

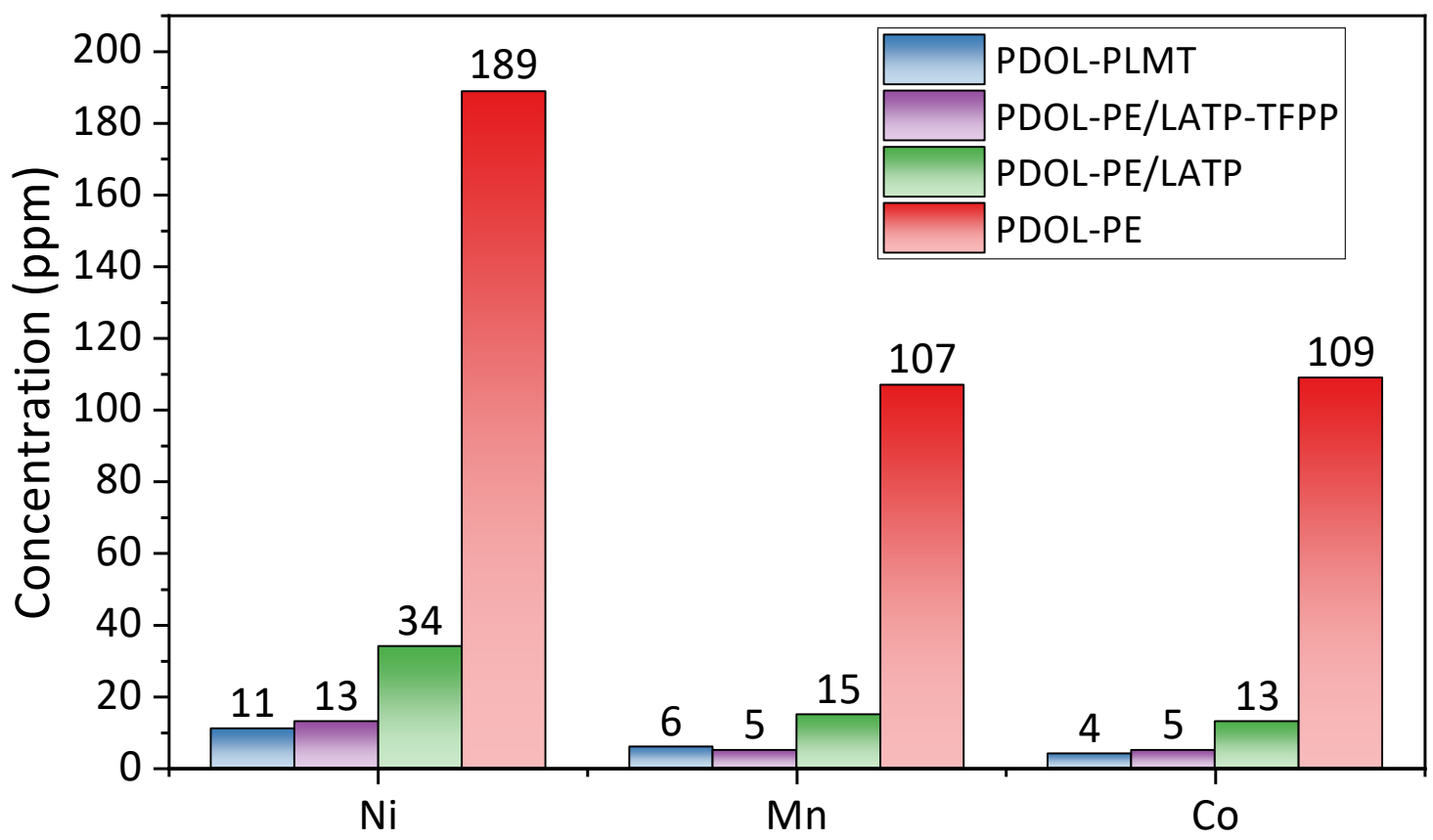

Supplementary Fig. 32 | Quantitative analysis of TM dissolution and migration. Ni, Co, and Mn contents detected on Li metal negative electrodes harvested from cycled Li||NCM811 cells assembled with four different electrolytes.

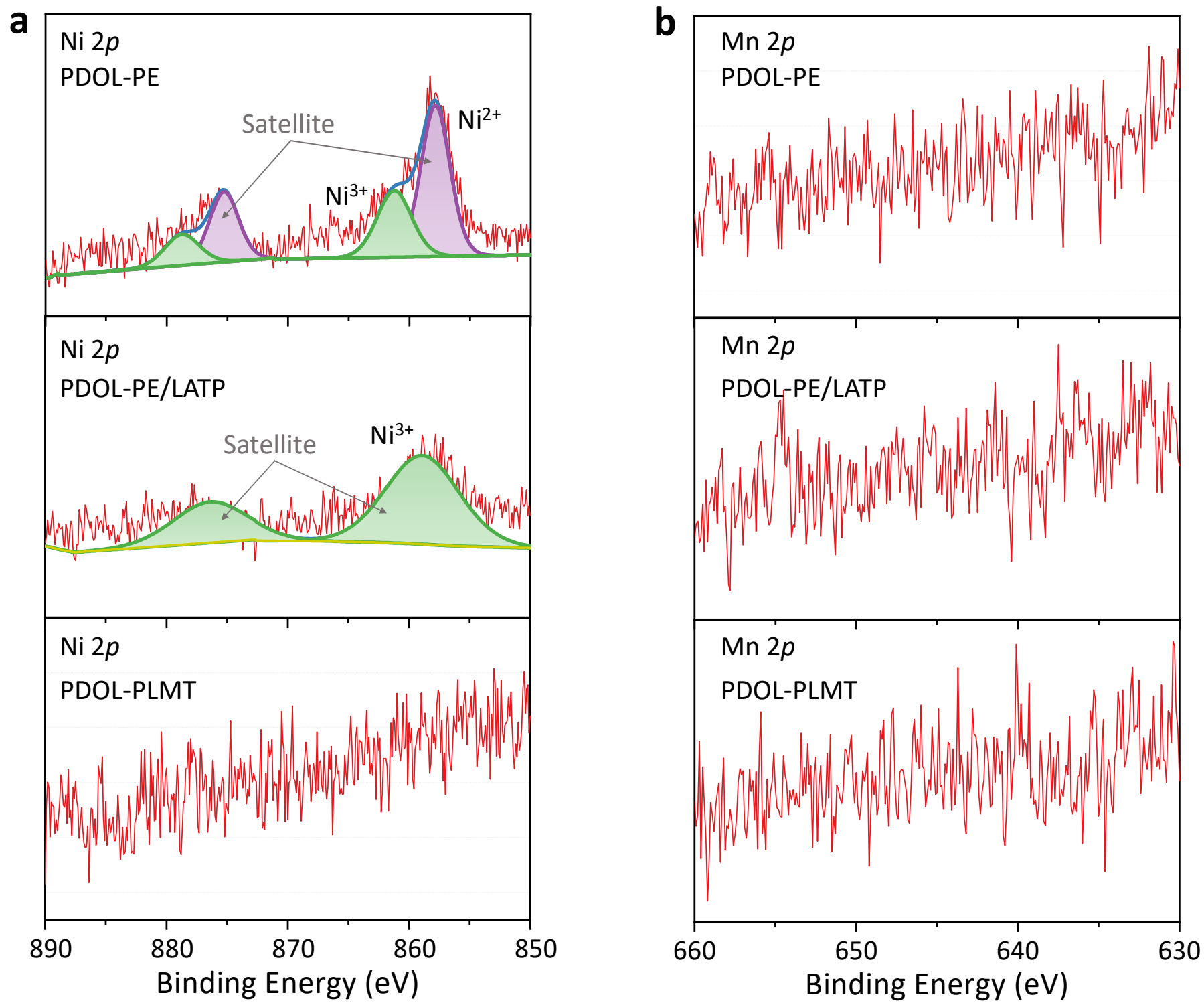

Supplementary Fig. 33 | Surface chemical analysis of the TM crossover. Comparison of the core-level (**a**) Ni 2p and (**b**) Mn 2p spectra detected on Li metal negative electrodes harvested from cycled Li||NCM811 coin cells assembled with PDOL-PE, PDOL-PE/LATP and PDOL-PLMT.

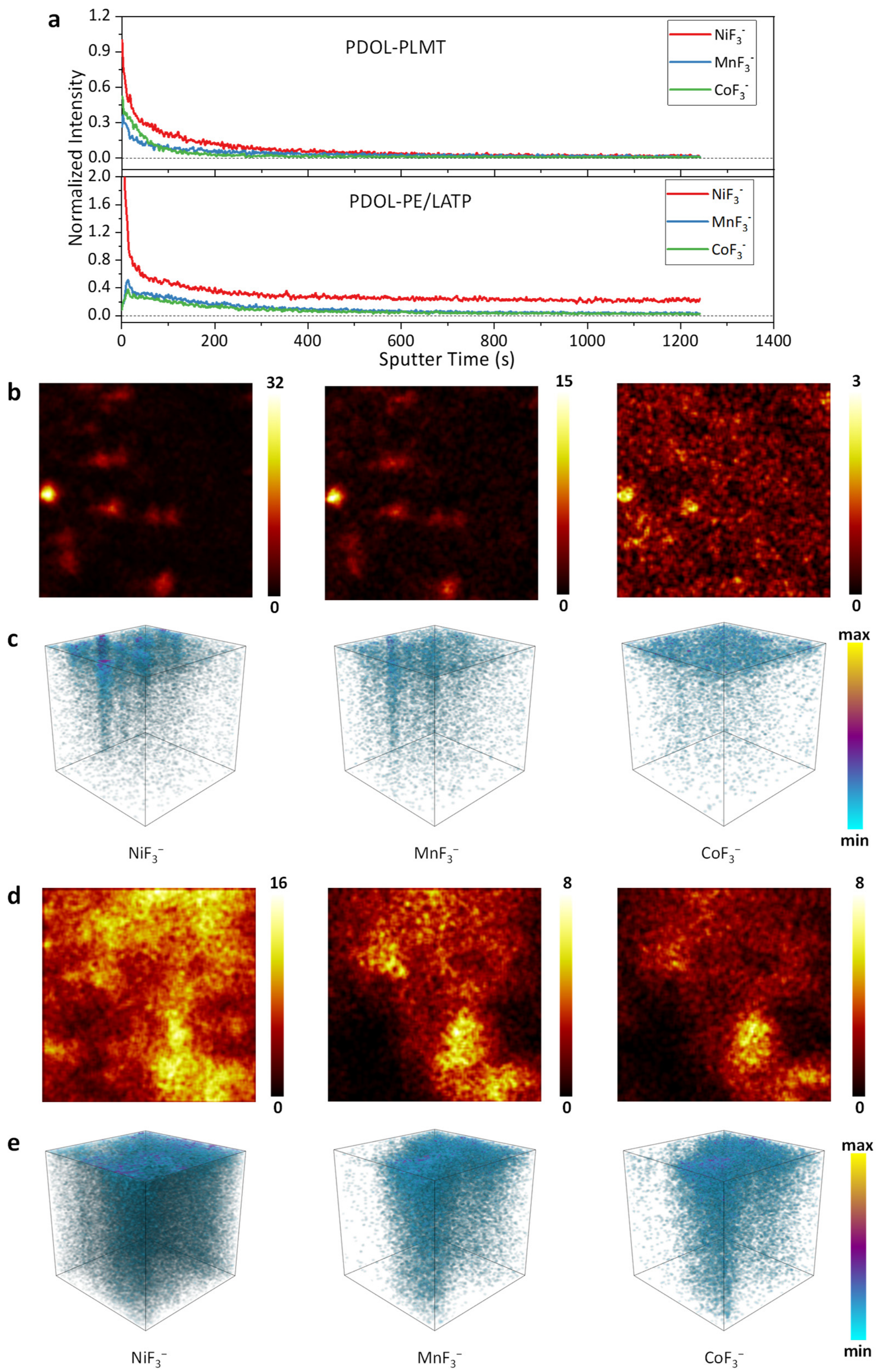

255

Supplementary Fig. 34 | 3D visualization of the TM species on cycled Li negative electrodes. (a) Comparative TOF-SIMS depth profiles of transition metal fluoride fragments (NiF_3^-, MnF_3^-, and

CoF_3^-) on Li metal negative electrodes harvested from Li|PDOL-PLMT|NCM811 and Li|PDOL-PE/LATP|NCM811 pouch cells after 400 cycles. 2D mapping images and 3D reconstructions of the corresponding species on the Li negative electrode surfaces retrieved from the (**b**, **c**) PDOL-PLMT and (**d**, **e**) PDOL-PE/LATP systems.

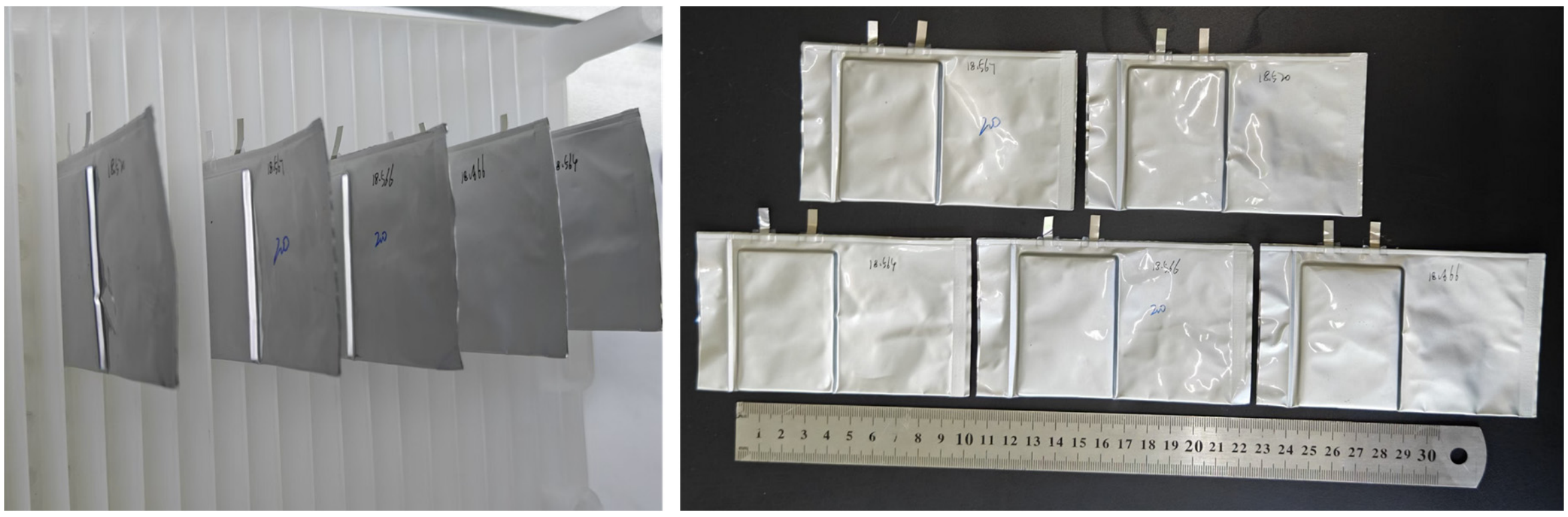

Supplementary Fig. 35 | Batch-assembly of 1.2 Ah Li||NCM811 pouch cells.

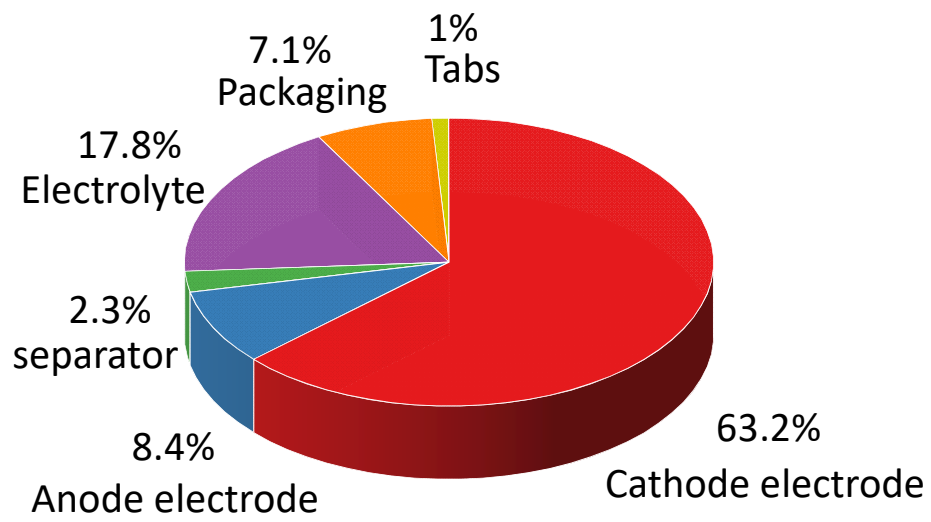

Supplementary Fig. 36 | Mass fractions of individual components in the 1.2 Ah Li||NCM811 pouch cell.

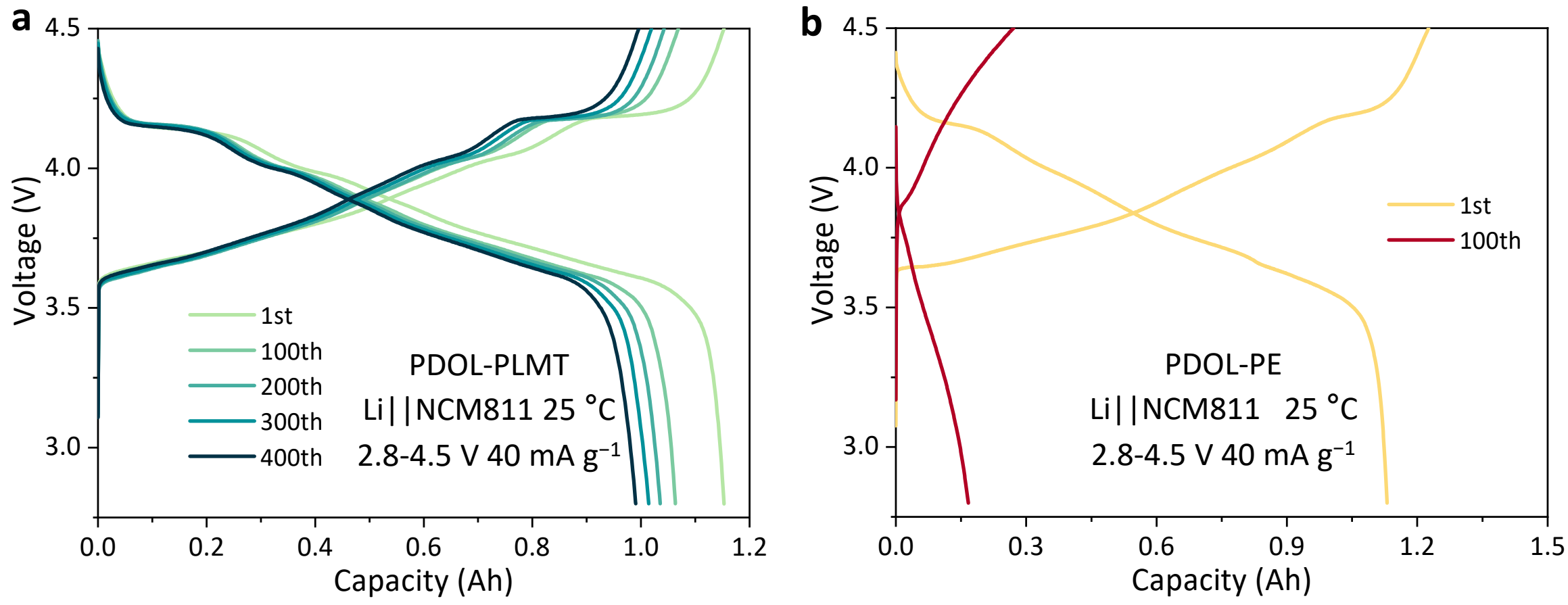

Supplementary Fig. 37 | Electrochemical performance of the 1.2 Ah practical-scale pouch cells at 4.5 V. Charge-discharge profiles of 1.2 Ah (**a**) Li|PDOL-PLMT|NCM811 and (**b**) Li|PDOL-PE|NCM811 pouch cells cycled between 2.8 − 4.5 V.

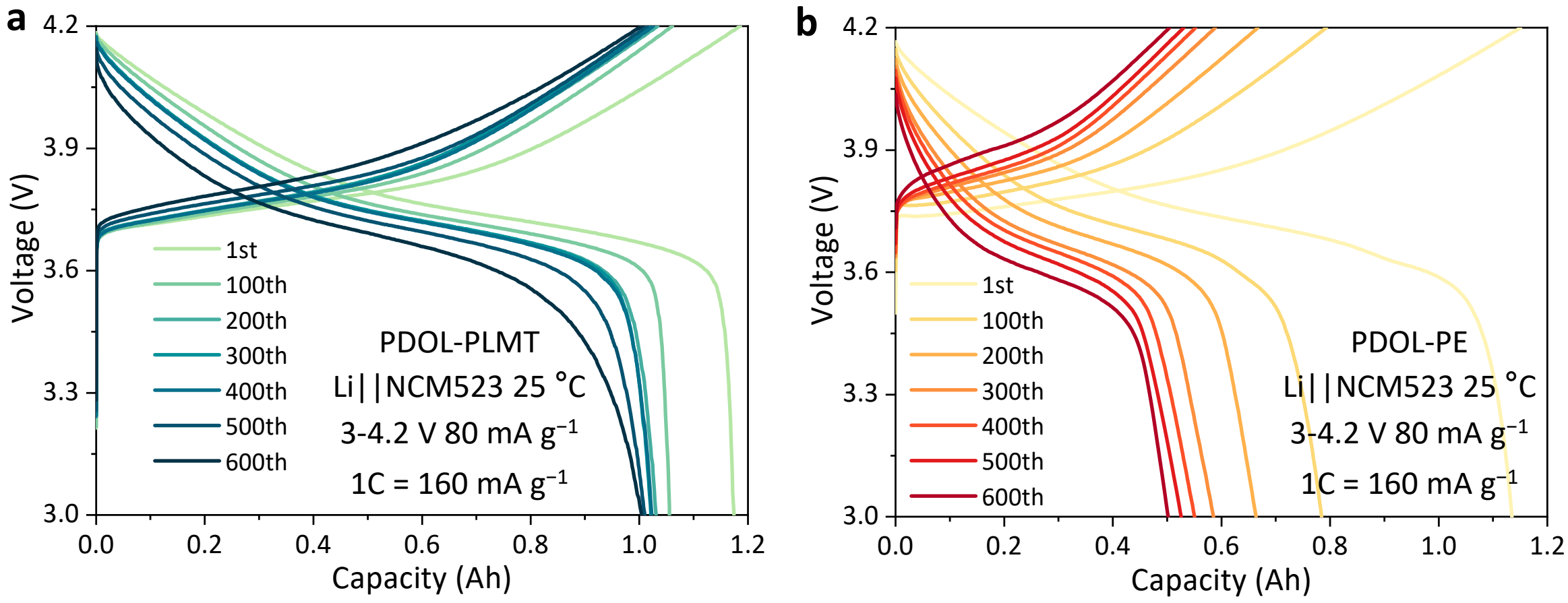

Supplementary Fig. 38 | Demonstration of the electrolyte universality in 1.2 Ah NCM523-based pouch cells. Charge-discharge profiles of (**a**) Li|PDOL-PLMT|NCM523 and (**b**) Li|PDOL-PE|NCM523 pouch cells cycled between 3 – 4.2 V.

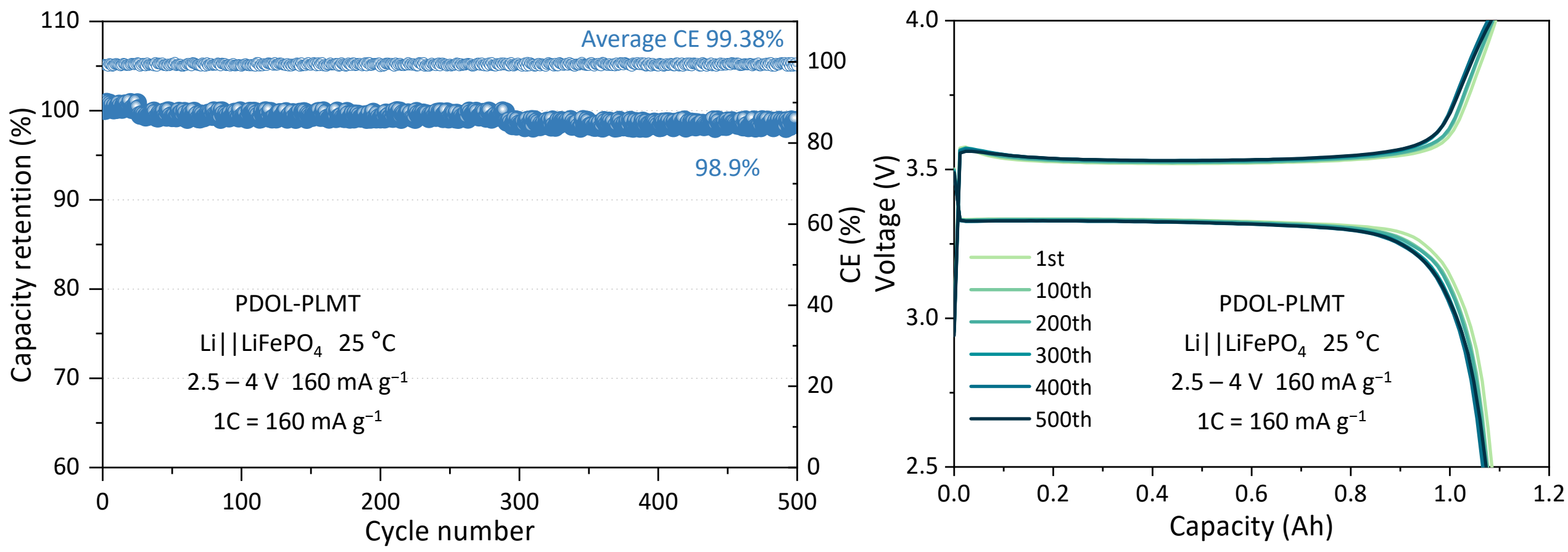

Supplementary Fig. 39 | Cycling performance and Charge-discharge profiles of the Li|PDOL-PLMT|$LiFePO_4$ pouch cell at 25 °C.

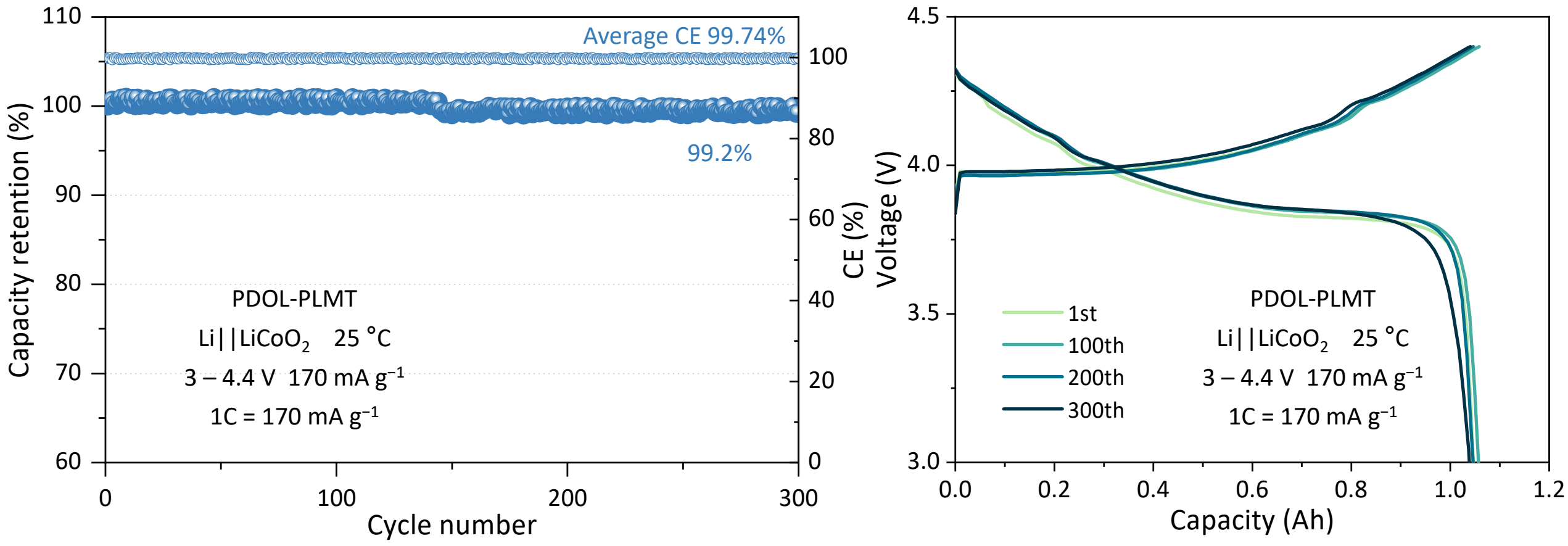

Supplementary Fig. 40 | Cycling performance and Charge-discharge profiles of the Li|PDOL-PLMT|$LiCoO_2$ pouch cell at 25 °C.

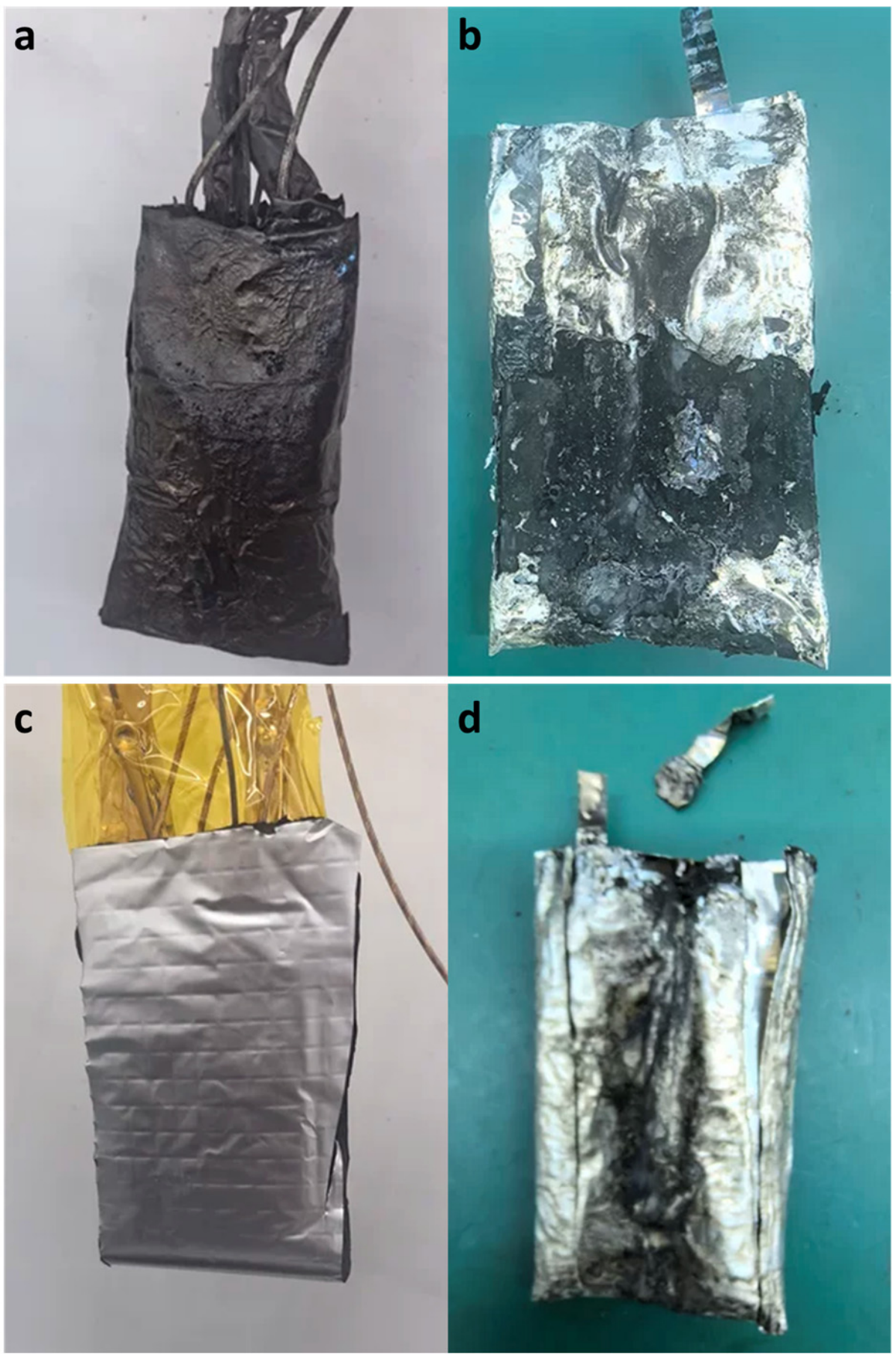

Supplementary Fig. 41 | Thermal stability and post-ARC morphological integrity of the pouch cells. Optical images of pouch cells after the ARC test: (**a**, **b**) Li|PDOL-PE|NCM811, (**c**, **d**) Li|PDOL-PLMT|NCM811.

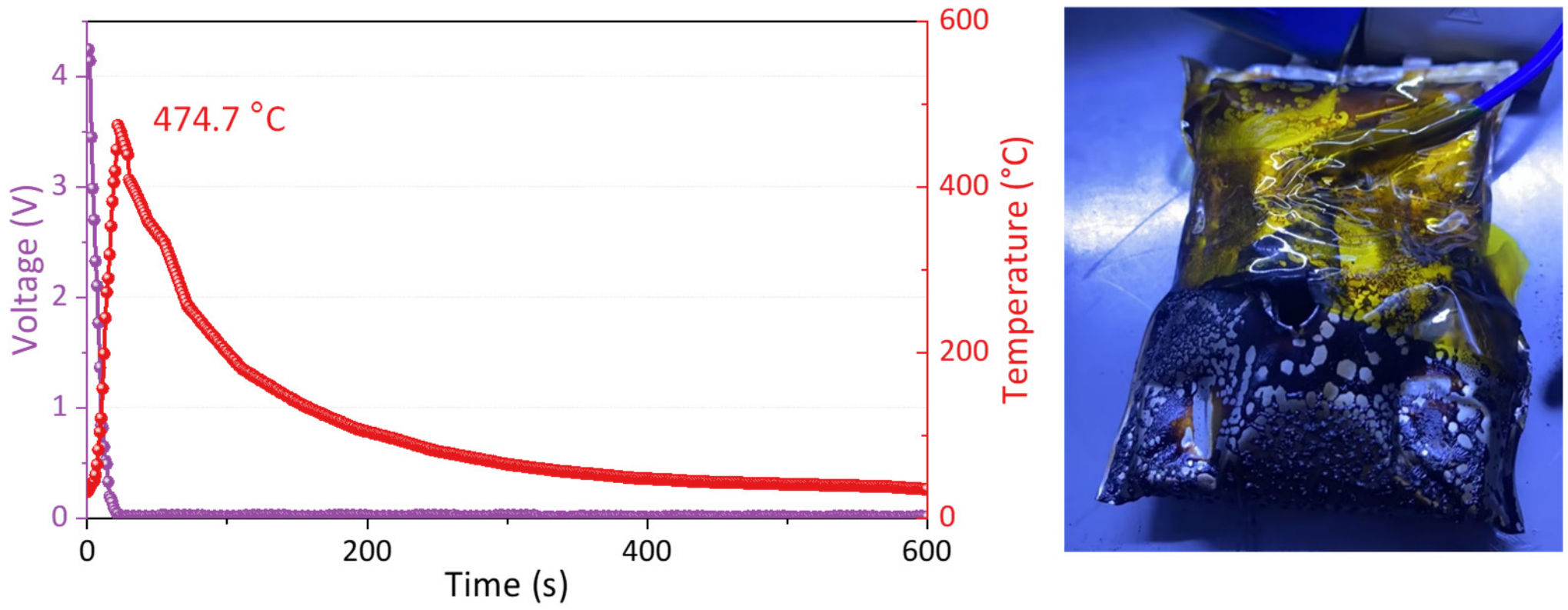

Supplementary Fig. 42 | Safety validation via nail penetration test under GB 38031-2020 standard. Optical photographs and real-time monitoring of the 1.2 Ah Li|PDOL-PE|NCM811 pouch cell during the nail penetration test, highlighting its thermal response and structural integrity under localized internal short-circuiting.

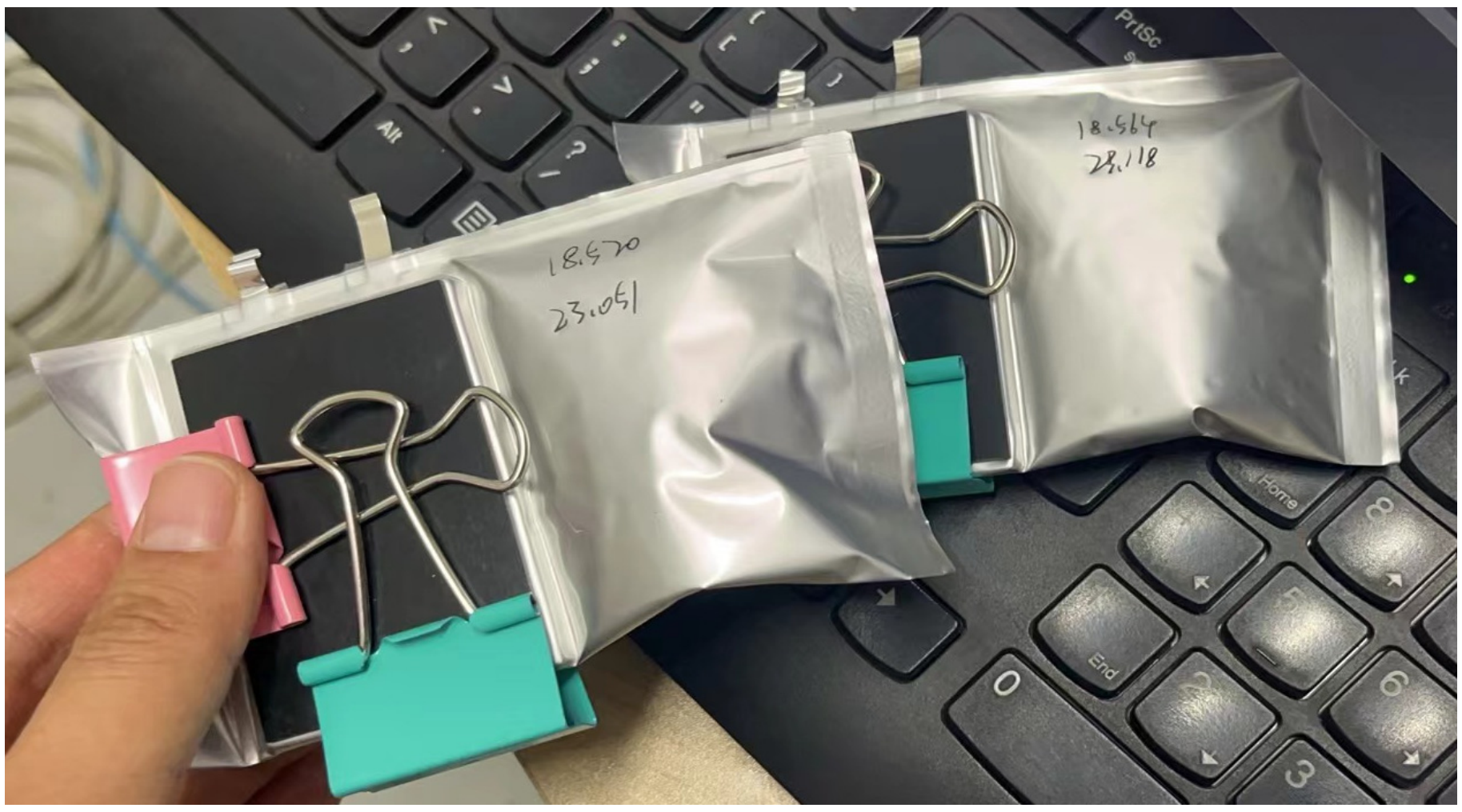

Supplementary Fig. 43 | Swelling observed in the Li|PDOL-PE|NCM811 pouch cell during formation cycle.

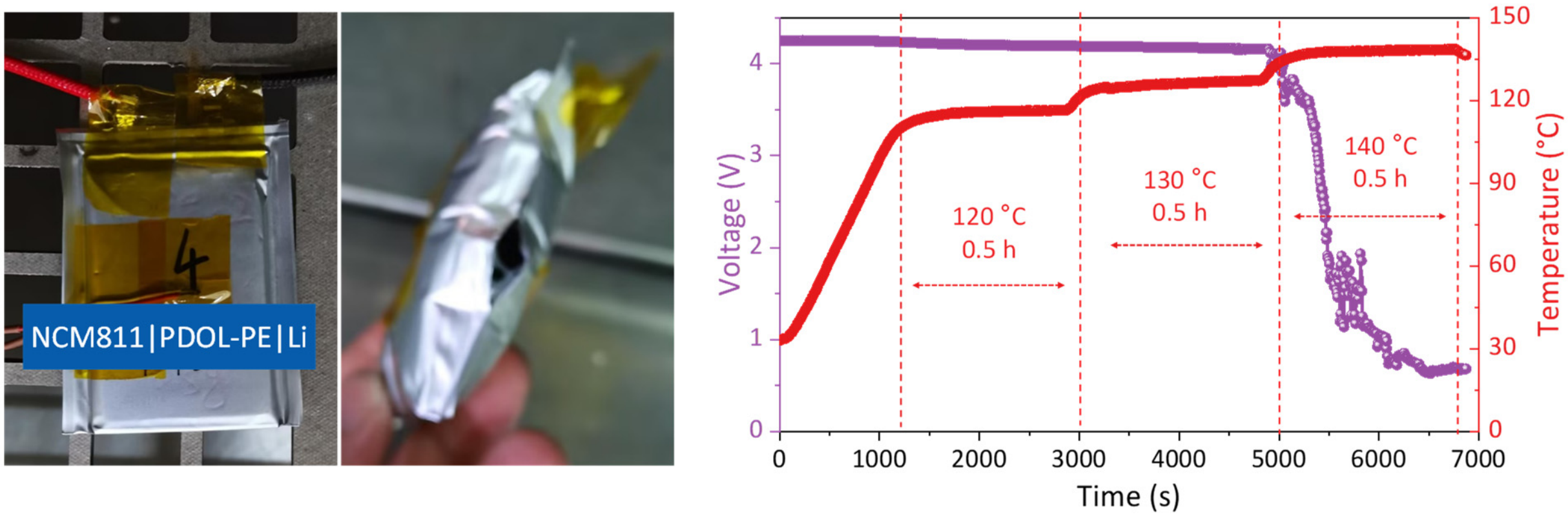

Supplementary Fig. 44 | Thermal stability validation under high-temperature abuse (GB 31241-2022). Optical images and temperature-time profiles of the 1.2 Ah Li|PDOL-PE|NCM811 pouch cell during the thermal chamber test.

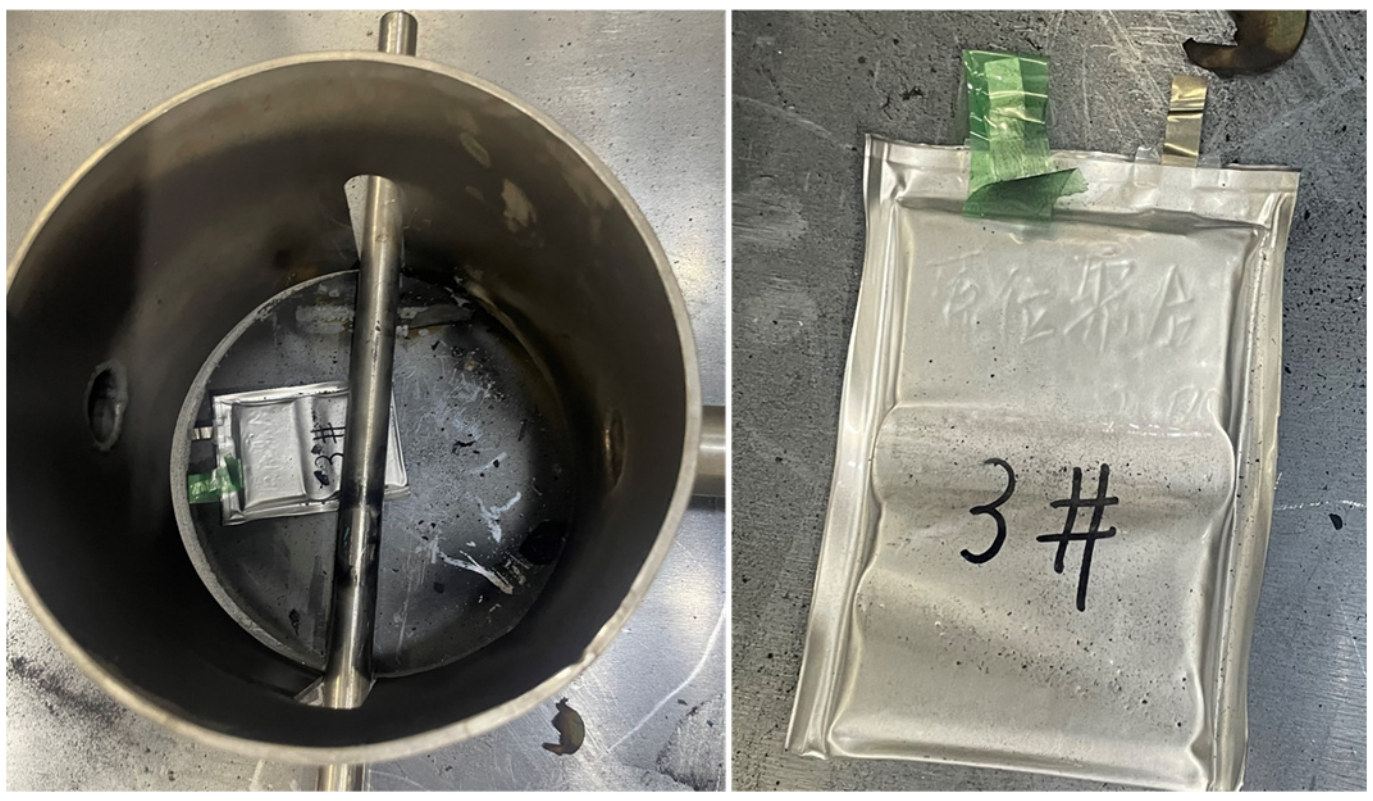

Supplementary Fig. 45 | Optical images of the 1.2 Ah Li|PDOL-PLMT|NCM811 pouch cell following the heavy impact test.

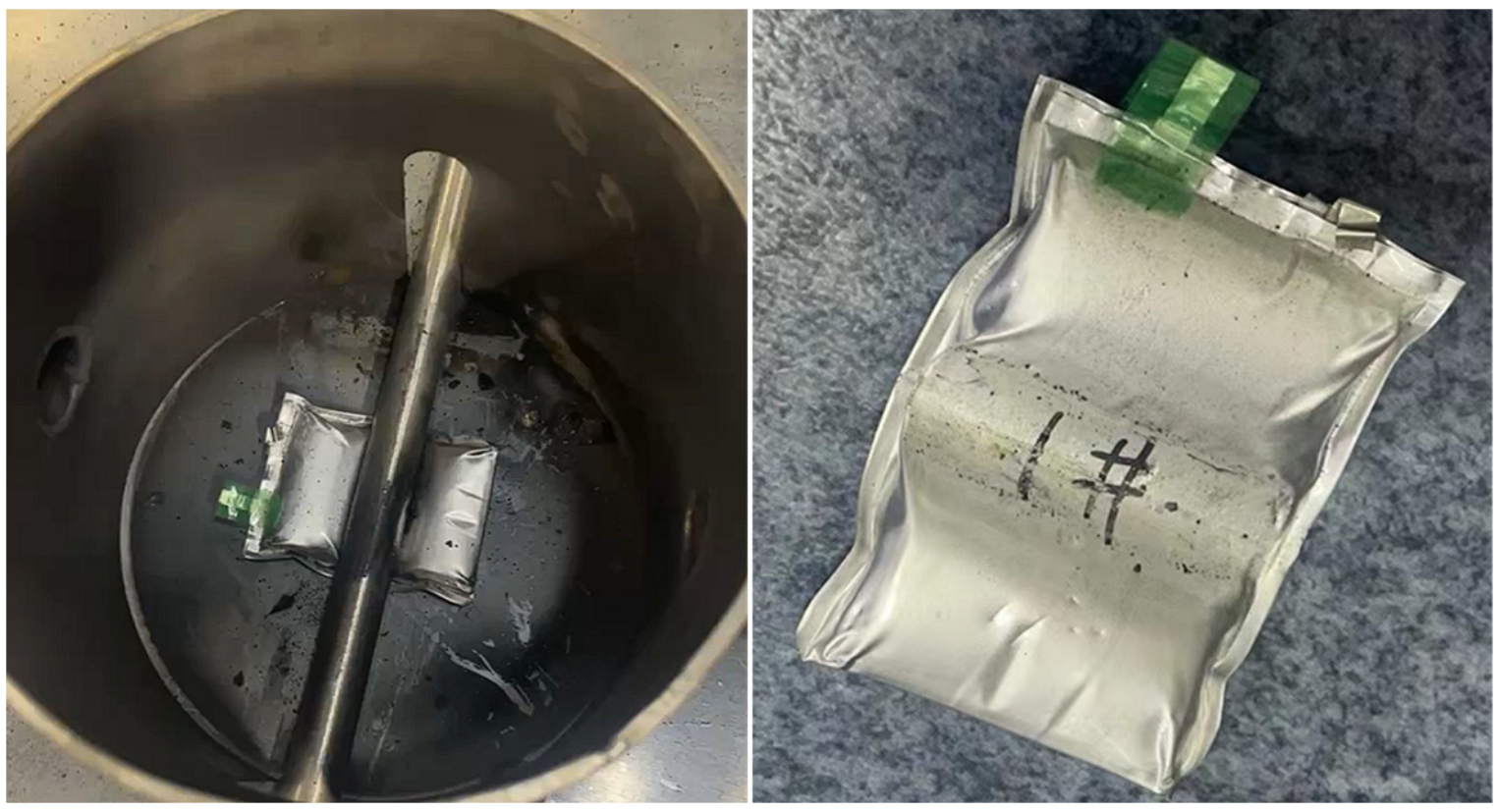

Supplementary Fig. 46 | Optical images of the 1.2 Ah Li|PDOL-PE|NCM811 pouch cell following the heavy impact test.

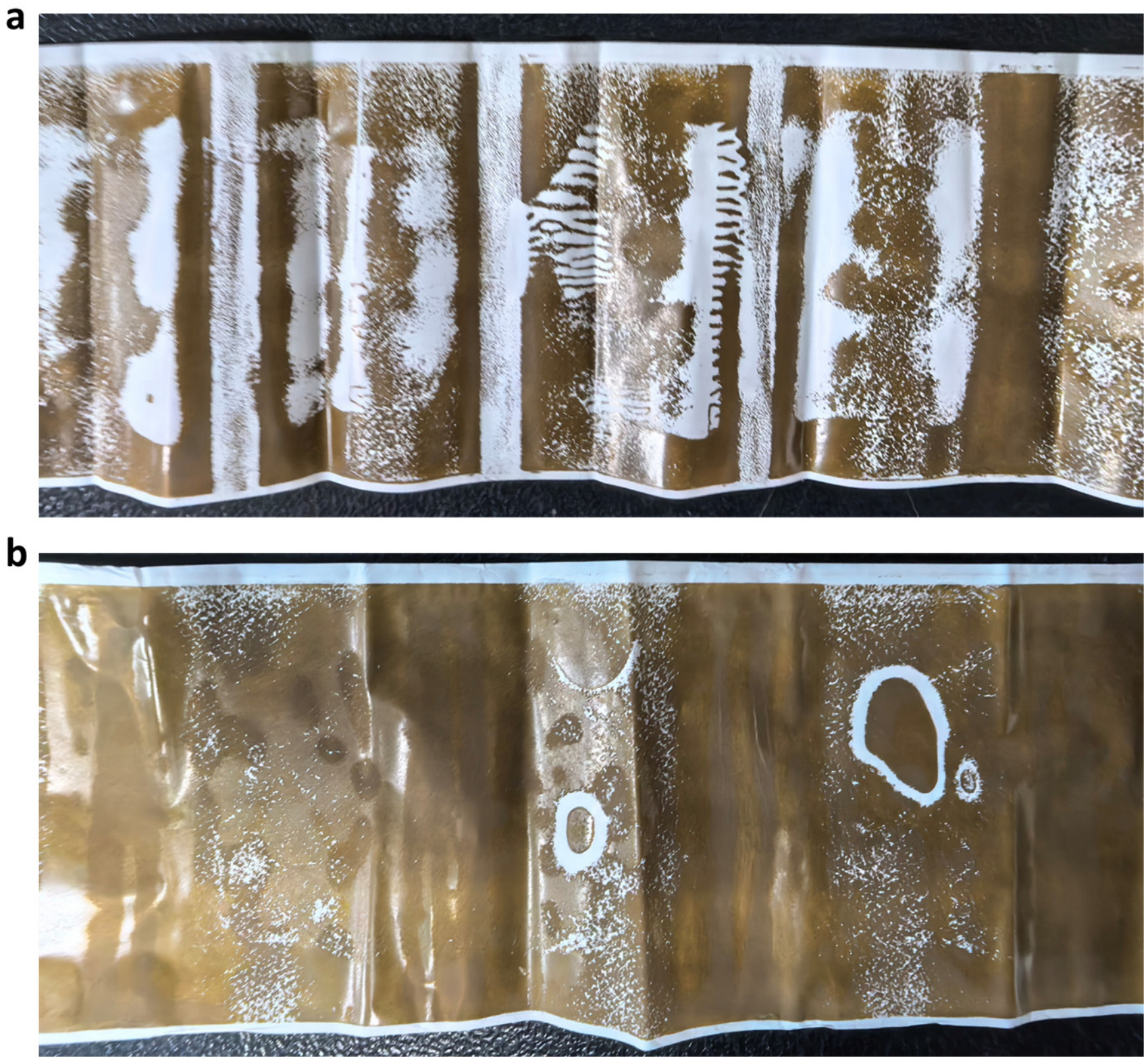

Supplementary Fig. 47 | Comparative visual analysis of the separators after 400 extended cycles. Optical images of the Li-facing side of separators harvested from (**a**) Li|PDOL-PLMT|NCM811 and (**b**) Li|PDOL-PE/LATP|NCM811 pouch cells after 400 cycles.

To evaluate the chemical stability of the LATP scaffold against the highly reductive Li metal, optical inspections of the separators were conducted post-cycling (Supplementary Fig. 47). Macroscopically, the LATP layer in the baseline PDOL-PE/LATP electrolyte turned noticeably black, a characteristic visual indicator of severe structural reduction. Conversely, the fully optimized PDOL-PLMT sample maintained its macroscopic integrity with only marginal discoloration. This stark visual contrast provides direct evidence that our engineered dual-additive system effectively mitigates the reductive degradation of the solid-state electrolyte interface.

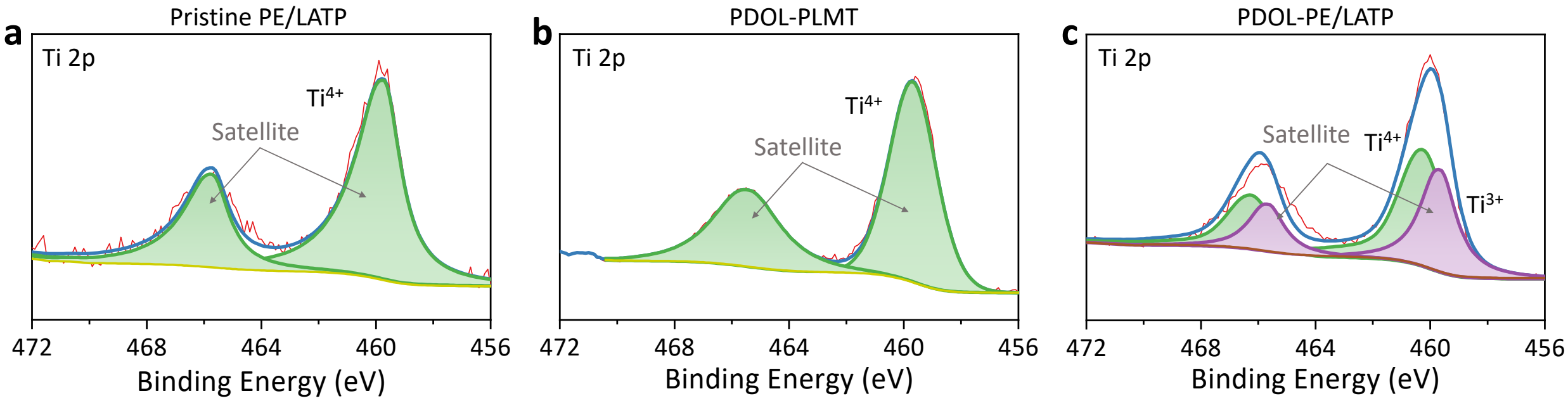

Supplementary Fig. 48 | XPS analysis of Ti valence states and interfacial reduction stability. Ti 2p XPS spectra of (**a**) the pristine PE/LATP separator surface, (**b**) the surfaces of PDOL-PLMT, and (**c**) PDOL-PE/LATP harvested from the respective Li||NCM811 pouch cells after cycling.

To quantitatively assess the reductive degradation of the solid-state electrolyte, high-resolution Ti 2p XPS analysis was performed on the Li-facing separator surfaces (Supplementary Fig. 48). While the pristine LATP displays exclusively Ti^{4+} oxidation states, the cycled baseline PDOL-PE/LATP separator exhibits a prominent Ti^{3+} signal, corroborating the severe reduction previously observed macroscopically. In sharp contrast, the cycled PDOL-PLMT separator maintains only the Ti^{4+} signal, identical to its pristine state. These spectroscopic results quantitatively demonstrate that the conformal PDOL polymer matrix successfully acts as an insulating barrier, restricting deleterious electron transfer to the LATP layer.

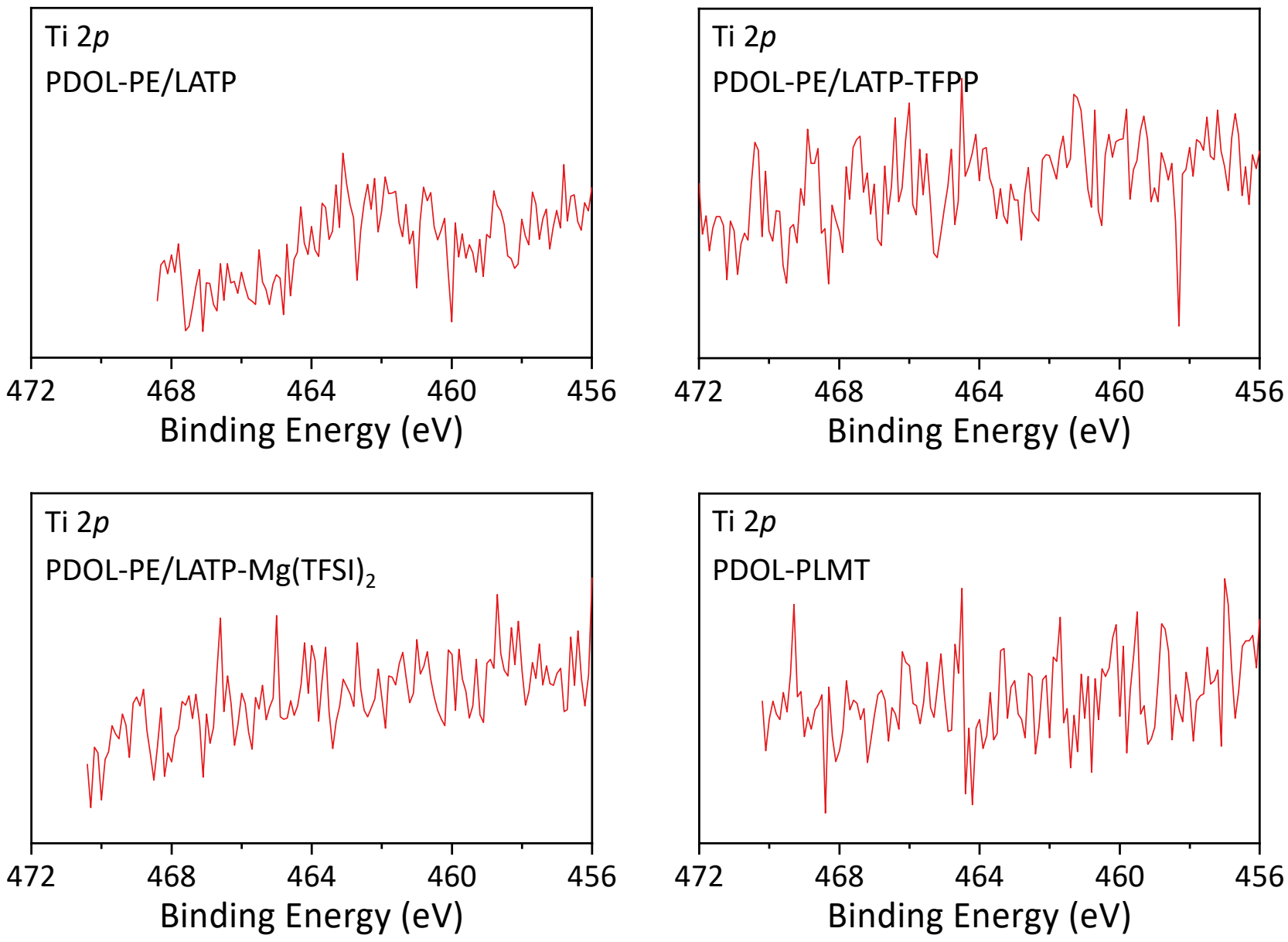

Supplementary Fig. 49 | Surface chemical analysis of the Ti cation crossover to the Li metal negative electrode. Ti 2p XPS spectra of the Li metal negative electrode surfaces after cycling in Li||NCM811 cells employing PDOL-PE/LATP, PDOL-PE/LATP-TFPP, PDOL-PE/LATP-$Mg(TFSI)_2$, and PDOL-PLMT.

To evaluate potential Ti crossover, the surfaces of cycled Li negative electrodes were examined via high-resolution Ti 2p XPS (Supplementary Fig. 49). Notably, no Ti signals were detected for any sample, including the baseline PDOL-PE/LATP. This absence of gross Ti migration highlights a distinct structural advantage of the in-situ polymerized architecture: rather than exposing ceramic particles as in conventional blended electrolytes, the dense PDOL matrix conformally coats the LATP fillers. This native encapsulation effectively averts direct contact with the Li metal, successfully preventing severe structural degradation and Ti dissolution.

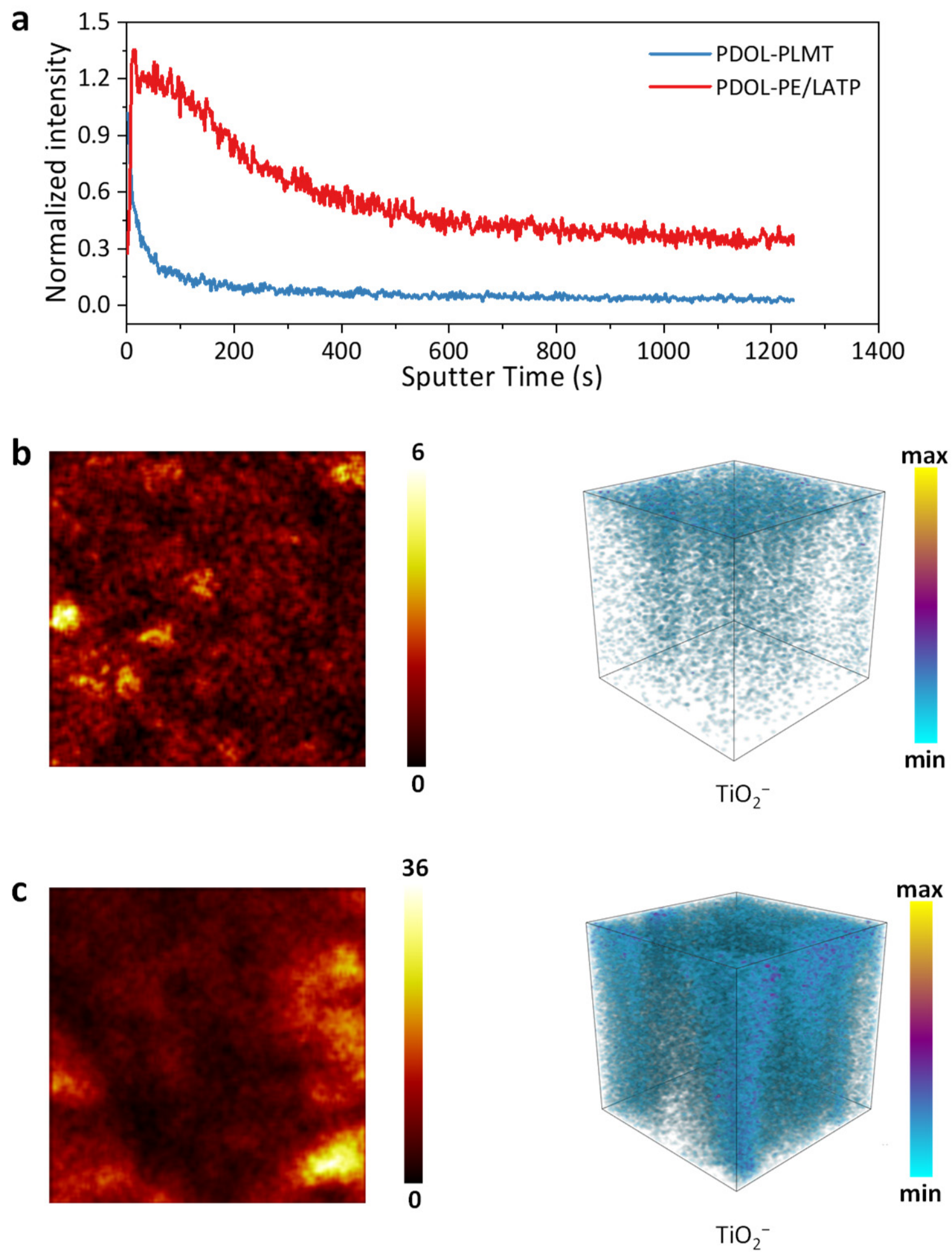

Supplementary Fig. 50 | 3D spatial distribution and confinement of Ti species. a Comparative depth profiles of TiO_2^- fragments on the Li metal negative electrode surfaces harvested from Li|PDOL-PLMT|NCM811 and Li|PDOL-PE/LATP|NCM811 pouch cells after 400 cycles. 2D and 3D TOF-SIMS mapping images of the TiO_2^- species distribution in the (**b**) PDOL-PLMT and (**c**) PDOL-PE/LATP samples.

While XPS analysis confirmed the absence of gross Ti accumulation (~0.1 at% detection limit), ultra-sensitive ToF-SIMS depth profiling was further employed to visualize the spatial distribution of trace Ti crossover (Supplementary Fig. 50). Tracking the characteristic TiO_2^- fragments reveal a stark contrast: the baseline PDOL-PE/LATP exhibits significant Ti signals penetrating deep into the

negative electrode bulk, suggesting that without protective additives, the LATP fillers remain susceptible to trace reductive attack. In sharp contrast, the optimized PDOL-PLMT system displays a nearly 'clean' bulk where the minimal TiO_2^- signal rapidly diminishes upon sputtering. These 3D distributions confirm that the engineered SEI effectively isolates the LATP scaffold, profoundly minimizing the generation and deep migration of reduced Ti species.

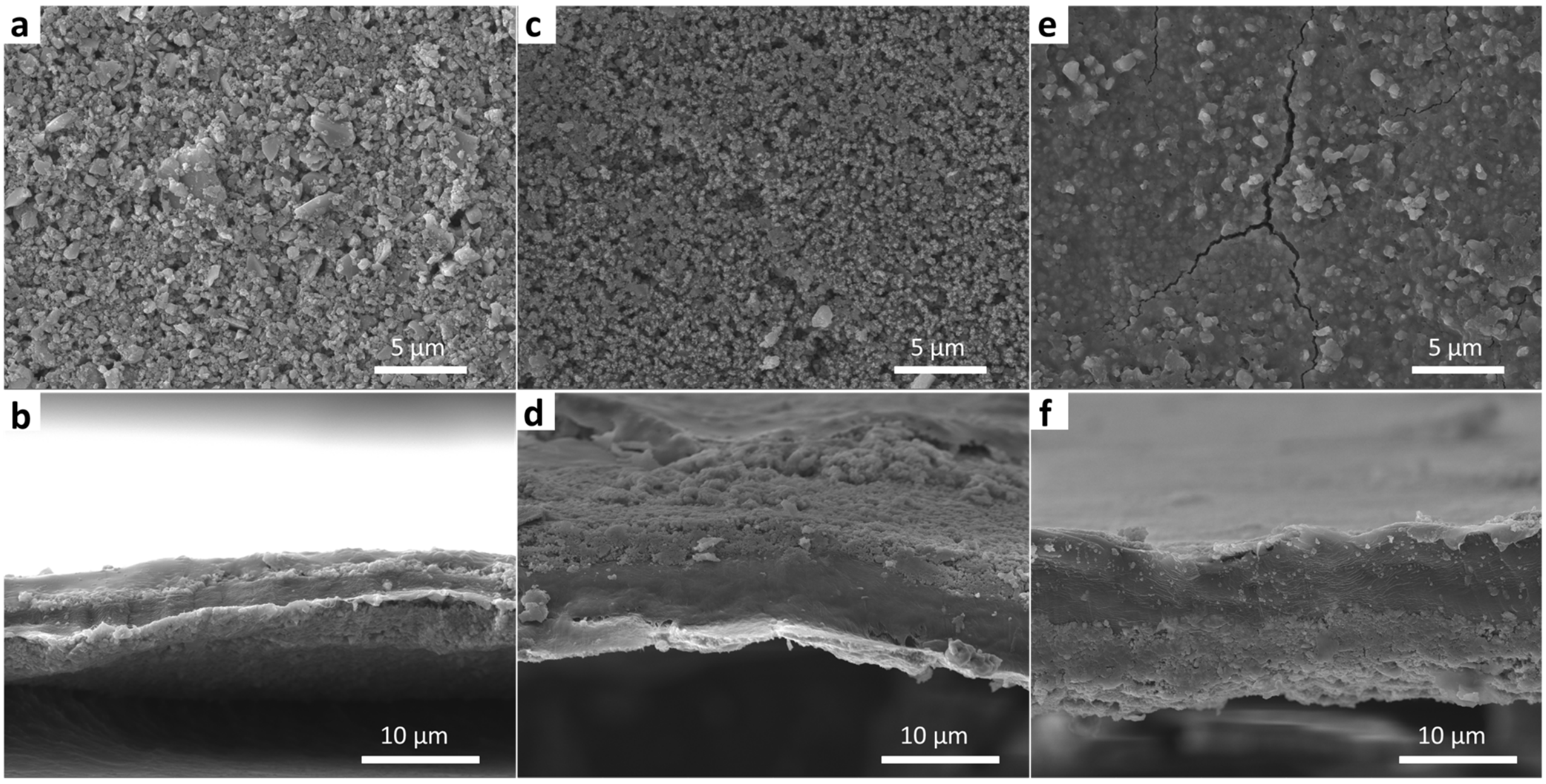

Supplementary Fig. 51 | Microstructural stability and morphological evolution of the composite separators. **a** Surface and (**b**) cross-sectional SEM images of the pristine PE/LATP separator. SEM images showing the (**c**) surface and (**d**) cross-section of the PE/LATP separator retrieved from the Li|PDOL-PLMT|NCM811 pouch cell after cycling (2.8–4.5 V, 40 mA g^{-1}, 25 °C). **e** Surface and (**f**) cross-sectional SEM images of the PE/LATP separator retrieved from the Li|PDOL-PE/LATP|NCM811 pouch cell after cycling (2.8–4.5 V, 40 mA g^{-1}, 25 °C).

To evaluate the mechanical robustness and post-cycling integrity of the solid-state electrolyte, cross-sectional SEM imaging was performed on the cycled PDOL-PLMT sample (Supplementary Fig. 51). The macroscopic structure of the LATP-coated scaffold is exceptionally well-preserved, completely devoid of the severe pulverization or delamination typically induced by interfacial stress. While a minor, surface-confined reaction is geometrically unavoidable, the conformal in-situ PDOL polymer matrix acts as an elastomeric buffer, absorbing interfacial fluctuations and preventing crack propagation. Crucially, rather than initiating a continuous degradation cycle, this localized reaction

yields a self-passivating mixed-conducting interphase (MCI). This native MCI acts in concert with our engineered SEI to spatially block further parasitic attack on the active lithium, ensuring that the capacity loss attributable to the LATP reduction remains negligible over extended operations.

Supplementary Tables

Supplementary Table 1 Specifications and test criteria for PE/LATP separators.

**Test item**	**Unit**	**Test standard**	**Typical value**
Thickness	µm	GB/T 6672-2001	8.1
Mass per unit area	g/m^2	GB/T 24218.1-2009	7.7
Air permeance	s/100ml	GB/T 458-2008	117
Puncture strength	gf	GB/T 23318-2009	447
Peel adhesion property	N/m	GB/T 2792-2014	44
Breakdown voltage	KV	/	0.8
Tensile property	TD(MPa)	GB/T 1040.3-2006	140
	MD(MPa)		160
Percentage elongation	TD (%)	GB/T 1040.3-2006	77
	MD (%)		60
Change on heating 120 °C * 1 h	TD (%)	GB/T 12027-2004	0.1
	MD (%)	/	0.1
Change on heating 130 °C * 1 h	TD (%)		0.1
	MD (%)		0.1
Change on heating 150 °C * 1 h	TD (%)		0.5
	MD (%)		0.7

Supplementary Table 2 Parameters of the 6-layer stacked Li|PDOL-PLMT|NCM811 pouch cell.

Cell component	Parameters	Value
**Positive electrode (NCM811)**	Reversible capacity (mAh g^{-1})	200
	Area (mm^2)	55*36*6 (layer)
	Areal Capacity (each side, mAh cm^{-2})	5
	Active material weight (each side, mg cm^{-2})	25
	Aluminum foil thickness (μm)	12
**Negative electrode (Li foil)**	Specific capacity (mAh g^{-1})	3860
	Area (mm^2)	58*39*7 (layer)
	Electrode thickness (double sides, μm)	100
	Areal capacity (each side, mAh cm^{-2})	10.3
**PDOL-PLMT (comprises 40% liquid electrolyte)**	E/C ratio (g Ah^{-1})	1.5

Supplementary Table 3 Detailed weight breakdown of components in the 1.2 Ah pouch cell.

Cell component	Specification	Mass (g)	Mass Fraction
Positive electrode electrode	High loading (25 mg cm^{-2}, double-sided)	6.3305	63.21%
Negative electrode electrode	Thin Li foil (100 μm）	0.8455	8.44%
Separator	Ultrathin (~8 μm)	0.234	2.34%
Electrolyte	PDOL-based SPE	1.785	17.82%
Packaging	Ultrathin Al-laminate film (68 μm)	0.716	7.15%
Tabs	Al/Ni tabs (Ultra light)	0.104	1.04%
Total		10.015	100.00%

The energy density was calculated based on the total mass of the entire pouch cell, which includes the active materials of the positive electrode and negative electrode, conductive additive, current collectors, separator, electrolyte, and the aluminum-plastic pouch, using the following equations:

Gravimetric energy density (Wh kg^{-1}) = specific capacity (mA h g^{-1}) × average voltage (V) =

$$\frac{capacity\ of\ the\ full\ cell}{weight\ of\ (cathode\ +\ anode\ +\ collector\ +\ separate\ +\ pouch)} \times average\ voltage\ (v)$$

Gravimetric specific power density (W kg^{-1}) = Gravimetric energy density (Wh kg^{-1}) × C rate (h^{-1})

Gravimetric energy density = $\frac{1188\ mAh}{10.015\ g} \times 3.85\ V\ = 456.7\ Wh\ kg^{-1}$

Supplementary Table 4 Energy density and power densities of the Li|PDOL-PLMT|NCM811 pouch cell at various C-rates (calculated based on total cell mass and electrode mass, respectively).

	**Based on whole pouch cell**		**Based on the electrode mass**	
**C-rate**	Energy density (Wh kg^{-1})	Power density (W kg^{-1})	Energy density (Wh kg^{-1})	Power density (W kg^{-1})
0.2	456.7	91.3	770	154
0.5	450.4	225.2	759.5	379.7
1	438.8	438.8	739.8	739.8
2	424.5	849.0	715.7	1431.4
3	414.7	1244.1	699.2	2097.6
5	395.2	1976.0	666.3	3331.5
10	150.1	1500.7	253.0	2530.0

Supplementary Table 5 Performance of high-energy-density Li||NCM811 metal batteries in this work and literature.

Positive electrode	Electrolyte	Negative electrode	Cyclability	Gravimetric Energy Density (Wh Kg-1)	Volumetric Energy Density (Wh L^{-1})	Power Density (W Kg^{-1})	Ref
NCM811	3D-SIPE-LiFPA	Li	60 cycles (95.4%)	437.0 (0.3C)	/	131.1 (0.3C)	[1]
NCM811	PVCM	Si/C@C-Gr	2000 cycles (88.7%)	325.9 (0.5C)	/	1463.5 (5C)	[2]
NCM811	LPSC	Si	1000 cycles (62.9%)	285.0 (0.05C)	/	177.0 (1C)	[3]
NCM811	PELL60	Li	80 cycles (90.3%)	310.0 (0.1C)	/	31.0 (0.1C)	[4]
NCM811	HCPE	Li	110 cycles (80%)	246.0 (0.1C)	/	24.6 (0.1C)	[5]
NCM811	Liquid electrolyte	Li	100 cycles (87.9%)	300.0 (0.5C)	/	150.0 (0.5C)	[6]
NCM811	Liquid electrolyte	Li	100 cycles (81.0%)	310.0 (0.4C)	/	410.0 (2C)	[7]
NCM811	PDOL-PLMT	Li	400 cycles (85.9%)	456.7 (0.2C)	911.1 (0.2C)	1976.0 (5C)	This work

Supplementary References

1. Zhang S, et al. In situ-polymerized lithium salt as a polymer electrolyte for high-safety lithium metal batteries. *Energy & Environmental Science* **16**, 2591-2602(2023).

2. Bai M, et al. An in-situ polymerization strategy for gel polymer electrolyte Si||Ni-rich lithium-ion batteries. *Nature Communications* **15**, 5375(2024).

3. Cao D, Sun X, Li Y, Anderson A, Lu W, Zhu H. Long‐cycling sulfide‐based all‐solid‐state batteries enabled by electrochemo‐mechanically stable electrodes. *Advanced Materials* **34**, (2022).

4. Zhu L, et al. Tunneling interpenetrative lithium ion conduction channels in polymer-in-ceramic composite solid electrolytes. *Journal of the American Chemical Society* **146**, 6591-6603(2024).

5. Boaretto N, et al. Hybrid ceramic polymer electrolytes enabling long cycling in practical 1 Ah‐class high‐voltage solid‐state batteries with Li metal negative electrode. *Advanced Functional Materials*, (2024).

6. Zhao Q, et al. Upgrading Carbonate Electrolytes for Ultra‐stable Practical Lithium Metal Batteries. *Angewandte Chemie International Edition* **61**, e202116214(2022).

7. Xia Y, et al. Designing an asymmetric ether-like lithium salt to enable fast-cycling high-energy lithium metal batteries. *Nature Energy* **8**, 934-945(2023).
