# Supplementary material for: A catalytically polymerized solid electrolyte enables 450 Wh kg−1 lithium–metal batteries with thermal–mechanical abuse tolerance
Source: Nat Commun. 2026 Aug 8;17:9545. doi: 10.1038/s41467-026-76381-y (PMC13547571; doi:10.1038/s41467-026-76381-y)
Supplement: Supplementary file 2 [file 41467_2026_76381_MOESM2_ESM.pdf]

Description of Additional Supplementary Files

Supplementary Data 1: The optimized structure of TFPP.

Supplementary Data 2: The optimized structure of TPFPP.

Supplementary Data 3: The optimized structure of TPP.

Supplementary Data 4: The optimized structure of TPPa.

Supplementary Data 5: The optimized structure of TTFP.

Supplementary Data 6: The optimized structure of PDOL-PE/LATP system.

Supplementary Data 7: The optimized structure of PDOL-PLMT system.
