# Supplementary material for: A catalytically polymerized solid electrolyte enables 450 Wh kg−1 lithium–metal batteries with thermal–mechanical abuse tolerance
Source: Nat Commun. 2026 Aug 8;17:9545. doi: 10.1038/s41467-026-76381-y (PMC13547571; doi:10.1038/s41467-026-76381-y)
Supplement: Supplementary file 3 [file 41467_2026_76381_MOESM4_ESM.pdf]

nature portfolio

Peer Review File

A Catalytically Polymerized Solid Electrolyte Enables 450 Wh kg−1 Lithium–Metal Batteries with Thermal–Mechanical Abuse Tolerance

Corresponding Author: Professor Yue Ma

This file contains all reviewer reports in order by version, followed by all author rebuttals in order by version.

Version 0:

Reviewer comments:

Reviewer #1

(Remarks to the Author)
In this work, the authors fabricate an ultrathin (≈9.6 μm) PDOL-based solid polymer electrolyte (SPE) by inducing in situ ring-opening polymerization (ROP) of 1,3-dioxolane (DOL) within a micro-grooved scaffold consisting of polyethylene (PE) coated on both sides with LATP nanoparticles. In addition, a dual-additive approach (TFPP and $Mg(TFSI)_2$) is introduced to stabilize both the high-voltage NCM811 cathode and the Li metal anode interfaces. The demonstration of an Ah-scale (1.2 Ah) pouch-format cell together with industry-relevant safety tests (e.g., ARC and nail penetration) is application-oriented and potentially impactful. In particular, the reported combination of high ionic conductance (418.7 mS) and high tensile strength (191.7 MPa) in an ultrathin SPE addresses a key "performance–manufacturability–safety" triad that is of strong interest to top-tier journals.
However, several central claims namely "trade-off breaking," "dual interfacial stabilization," "transition-metal (TM) crossover suppression," and "intrinsic safety" are not yet fully supported at a Nature Communications standard. Mechanistic evidence remains largely qualitative, and critical practical cell metrics are insufficiently disclosed. In addition, the necessity and novelty of the selected additives (TFPP, $Mg(TFSI)_2$) are not convincingly established. A more rigorous ablation study, quantitative validation of TM dissolution/cross-talk suppression, and a dedicated assessment of LATP stability in a Li-metal environment are required.

1. Insufficient validation of LATP stability against Li metal (chemical/structural)
LATP is widely recognized to exhibit reductive instability in contact with Li metal, potentially leading to Ti reduction, structural degradation, and continuous Li consumption via parasitic interfacial reactions. Although the authors position LATP as a "catalytic scaffold + mechanical reinforcement + ion-conduction pathway," the long-term stability of the LATP-containing scaffold under cycling conditions constitutes a key risk for both durability and Coulombic efficiency.
Suggested revisions/experiments :
• Characterize the structural and chemical-state evolution of LATP before/after cycling (e.g., XRD or Raman; XPS Ti 2p for oxidation-state changes).
• Verify whether Ti-containing species appear at the Li-side interface after cycling (e.g., XPS depth analysis on the anode surface).
• Provide cross-sectional SEM/TEM evidence to assess cracking, delamination, and morphological collapse of the LATP-coated layer after prolonged cycling and/or under pressure conditions relevant to pouch-cell stacking.

2. Apparent inconsistency between the proposed CEI-centered role of TFPP and its expected reactivity toward Li metal (SEI contribution)
From an electronic-structure perspective (HOMO/LUMO), TFPP appears likely to undergo reduction at the Li metal interface and thus may substantially influence SEI chemistry. Prior literature also reports TFPP-like additives altering SEI composition (often toward Li_2CO_3/LiF-rich SEI). In contrast, the present manuscript primarily attributes TFPP function to cathode-side CEI formation and TM crossover suppression. If anode-side XPS indicates reduced LiF and increased $ROCO_2Li$-like species, this may contradict the intuitive expectation that fluorinated motifs would promote LiF enrichment, and it raises questions about TFPP's dominant reaction pathway in this system.
Moreover, if TFPP's HOMO level is not significantly higher than alternative high-voltage cathode additives, the rationale for selecting TFPP as a cathode-focused additive is not sufficiently justified. Without a clearer mechanistic and comparative

justification, TFPP may be perceived mainly as an anode-reactive additive, weakening the manuscript's claim of TFPP-driven CEI stabilization and cathode protection.
Suggested revisions/experiments :
• Provide a direct, systematic assessment of TFPP's impact on SEI composition and evolution, including quantitative/semi-quantitative comparisons after formation and after long-term cycling.
• If TFPP is claimed to be critical for CEI and TM control, the manuscript must include quantitative evidence of TM dissolution/cross-talk suppression.
• Strengthen the justification of TFPP as a cathode additive by comparing against (or at minimum discussing) benchmark high-HOMO cathode additives, and clarifying why TFPP is uniquely effective under the proposed polymer-electrolyte chemistry.

3. Lack of an ablation study to disentangle the "dual additive" contributions
The current comparison appears centered on PE vs LATP vs PLMT. Under such a design, it is difficult to isolate the respective contributions of TFPP and $Mg(TFSI)_2$. This is a critical weakness because the manuscript's main narrative relies on the claim that the dual-additive strategy simultaneously stabilizes both interfaces.
Suggested revisions/experiments:
• Include at least two additional control groups: LATP + TFPP (without $Mg(TFSI)_2$) and LATP + $Mg(TFSI)_2$ (without TFPP), evaluated in full cells (and ideally pouch cells) with cycling and rate performance.
• Provide corresponding interfacial characterization (CEI/SEI) for each ablation condition to support the proposed division of labor (TFPP → CEI; $Mg(TFSI)_2$ → Li–Mg interphase).

4. Insufficient disclosure of practical pouch-cell metrics and incomplete electrochemical reporting
For claims of high gravimetric/volumetric energy density (Wh kg^{-1}, Wh L^{-1}) in Ah-scale pouch cells, it is essential to disclose the cell design metrics that govern practical relevance. At minimum, N/P ratio, electrolyte amount (E/C, g Ah^{-1}), and stack pressure must be reported explicitly. Additionally, presenting capacity retention alone is not sufficient for evaluating cell-level performance.
Suggested revisions/experiments:
• Report N/P ratio, E/C ratio (g Ah^{-1}), and stack pressure used during assembly and cycling.
• Provide plots of capacity vs cycle number, along with coulombic efficiency and voltage polarization evolution.

5. Discrepancy between Li symmetric-cell behavior (higher overpotential/impedance) and superior full-cell rate performance
In the Li symmetric cell tests, PLMT appears to exhibit the largest resistance (or overpotential), which could imply poor rate capability. However, PLMT shows the best rate performance in full cells, which is not consistent with the symmetric-cell results. Therefore, the authors should explain the origin of this discrepancy.

6. Claims of TM dissolution/cross-talk suppression lack quantitative validation
The manuscript implies that TFPP-derived CEI inhibits TM crossover and mitigates cathode-driven degradation. However, presenting a scheme, CEI thickness, and limited microscopy alone is insufficient to substantiate a reduction in TM dissolution and cross-talk especially for Ni-rich NCM811, where TM dissolution and surface reconstruction critically govern long-term fading.
Suggested revisions/experiments:
• Quantify dissolved TM species (Ni/Mn/Co) in the electrolyte using ICP-MS (or equivalent) after defined cycling protocols.
• Quantify TM deposition at the anode via XPS/ToF-SIMS depth profiling (semi-quantitative comparisons across conditions).
• Examine cathode structural degradation after cycling, including particle cracking/pulverization and surface rock-salt reconstruction (cross-sectional SEM/TEM, HRTEM where feasible).

7. Insufficient interpretation of LSV behavior, especially the sharp current rise near ~4.8 V in LATP–TFPP
Because electrochemical stability window expansion is a key claim, the LSV interpretation must be rigorous. A rapid current increase near ~4.8 V for the LATP–TFPP condition could be perceived as early oxidation/decomposition rather than improved stability, especially if other controls show more gradual current onset. The manuscript needs to clarify whether this feature corresponds to TFPP sacrificial oxidation forming a protective CEI, or to undesirable electrolyte decomposition.
Suggested revisions/experiments (recommended, likely necessary):
• Provide a clear explanation of the LSV onset criteria, electrode configuration, and scan conditions, and interpret the current rise with supporting evidence.
• If the sharp rise reflects sacrificial oxidation, correlate it with CEI formation evidence.

Reviewer #2

(Remarks to the Author)
The manuscript by Tang et al. reports a catalytically designed Solid Polymer Electrolyte (SPE) using a poly-DOL/LiTFSI system integrated with LATP and TFPP. The reported performance, specifically the cycling stability of a 450 Wh/kg, 1.2 Ah pouch cells at a high voltage of 4.5V, is very impressive and surprising. Based on the reviewers understanding, poly-DOL SPE would face stability challenges towards high-voltage cathode and Li metal anode. The current version lacks several critical data required to support such extraordinary claims. The following points should be addressed before further consideration.
1. Since the electrolyte system is essentially ether-based, its operation at 4.5V presents a huge challenge. To justify the claimed high-voltage tolerance, the authors should provide leakage current measurements. Specifically, potentiostatic hold tests (leakage current) should be performed on full cells held at a constant 4.5V. And comparisons between the steady

current of different system are also important.
2. The manuscript just gived capacity retention data regarding the electrochemical behavior of the pouch cells. The charge-discharge profiles and corresponding Coulombic efficiency data, for all pouch cells paired with LFP, LCO, and NCM should be provided.
3. Poly-dol system may face instability towards Li metal anode. To rigorously evaluate the lithium plating/stripping efficiency, the authors should provide Li||Cu asymmetric cell data. Comparisons between different SPEs are also needed. Recommended parameters: Current density 0.5 mA cm-2 and an areal capacity of 5 mAh cm-2.
4. There is an unit error of the reported room-temperature ionic conductivity. The authors should correct units and disclose the electrolyte thickness and the bulk resistance derived from EIS.

Reviewer #3

(Remarks to the Author)
This manuscript (NCOMMS-25-105486-T) presents an in-situ polymerization approach to fabricate ultrathin (<10 μm) SPEs, utilizing LATP fillers on a PE separator to catalyze DOL polymerization. Through a dual-additive formulation (TFPP and Mg(TFSI)2), the work targets interfacial stability on both NCM811 cathodes and Li-metal anodes. A key strength of this study is the successful evaluation of 1.2 Ah pouch cells, which demonstrate high energy density (456.7 Wh kg-1) and resilience under safety abuse testing. While the performance is encouraging, the logical connection between the mechanistic investigation and the full-cell results is not fully established. I have identified several significant deficiencies in the experimental design and calculation that require major revisions.
1. Although Figures 2 and 3 effectively isolate the mechanistic roles of TFPP and Mg(TFSI)2, the battery performance in Figure 4 lacks a necessary comparison with these single-additive systems. To conclusively prove the synergistic effect, the authors are requested to provide cycling data for the PDOL-PE/LATP-TFPP and PDOL-PE/LATP-Mg(TFSI)2 configurations, demonstrating that the dual-additive strategy significantly outperforms the individual approaches.
2. Although the Supporting Information (lines 130-138) states the total cell mass (~10 g), this is unconvincing for a 1.2 Ah pouch cell. Given that the NCM811 cathode required for this capacity already accounts for the majority of the mass, the remaining weight allowance seems insufficient for the necessary inactive components (Al-laminated film, tabs, etc.). To validate the claimed 456.7 Wh kg-1, the authors must provide a table detailing the specific weight of each component (cathode, Li anode, electrolyte/separator, current collectors, tabs, and packaging film) to prove the mass data is realistic.
3. To rigorously validate the high-voltage performance, the provided LSV data is insufficient. The authors are advised to perform high-voltage electrochemical floating tests (monitoring leakage current over time at 4.7 V or 4.8 V) to demonstrate that the electrolyte remains stable under sustained oxidative stress.
4. There are several typos in the manuscript. For instance, in Equation 1, aera should be corrected to area. The authors should carefully check the entire text to eliminate similar errors.
5. The current evaluation relies on Li/Li symmetric cells, which utilize an infinite Li that can mask irreversible Li loss. To rigorously assess the anode reversibility and the specific efficacy of the $Mg(TFSI)_2$ additive in suppressing dead lithium formation, the cycling performance of Li/Cu cells are highly recommended.
6. The authors should conduct Molecular Dynamics simulations to determine whether the additives participate in Li+ coordination.
7. The TFPP additive causes a significant irreversible capacity loss (43 mAh g-1), leading to a low initial Coulombic efficiency (< 75%), which is concerning for practical cells. The authors must explicitly state the N/P ratio of the 1.2 Ah pouch cells and critically discuss whether this high Li consumption is viable for lean-Li battery designs.
8. Figure S10 and Line 239 indicate a low initial Coulombic efficiency due to TFPP decomposition (43 mAh g-1 loss). However, the pouch cell cycling data (e.g., Figure 5c) does not seem to reflect such a severe capacity loss in the first cycle, which may be due to the undisclosed formation step. Please define the formation protocol (C-rate, voltage range, cycles) used for the pouch cells in Methods.

Version 1:

Reviewer comments:

Reviewer #1

(Remarks to the Author)
The authors revised the manuscript carefully, I recommend acceptation of this revised manuscript.

Reviewer #2

(Remarks to the Author)
The authors have addressed my questions.
I support its publication in this version.

Reviewer #3

(Remarks to the Author)
The authors have addressed most of my comments satisfactorily. However, I noticed that several figures (such as Supplementary Figure 28-31) in the Supplementary Information are neither cited in the main text nor accompanied by descriptive text within the SI document itself. The authors should check all the Figures and revise the text to direct the

readers to these supplemental results where relevant.

Open Access This Peer Review File is licensed under a Creative Commons Attribution 4.0 International License, which permits use, sharing, adaptation, distribution and reproduction in any medium or format, as long as you give appropriate credit to the original author(s) and the source, provide a link to the Creative Commons license, and indicate if changes were made.
In cases where reviewers are anonymous, credit should be given to 'Anonymous Referee' and the source.
The images or other third party material in this Peer Review File are included in the article's Creative Commons license, unless indicated otherwise in a credit line to the material. If material is not included in the article's Creative Commons license and your intended use is not permitted by statutory regulation or exceeds the permitted use, you will need to obtain permission directly from the copyright holder.
To view a copy of this license, visit https://creativecommons.org/licenses/by/4.0/

General Response to the Concerns from the Editor and Reviewers' comments

First and foremost, we would like to express our sincere gratitude to the editor and reviewers for their sharp and constructive assessment. We fully acknowledge that the reviewers' critique regarding the mechanistic qualitative nature, and insufficient control experiments and practical metrics, which have profoundly inspired us to further polish and improve the manuscript.

Therefore, strictly following the editor and reviewer's constructive comments and guidance, we have treated this revision as a comprehensive reconstruction of our evidence chain. We have not only supplemented substantial data but also reshaped the logical narrative to bridge the gap between our "performance claims" and "underlying mechanisms." Specifically, our major revisions focus on three aspects:

1. Deepening Mechanistic Scholarship: Addressing the core concern about LATP stability and the "Division of Labor" mechanism, we moved beyond qualitative inference. We have introduced 3D ToF-SIMS and XPS depth profiling to visualize the interfacial evolution. We now provide direct evidence that the TFPP-derived CEI effectively blocks transition metal crosstalk, and the in-situ formed polymer matrix physically isolates LATP from reduction by lithium metal. Furthermore, we substantiated these experimental findings with comprehensive multi-scale analyses: 1) **Theoretical Origins**: DFT and ESP calculations were incorporated to prove the thermodynamic selectivity and targeted sacrificial oxidation of TFPP. 2) **Bulk Transport**: MD simulations were employed to definitively prove that these additives strictly operate at the interfaces without perturbing the primary Li^+ solvation sheath.

2. Strengthening Logical Rigor: To address the concern regarding the "necessity" of our dual-additive strategy, we conducted a rigorous Ablation Control Study (comparing Single- vs. Dual-additive systems in cells). This new dataset (Supplementary Fig. 28-31) conclusively necessarily validates the synergistic effect: $Mg(TFSI)_2$ alone fails at high voltage, and TFPP alone cannot suppress long-term dendrites, while the Li-Mg alloy intermediates effectively boosts the ionic diffusivity across the Li metal interface. Meanwhile, the catalytic in-situ polymerization with the LATP reinforced PE guarantees the thermal and high-voltage stability. Only this scrutinized dual system and complementary strategy helps realize the safety protocol for the energy/power-dense prototype under the industrial standards, which is rarely reported in previous study.

3. Validating Practical Relevance: Based on reviewers' suggestions, we have supplemented substantial data of practical metrics and further illustrate the safety protocol of practical relevance, which includes: 1) A detailed **Mass Breakdown Table** (Supplementary Table 3) to strictly validate the 456.7 Wh kg^{-1} energy density. 2) **High-Voltage Floating Tests** (at 4.8 V) to replace ambiguous LSV scans, offering a definitive standard for oxidative stability. 3) **Ah-scale Safety Validation** under industry standards (GB 38031), proving the intrinsic safety of our design.

We believe these substantive upgrades of revision, in responses to reviewer's insightful comments, have transformed the manuscript from a promising report into a definitive, mechanism-guided framework for practical solid-state batteries. Below, we provide a detailed, itemized account of how we have addressed every single concern.

Point-by-point responses to reviewer's comments

Reviewer #1:

In this work, the authors fabricate an ultrathin (≈9.6 μm) PDOL-based solid polymer electrolyte (SPE) by inducing in situ ring-opening polymerization (ROP) of 1,3-dioxolane (DOL) within a micro-grooved scaffold consisting of polyethylene (PE) coated on both sides with LATP nanoparticles. In addition, a dual-additive approach (TFPP and $Mg(TFSI)_2$) is introduced to stabilize both the high-voltage NCM811 cathode and the Li metal anode interfaces. The demonstration of an Ah-scale (1.2 Ah) pouch-format cell together with industry-relevant safety tests (e.g., ARC and nail penetration) is application-oriented and potentially impactful. In particular, the reported combination of high ionic conductance (418.7 mS) and high tensile strength (191.7 MPa) in an ultrathin SPE addresses a key "performance–manufacturability–safety" triad that is of strong interest to top-tier journals.

However, several central claims namely "trade-off breaking," "dual interfacial stabilization," "transition-metal (TM) crossover suppression," and "intrinsic safety" are not yet fully supported at a Nature Communications standard. Mechanistic evidence remains largely qualitative, and critical practical cell metrics are insufficiently disclosed. In addition, the necessity and novelty of the selected additives (TFPP, $Mg(TFSI)_2$) are not convincingly established. A more rigorous ablation study, quantitative validation of TM dissolution/cross-talk suppression, and a dedicated assessment of LATP stability in a Li-metal environment are required.

Response: *We sincerely appreciate the reviewer for the comprehensive evaluation and for specifically recognizing the "performance–manufacturability–safety" triad achieved by our ultrathin SPE design. We are deeply encouraged by the reviewer's validation of our Ah-scale pouch cell demonstration and the acknowledgement that our work addresses key challenges in practical solid-state battery manufacturing. We remain convinced that realizing high ionic conductance (418.7 mS) and mechanical robustness (191.7 MPa) in a scalable thin-layer SPE membrane format represents a significant step toward bridging the gap between lab-scale materials and industrial application.*

Meanwhile, we fully accept the reviewer's critical observation that the central claims, specifically regarding "trade-off breaking", "dual interfacial stabilization" and "transition-metal (TM) crossover suppression", were not sufficiently supported by quantitative evidence in the original

submission. We agree that establishing the "necessity and novelty" of the dual-additive strategy requires a more rigorous logical connection between the chemical mechanism and the full-cell performance to meet the high standards of Nature Communications.

Guided by this rigorous assessment, we have treated this revision as a comprehensive overhaul of our evidence chain. We have moved beyond qualitative inference to establish a robust, quantitative framework. Specifically, we have implemented the following major upgrades: (1) Definitive Mechanistic Validation (LATP & TM Suppression): Addressing the concern about LATP stability and transition-metal (TM) crossover, we have introduced 3D ToF-SIMS reconstruction and XPS depth profiling (Supplementary Fig. 33-34 and Supplementary Fig. 48-50). These techniques visualize the spatial distribution of species, quantitatively proving that the in-situ polymerized matrix and dual additives effectively shield LATP from reduction and block TM migration at the source. (2) Rigorous Ablation Study: To conclusively establish the necessity of the chosen additives, we have added a systematic ablation study comparing single-additive systems (TFPP-only vs. Mg-only) against the dual-additive prototype. This new dataset confirms a synergistic "1+1>2" effect: $Mg(TFSI)_2$ is indispensable for anode dendrite suppression, while TFPP is critical for high-voltage cathode stability. (3) Included a dedicated post-mortem analysis to verify the stability of LATP within the polymer matrix. (4) Comparison of electrostatic potentials (ESP) and HOMO/LUMO energy levels for various cathode-side film-forming additives. Moreover, we added dynamic LSV scans with rigorous High-Voltage Floating Tests (at 4.8 V) to provide a definitive standard for oxidative stability.

We believe these substantial revisions have transformed the manuscript from a promising report into a definitive, mechanism-guided framework. Below, we provide a detailed, itemized account of how we have addressed every specific concern.

1. Insufficient validation of LATP stability against Li metal (chemical/structural)

LATP is widely recognized to exhibit reductive instability in contact with Li metal, potentially leading to Ti reduction, structural degradation, and continuous Li consumption via parasitic interfacial reactions. Although the authors position LATP as a “catalytic scaffold + mechanical reinforcement + ion-conduction pathway,” the long-term stability of the LATP-containing scaffold under cycling conditions constitutes a key risk for both durability and Coulombic efficiency.

Suggested revisions/experiments :

• Characterize the structural and chemical-state evolution of LATP before/after cycling (e.g., XRD or Raman; XPS Ti 2p for oxidation-state changes).

• Verify whether Ti-containing species appear at the Li-side interface after cycling (e.g., XPS depth analysis on the anode surface).

• Provide cross-sectional SEM/TEM evidence to assess cracking, delamination, and morphological collapse of the LATP-coated layer after prolonged cycling and/or under pressure conditions relevant to pouch-cell stacking.

Response: *We appreciate the reviewer raising this critical point regarding the thermodynamic instability of LATP against metallic Li. We fully acknowledge that bare LATP is highly susceptible to reductive degradation ($Ti^{4+} \rightarrow Ti^{3+}$) upon direct contact with lithium, which historically poses risks of structural collapse and continuous Li consumption.*

However, our specific architectural design fundamentally addresses this thermodynamic vulnerability through kinetic stabilization. As illustrated in the revised supporting information, the dense in-situ polymerized PDOL network, reinforced by the dual-additive-derived SEI, functions as a robust physical barrier that effectively isolates the LATP particles from direct contact with reactive Li metal. To rigorously validate this stabilization mechanism, we conducted a comprehensive post-mortem analysis on cycled NCM811||Li pouch cells, combining visual inspection, surface chemistry analysis (XPS), and 3D depth profiling (ToF-SIMS).

1. Mitigation of Surface Reduction

We first examined the macroscopic and chemical evolution of the LATP interface. As shown in Supplementary Fig. 47, we compared the pristine separator, the cycled PDOL-PE/LATP, and the cycled PDOL-PLMT. While the LATP layer in the PDOL-PE/LATP turned noticeably dark/black (indicating severe reduction) after contact with Li, the PDOL-PLMT sample exhibited only a slight color change, visually suggesting that the reductive reaction is effectively suppressed.

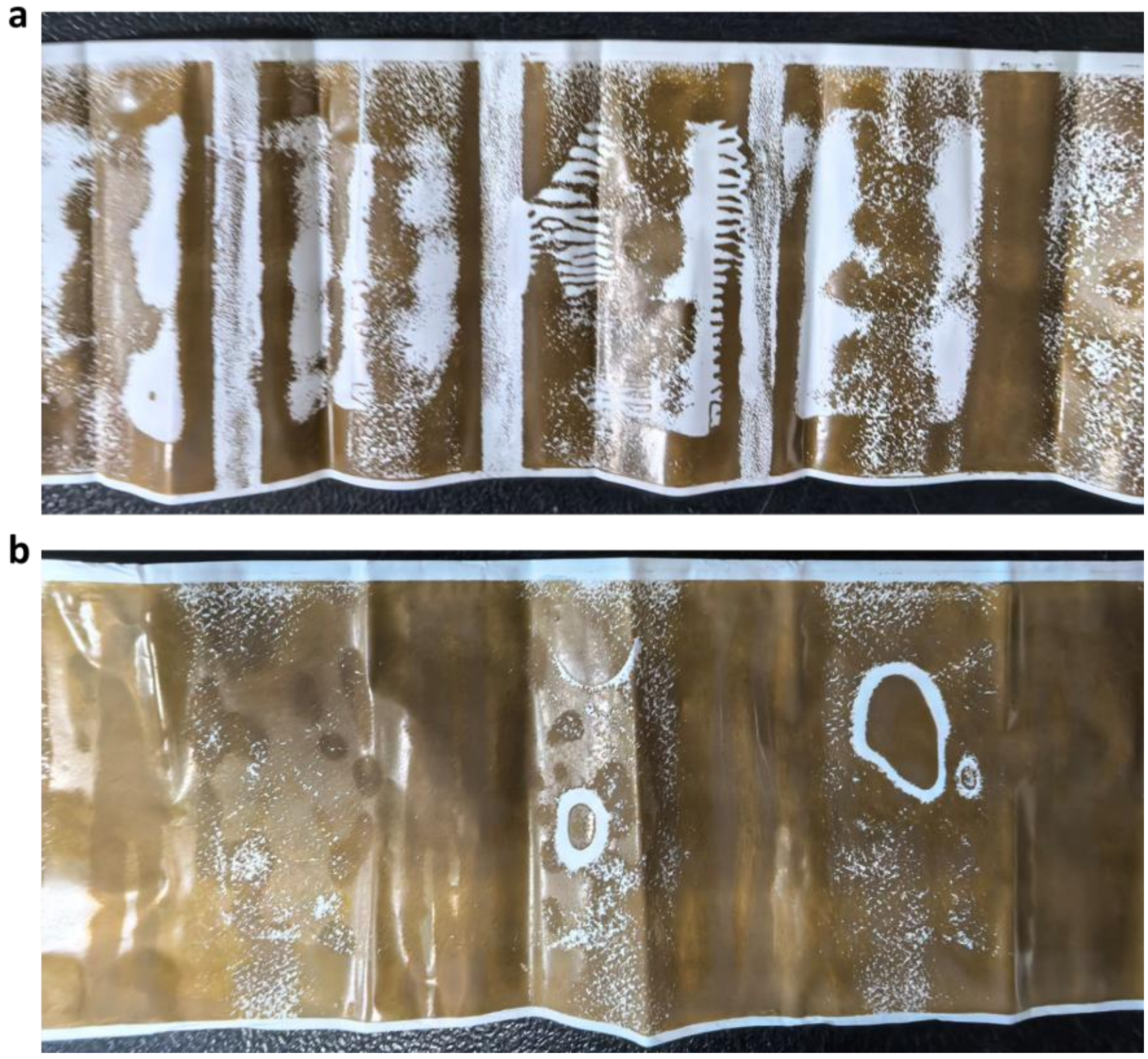

Supplementary Fig. 47 | Comparative visual analysis of the separators after 400 extended cycles. Optical images of the Li-facing side of separators harvested from (**a**) NCM811|PDOL-PLMT|Li and (**b**) NCM811 |PDOL-PE/LATP|Li pouch cells after 400 cycles.

To quantify this, we conducted high-resolution Ti 2p XPS analysis on the Li-facing separator surface (Supplementary Fig. 48). The pristine LATP shows only Ti^{4+} peaks. After cycling, the PDOL-PE/LATP sample shows a dominant Ti^{3+} signal, confirming substantial reduction (Supplementary Fig. 48c). In contrast, the PDOL-PLMT sample maintains only Ti^{4+} signal. This indicates that the specific electrolyte formulation and the dense PDOL polymer matrix effectively restrict the electron transfer to LATP.

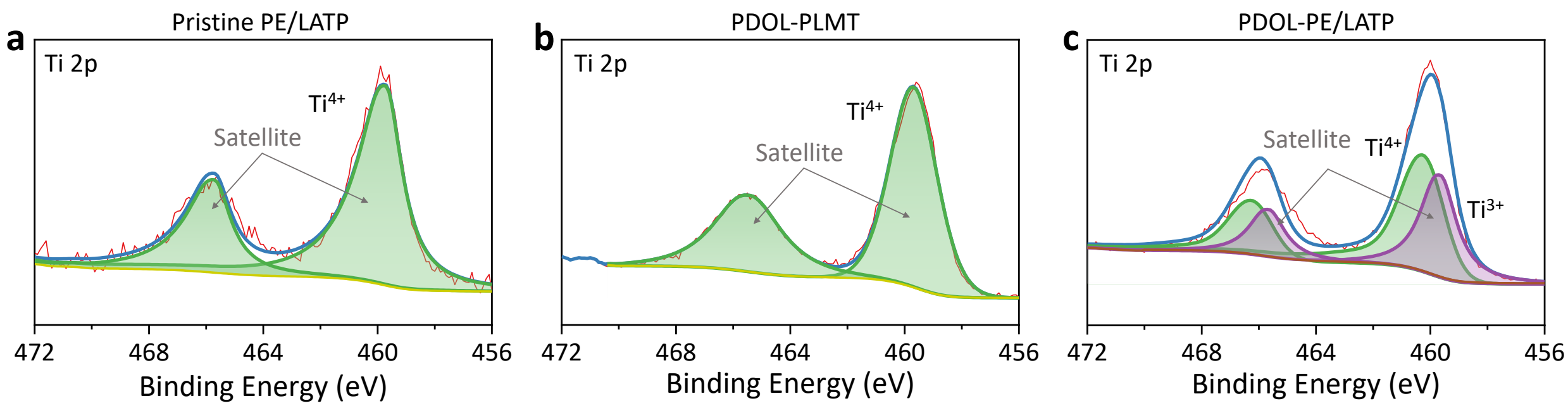

Supplementary Fig. 48 | XPS analysis of Ti valence states and interfacial reduction stability. Ti 2p XPS spectra of (**a**) the pristine PE/LATP separator surface, (**b**) the surfaces of PDOL-PLMT, and

(**c**) PDOL-PE/LATP harvested from the respective NCM811|Li pouch cells after cycling.

2. Suppression of Ti Cross-talk (XPS and ToF-SIMS Evidence)

Following your specific suggestion, we disassembled the cycled NCM811||Li full cells and conducted XPS analysis on the surfaces of the harvested Li metal anodes (specifically the side facing the PE/LATP separator). We systematically compared all four electrolyte configurations: the baseline PDOL-PE/LATP, the single-additive systems (TFPP-only and Mg-only), and the PDOL-PLMT. In the high-resolution Ti 2p spectra (Supplementary Fig. 49), no obvious characteristic peaks corresponding to Ti^{4+} or reduced Ti^{3+} species were detected on the Li anode surfaces across all four samples. The signals remained at the baseline noise level, which is below the XPS detection limit (~0.1 at%).

The absence of massive Ti crossover, even in the baseline PDOL-PE/LATP control, highlights a distinct structural advantage of our design. Unlike conventional blended composite electrolytes where ceramic particles are frequently exposed, our in-situ ring-opening polymerization of DOL conformally encapsulates the LATP nanoparticles. This dense PDOL polymer matrix acts as an effective physical buffer layer, preventing direct solid-solid contact between the highly reductive Li metal and the LATP fillers. Therefore, the XPS data confirms that gross structural degradation and severe Ti dissolution of LATP are successfully avoided in our specific architecture. The in-situ polymer coating natively protects the LATP, while the dual additives further secure the electrochemical stability of the interfaces.

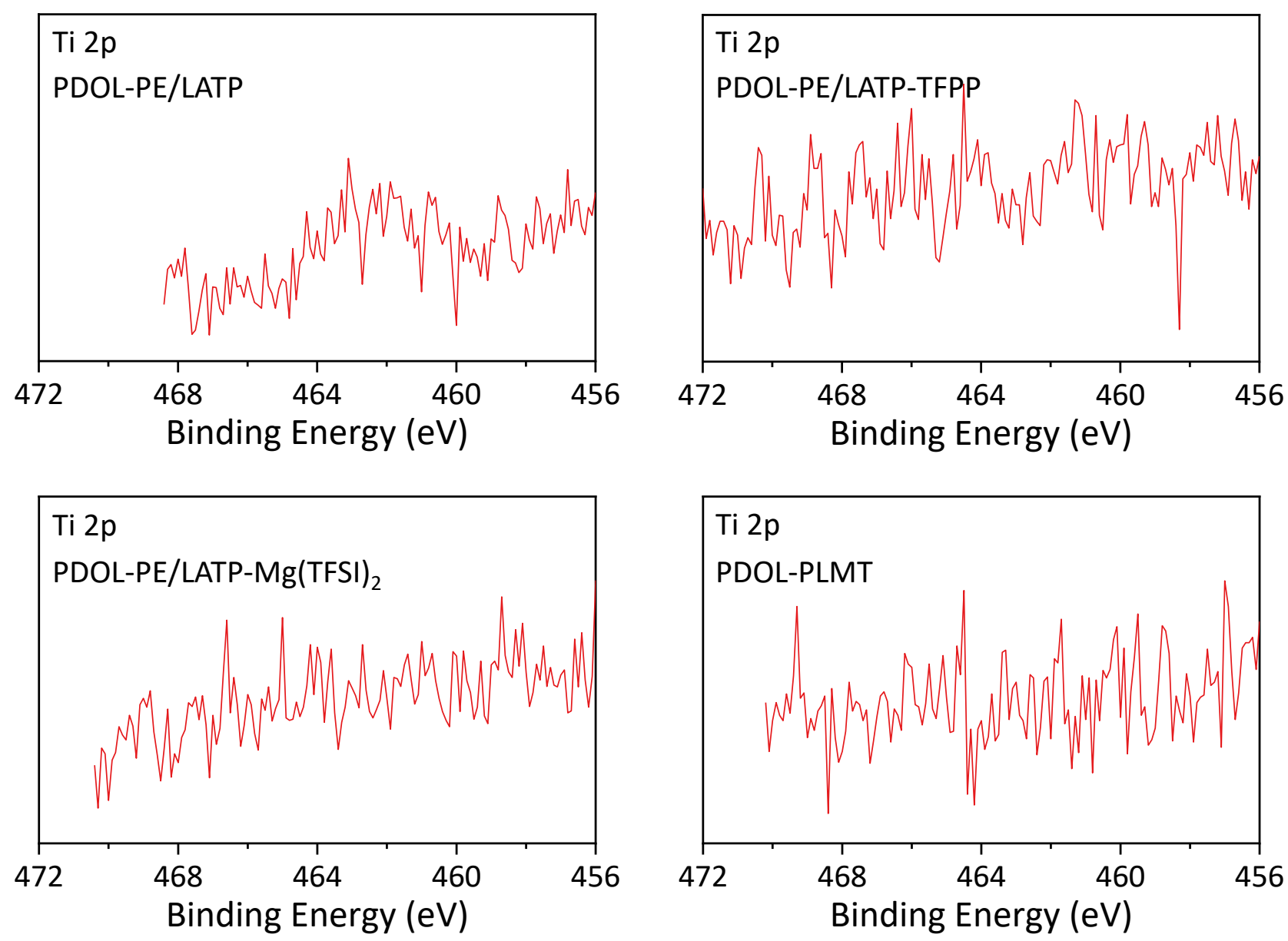

Supplementary Fig. 49| Surface chemical analysis of the Ti cation crossover to the Li metal anode. Ti 2p XPS spectra of the Li metal anode surfaces after cycling in NCM811|Li cells employing PDOL-PE/LATP, PDOL-PE/LATP-TFPP, PDOL-PE/LATP-Mg(TFSI)$_2$, and PDOL-PLMT.

While surface-sensitive XPS (Supplementary Fig. 49) showed no gross Ti accumulation above the ~0.1 at% detection limit for any sample (thanks to the conformal PDOL encapsulation), we went beyond the suggested XPS analysis and employed Time-of-Flight Secondary Ion Mass Spectrometry (ToF-SIMS). ToF-SIMS offers superior sensitivity for trace ion fragments and enables 3D visualization of their spatial distribution, allowing for a more rigorous assessment of LATP stability and Ti cross-talk. We disassembled the cycled cells and conducted depth profiling on the Li metal anodes from both the PDOL-PE/LATP and PDOL-PLMT systems. We tracked the characteristic Ti-containing fragment (TiO_2^-) on the cycled Li metal anodes Supplementary Fig. 50, to compare the intensity and distribution of the characteristic species on the anode:

For the PDOL-PLMT sample, the 3D reconstruction reveals a remarkably "clean" bulk (Supplementary Fig. 50b). The TiO_2^- signal is negligible and confined strictly to the outermost surface layer, rapidly disappearing upon sputtering. This indicates that the catalytic dual-additive strategy and the resulting SEI effectively isolates the LATP particles from the Li metal, preventing bulk reduction and minimizing Ti dissolution/migration. In stark contrast, the PDOL-PE/LATP exhibits a significantly higher intensity of TiO_2^- species (Supplementary Fig. 50c). Crucially, this signal persists deeper into the bulk of the SEI/Li interface before gradually tapering off. This confirms the reviewer's concern that without the protective additives, the bare LATP particles are susceptible to reductive attack by Li metal, leading to the generation and crossover of reduced Ti species.

This comparative depth profiling provides compelling evidence that in situ polymerized PDOL-PLMT matrix, reinforced by the Mg/TFPP-derived interfaces, effectively mitigates the chemical instability of LATP. The "parasitic Ti cross-talk" is successfully suppressed, ensuring the long-term durability of the scaffold."

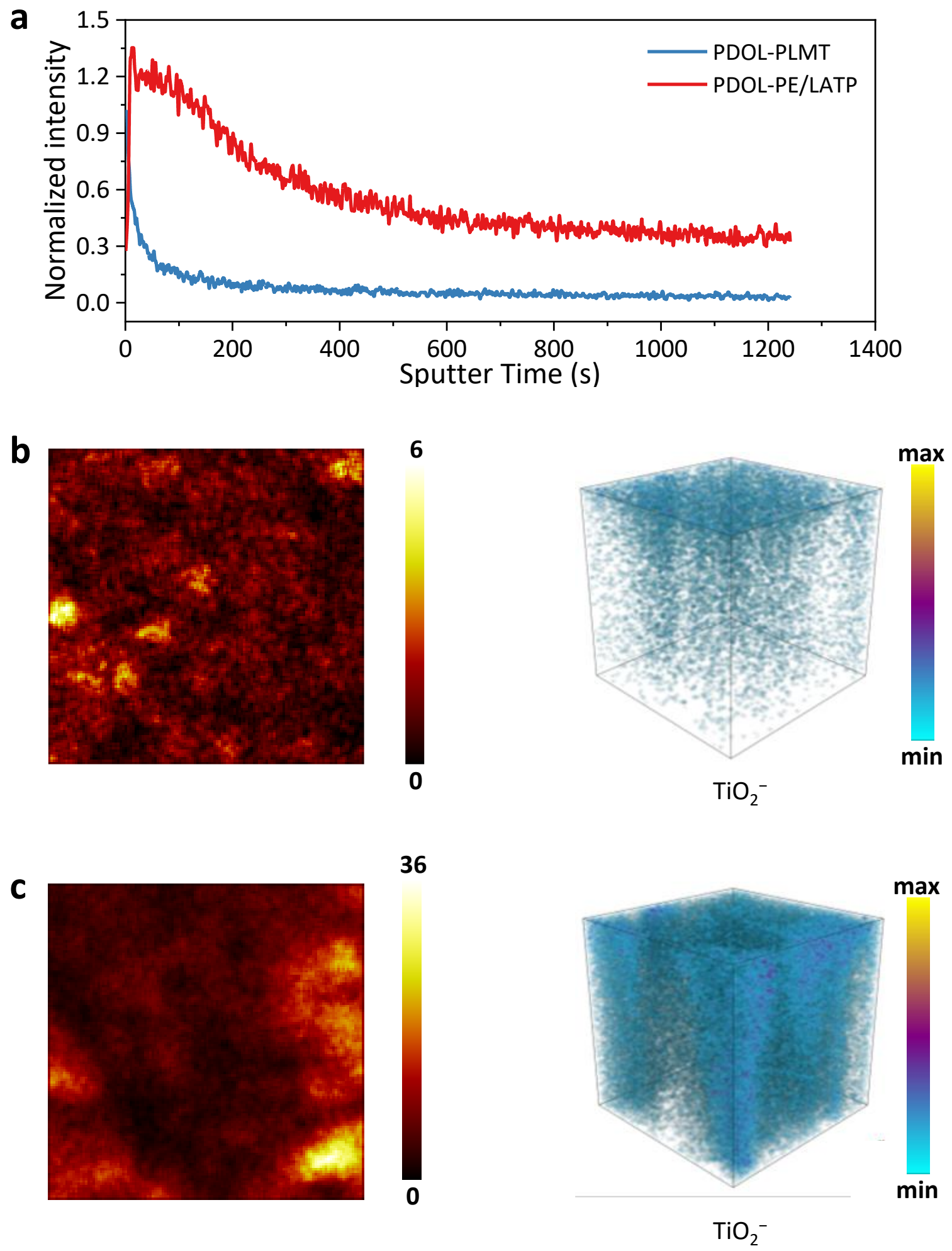

Supplementary Fig. 50 | 3D spatial distribution and confinement of Ti species. a Comparative depth profiles of TiO_2^- fragments on the Li metal anode surfaces harvested from NCM811|PDOL-PLMT|Li and NCM811|PDOL-PE/LATP|Li pouch cells after 400 cycles. 2D and 3D TOF-SIMS mapping images of the TiO_2^- species distribution in the (**b**) PDOL-PLMT and (**c**) PDOL-PE/LATP samples.

3. Structural Integrity (Cross-sectional SEM)

Finally, to address concerns regarding cracking or delamination, Supplementary Fig. 50 presents cross-sectional SEM images of the cycled PDOL-PLMT electrolyte. The images reveal that the reaction between LATP and Li is surface-limited. Crucially, the bulk structure of the LATP-coated layer remains intact without any signs of pulverization, delamination, or collapse. We attribute this structural stability to the in situ formed PDOL polymer matrix, which acts as a resilient binder to accommodate the volume changes and preserve the integrity of the coating layer.

Although a minor interfacial reaction occurs, our long-term cycling data demonstrates that it

does not lead to continuous capacity degradation or "short-circuiting" behavior. The reaction products likely form a self-passivating mixed-conducting interphase (MCI) that, in conjunction with our additive-derived SEI, prevents further parasitic consumption of active lithium. Therefore, the Coulombic efficiency remains stable, and the capacity loss attributable to LATP reduction is negligible in this optimized system. These data and related discussions are now presented in the supplementary information as Supplementary Fig. 51.

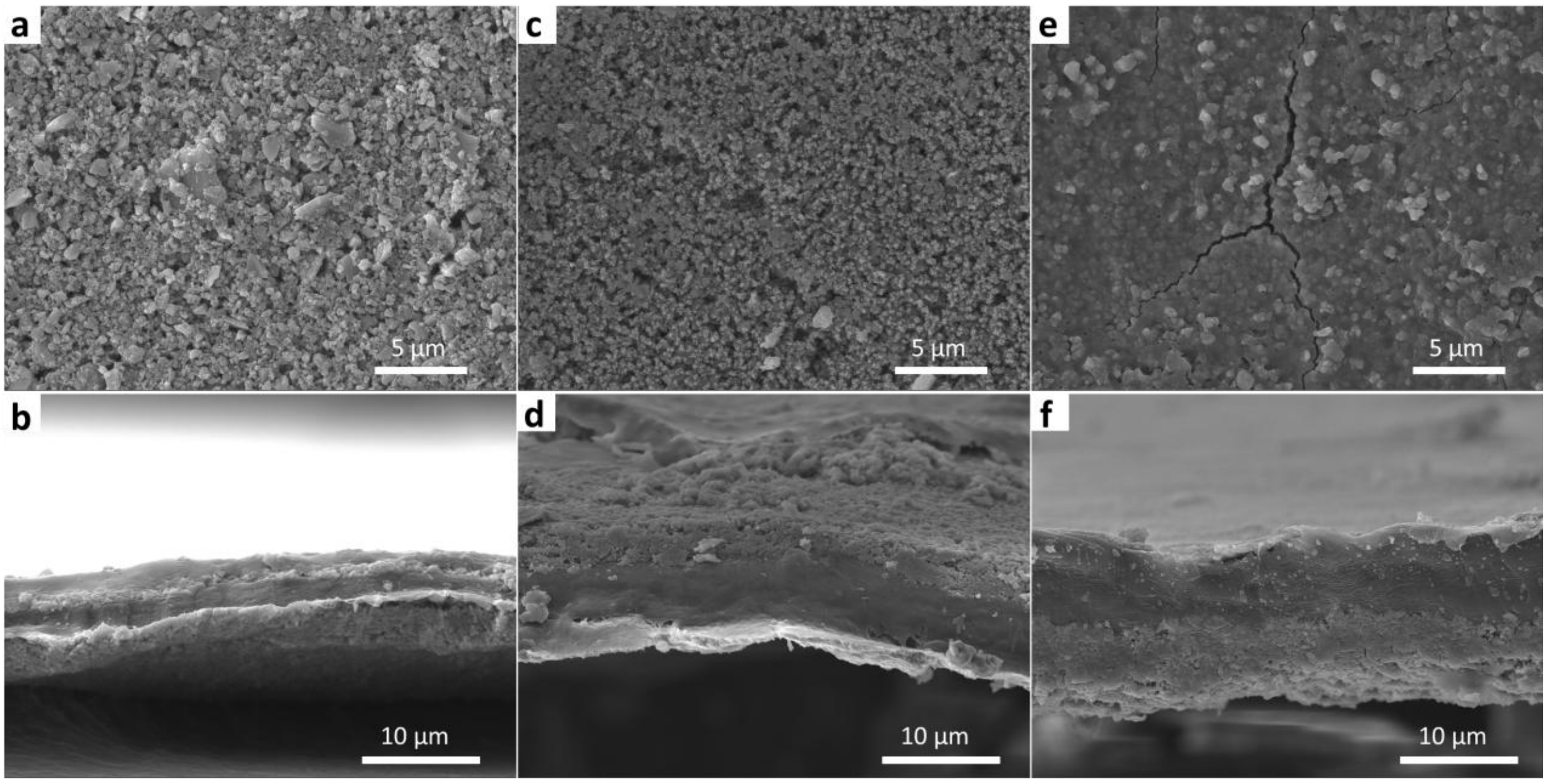

Supplementary Fig. 51 | Microstructural stability and morphological evolution of the composite separators. a Surface and (**b**) cross-sectional SEM images of the pristine PE/LATP separator. SEM images showing the (**c**) surface and (**d**) cross-section of the PE/LATP separator retrieved from the NCM811|PDOL-PLMT|Li pouch cell after cycling. **e** Surface and (**f**) cross-sectional SEM images of the PE/LATP separator retrieved from the NCM811|PDOL-PE/LATP|Li pouch cell after cycling.

2. Apparent inconsistency between the proposed CEI-centered role of TFPP and its expected reactivity toward Li metal (SEI contribution)

From an electronic-structure perspective (HOMO/LUMO), TFPP appears likely to undergo reduction at the Li metal interface and thus may substantially influence SEI chemistry. Prior literature also reports TFPP-like additives altering SEI composition (often toward Li_2CO_3/LiF-rich SEI). In contrast, the present manuscript primarily attributes TFPP function to cathode-side CEI formation and TM crossover suppression. If anode-side XPS indicates reduced LiF and increased $ROCO_2Li$-like species,

this may contradict the intuitive expectation that fluorinated motifs would promote LiF enrichment, and it raises questions about TFPP's dominant reaction pathway in this system

Moreover, if TFPP's HOMO level is not significantly higher than alternative high-voltage cathode additives, the rationale for selecting TFPP as a cathode-focused additive is not sufficiently justified. Without a clearer mechanistic and comparative justification, TFPP may be perceived mainly as an anode-reactive additive, weakening the manuscript's claim of TFPP-driven CEI stabilization and cathode protection.

Suggested revisions/experiments :

- Provide a direct, systematic assessment of TFPP's impact on SEI composition and evolution, including quantitative/semi-quantitative comparisons after formation and after long-term cycling.
- If TFPP is claimed to be critical for CEI and TM control, the manuscript must include quantitative evidence of TM dissolution/cross-talk suppression.
- Strengthen the justification of TFPP as a cathode additive by comparing against (or at minimum discussing) benchmark high-HOMO cathode additives, and clarifying why TFPP is uniquely effective under the proposed polymer-electrolyte chemistry.

Response: *We appreciate the reviewer's keen insight regarding the dual reactivity of TFPP. We agree that from a HOMO/LUMO perspective, TFPP possesses a reduction potential compatible with Li metal. However, our experimental data reveals that in this specific "dual-additive" system, the roles are functionally partitioned: $Mg(TFSI)_2$ dominates the anodic SEI formation, while TFPP plays a decisive role in cathodic CEI stabilization and TM crossover suppression. To address the reviewer's concerns, we have performed a systematic XPS/TOF-SIMS analysis and a quantitative ICP-OES assessment, alongside a theoretical justification for TFPP selection.*

1. Validation of TFPP Function (XPS and ICP-MS Evidence)

We compared the high-resolution F 1s XPS spectra of Li anodes cycled in PDOL-PE/LATP-$Mg(TFSI)_2$, PDOL-PE/LATP-TFPP and PDOL-PLMT electrolytes (Supplementary Fig. 27). Both systems exhibit comparable, high intensities of LiF signals. This indicates that the LiF-rich SEI is primarily derived from the decomposition of the $TFSI^-$ anion and the Mg salt, rather than TFPP. This allows us to attribute the unique performance improvements (specifically high-voltage stability) to TFPP's function at the cathode interface.

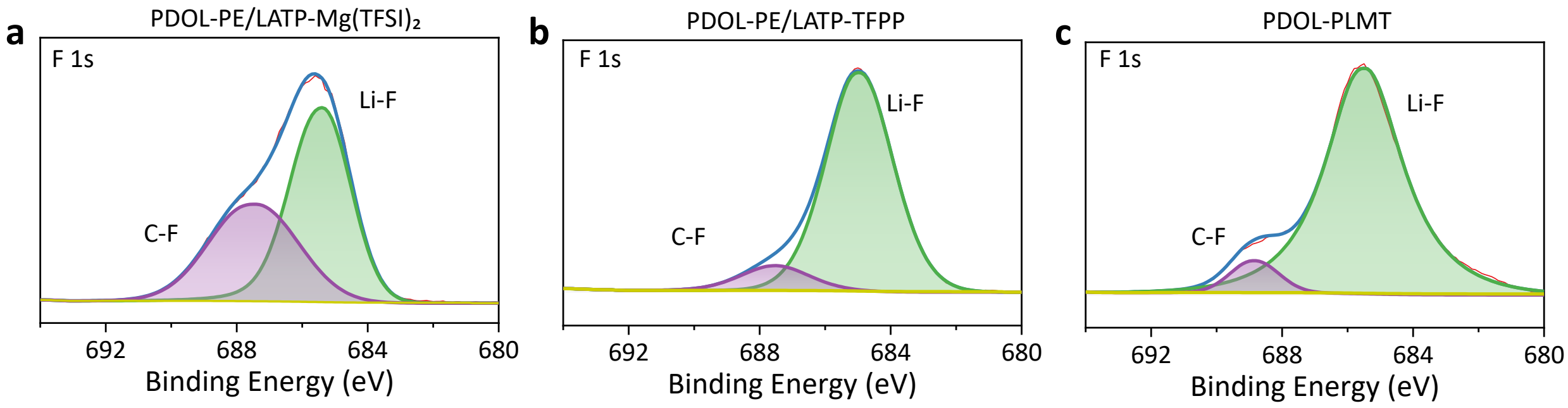

Supplementary Fig. 27 | Surface chemistry and SEI composition analysis on the Li metal anodes. High-resolution F 1s XPS spectra of the Li metal anode surfaces harvested from NCM811|Li coin cells after 100 cycles, employing (**a**) PDOL-PE/LATP-$Mg(TFSI)_2$, (**b**) PDOL-PE/LATP-TFPP, and (**c**) PDOL-PLMT.

To validate the claim that TFPP functions primarily to stabilize the cathode and suppress Transition Metal (TM) dissolution, we conducted ICP analysis on the Li metal anodes harvested from cycled cells (Supplementary Fig. 32). The inclusion of TFPP (in both the TFPP-only and full PLMT systems) reduces the total TM deposition on the anode by ~66% compared to the control. This provides direct quantitative evidence that TFPP preferentially oxidizes to form a robust, protective CEI on the NCM811 surface, effectively blocking TM dissolution.

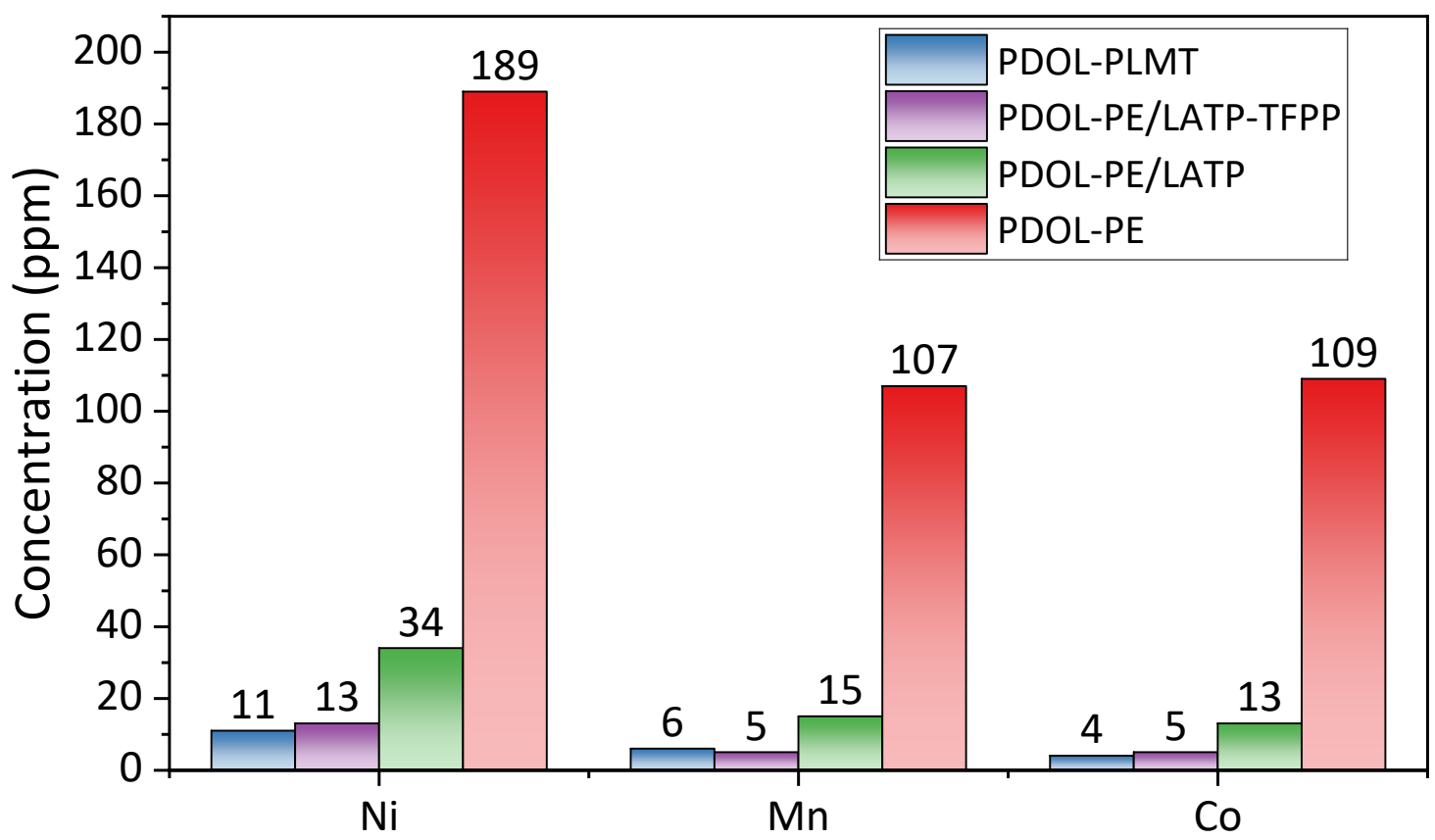

Supplementary Fig. 32 | Quantitative analysis of TM dissolution and migration. Ni, Co, and Mn contents detected on Li metal anodes harvested from cycled NCM811|Li cells assembled with four different electrolytes.

2. Thermodynamic Rationale for Priority Oxidation

We respectfully clarify that the unique efficacy of TFPP in our specific PDOL-PLMT system stems from a delicately balanced thermodynamic design. To rigorously justify the selection of TFPP as a cathode-focused additive, we have expanded our Density Functional Theory (DFT) calculations

to compare TFPP (Tris(4-fluorophenyl)phosphine) against a series of benchmark organophosphorus/fluorinated additives (TPFPP (Tris(pentafluorophenyl)phosphine), TPP (Triphenylphosphine), TPPa (Triphenyl Phosphate), TTFP (Tris(2,2,2-trifluoroethyl) phosphite) as well as the baseline electrolyte components (DOL, PDOL, LiTFSI).

To effectively protect the polymer matrix, a cathode additive must possess a HOMO level higher than that of the matrix, ensuring it undergoes preferential, sacrificial oxidation. The polymer backbone (PDOL, -6.711 eV) and salt (LiTFSI, -6.613 eV) establish the baseline oxidative stability. Highly fluorinated benchmarks like TPFPP (-7.475 eV) and TTFP (-7.809 eV) exhibit HOMOs that are too deep (Supplementary Fig. 9). They are overly stable against oxidation and will fail to sacrificially protect the PDOL matrix at high voltages, allowing the polymer to decompose first. Conversely, TPP (-6.013 eV) oxidizes too easily. While it reacts early, it lacks fluorine, resulting in a purely organic/phosphorus-rich CEI that is structurally weak and unable to block TM crossover at 4.5 V. TFPP (-6.297 eV) strikes the perfect thermodynamic balance. Its HOMO is sufficiently higher than PDOL/LiTFSI to guarantee priority oxidation, yet its molecular structure contains critical perfluoroalkyl groups (unlike TPP) to construct a robust, high-voltage resistant, and F-rich CEI.

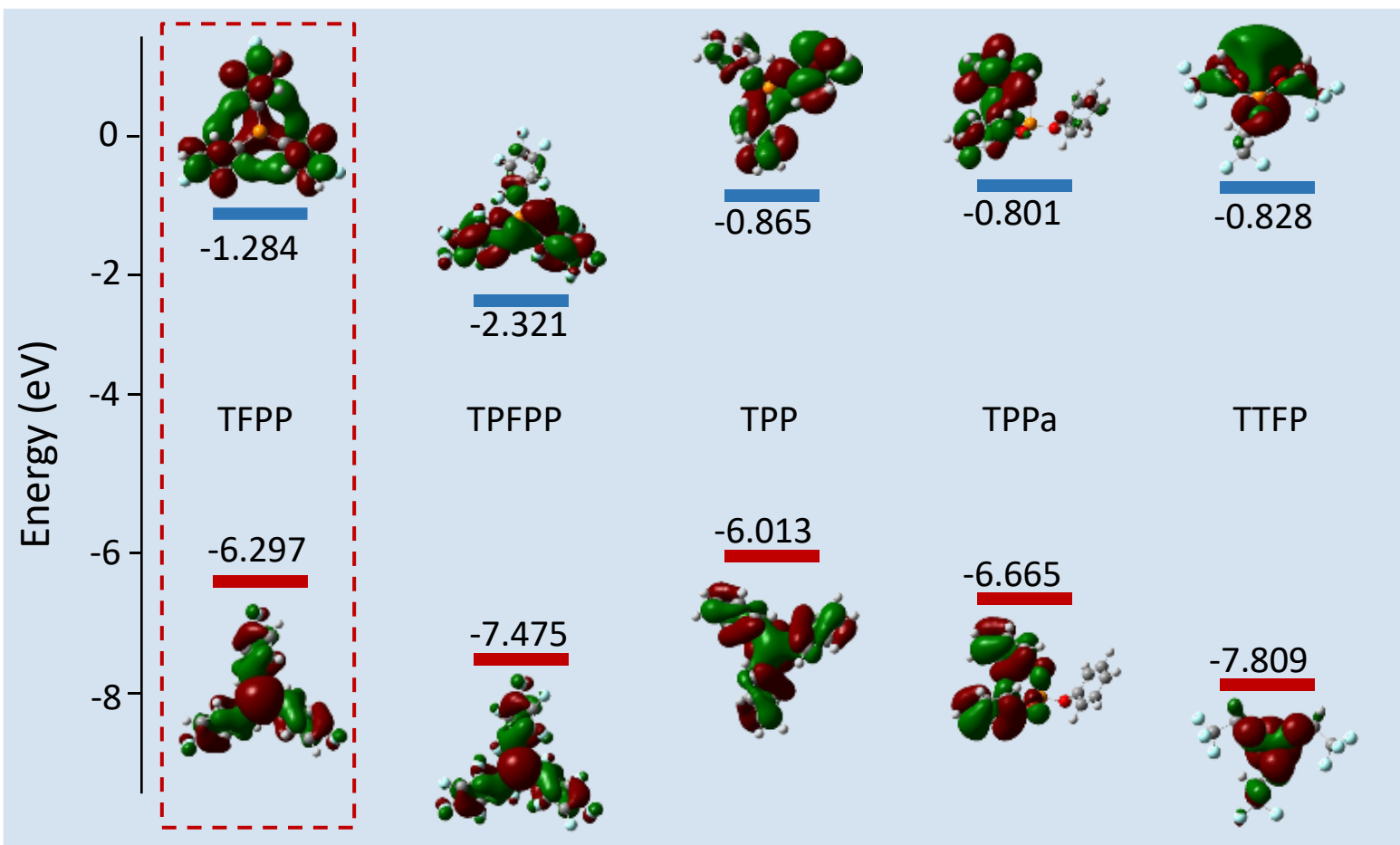

Supplementary Fig. 9 | Frontier molecular orbital energies of the investigated additives. Comparison of the HOMO and LUMO energy levels for the organophosphorus and fluorinated electrolyte additives.

3. Site-Specific Reactivity via ESP Mapping

To provide decisive spatial evidence on how these additives prioritize reaction at the high-voltage cathode, we calculated and analyzed the Electrostatic Potential (ESP) mapped surfaces of all five candidates, presented in Supplementary Fig. 10. ESP maps visualize the electrophilic (blue)

and nucleophilic (red) regions of a molecule, thereby pinpointing the precise sites of initial reaction.

Our head-to-head comparison of TFPP against the benchmarks reveals unique structural and electronic advantages that justify its selection: The ESP map of TFPP unambiguously reveals concentrated electron-rich regions (indicated by red hot-spots) precisely located on the central phosphorus atom and specific edges of the phenyl rings. This indicates a high propensity for preferential oxidation (nucleophilic attack from the high-voltage cathode). Crucially, the para-fluorine atoms show neutral-to-electron-poor character (green-to-blue), ensuring they are durable against initial oxidation. This spatial configuration guarantees that TFPP not only reacts first (high HOMO) but does so at targeted sites, preserving the robust fluorinated motifs to build a sturdy, inorganic-rich (LiF-containing) CEI.

Non-fluorinated TPP and TPPa show diffuse red regions all over their organic backbones (indicating low electron density polarization). While TPP oxidizes easily (highest HOMO), its ESP map shows no chemically focused sites to create robust inorganic barrier. Its decomposition is more likely to yield purely organic polymers or loose organic species (implied by the generalized red areas on phenyl rings), which are structurally weak and fail to suppress deleterious TM crossover. Contrast with Overly Inert TPFPP/TTFP: Highly fluorinated TPFPP and TTFP are heavily electron-deficient, indicated by their predominantly green/blue surfaces (highly positive ESP scales). TPFPP is highly polarized; while it has a red peak on Phosphorus, the overall molecule is inert. TTFP, with no distinct red regions, is too nucleophilicly stable. As confirmed by their deep HOMOs, these molecules cannot sacrificially protect the PDOL matrix because they will not react before the polymer backbone is attacked by high-voltage stress.

Together, the HOMO/LUMO energetics and the ESP-mapped spatial reactivity confirm that TFPP is uniquely optimized for this PDOL matrix. It is the only additive among the benchmarks that thermodynamic balance (-6.297 eV) to guarantee sacrifice-first protection combined with a specific molecular ESP signature that targets focused reaction sites on the P-center while preserving the F-groups to form a durable, TM-blocking CEI.

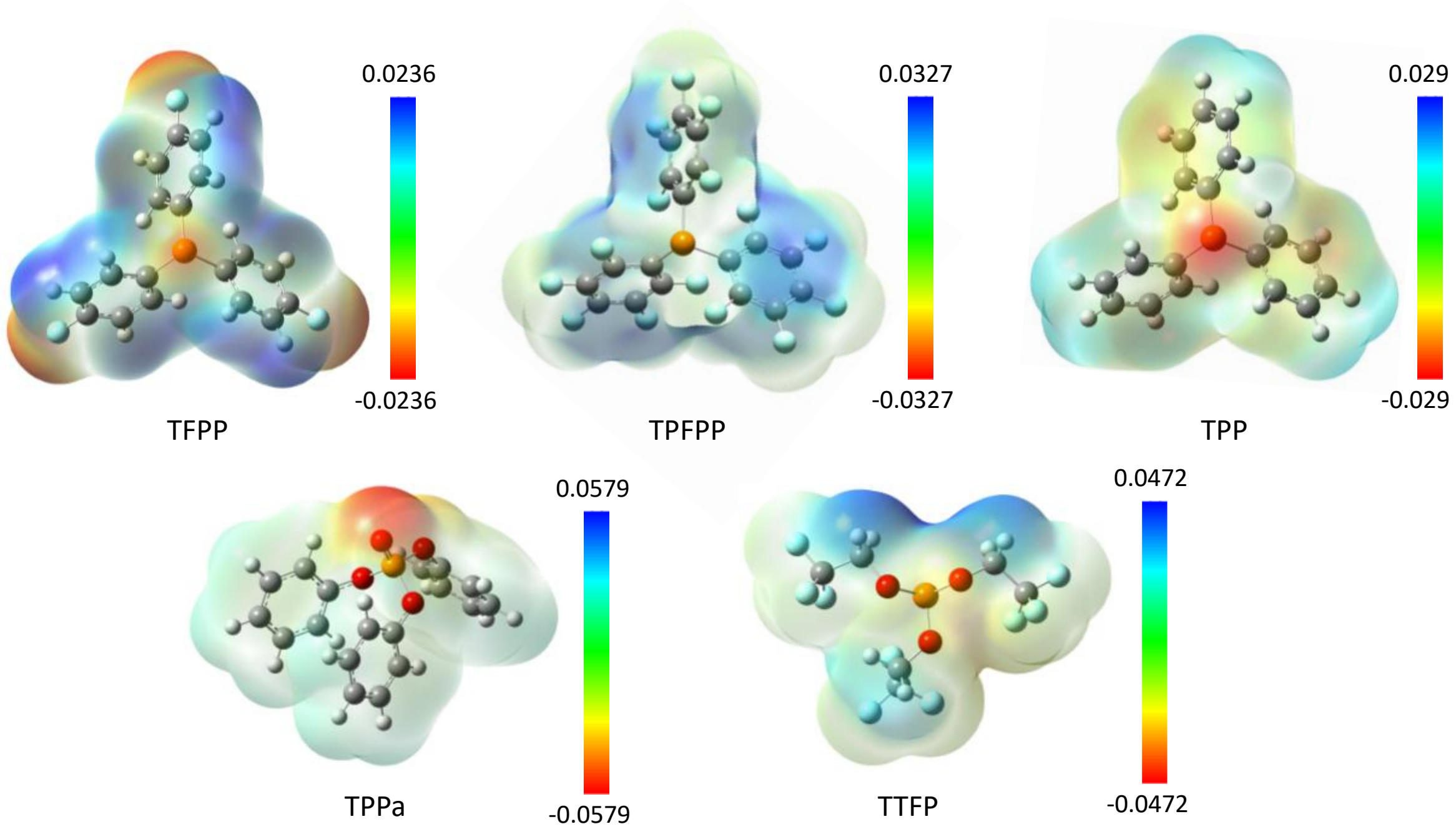

Supplementary Fig. 10 | Molecular charge distribution and identification of active interaction sites. Electrostatic potential (ESP) maps for the investigated additive molecules: TFPP, TPFPP, TPP, TPPa, and TTFP.

We have incorporated these discussions and the corresponding figures into the revised supplementary information (Supplementary Fig. 10, the highlighted part).

3. Lack of an ablation study to disentangle the "dual additive" contributions

The current comparison appears centered on PE vs LATP vs PLMT. Under such a design, it is difficult to isolate the respective contributions of TFPP and $Mg(TFSI)_2$. This is a critical weakness because the manuscript's main narrative relies on the claim that the dual-additive strategy simultaneously stabilizes both interfaces.

Suggested revisions/experiments:

• Include at least two additional control groups: LATP + TFPP (without $Mg(TFSI)_2$) and LATP + $Mg(TFSI)_2$ (without TFPP), evaluated in full cells (and ideally pouch cells) with cycling and rate performance.

• Provide corresponding interfacial characterization (CEI/SEI) for each ablation condition to support the proposed division of labor (TFPP → CEI; $Mg(TFSI)_2$ → Li–Mg interphase).

Response: *We appreciate the reviewer's request to clarify the individual contributions of TFPP and*

$Mg(TFSI)_2$. To demonstrate their specific roles, we performed comparative studies on single-additive electrolytes.

1. Decoupling the Interfacial Functions of TFPP and $Mg(TFSI)_2$

Our Li symmetric cell tests confirm that the inclusion of $Mg(TFSI)_2$ is critical for preventing dendrite-induced failure. While the PDOL-PE/LATP-TFPP sample succumbed to soft short-circuits within 500 hours, the PDOL-PE/LATP-$Mg(TFSI)_2$ maintained stable cycling for over 800 hours, verifying that the in situ formed Li-Mg alloy interface is the dominant factor in regulating Li deposition.

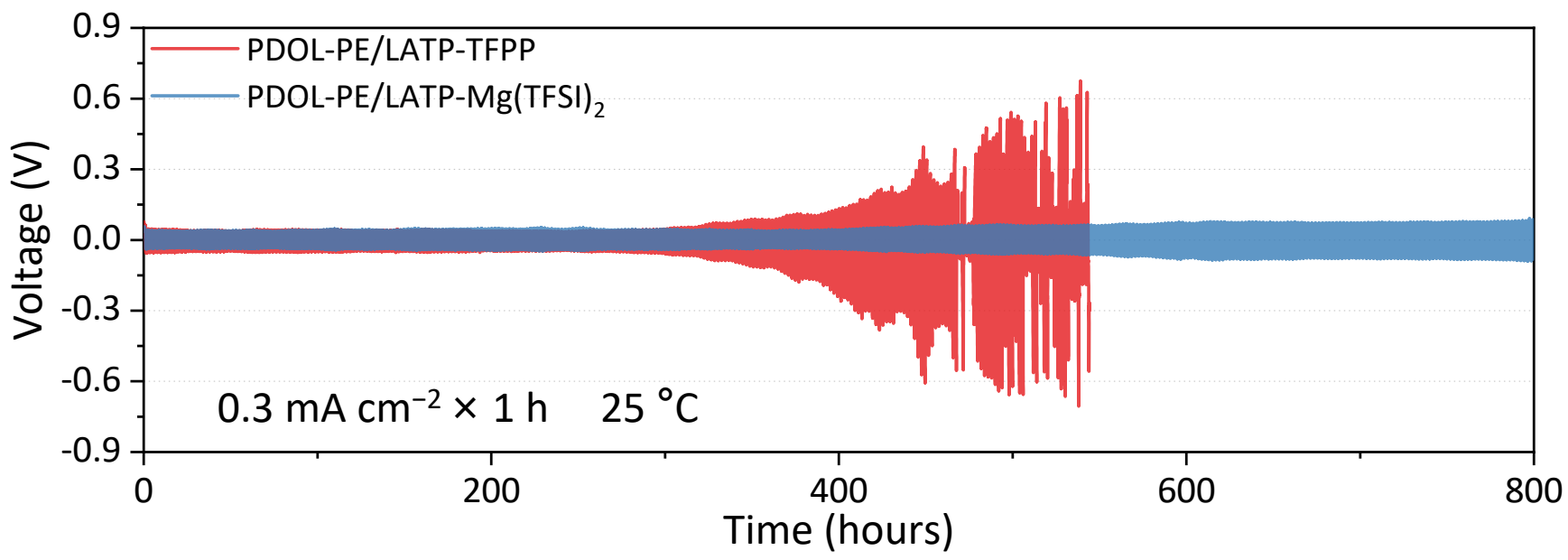

Supplementary Fig. 28 | Long-term interfacial stability and lithium cycling endurance. Galvanostatic Li plating/stripping profiles of the Li|Li symmetric cells employing the PDOL-PE/LATP-TFPP and PDOL-PE/LATP-$Mg(TFSI)_2$ electrolytes.

Conversely, full-cell testing highlights TFPP as the primary stabilizer for the cathode. Under aggressive 4.5 V cycling (Supplementary Fig. 29), the PDOL-PE/LATP-$Mg(TFSI)_2$ system suffered severe degradation (71.1% retention after 100 cycles). In sharp contrast, the PDOL-PE/LATP-TFPP system retained 86.2% of its capacity, nearly matching the dual-additive system. This proves that the TFPP-derived CEI is indispensable for preserving NCM811 structural integrity at high voltages. The 0.5C cycling (Supplementary Fig. 30) and rate capability tests (Supplementary Fig. 31) provide further insight: while $Mg(TFSI)_2$ enhances high-rate kinetics (due to the fast-ion conducting SEI), TFPP ensures superior capacity recovery after stress.

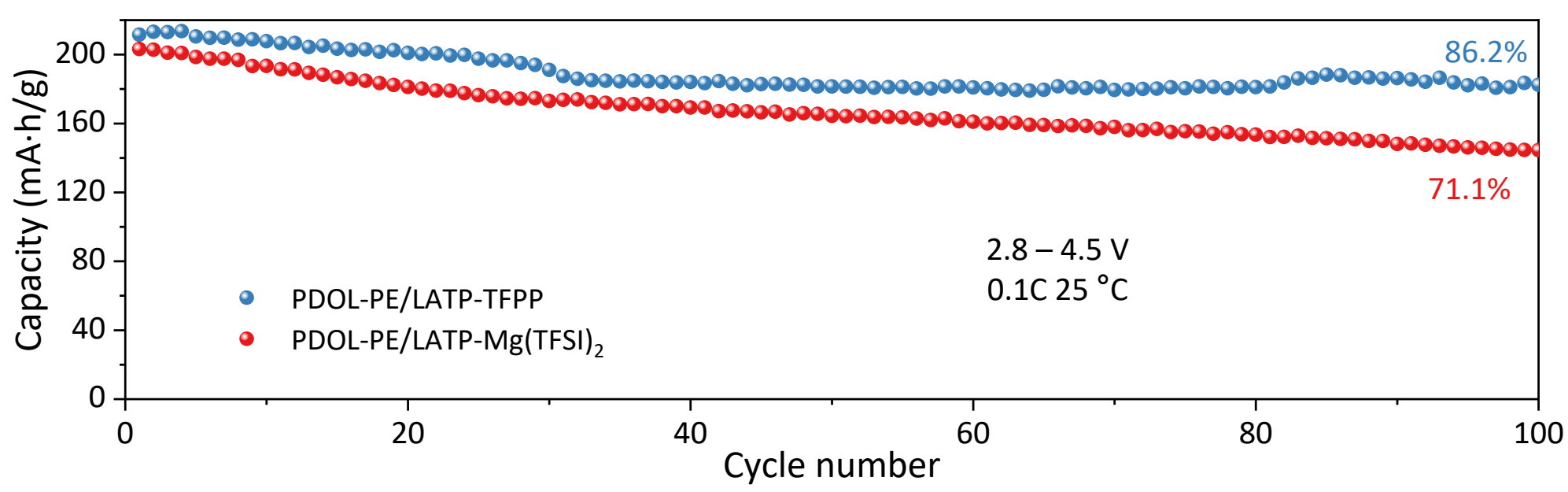

Supplementary Fig. 29 | High-voltage cycling endurance and capacity retention. Long-term cycling performance of NCM811|Li cells using PDOL-PE/LATP-TFPP and PDOL-PE/LATP-$Mg(TFSI)_2$ electrolytes at 4.5V.

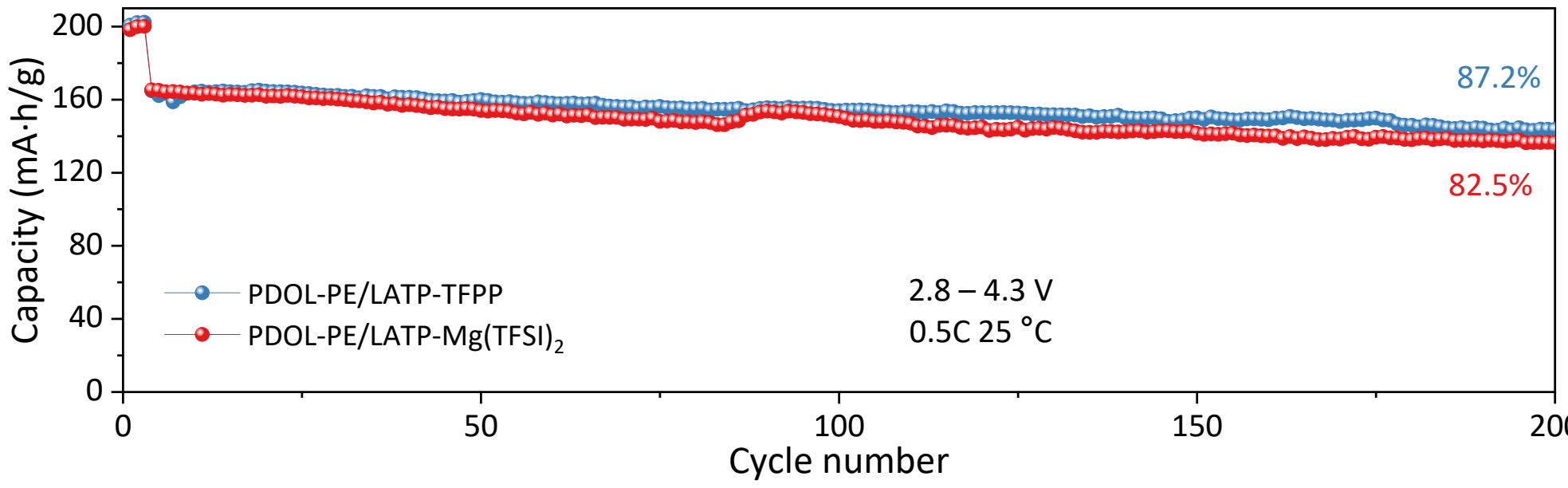

Supplementary Fig. 30 | Comparative evaluation of the long-term cycling endurance. Long-term cycling performance of NCM811|Li coin-cells using PDOL-PE/LATP-TFPP and PDOL-PE/LATP-$Mg(TFSI)_2$ electrolytes at 0.5C.

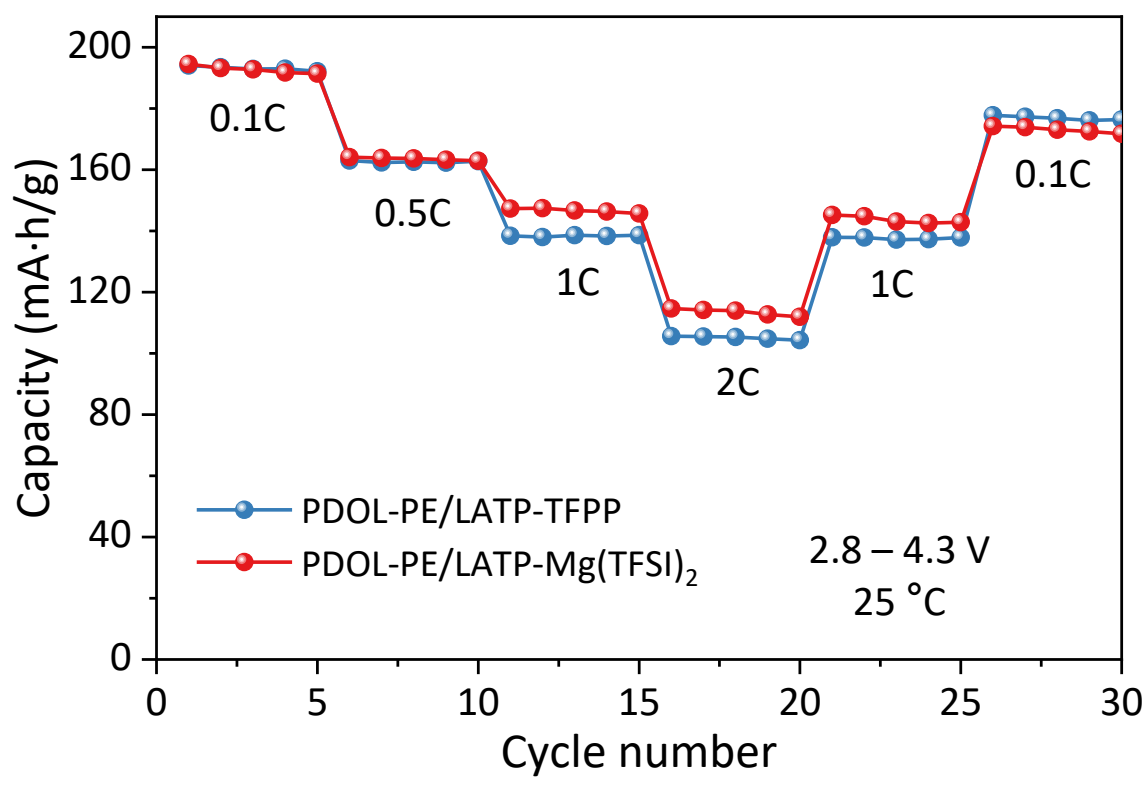

Supplementary Fig. 31 | Rate capability and electrochemical kinetic response at 25 °C. Comparative rate performance of the NCM811|Li cells employing PDOL-PE/LATP-TFPP and PDOL-PE/LATP-$Mg(TFSI)_2$ electrolytes from 0.1C to 2C.

By comparing these single-additive baselines, we conclusively demonstrate a synergistic "1+1>2" effect: the dual-additive PDOL-PLMT system combines the kinetic and anode-stabilizing benefits of Mg with the high-voltage cathode protection of TFPP, achieving performance levels that neither component can sustain individually. We have included these corresponding figures in the Supplementary Information.

2. Clarification on the Interfacial Characterization (CEI/SEI)

We sincerely thank the reviewer for this constructive suggestion regarding the verification of the "division of labor" mechanism. We agree that direct interfacial characterization of the ablation conditions (single-component systems) is crucial to support our mechanistic claims. We respectfully

wish to highlight that Fig. 3 and Fig. 4 in the original manuscript were specifically designed and structured to isolate these distinct interfacial roles, acting as the characterization counterpart to the electrochemical ablation studies.

As illustrated in Fig. 3a, our density functional theory (DFT) calculations compared the frontier molecular orbital energies of all electrolyte components. The results indicate that TFPP possesses the highest occupied molecular orbital (HOMO) energy level, suggesting it is thermodynamically favored to undergo sacrificial oxidation prior to the DOL monomer or other solvent molecules. This theoretical prediction is experimentally corroborated in Fig. 3b-c, where the electrochemical profiles confirm the specific film-forming potential of TFPP. The distinct oxidation peak observed confirms the preferential decomposition of TFPP to initiate CEI formation.

Crucially, the chemical and physical nature of the derived interphase is detailed in Fig. 3d-g: The spectra reveal that the CEI formed in the presence of TFPP is rich in specific LiF and C-F inorganic species (derived from TFPP), distinct from the organic-dominated layer in control samples. Transmission Electron Microscopy (TEM) further visualizes this CEI as a thin, dense, and conformal passivation layer on the cathode surface. Finally, Fig. 3h-k demonstrates the electrochemical impact of this modified interface. The inclusion of TFPP significantly enhances the high-voltage tolerance of the SPE, confirming that the TFPP-derived CEI effectively suppresses parasitic electrolyte decomposition.

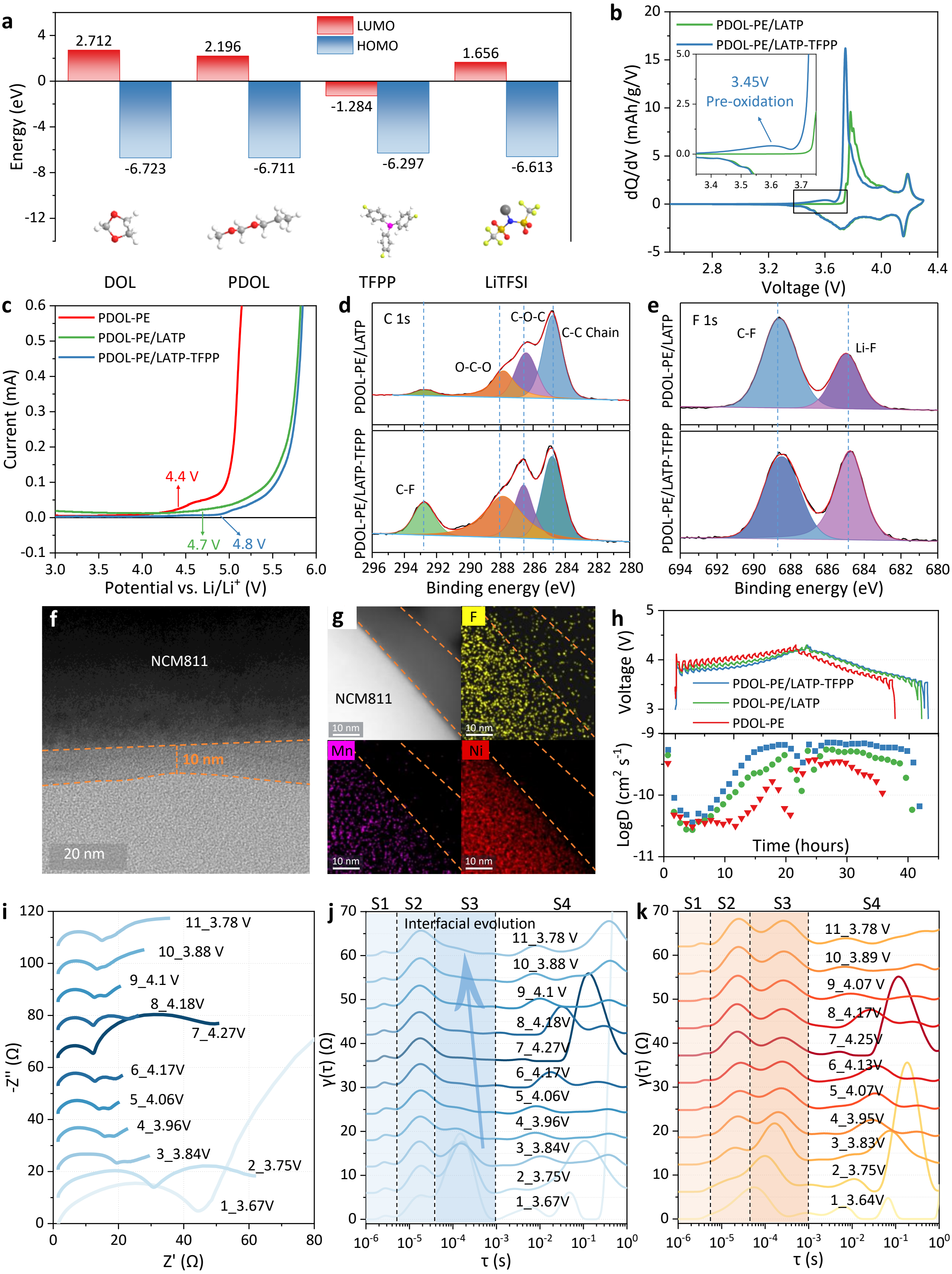

Fig. 3 | Characterization of the TFPP-derived CEI and its effect on NCM811 interfacial stability.

a DFT-calculated HOMO/LUMO energies of DOL, PDOL, TFPP and LiTFSI salt. **b** dQ/dV curves

of NCM811| PDOL-PE/LATP |Li and NCM811| PDOL-PE/LATP-TFPP |Li cells during the initial cycle. **c** LSV curves of PDOL, PDOL-PE/LATP and PDOL-PE/LATP-TFPP SPEs after the formation cycle. **d**, **e** C 1s and F 1s core-level XPS spectra of NCM811 cathodes after cycling with the PDOL-PE/LATP and PDOL-PE/LATP-TFPP SPEs. **f**, **g** TEM image, high-angle annular dark-field image and F, Mn, Ni elemental mappings of a cycled NCM811 cathode particle in the NCM811| PDOL-PE/LATP-TFPP |Li cell. **h** GITT plots and corresponding diffusion coefficients of NCM811|Li cells as paired with PDOL, PDOL-PE/LATP, and PDOL-PE/LATP-TFPP. **i** Galvanostatic electrochemical impedance spectra (GEIS) collected during initial charge/discharge of the NCM811| PDOL-PE/LATP-TFPP |Li cell, and (**j**) corresponding DRT analysis. **k** DRT analysis derived from the initial charge/discharge GEIS data of the NCM811| PDOL-PE/LATP |Li cell.

Similarly, Fig. 4 is dedicated to characterizing the anode interface to prove the role of the Mg additive. The operando XRD, ToF-SIMS depth profiling and XPS in Fig. 4a-f clearly identify the presence of a distinct Li-Mg alloy signal at the anode surface only in the Mg-containing systems. This kinetic benefit is quantitatively confirmed in Fig. 4g. The Mg-containing system exhibits a markedly higher exchange current density (j_0), indicating faster and more reversible interfacial charge transfer kinetics. As calculated in Fig. 4h, the introduction of the Li-Mg alloy phase significantly lowers the nucleation and diffusion energy barriers for lithium atoms on the anode surface compared to bare lithium. This thermodynamic advantage promotes the lateral migration of Li adatoms, effectively preventing the localized charge accumulation that typically triggers dendrite growth.

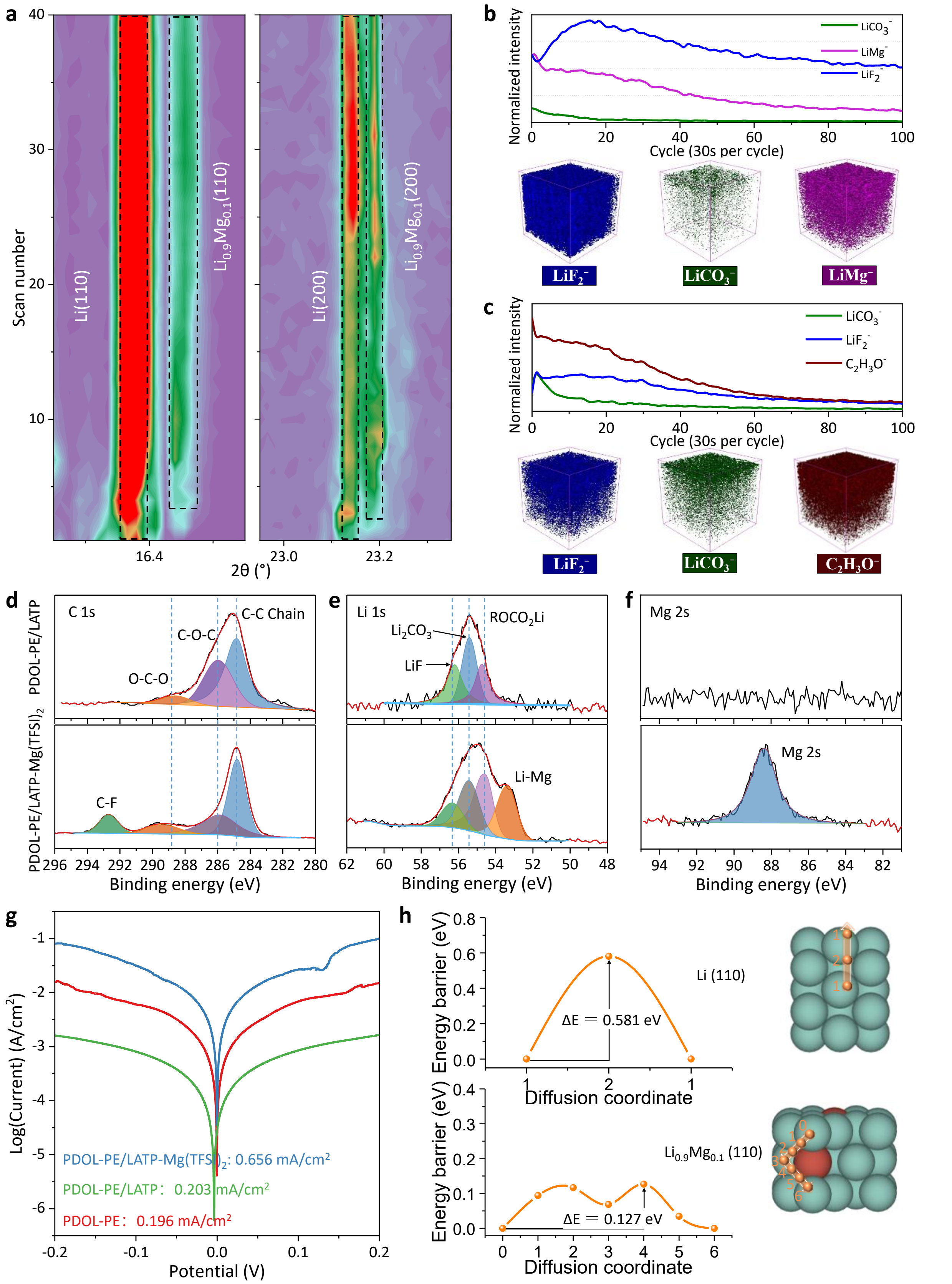

Fig. 4 | The compositional characterization and spatial arrangement of $Mg(TFSI)_2$-dervied SEI.

a The real-time phase evolution of the Li| PDOL-PE/LATP-$Mg(TFSI)_2$ |Li cell during formation

process (Mo K-alpha radiation). **b** TOF-SIMS depth profiling and 3D spatial distribution of LiF_2^-, $LiCO_3^-$ and $LiMg^-$ fragments on the surface of lithium foil cycled for 100 hours in the Li| PDOL-PE/LATP-Mg(TFSI)$_2$ |Li cell. **c** TOF-SIMS depth profiles and 3D spatial distribution of LiF_2^-, $LiCO_3^-$ and $C_2H_3O^-$ fragments on the surface of lithium foil cycled for 100 hours in the Li| PDOL-PE/LATP |Li cell. **d–f** C 1s, Li 1s and Mg 2s core-level XPS spectra of Li foil disassembled from the Li| PDOL-PE/LATP-Mg(TFSI)$_2$ |Li cells after 100 hours cycling. **g** Tafel plots of the Li symmetric cells utilizing PDOL-PE, PDOL-PE/LATP and PDOL-PE/LATP-Mg(TFSI)$_2$. **h** Calculated diffusion pathways and corresponding energy barriers for Li atom diffusion on the surface of Li foil and Li–Mg alloy.

These independent characterizations (Fig. 3 focusing on Cathode/TFPP and Fig. 4 focusing on Anode/Mg) collectively provide the chemical basis for the "Division of Labor" strategy. To make this clearer for readers, we have revised the figure captions in the revised manuscript to explicitly link these characterization results to the ablation study logic.

4. Insufficient disclosure of practical pouch-cell metrics and incomplete electrochemical reporting

For claims of high gravimetric/volumetric energy density (Wh kg^{-1}, Wh L^{-1}) in Ah-scale pouch cells, it is essential to disclose the cell design metrics that govern practical relevance. At minimum, N/P ratio, electrolyte amount (E/C, g Ah^{-1}), and stack pressure must be reported explicitly. Additionally, presenting capacity retention alone is not sufficient for evaluating cell-level performance.

Suggested revisions/experiments:

• Report N/P ratio, E/C ratio (g Ah^{-1}), and stack pressure used during assembly and cycling.

• Provide plots of capacity vs cycle number, along with coulombic efficiency and voltage polarization evolution.

Response: *We sincerely appreciate the reviewer's emphasis on the transparency of cell design metrics. We agree that explicitly reporting parameters such as N/P ratio, E/C ratio, and stack pressure-along with a complete electrochemical profile-is essential for validating claims of high energy density and ensuring the practical relevance of our work.*

1. Disclosure of Pouch Cell Engineering Parameters

To address this, we have comprehensively revised the manuscript and Supplementary Information as follows: We have updated Fig. 6b to explicitly list the key engineering parameters and added a detailed weight breakdown table in the Supplementary Table 3. As shown in the Fig. 6b, the

1.2 Ah NCM811||Li pouch cells were assembled with an N/P ratio of 2.06, an E/C ratio of 1.5 g Ah^{-1}, and cycled under a controlled stack pressure of 0.2 MPa.

To rigorously support our gravimetric energy density calculations, Supplementary Table 3 provides the specific mass of every cell component, including the cathode, Li anode, electrolyte, separator, pouch film, and tabs. This transparency confirms that our reported energy density values are based on the total cell weight, reflecting a realistic assessment of the technology.

Cell parameters of the pouch cell					
	Parameter	Value		Parameter	Value
Cathode (NCM811)	Area capacity (mAh cm^{-2})	5	Anode (Li)	Area capacity (mAh cm^{-2})	10.3
	Stack pressure (Mpa)	0.2		N/P ratio	2.06
	Number of stacks	6		E/C ratio (g Ah^{-1})	1.5

Fig. 6 | b Specific parameters of the 1.2 Ah pouch cell.

Supplementary Table 3 Detailed weight breakdown of components in the 1.2 Ah pouch cell.

Cell component	Specification	Mass (g)	Mass Fraction
Cathode electrode	High loading (25 mg cm^{-2}, double-sided)	6.3305	63.21%
Anode electrode	Thin Li foil (100 μm)	0.8455	8.44%
Separator	Ultrathin (~8 μm)	0.234	2.34%
Electrolyte	PDOL-based SPE	1.785	17.82%
Packaging	Ultrathin Al-laminate film (68 μm)	0.716	7.15%
Tabs	Al/Ni tabs (Ultra light)	0.104	1.04%

Total		10.015	100.00%

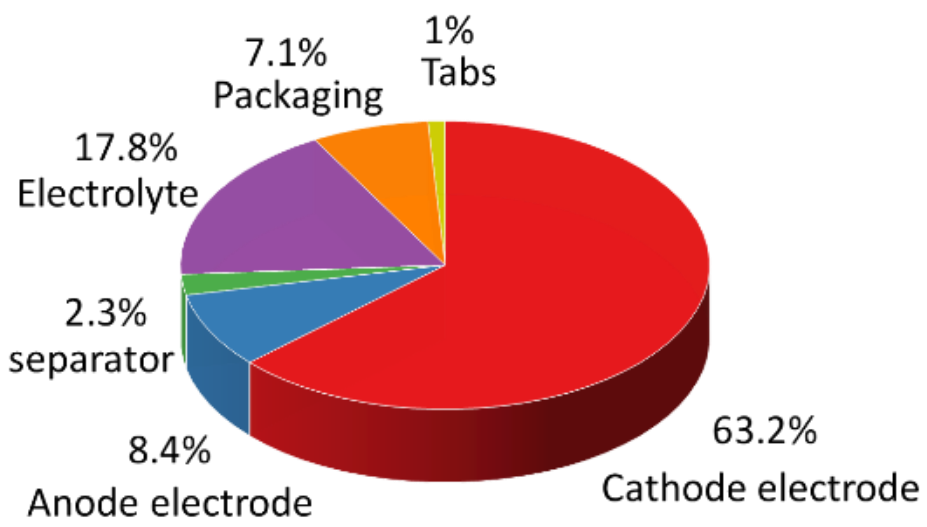

Supplementary Fig. 36 | Mass fractions of individual components in the 1.2 Ah pouch cell.

2. Comprehensive Electrochemical Evaluation of Pouch Cells

We have expanded the electrochemical characterization to demonstrate not just capacity retention, but also voltage stability and Coulombic efficiency (CE) (Fig. 6 c, d). Supplementary Fig. 37 now presents the charge/discharge profiles of the NCM811 pouch cell at selected intervals (e.g., 1st, 100th, 200th, 300th and 400th cycles). The overlap of these voltage profiles and the negligible increase in voltage hysteresis (ΔV) confirm that the cell impedance remains low and stable throughout cycling, validating the robustness of the PDOL-PLMT based interface. The CE evolution is plotted alongside capacity retention, showing a steady value of >99.5%, indicating highly reversible Li plating/stripping.

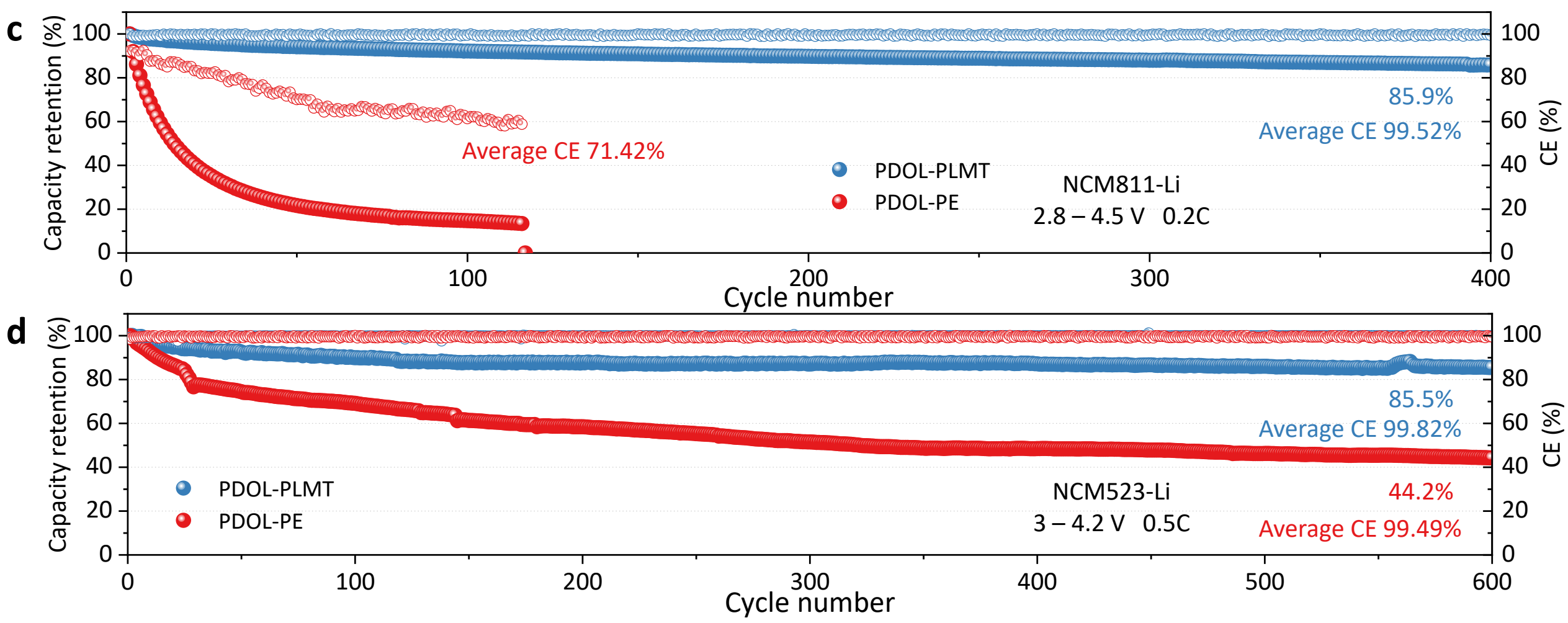

Fig. 6 | c Room-temperature cycling stability of NCM811|Li pouch cells with PDOL-PE and PDOL-

PLMT SPEs within 2.8–4.5 V. **d** Room-temperature cycling performance of NCM523|Li pouch cells with PDOL-PE and PDOL-PLMT SPEs within 3.0–4.2 V.

To further demonstrate the broad applicability of our electrolyte system beyond NCM811, we have included performance data for pouch cells based on NCM523, LCO, and LFP cathodes in Supplementary Fig. 38-40. The charge/discharge curves and CE plots for these diverse chemistries all exhibit stable cycling behaviors. This additional data confirms that our "dual-stabilization" electrolyte strategy is universally effective across different voltage windows and cathode materials, reinforcing the practical potential of the proposed SPE.

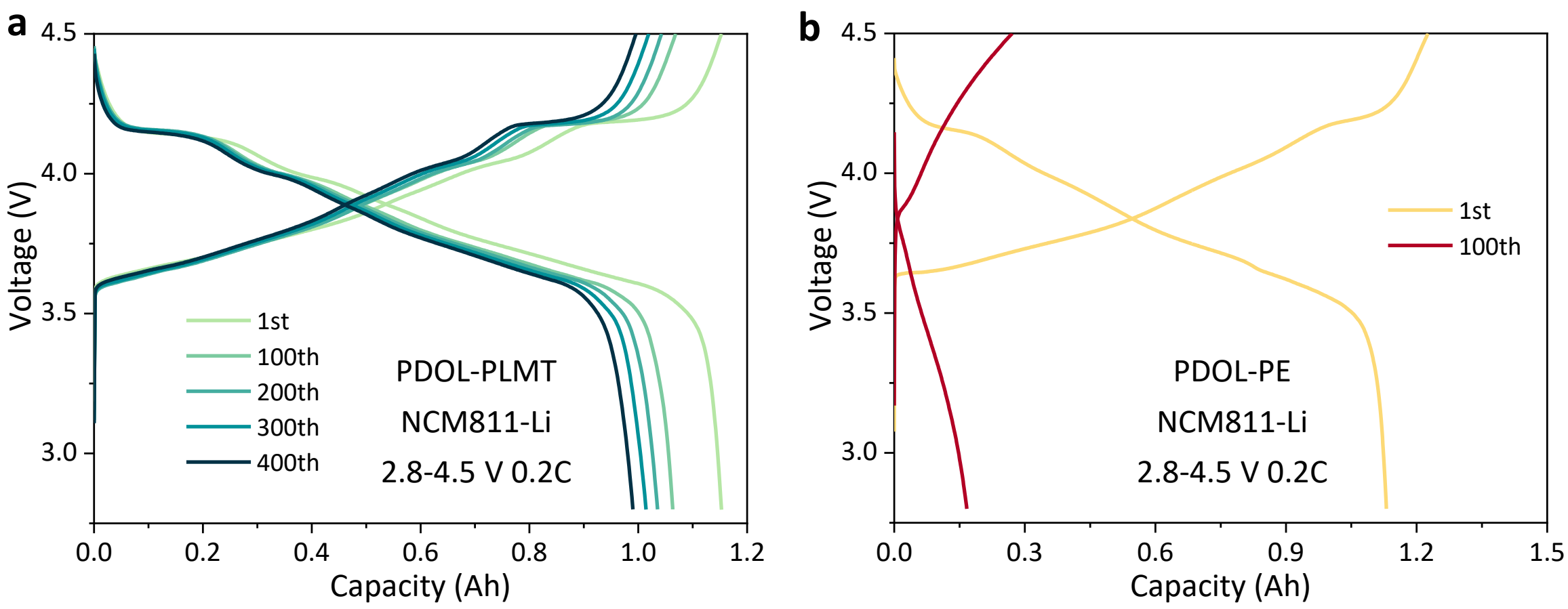

Supplementary Fig. 37 | Electrochemical performance of the 1.2 Ah practical-scale pouch cells at 4.5 V. Charge-discharge profiles of 1.2 Ah (**a**) NCM811|PDOL-PLMT|Li and (**b**) NCM811|PDOL-PE|Li pouch cells cycled between 2.8 - 4.5 V.

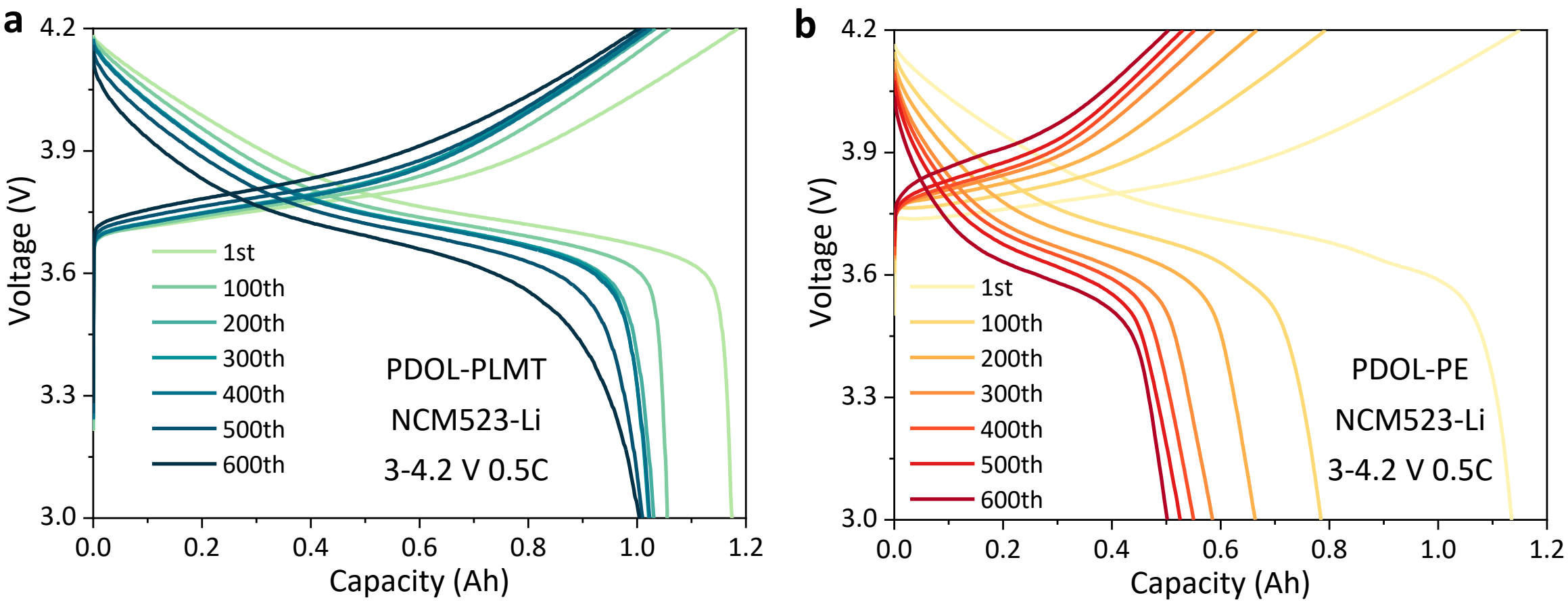

Supplementary Fig. 38 | Demonstration of the electrolyte universality in 1.2 Ah NCM523-based pouch cells. Charge-discharge profiles of (**a**) NCM523|PDOL-PLMT|Li and (**b**) NCM523|PDOL-

PE|Li pouch cells cycled between 3 – 4.2 V.

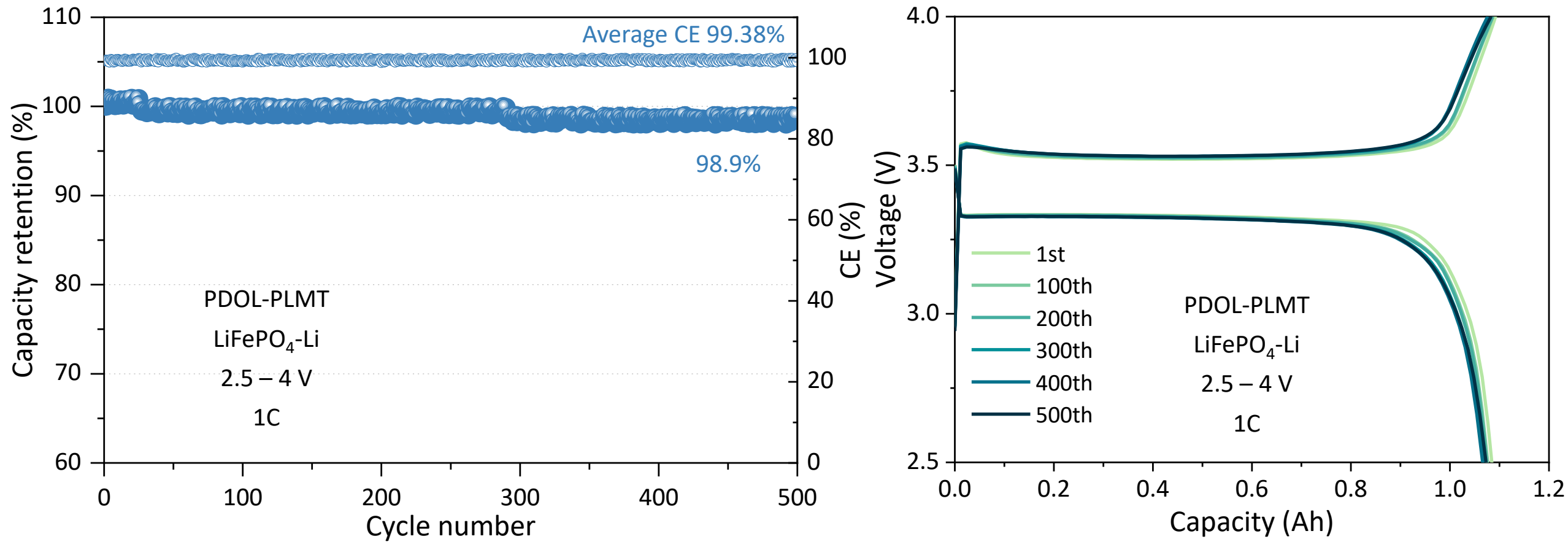

Supplementary Fig. 39 | Cycling performance and Charge-discharge profiles of the $LiFePO_4$|PDOL-PLMT|Li pouch cell at 25 °C.

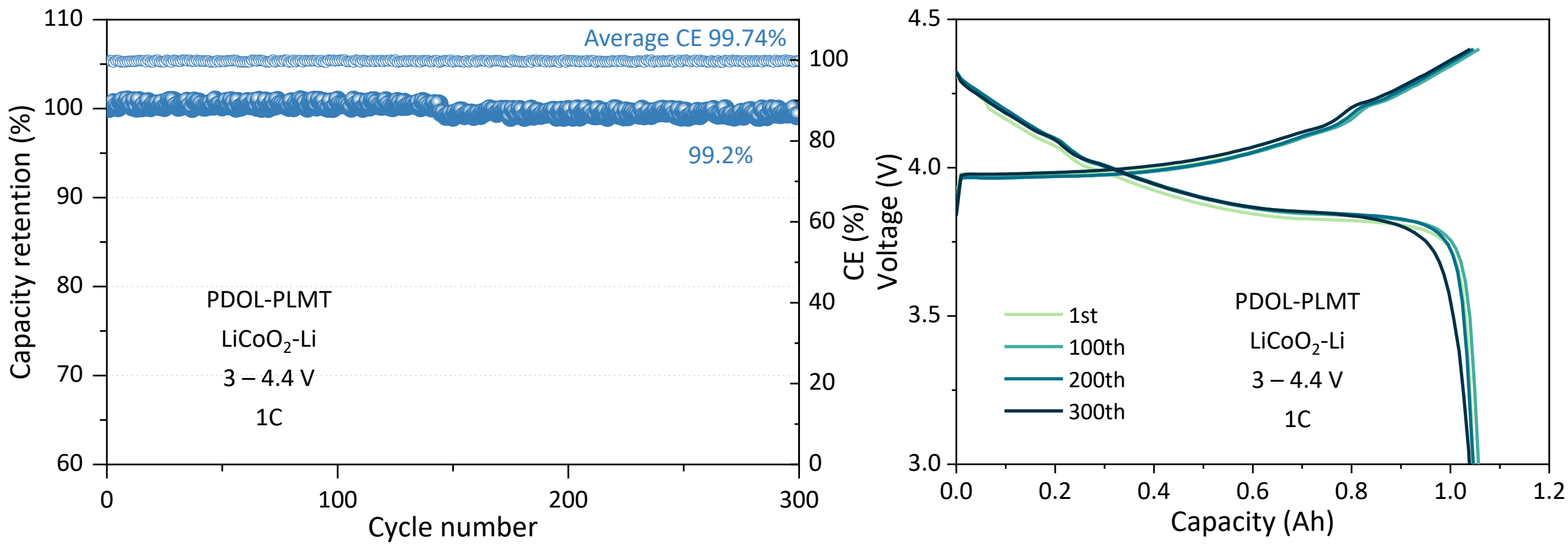

Supplementary Fig. 40 | Cycling performance and Charge-discharge profiles of the $LiCoO_2$|PDOL-PLMT|Li pouch cell at 25 °C.

We have incorporated the aforementioned data into the revised manuscript and supplementary information.

5. Discrepancy between Li symmetric-cell behavior (higher overpotential/impedance) and superior full-cell rate performance.

In the Li symmetric cell tests, PLMT appears to exhibit the largest resistance (or overpotential), which could imply poor rate capability. However, PLMT shows the best rate performance in full cells, which is not consistent with the symmetric-cell results. Therefore, the authors should explain the origin of this discrepancy.

Response: *We thank the reviewer for this insightful observation. The higher initial overpotential*

observed in the PLMT symmetric cells does not reflect poor intrinsic ionic transport, but rather the transient 'formation resistance' required to dynamically construct the Li–Mg alloy interface. Specifically, the in situ reduction of the $Mg(TFSI)_2$ additive demands an initial electrochemical activation energy, inherently creating a temporary kinetic barrier. However, once this activation phase completes, it yields a highly conductive and lithiophilic Li–Mg alloy-rich SEI. This matured interphase drastically lowers the Li^+ diffusion barrier and significantly enhances the exchange current density (j_0). In essence, the symmetric cell data captures the transient 'cost' of building this protective layer, whereas the full-cell rate testing demonstrates the exceptional steady-state kinetic 'benefit' of the established interface.

While we had described this in the original manuscript, we have now added the following key description to the revised manuscript (page 22, line 349, the highlight page) to further clarify this point. "Crucially, the formation of this lithiophilic Li–Mg interphase lowers the Li^+ diffusion barrier and enhances the exchange current density (j_0), fundamentally driving the elevated CCD and fast-kinetic rate performance."

6. Claims of TM dissolution/cross-talk suppression lack quantitative validation

The manuscript implies that TFPP-derived CEI inhibits TM crossover and mitigates cathode-driven degradation. However, presenting a scheme, CEI thickness, and limited microscopy alone is insufficient to substantiate a reduction in TM dissolution and cross-talk especially for Ni-rich NCM811, where TM dissolution and surface reconstruction critically govern long-term fading.

Suggested revisions/experiments:

• Quantify dissolved TM species (Ni/Mn/Co) in the electrolyte using ICP-MS (or equivalent) after defined cycling protocols.

• Quantify TM deposition at the anode via XPS/ToF-SIMS depth profiling (semi-quantitative comparisons across conditions).

• Examine cathode structural degradation after cycling, including particle cracking/pulverization and surface rock-salt reconstruction (cross-sectional SEM/TEM, HRTEM where feasible).

Response: *We fully agree with the reviewer that quantifying TM dissolution is pivotal for validating the efficacy of the CEI, especially for Ni-rich NCM811 cathodes. Qualitative schemes alone are*

indeed insufficient.

1. Quantitative Proof of TM Suppression (ICP-MS)

To rigorously substantiate our claims of "TM crossover suppression" and "dual-interfacial stabilization," we harvested the Li metal anodes from four distinct cell groups after cycling and quantified the deposited TM species using inductively coupled plasma mass spectrometry (ICP-MS). As detailed in Supplementary Fig. 32, the PDOL-PE/LATP exhibited significant TM accumulation (Ni: 34 ppm, Mn: 15 ppm, Co: 13 ppm). In stark contrast, the inclusion of TFPP drastically reduced these values. The PDOL-PE/LATP-TFPP and the full PDOL-PLMT systems showed Ni levels of only 13 ppm and 11 ppm, respectively. This represents a ~66% reduction in total TM crossover. The data provides direct quantitative proof that the TFPP-derived CEI effectively blocks the dissolution of active materials at the source.

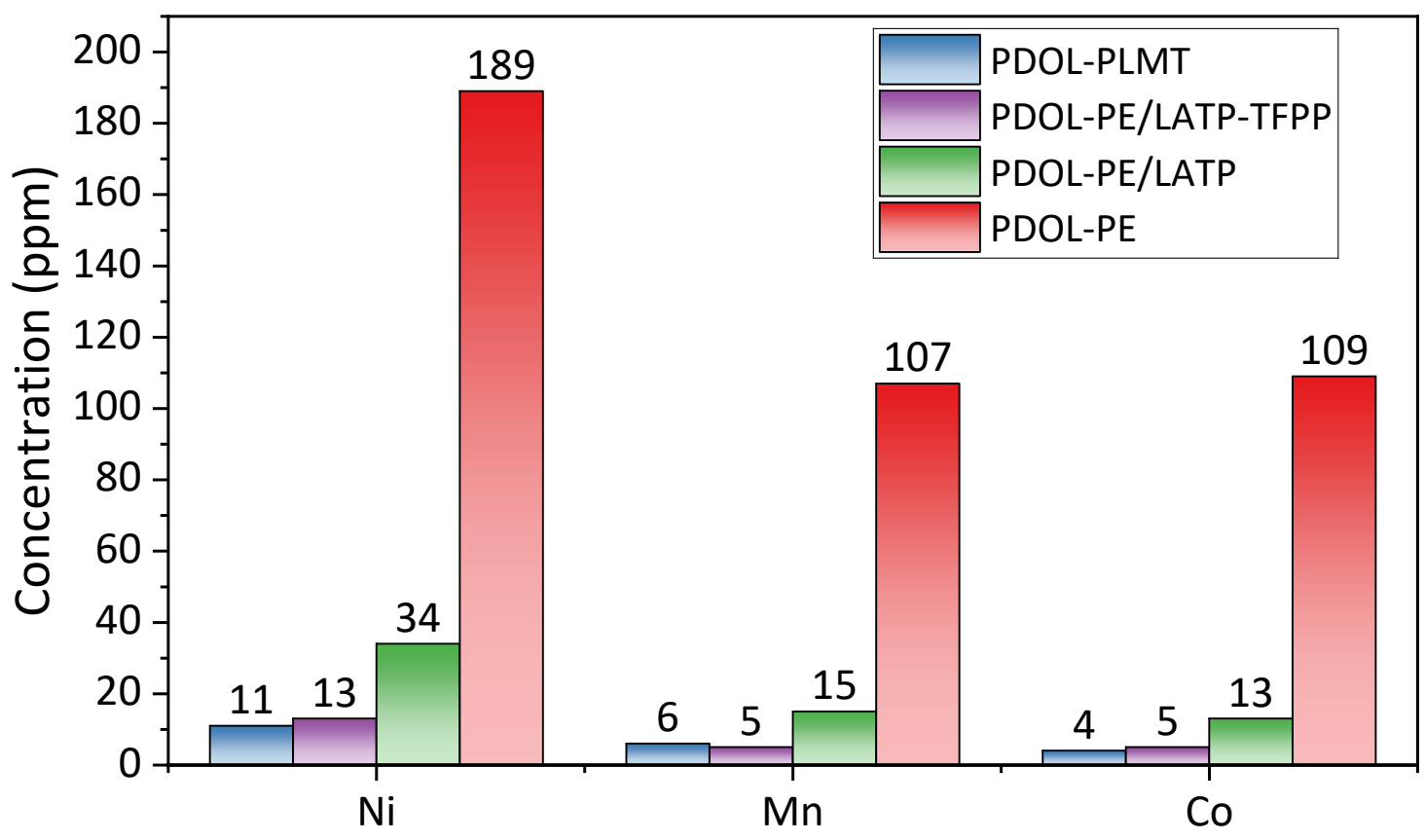

Supplementary Fig. 32 | Quantitative analysis of TM dissolution and migration. Ni, Co, and Mn contents detected on Li metal anodes harvested from cycled NCM811|Li cells assembled with four different electrolytes.

2. Semi-Quantitative XPS Analysis of TM Deposition

We performed high-resolution X-ray Photoelectron Spectroscopy (XPS) analysis on the Li metal anodes harvested from NCM811|Li cells after cycling. We systematically compared the Ni 2p and Mn 2p spectra across three distinct electrolyte configurations: PDOL-PE, PDOL-PE/LATP, and PDOL-PLMT. The XPS results, now presented in Supplementary Fig. 33, clearly reveal a step-wise suppression of TM crossover.

For the additive-free PDOL-PE control sample, we detected pronounced characteristic peaks for both Ni^{3+} *and* Ni^{2+} *species on the Li anode surface. This unambiguously indicates that without*

proper interfacial protection, the NCM811 cathode suffers from severe structural degradation and Ni dissolution, which subsequently migrates to and deposits on the anode, likely catalyzing further parasitic SEI reactions. In the PDOL-PE/LATP sample, the TM deposition is noticeably reduced. Only the Ni^{3+} signal was weakly detected, while the reduced Ni^{2+} species were absent. This semi-quantitative decrease suggests that the LATP-coated PE scaffold provides a certain degree of physical or chemical blocking effect against TM migration, but it remains fundamentally insufficient to completely halt the crossover process. Crucially, for the PDOL-PLMT system, no obvious Ni characteristic peaks could be detected (signals remained at the baseline noise level). This confirms that the TFPP-derived CEI effectively passivates the active material surface, shutting down the dissolution of Ni at its source. Across all three samples, no obvious Mn characteristic peaks were detected. This is likely due to the inherently low atomic fraction of Mn in the NCM811 cathode (10%), rendering its dissolved trace amounts below the XPS surface detection limit in this specific testing timeframe.

This semi-quantitative XPS surface comparison directly corroborates our quantitative ICP-MS data. Together, they provide incontrovertible spectroscopic proof that the dual-additive PDOL-PLMT system successfully eliminates deleterious TM cross-talk, thereby preserving both the cathode structural integrity and the anode SEI stability.

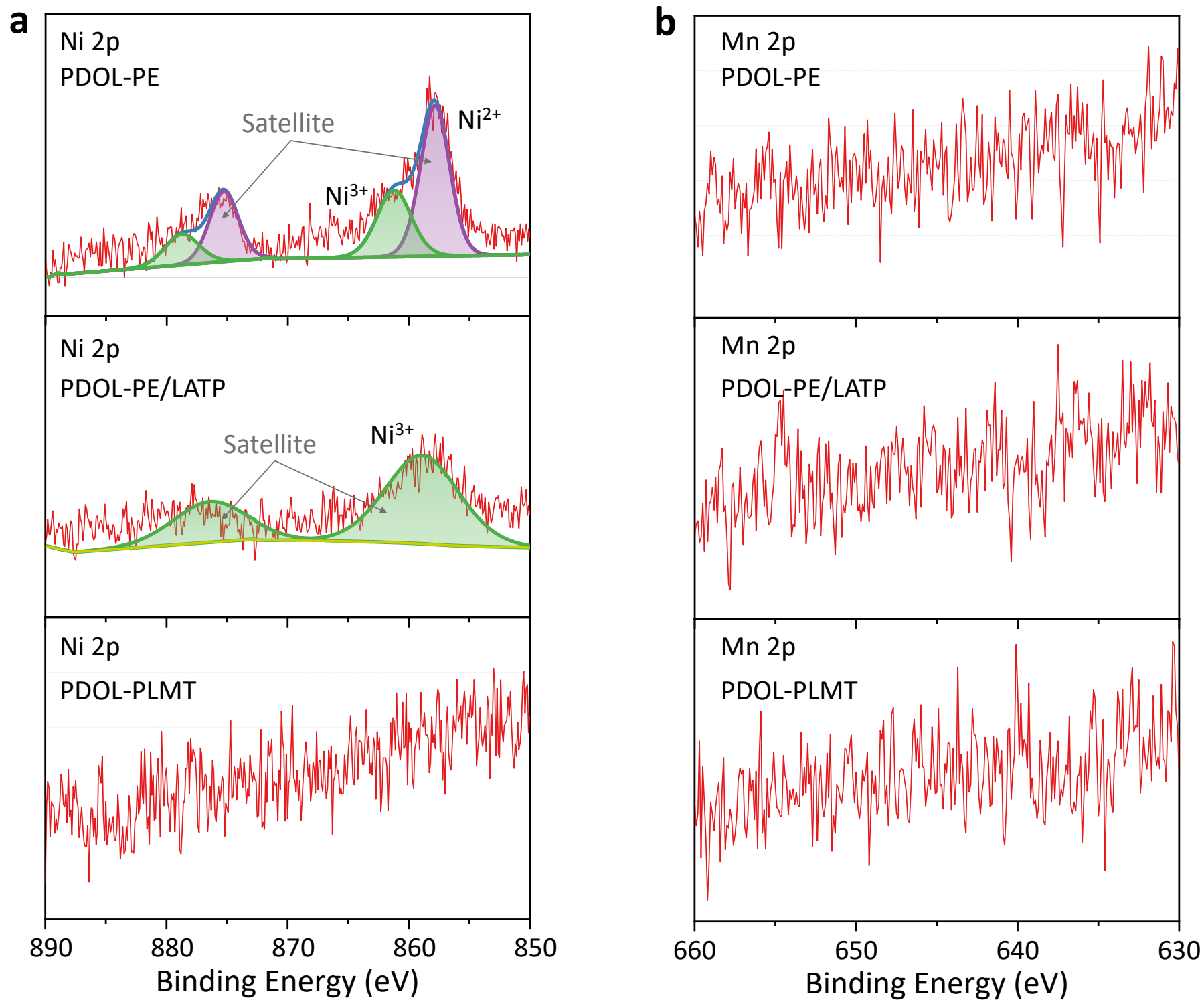

Supplementary Fig. 33 | Surface chemical analysis of the TM crossover. Comparison of the core-level (**a**) Ni 2p and (**b**) Mn 2p spectra detected on Li metal anodes harvested from cycled NCM811|Li coin cells assembled with PDOL-PE, PDOL-PE/LATP and PDOL-PLMT.

3. ToF-SIMS Visualization of TM Suppression

To further visualize the suppression of TM cross-talk, we conducted ToF-SIMS depth profiling on the cycled lithium anodes from the PDOL-PE/LATP and PDOL-PLMT. Consistent with the intrinsic surface instability of Ni-rich cathodes, the Ni signal intensity was predominantly higher (>2x) than that of Mn and Co in the control group. Crucially, the depth evolution profiles reveal a distinct contrast between the two systems: while the control PDOL-PE/LATP sample exhibited significant TM signals persisting deep into the anode bulk even after prolonged sputtering, indicating severe penetration and continuous side reactions, the TM signals in the optimized PDOL-PLMT system were confined strictly to the uppermost surface and rapidly diminished to negligible levels upon sputtering. This confirms that the PDOL-PLMT electrolyte not only reduces the total quantity of dissolved TMs-corroborating our ICP-MS results-but also effectively blocks their deep diffusion into the anode SEI, thereby mitigating the catalytic degradation of the lithium metal.

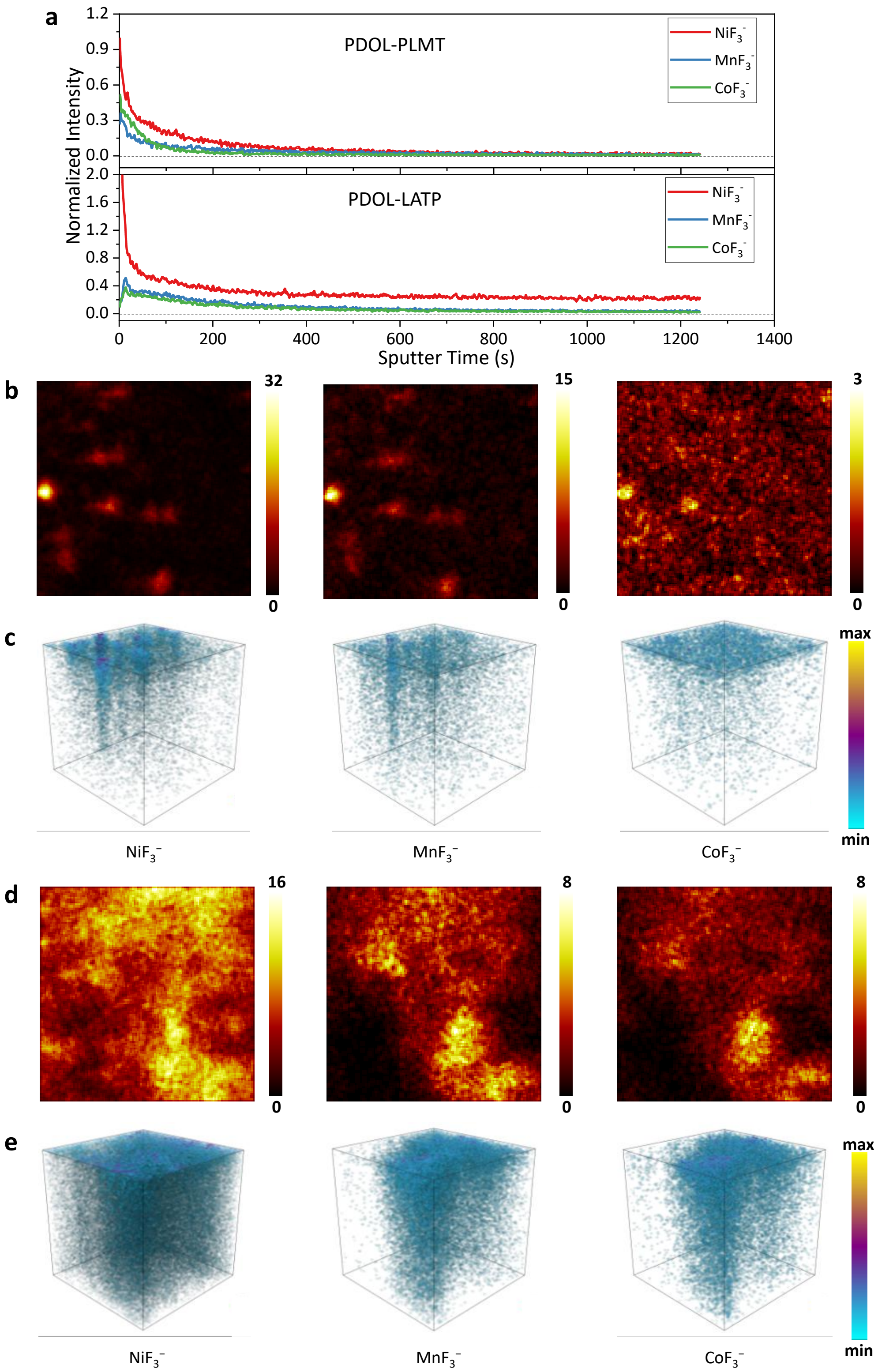

Supplementary Fig. 34 | 3D visualization of the TM species on cycled Li anodes. **(a)** Comparative TOF-SIMS depth profiles of transition metal fluoride fragments (NiF_3^-, MnF_3^-, and CoF_3^-) on Li metal anodes harvested from NCM811|PDOL-PLMT|Li and NCM811|PDOL-PE/LATP|Li pouch cells after 400 cycles. 2D mapping images and 3D reconstructions of the corresponding species on the Li anode

surfaces retrieved from the (**b**, **c**) PDOL-PLMT and (**d**, **e**) PDOL-PE/LATP systems.

4. Correlating Chemical and Mechanical Stability

We have also correlated this chemical stability with mechanical integrity. As presented in Supplementary Fig. 25a–d, we utilized Focused ion beam scanning electron microscopy (FIB-SEM) to examine the cross-sections of cycled NCM811 particles. The control particles (PDOL-PE/LATP) displayed extensive intergranular microcracks, a hallmark of stress corrosion cracking accelerated by electrolyte infiltration. Conversely, the PDOL-PLMT cycled particles remained smooth and structurally intact. This morphological stability directly supports the ICP-MS and ToF-SIMS results: the robust, TFPP-derived CEI suppresses the surface parasitic reactions that lead to particle pulverization and subsequent TM dissolution.

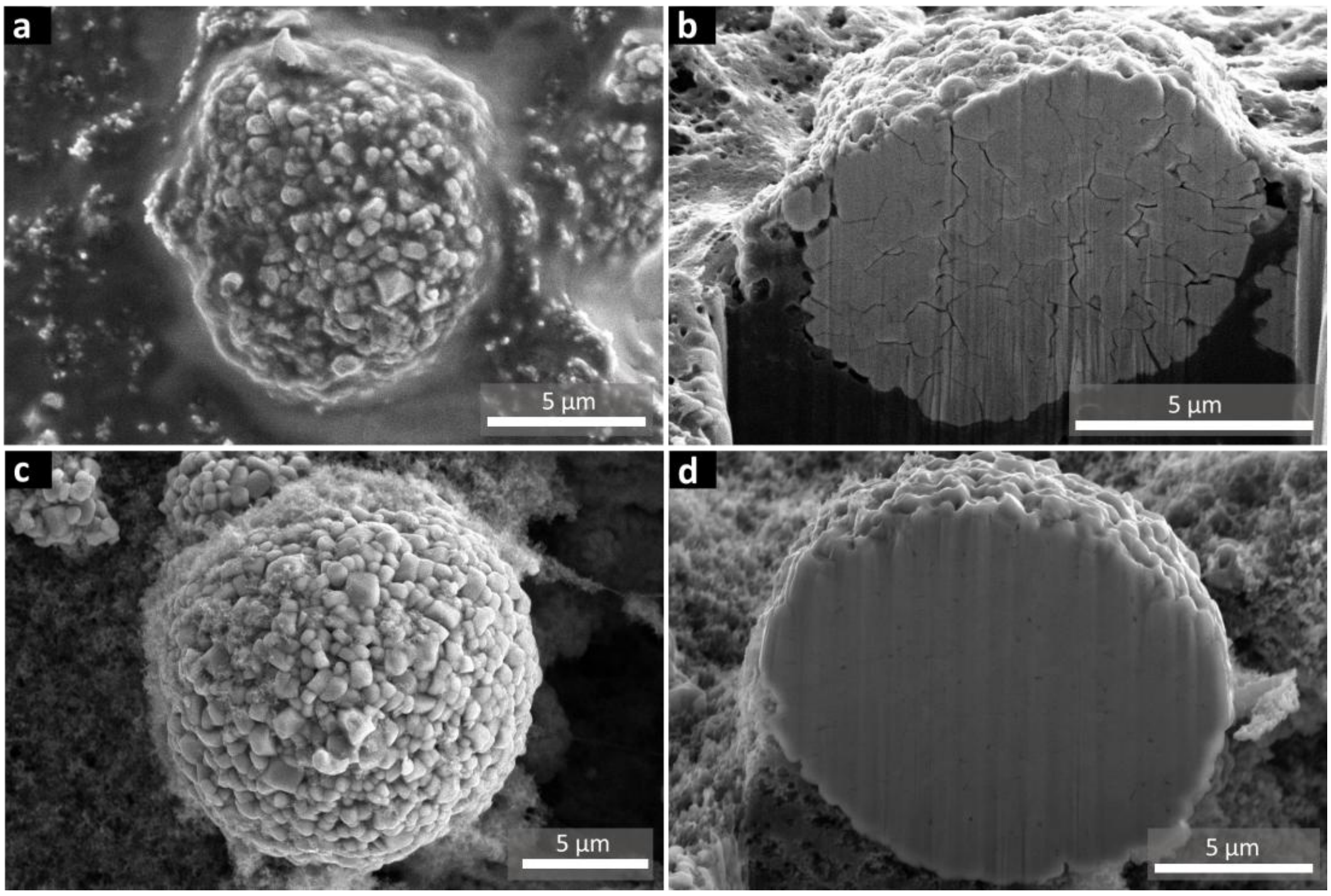

Supplementary Fig. 25 | Morphological stability and suppression of internal microcracking in the NCM811 cathodes. Surface and FIB-prepared cross-sectional SEM images of the NCM811 cathodes after 150 cycles (2.8–4.5 V) retrieved from the (**a**, **b**) NCM811|PDOL-PE/LATP|Li and (**c**, **d**) NCM811|PDOL-PLMT|Li cells.

7. Insufficient interpretation of LSV behavior, especially the sharp current rise near ~4.8 V in LATP–TFPP

Because electrochemical stability window expansion is a key claim, the LSV interpretation must be

rigorous. A rapid current increase near ~4.8 V for the LATP–TFPP condition could be perceived as early oxidation/decomposition rather than improved stability, especially if other controls show more gradual current onset. The manuscript needs to clarify whether this feature corresponds to TFPP sacrificial oxidation forming a protective CEI, or to undesirable electrolyte decomposition.

Suggested revisions/experiments (recommended, likely necessary):

• Provide a clear explanation of the LSV onset criteria, electrode configuration, and scan conditions, and interpret the current rise with supporting evidence.

• If the sharp rise reflects sacrificial oxidation, correlate it with CEI formation evidence.

Response: *We appreciate the reviewer's rigorous scrutiny regarding the electrochemical stability window. We agree that LSV interpretations can sometimes be ambiguous and that a sharp current rise could be misconstrued as electrolyte decomposition. To address this concern and provide a definitive assessment of the oxidative stability, we have (1) re-evaluated the LSV behavior to ensure reproducibility and (2) conducted rigorous steady-state Voltage Floating Tests (chronoamperometry) to complement the dynamic LSV scans.*

1. Re-evaluation of LSV Reproducibility

We repeated the LSV measurements for the PDOL-PE/LATP-TFPP electrolyte. As shown in the Fig. 3c, the new data exhibits a smooth current profile without the sharp, abrupt rise near 4.8 V that was previously observed. The earlier anomaly may have been due to minor interfacial impurities or instrumental artifacts. The updated LSV curve confirms an onset oxidation potential extending beyond 4.8 V (vs. Li/Li$^+$).

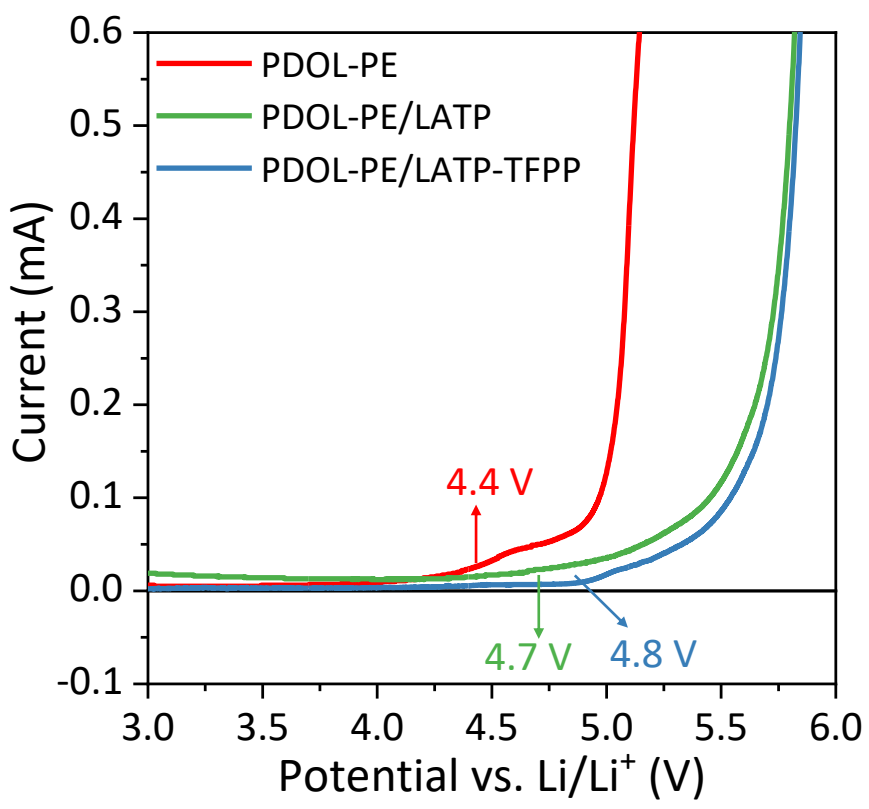

Fig. 3 | c LSV curves of PDOL, PDOL-PE/LATP and PDOL-PE/LATP-TFPP SPEs after the formation cycle.

2. Steady-State Voltage Floating Tests

Recognizing that LSV can sometimes overestimate stability due to its dynamic nature, we performed voltage floating tests (holding the cell at fixed high voltages and monitoring leakage current) to rigorously verify the stability limit. We compared three distinct electrolyte systems: PDOL-PE: Exhibits a sharp increase in leakage current at 4.5 V, indicating rapid polymer decomposition. PDOL-PE/LATP: Shows improved stability but fails near 4.7 V, where leakage current rises significantly. PDOL-PE/LATP-TFPP: Demonstrates superior stability. Even when held at 4.8 V, the leakage current remains negligible and stable over time.

The combination of the smooth LSV profile and the low leakage current in the floating test confirms that the PDOL-PE/LATP-TFPP electrolyte does not undergo continuous decomposition at 4.8 V. Instead, the TFPP additive likely undergoes a self-limiting sacrificial oxidation to form a thin, passivating CEI, which effectively suppresses further electrolyte oxidation. We have included the figures in the Supplementary Information (Supplementary Fig. 12) and added additional discussion to the revised manuscript (page 14 line 233).

We have also updated the Experimental Section to include the detailed protocols for both the LSV and Voltage Floating Tests: The electrochemical stability windows of PDOL-PE, PDOL-PE/LATP and PDOL-PE/LATP-TFPP electrolytes were evaluated via linear sweep voltammetry (LSV) using a stainless steel (SS) working electrode and a Li counter/reference electrode at a scan rate of 0.1 mV s^{-1}. This paragraph "Electrochemical floating tests were performed on NCM811|Li cells using various SPEs. The cells were initially charged to 4.3 V, followed by a series of potentiostatic steps from 4.3 to 4.8 V, with each voltage held for 10 h to monitor the resultant leakage current." has been added in experiment (page 37 line 624).

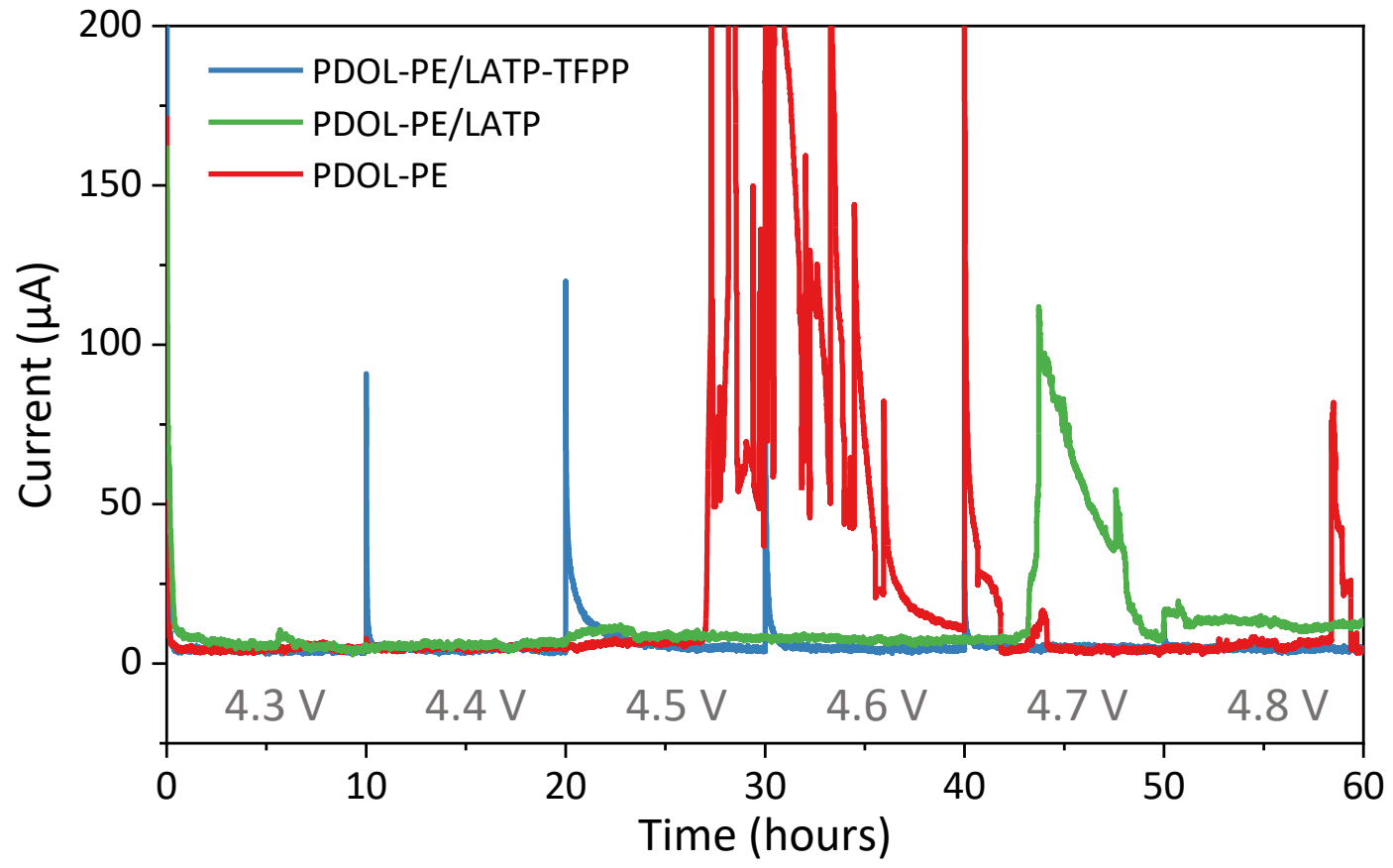

Supplementary Fig. 12 | High-voltage stability of the SPEs. Electrochemical floating tests of NCM811|Li cells assembled with PDOL-PE, PDOL-PE/LATP and PDOL-PE/LATP/TFPP SPEs.

Reviewer #2 (Remarks to the Author):

The manuscript by Tang et al. reports a catalytically designed Solid Polymer Electrolyte (SPE) using a poly-DOL/LiTFSI system integrated with LATP and TFPP. The reported performance, specifically the cycling stability of a 450 Wh/kg, 1.2 Ah pouch cells at a high voltage of 4.5 V, is very impressive and surprising. Based on the reviewers understanding, poly-DOL SPE would face stability challenges towards high-voltage cathode and Li metal anode. The current version lacks several critical data required to support such extraordinary claims. The following points should be addressed before further consideration.

Response: *We sincerely thank the reviewer for the encouraging recognition of our Ah-scale pouch cell performance and the critical insights regarding the stability of poly-DOL systems. In this revision, we have comprehensively strengthened the manuscript with the requested critical data—focusing on rigorous quantitative interfacial analysis—to bridge the gap between conventional understanding and our reported performance, thereby firmly substantiating these claims.*

1. Since the electrolyte system is essentially ether-based, its operation at 4.5V presents a huge challenge. To justify the claimed high-voltage tolerance, the authors should provide leakage current measurements. Specifically, potentiostatic hold tests (leakage current) should be performed on full cells held at a constant 4.5V. And comparisons between the steady current of different system are also important.

Response: *We sincerely appreciate the reviewer's rigorous scrutiny regarding the oxidative stability of our ether-based system. We fully agree that operating at 4.5 V presents a significant challenge for conventional ether electrolytes, and that potentiostatic leakage current measurements are the definitive standard to justify high-voltage tolerance.*

To rigorously verify the stability limit beyond dynamic LSV scans, we performed Voltage Floating Tests (chronoamperometry) by holding the cells at fixed high voltages and monitoring the steady-state leakage current. As illustrated in Supplementary Fig. 12, the comparative analysis reveals a distinct stability hierarchy. PDOL-PE exhibits a sharp surge in leakage current at 4.5 V, indicative of rapid polymer decomposition. PDOL-PE/LATP shows improved stability but eventually succumbs to significant oxidation near 4.7 V. PDOL-PE/LATP-TFPP demonstrates superior high-

voltage tolerance. Even when held at 4.8 V, the leakage current remains negligible and stable over time.

This low steady-state current confirms that the PDOL-PE/LATP-TFPP electrolyte does not undergo the continuous decomposition typical of ether-based solvents at such voltages. Instead, the data suggests that TFPP undergoes a self-limiting sacrificial oxidation process. This mechanism forms a thin, passivating Cathode Electrolyte Interphase (CEI) that effectively shields the electrolyte from further degradation, thereby enabling stable operation at 4.5 V." We have included the figures in the Supplementary Information (Supplementary Fig. 12) and added additional discussion to the revised manuscript (page 14 line 233).

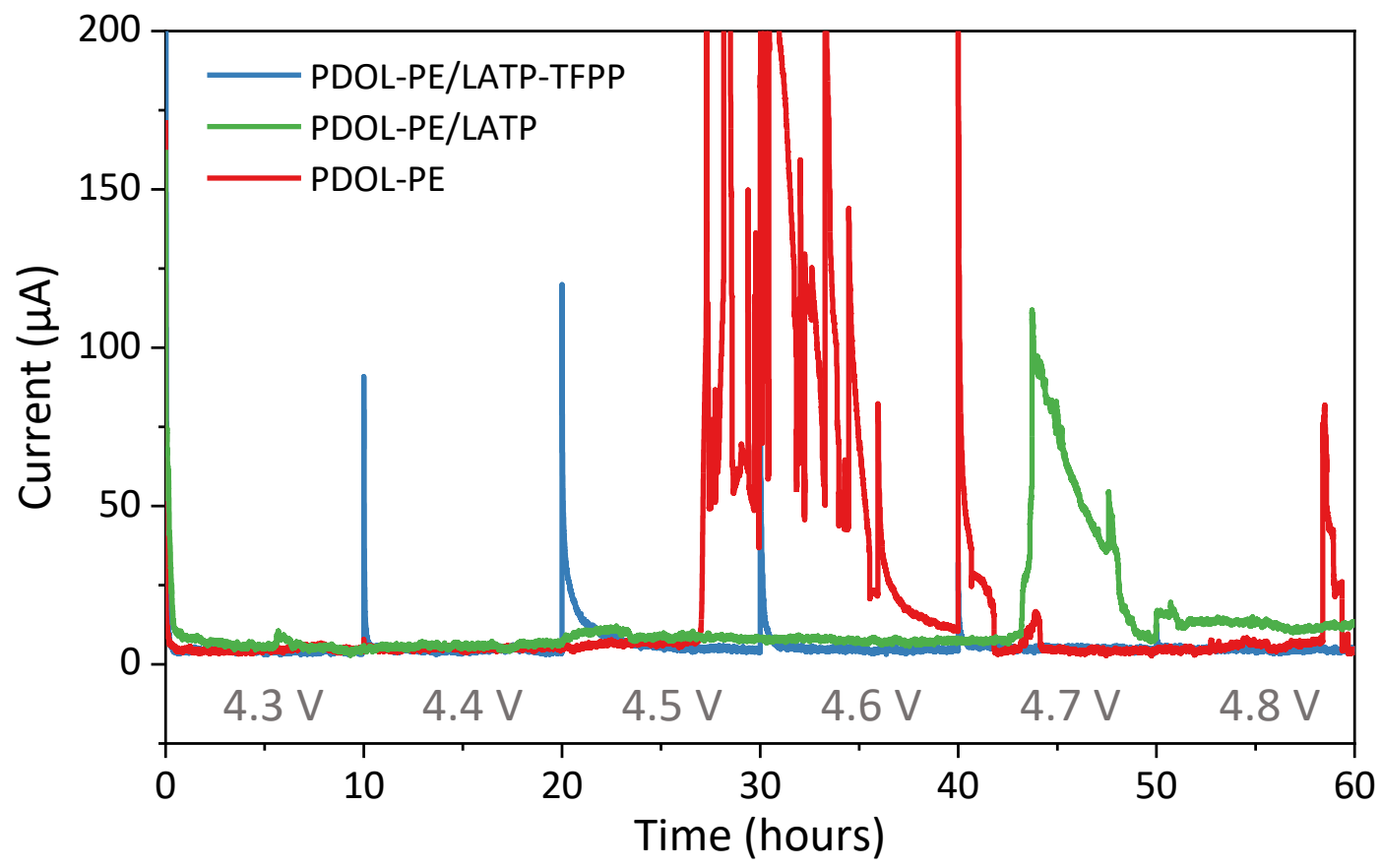

Supplementary Fig. 12 | High-voltage stability of the SPEs. Electrochemical floating tests of NCM811|Li cells assembled with PDOL-PE, PDOL-PE/LATP and PDOL-PE/LATP/TFPP SPEs.

2. The manuscript just gived capacity retention data regarding the electrochemical behavior of the pouch cells. The charge-discharge profiles and corresponding Coulombic efficiency data, for all pouch cells paired with LFP, LCO, and NCM should be provided.

Response: *We appreciate the reviewer's suggestion to provide a more comprehensive electrochemical evaluation. We agree that capacity retention alone is insufficient to fully capture the cell-level dynamics and interfacial stability.*

Accordingly, we have significantly expanded the data in the revised manuscript and supplementary information to include Coulombic efficiency (CE) (Fig. 6c, d) and full charge-discharge profiles evolution for all tested systems: In Supplementary Fig. 37, we present the voltage profiles of the NCM811 pouch cells at selected intervals (e.g., 1st, 100th, 200th, 300th, and 400th

cycles). The voltage profiles exhibit a high degree of overlap with negligible growth in voltage hysteresis (ΔV) over 400 cycles. This confirms that the internal resistance remains low and the PDOL-PLMT based interface is mechanically and chemically robust. The corresponding average CE show a steady value of >99.5%, indicating highly reversible Li plating/stripping without continuous electrolyte consumption.

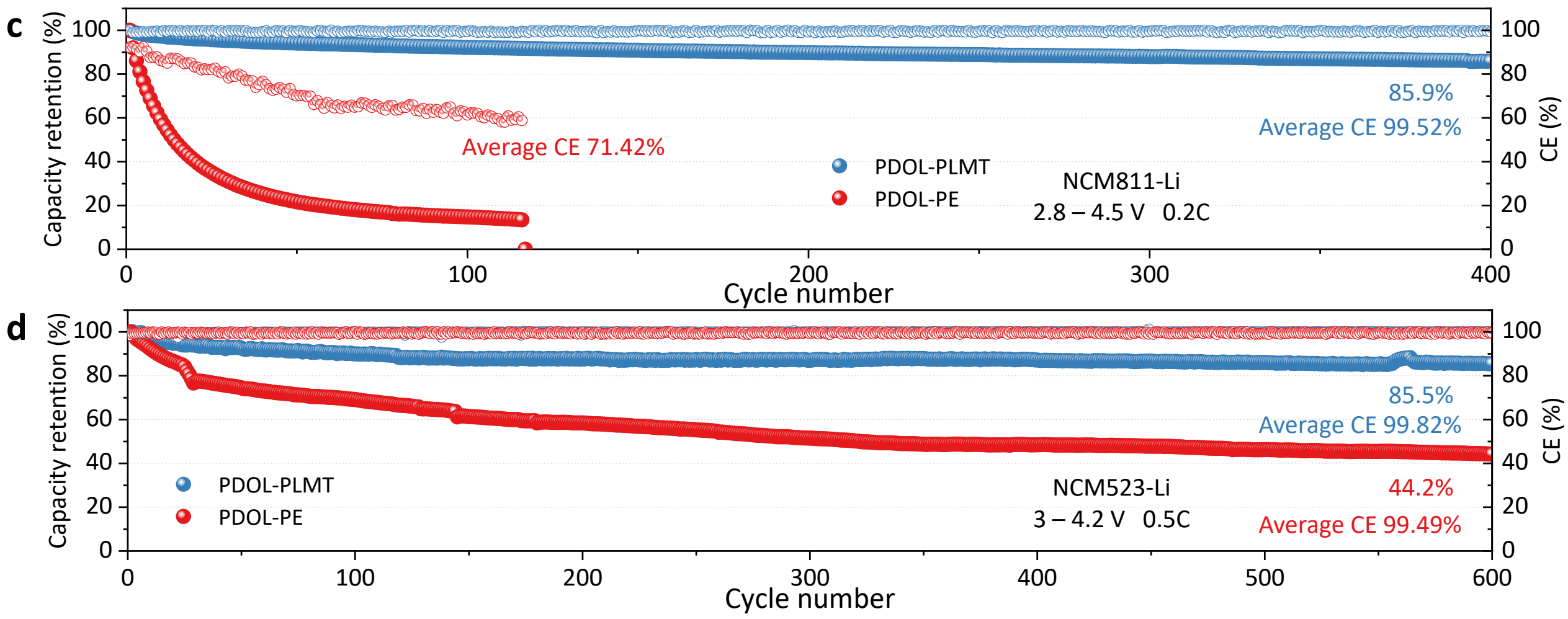

Fig. 6 | c Room-temperature cycling stability of NCM811|Li pouch cells with PDOL-PE and PDOL-PLMT SPEs within 2.8–4.5 V. **d** Room-temperature cycling performance of NCM523|Li pouch cells with PDOL-PE and PDOL-PLMT SPEs within 3.0–4.2 V.

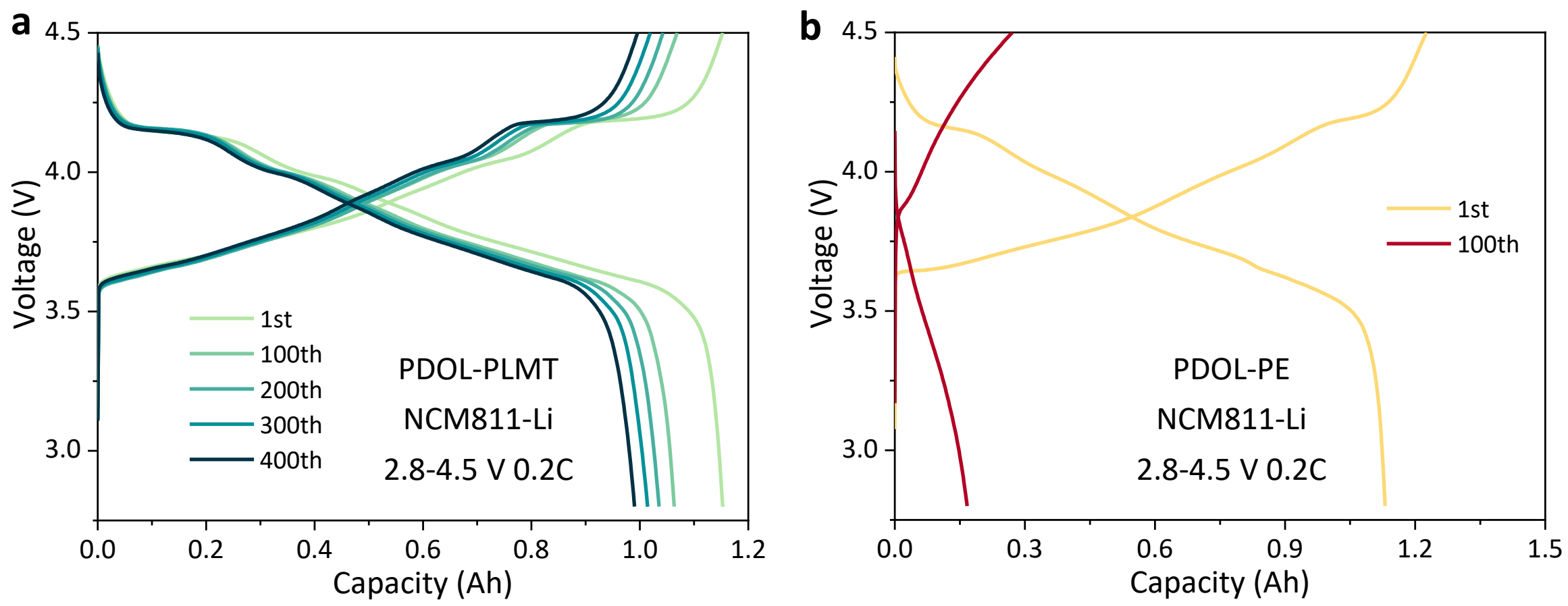

Supplementary Fig. 37 | Electrochemical performance of the 1.2 Ah practical-scale pouch cells at 4.5 V. Charge-discharge profiles of 1.2 Ah (**a**) NCM811|PDOL-PLMT|Li and (**b**) NCM811|PDOL-PE|Li pouch cells cycled between 2.8 – 4.5 V.

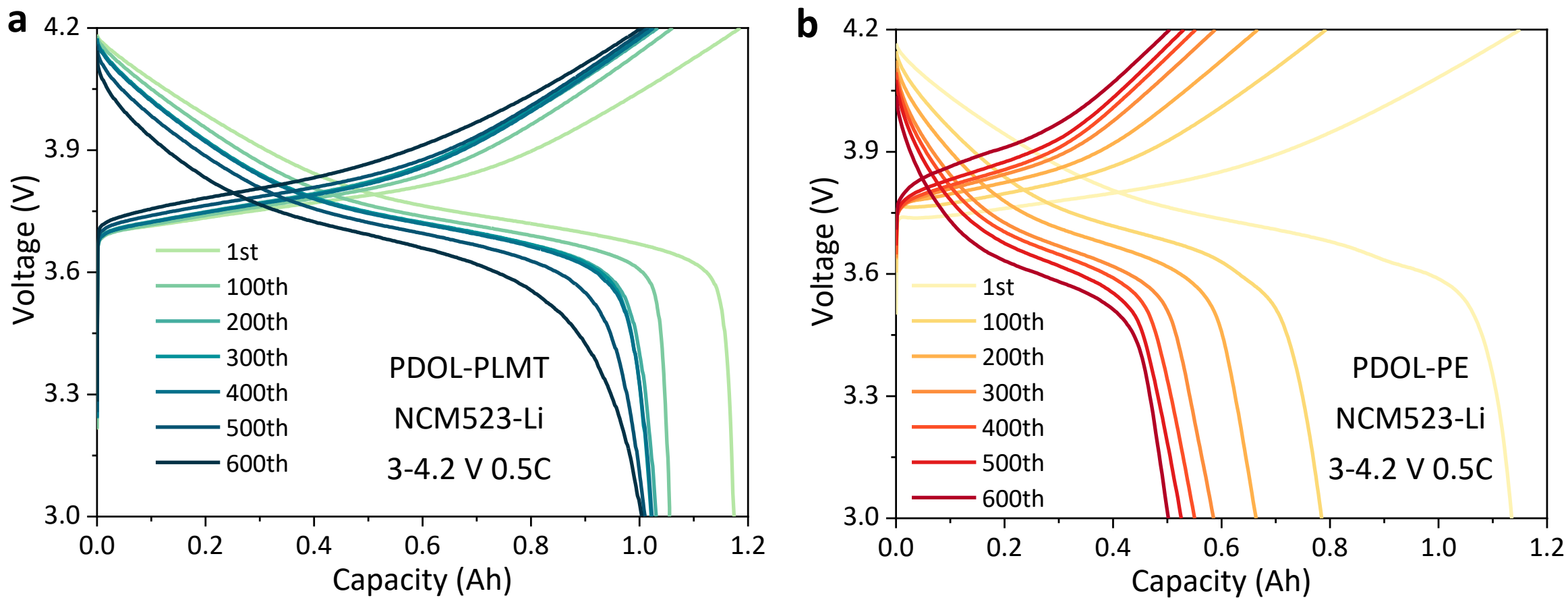

Supplementary Fig. 38 | Demonstration of the electrolyte universality in 1.2 Ah NCM523-based pouch cells. Charge-discharge profiles of (**a**) NCM523|PDOL-PLMT|Li and (**b**) NCM523|PDOL-PE|Li pouch cells cycled between 3 – 4.2 V.

To demonstrate the broad applicability of our electrolyte system and fully address the reviewer's request, we have provided parallel performance data for pouch cells paired with LFP, LCO, and NCM523 cathodes in Supplementary Fig. 38~40. Consistently stable charge/discharge curves and high CE were observed across all these diverse chemistries. This confirms that our "dual-stabilization" strategy is universally effective across different voltage windows and cathode materials, reinforcing the practical potential of the proposed SPE.

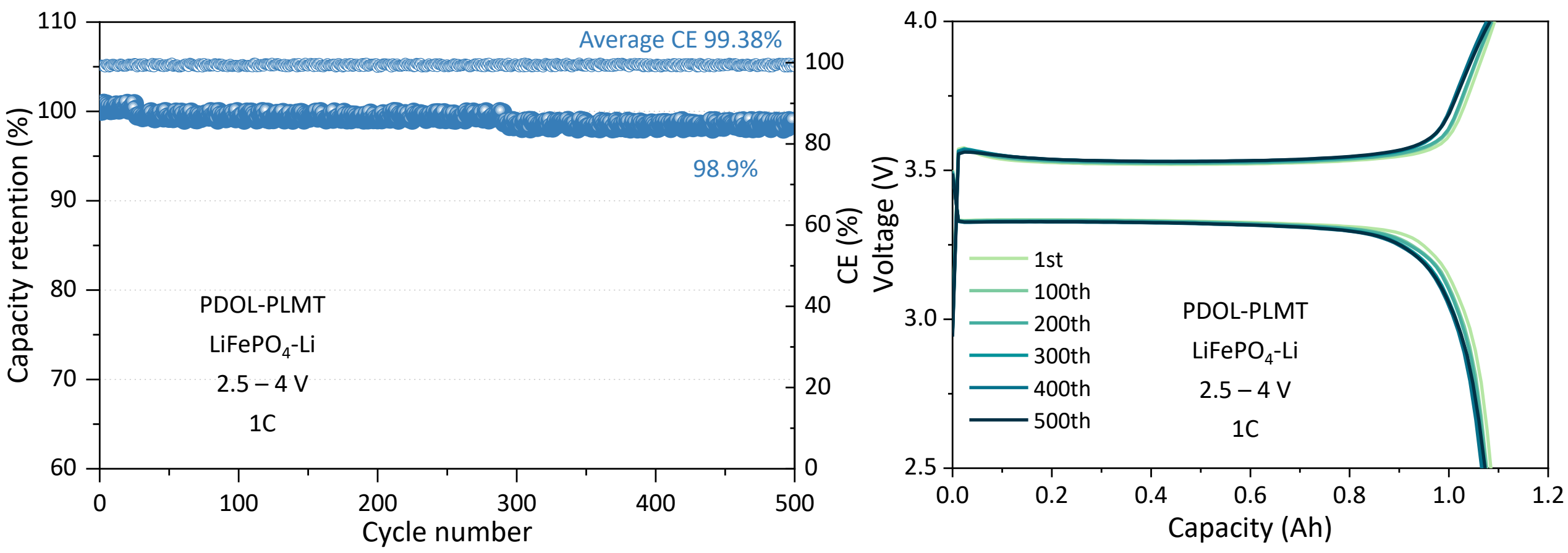

Supplementary Fig. 39 | Cycling performance and Charge-discharge profiles of the $LiFePO_4$|PDOL-PLMT|Li pouch cell at 25 °C.

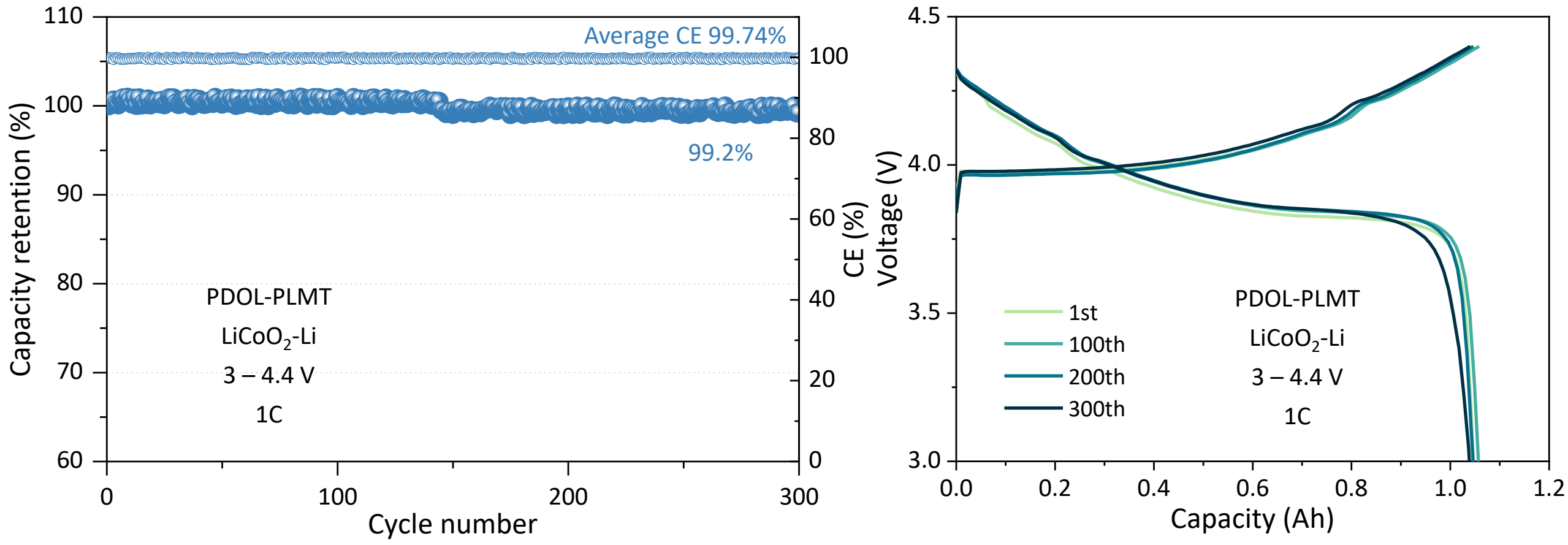

Supplementary Fig. 40 | Cycling performance and Charge-discharge profiles of the $LiCoO_2$|PDOL-PLMT|Li pouch cell at 25 °C.

3. Poly-dol system may face instability towards Li metal anode. To rigorously evaluate the lithium plating/stripping efficiency, the authors should provide Li||Cu asymmetric cell data. Comparisons between different SPEs are also needed.

Recommended parameters: Current density 0.5 mA cm^{-2} and an areal capacity of 5 mAh cm^{-2}.

Response: *We appreciate the reviewer's recommendation to rigorously evaluate the Li plating/stripping efficiency using Li|Cu asymmetric cells. We agree that verifying the anode stability under high areal capacity is critical for assessing the limits of the poly-DOL system. Following the reviewer's suggestion, we assembled Li|Cu cells with four comparative electrolyte systems and tested them under the stringent conditions of 0.5 mA cm^{-2} and 5 mAh cm^{-2}. The results are presented in Supplementary Fig. 20 and summarized below:*

The PDOL-PLMT (dual-additive) system demonstrated exceptional reversibility, achieving a high Average Coulombic Efficiency (ACE) of 99.03%. Crucially, the PDOL-PE/LATP-$Mg(TFSI)_2$ sample exhibited nearly identical performance to the PDOL-PLMT system. This comparative result provides definitive evidence that the $Mg(TFSI)_2$ additive is the dominant factor governing anode stability. The in-situ formation of a lithiophilic Li-Mg alloy interface regulates uniform Li deposition, enabling high efficiency even under deep cycling (5 mAh cm^{-2}) conditions.

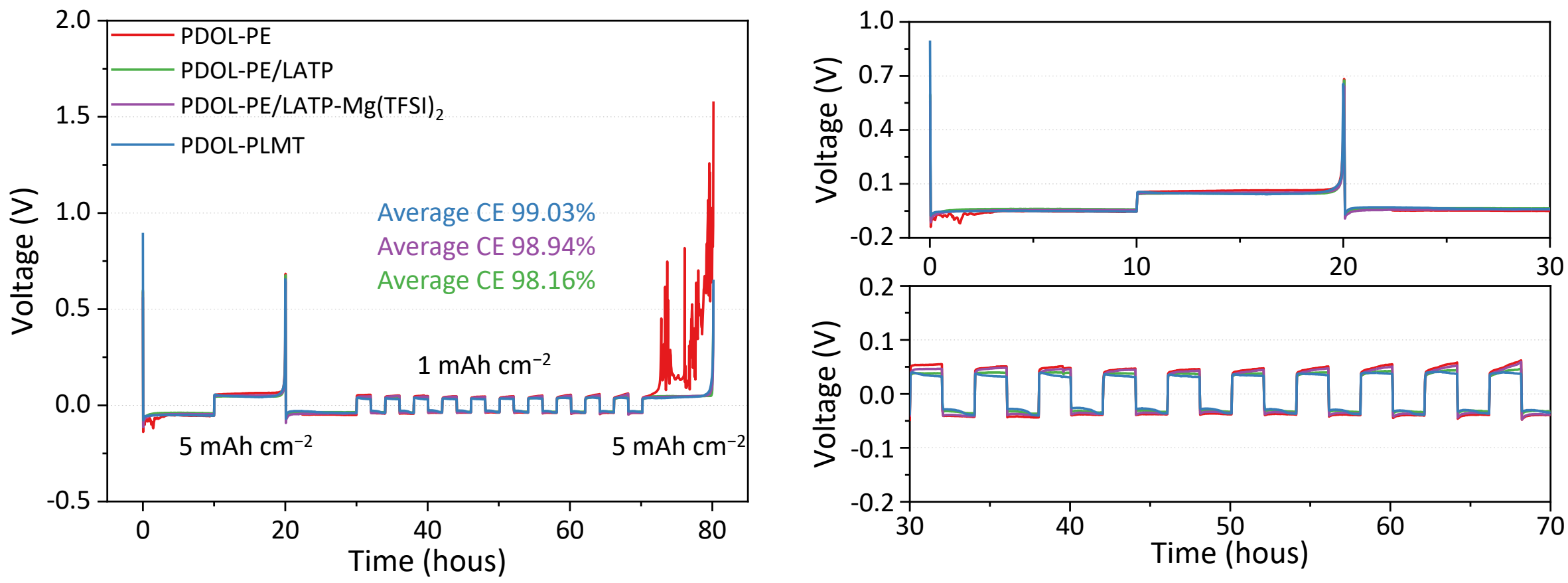

Supplementary Fig. 20 | Electrochemical reversibility of lithium plating and stripping. Comparative Li plating/stripping stability of Li|Cu cells with the four indicated SPEs (PDOL-PE, PDOL-PE/LATP, PDOL-PE/LATP-$Mg(TFSI)_2$, and PDOL-PLMT).

In sharp contrast, the PDOL-PE failed to support such high-capacity cycling. Instability: As shown in the voltage profiles (Supplementary Fig. 20), the PDOL-PE cell exhibited erratic voltage oscillations during the initial plating stages, indicative of uneven deposition and potential dendrite nucleation. During the stripping step after approximately 70 hours, the voltage polarization increased sharply (rapid impedance buildup). We have included the Li|Cu half-cell data figures in the Supplementary Information and added additional discussion to the revised manuscript (page 22, line 352).

4. There is an unit error of the reported room-temperature ionic conductivity. The authors should correct units and disclose the electrolyte thickness and the bulk resistance derived from EIS.

Response: *We thank the reviewer for the careful examination of the transport properties. This comment allows us to clarify the distinction between the reported absolute ionic conductance (G, mS) and the intrinsic ionic conductivity (σ, S cm^{-1}), and to provide the full set of raw parameters derived from EIS.*

The value of 418.7 mS highlighted in the manuscript represents the absolute ionic conductance of the ultrathin electrolyte membrane. We prioritized reporting conductance to emphasize the engineering advantage of our ultrathin design (9.6 μm), as conductance is the direct metric governing the cell's internal resistance (DCR). Full Disclosure of Bulk Resistance and Conductivity: To ensure rigorous reporting standards as suggested, we have explicitly listed the electrolyte thickness (L),

active area (S), and the calculated ionic conductivity (σ) in the revised manuscript. Based on the normalized Nyquist plots provided in Supplementary Fig. 5, the detailed parameters are calculated as follows:

$$G = \frac{\sigma \times S}{L}$$

where L is the thickness of electrolyte membrane (PDOL-PE: 16.7 μm; PDOL-PE/LATP: 9.6 μm), S represents the aera of SS electrode (8 mm × 8 mm × π), σ is the ionic conductivity.

For full transparency, the representative normalized Nyquist plots used for these room-temperature conductivity calculations are explicitly provided in Supplementary Fig. 5, and the temperature-dependent impedance evolution is further illustrated in Supplementary Fig. 7.

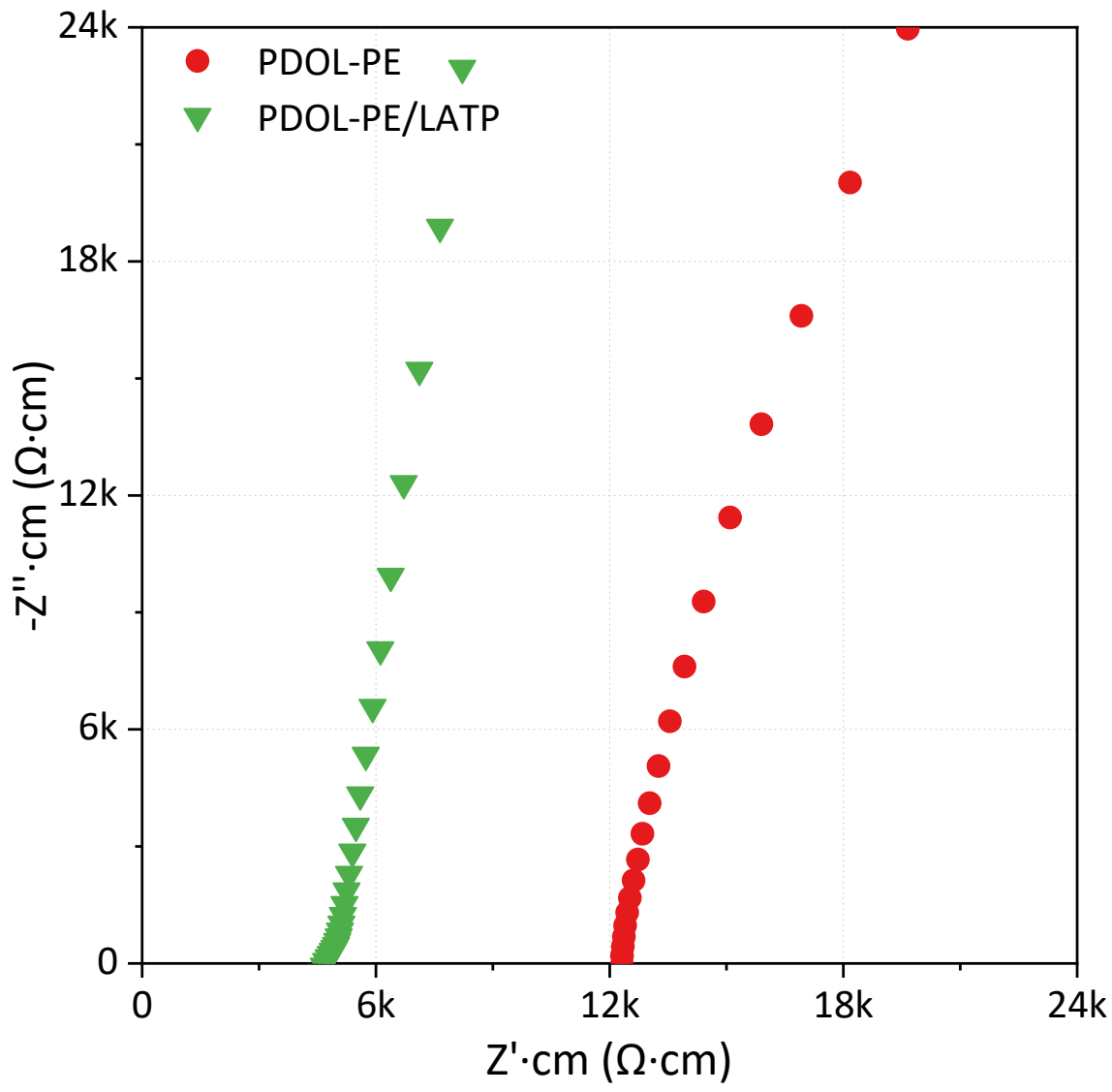

Supplementary Fig. 5 | Room-temperature ionic conductivity evaluation of the SPEs. Typical Nyquist plots of SS|PDOL-PE|SS and SS|PDOL-PE/LATP|SS symmetric cells at room temperature. The impedance data are normalized by the electrode area (8 mm × 8 mm × π) and the respective electrolyte thicknesses (16.7 μm for PDOL-PE and 9.6 μm for PDOL-PE/LATP). SS denotes stainless steel.

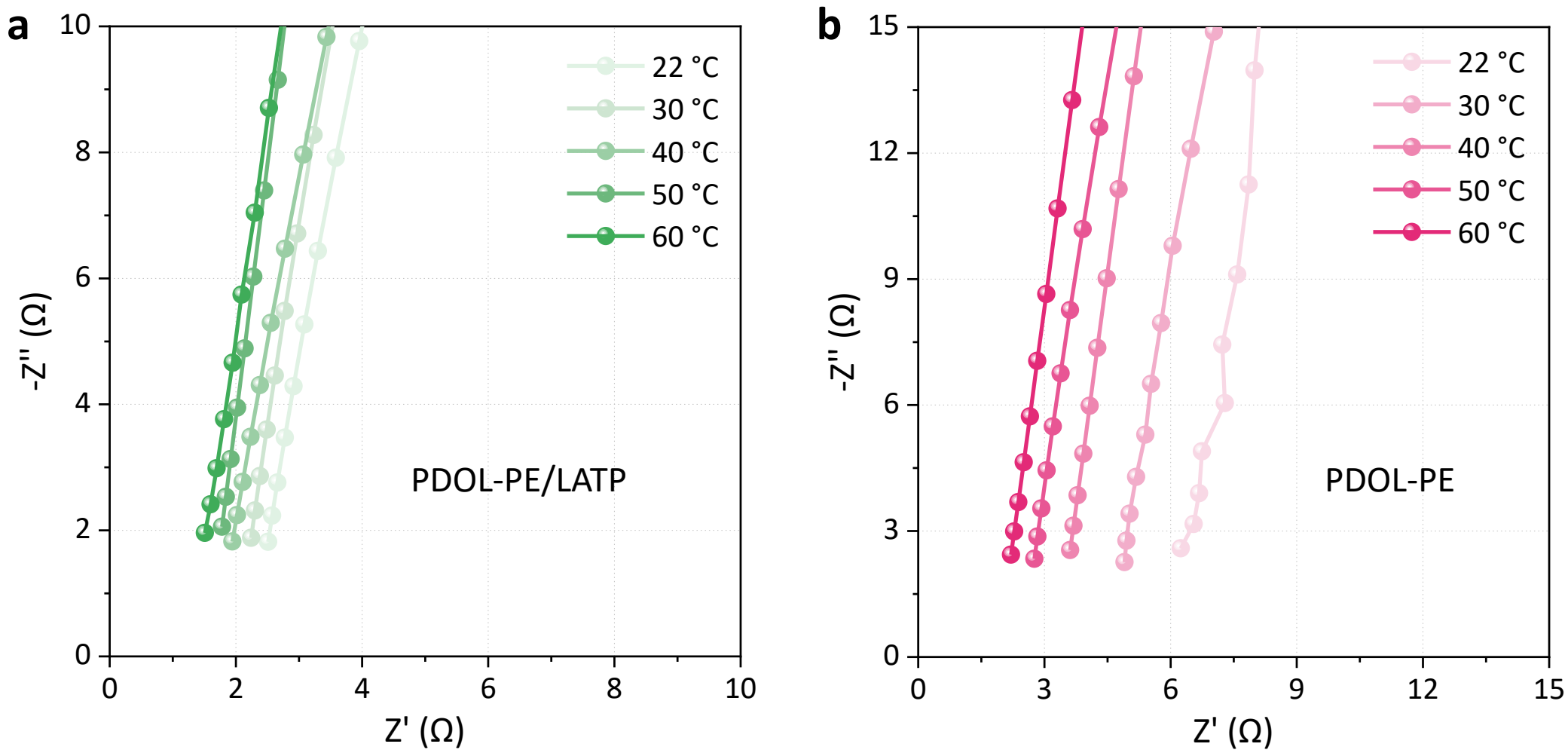

Supplementary Fig. 7 | Temperature-dependent EIS of the SPEs. The EIS curves of the (**a**) SS|PDOL-PE/LATP|SS and (**b**) SS|PDOL-PE|SS symmetric cells at different temperatures.

Reviewer #3 (Remarks to the Author):

This manuscript (NCOMMS-25-105486-T) presents an in-situ polymerization approach to fabricate ultrathin (<10 μm) SPEs, utilizing LATP fillers on a PE separator to catalyze DOL polymerization. Through a dual-additive formulation (TFPP and $Mg(TFSI)_2$), the work targets interfacial stability on both NCM811 cathodes and Li-metal anodes. A key strength of this study is the successful evaluation of 1.2 Ah pouch cells, which demonstrate high energy density (456.7 Wh kg^{-1}) and resilience under safety abuse testing. While the performance is encouraging, the logical connection between the mechanistic investigation and the full-cell results is not fully established. I have identified several significant deficiencies in the experimental design and calculation that require major revisions.

Response: *We sincerely thank the reviewer for the comprehensive evaluation and for recognizing the practical significance of our work, particularly the successful demonstration of the 1.2 Ah pouch cells and their resilience under abuse testing. We respectfully acknowledge the reviewer's critical observation that the logical connection between the mechanistic investigation and the full-cell performance was not sufficiently established in the original manuscript. We also fully accept the criticism regarding the rigor of our experimental design and calculations. To address these significant deficiencies, we have extensively revised the manuscript. Below is a point-by-point response to the questions:*

1. Although Figures 2 and 3 effectively isolate the mechanistic roles of TFPP and $Mg(TFSI)_2$, the battery performance in Figure 4 lacks a necessary comparison with these single-additive systems. To conclusively prove the synergistic effect, the authors are requested to provide cycling data for the PDOL-PE/LATP-TFPP and PDOL-PE/LATP-$Mg(TFSI)_2$ configurations, demonstrating that the dual-additive strategy significantly outperforms the individual approaches.

Response: *We sincerely thank the reviewer for this insightful suggestion. We agree that verifying the performance of single-additive systems is indispensable to conclusively prove the synergistic mechanism of our dual-additive strategy.*

To address this, we synthesized two electrolytes: PDOL-PE/LATP-TFPP and PDOL-PE/LATP-$Mg(TFSI)_2$. We then conducted a comprehensive comparative analysis using both Li symmetric cells and NCM811|Li full cells. The results, presented in supporting information Supplementary Fig. 28-

31, clearly decouple the distinct roles of each additive:

Li symmetric cell cycling at 0.3 mA cm^{-2} reveals the dominance of the Mg additive in anode protection. The PDOL-PE/LATP-Mg(TFSI)$_2$ cell exhibited stable plating/stripping for >800 hours without short-circuiting. This confirms that the in situ formed Li-Mg alloy interface effectively homogenizes Li deposition. In contrast, the PDOL-PE/LATP-TFPP cell suffered from a soft short-circuit no more than 500 hours, indicating that TFPP alone is insufficient to suppress dendrite growth over long durations.

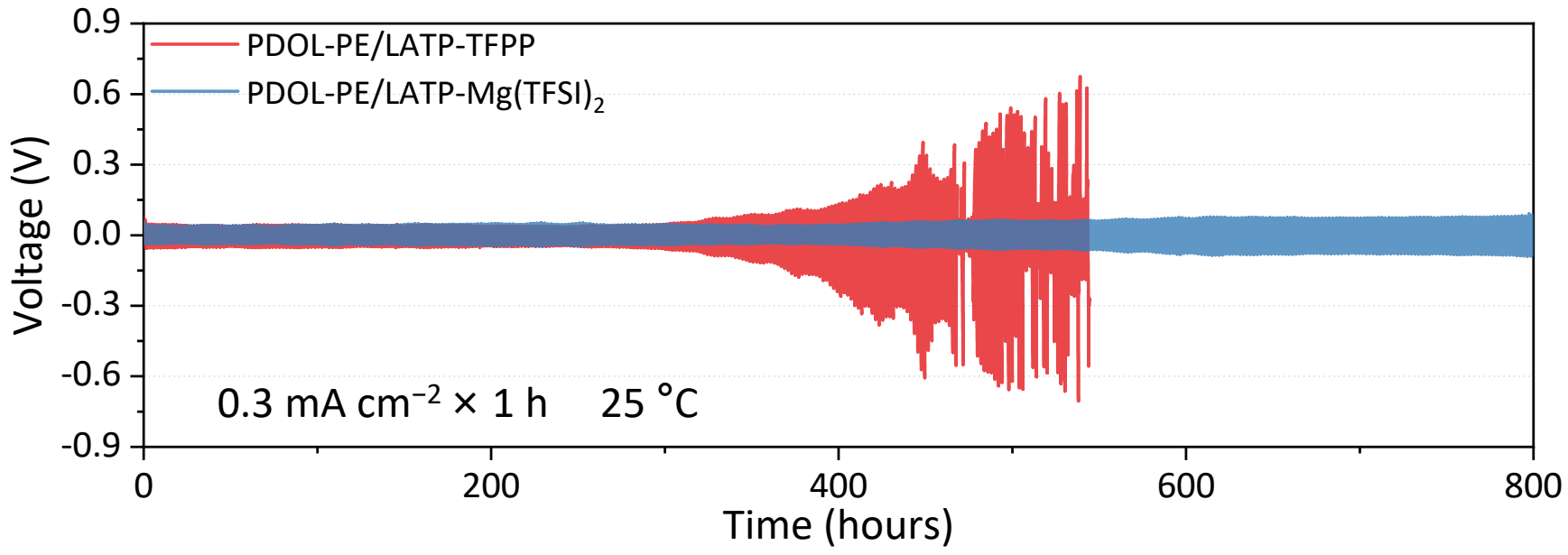

Supplementary Fig. 28 | Long-term interfacial stability and lithium cycling endurance. Galvanostatic Li plating/stripping profiles of the Li|Li symmetric cells employing the PDOL-PE/LATP-TFPP and PDOL-PE/LATP-Mg(TFSI)$_2$ electrolytes.

Full cell cycling of NCM811|Li reveals that TFPP is the key to high-voltage endurance. The disparity becomes stark at a higher cutoff voltage of 4.5 V (Supplementary Fig. 29). The PDOL-PE/LATP-Mg(TFSI)$_2$ system degraded rapidly, retaining only 71.1% capacity after 100 cycles. Conversely, the PDOL-PE/LATP-TFPP system maintained a high retention of 86.2%, approaching the performance of the dual-additive PDOL-PLMT system (89.7%). At 0.5 C (2.8–4.3 V), the PDOL-PE/LATP-TFPP cell showed superior capacity retention (87.2% after 200 cycles) compared to the PDOL-PE/LATP-Mg(TFSI)$_2$ (82.5%). This highlights the indispensability of the TFPP-derived CEI for stabilizing the NCM811 structure under high-voltage stress.

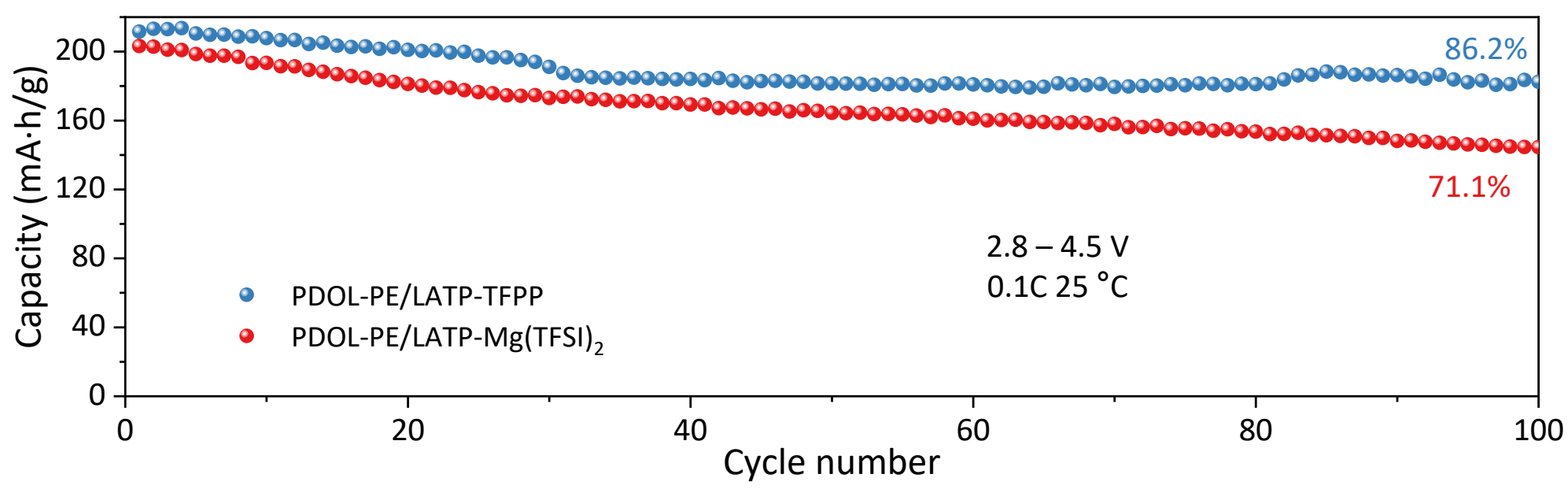

Supplementary Fig. 29 | High-voltage cycling endurance and capacity retention. Long-term

cycling performance of NCM811|Li cells using PDOL-PE/LATP-TFPP and PDOL-PE/LATP-$Mg(TFSI)_2$ electrolytes at 4.5V.

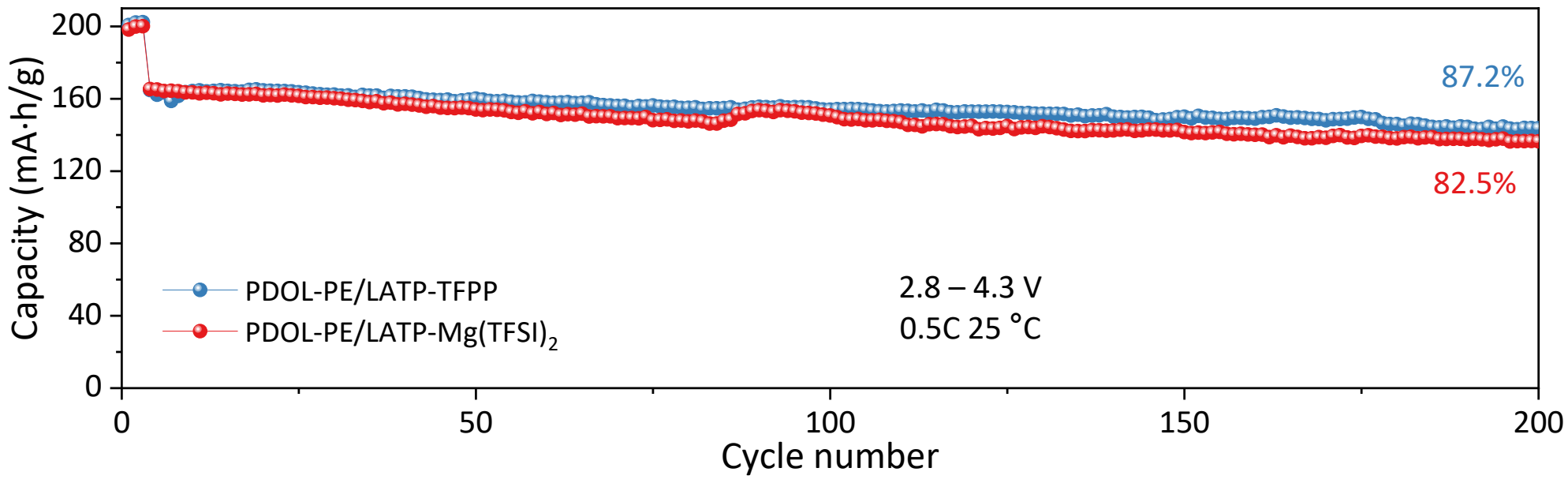

Supplementary Fig. 30 | Comparative evaluation of the long-term cycling endurance. Long-term cycling performance of NCM811|Li coin-cells using PDOL-PE/LATP-TFPP and PDOL-PE/LATP-$Mg(TFSI)_2$ electrolytes at 0.5C.

The PDOL-PE/LATP-$Mg(TFSI)_2$ system exhibited better rate capability at high currents (> 0.5C) due to the fast ion-transport kinetics of the Li-Mg alloy. However, its capacity recovery after high-rate stress was lower, indicating irreversible structural damage. The PDOL-PE/LATP-TFPP system, while showing slightly lower rate capability, demonstrated superior capacity recovery when returned to 0.1C. This implies that the TFPP-derived CEI effectively preserves the cathode integrity during stress, even if it offers slightly lower kinetics than the Mg-based SEI.

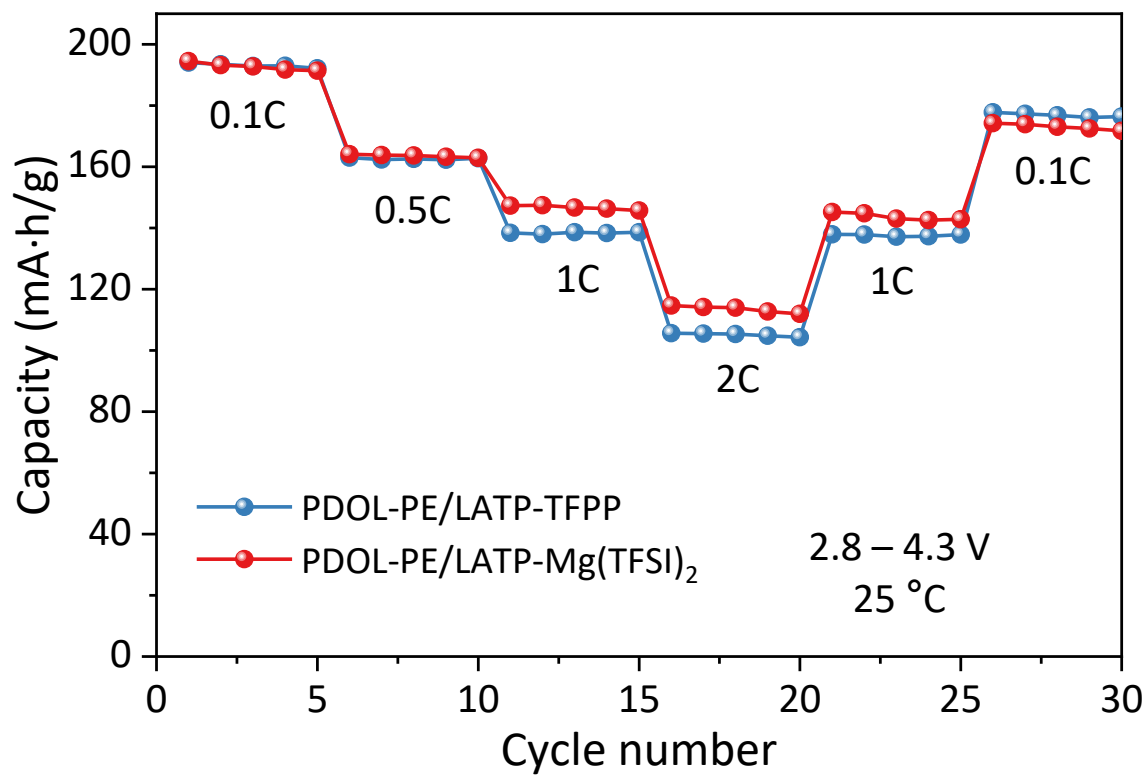

Supplementary Fig. 31 | Rate capability and electrochemical kinetic response at 25 °C. Comparative rate performance of the NCM811|Li cells employing PDOL-PE/LATP-TFPP and PDOL-PE/LATP-$Mg(TFSI)_2$ electrolytes from 0.1C to 2C.

These comparisons conclusively validate the "1+1>2" synergistic effect: $Mg(TFSI)_2$ prevents anode dendrites and enhances kinetics, while TFPP secures high-voltage cathode stability. The dual-additive PDOL-PLMT system integrates these distinct advantages, achieving a performance level that neither single additive can accomplish alone. We have incorporated the aforementioned

additional battery data into the revised Supplementary Information.

2. Although the Supporting Information (lines 130-138) states the total cell mass (~10 g), this is unconvincing for a 1.2 Ah pouch cell. Given that the NCM811 cathode required for this capacity already accounts for the majority of the mass, the remaining weight allowance seems insufficient for the necessary inactive components (Al-laminated film, tabs, etc.). To validate the claimed 456.7 Wh kg-1, the authors must provide a table detailing the specific weight of each component (cathode, Li anode, electrolyte/separator, current collectors, tabs, and packaging film) to prove the mass data is realistic.

Response: *We sincerely thank the reviewer for this rigorous examination of our energy density calculations. We fully acknowledge that a gravimetric energy density of 456.7 Wh kg^{-1} is exceptionally high, and it is entirely reasonable to question the mass inventory of the inactive components (packaging, tabs, etc.) in a 1.2 Ah pouch cell.*

We have provided a comprehensive Mass Breakdown Table in Supplementary Table S3. We have weighed every component used in the assembly of the 1.2 Ah pouch cell. The specific breakdown is summarized below:

Supplementary Table 3 Detailed weight breakdown of components in the 1.2 Ah pouch cell.

Cell component	Specification	Mass (g)	Mass Fraction
Cathode electrode	High loading (25 mg cm^{-2}, double-sided)	6.3305	63.21%
Anode electrode	Thin Li foil (100 μm)	0.8455	8.44%
Separator	Ultrathin (~8 μm)	0.234	2.34%
Electrolyte	PDOL-based SPE	1.785	17.82%
Packaging	Ultrathin Al-laminate film (68 μm)	0.716	7.15%

Tabs	Al/Ni tabs (Ultralight)	0.104	1.04%
Total		10.015	100.00%

The total cell weight of ~10 g is achieved not by omitting inactive components, but through specific lightweighting strategies inherent to our Li-metal design:

- *Ultrathin Li-Metal Anode: unlike traditional graphite anodes (which require heavy copper current collectors and have low specific capacity), our use of a 100 μm ultrathin Li foil significantly eliminates dead weight, which is the primary contributor to the high Wh kg $^{-1}$.*
- *Ultrathin Electrolyte/Separator: The in situ polymerized SPE is only 9.6 μm thick. This is substantially lighter than commercial separator-electrolyte systems (~ 20-25 μm + liquid electrolyte uptake), minimizing the inactive mass of the separator and eliminating excess liquid electrolyte weight.*
- *Optimized Packaging: For this demonstration, we utilized a customized ultrathin aluminum-laminated film (thickness: 68 μm) and minimized the excess sealing area to maximize the active-volume-to-packaging ratio.*

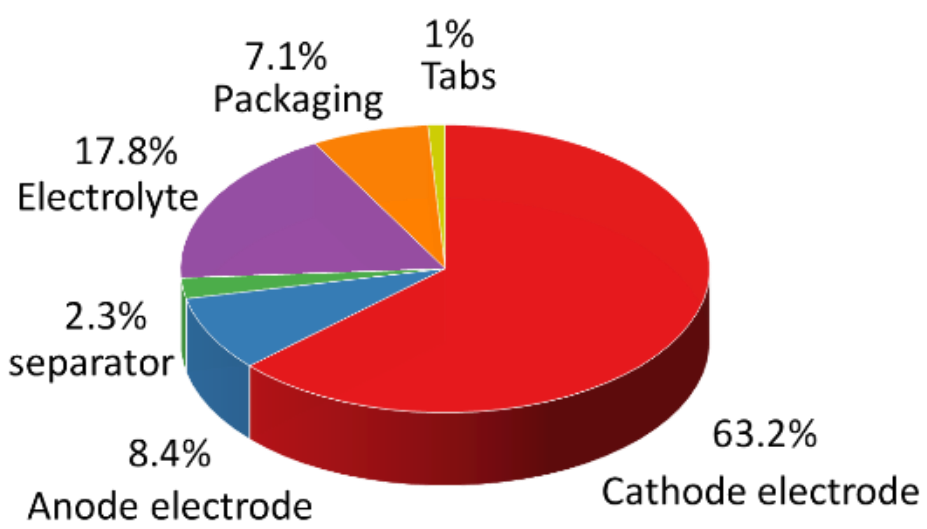

Supplementary Fig. 36 | Mass fractions of individual components in the 1.2 Ah pouch cell.

This calculation strictly includes all cell components, confirming the validity of our report.

3. To rigorously validate the high-voltage performance, the provided LSV data is insufficient. The authors are advised to perform high-voltage electrochemical floating tests (monitoring leakage

current over time at 4.7 V or 4.8 V) to demonstrate that the electrolyte remains stable under sustained oxidative stress.

Response: *We sincerely appreciate the reviewer's rigorous scrutiny regarding the high-voltage stability assessment. We fully agree that operating at 4.5 V presents a significant challenge for conventional ether electrolytes, and we acknowledge that potentiostatic leakage current measurements serve as the definitive standard for validating electrolyte tolerance under sustained oxidative stress, surpassing the dynamic limitations of LSV.*

To rigorously verify the stability limit beyond dynamic LSV scans, we performed Voltage Floating Tests (chronoamperometry) by holding the cells at fixed high voltages and monitoring the steady-state leakage current. As illustrated in Supplementary Fig. 12, the comparative analysis reveals a distinct stability hierarchy: PDOL-PE: Exhibits a sharp surge in leakage current at 4.5 V, indicative of rapid polymer decomposition. PDOL-PE/LATP: Shows improved stability but eventually succumbs to significant oxidation near 4.7 V. PDOL-PE/LATP-TFPP: Demonstrates superior high-voltage tolerance. Even when held at 4.8 V, the leakage current remains negligible and stable over time.

This low steady-state current confirms that the PDOL-PE/LATP-TFPP electrolyte does not undergo the continuous decomposition typical of ether-based solvents at such voltages. Instead, the data suggests that TFPP undergoes a self-limiting sacrificial oxidation process. This mechanism forms a thin, passivating CEI that effectively shields the electrolyte from further degradation, thereby enabling stable operation at 4.5 V. We have incorporated the floating test data into the Supplementary Information and expanded the corresponding discussion in the revised manuscript (page 14, line 233).

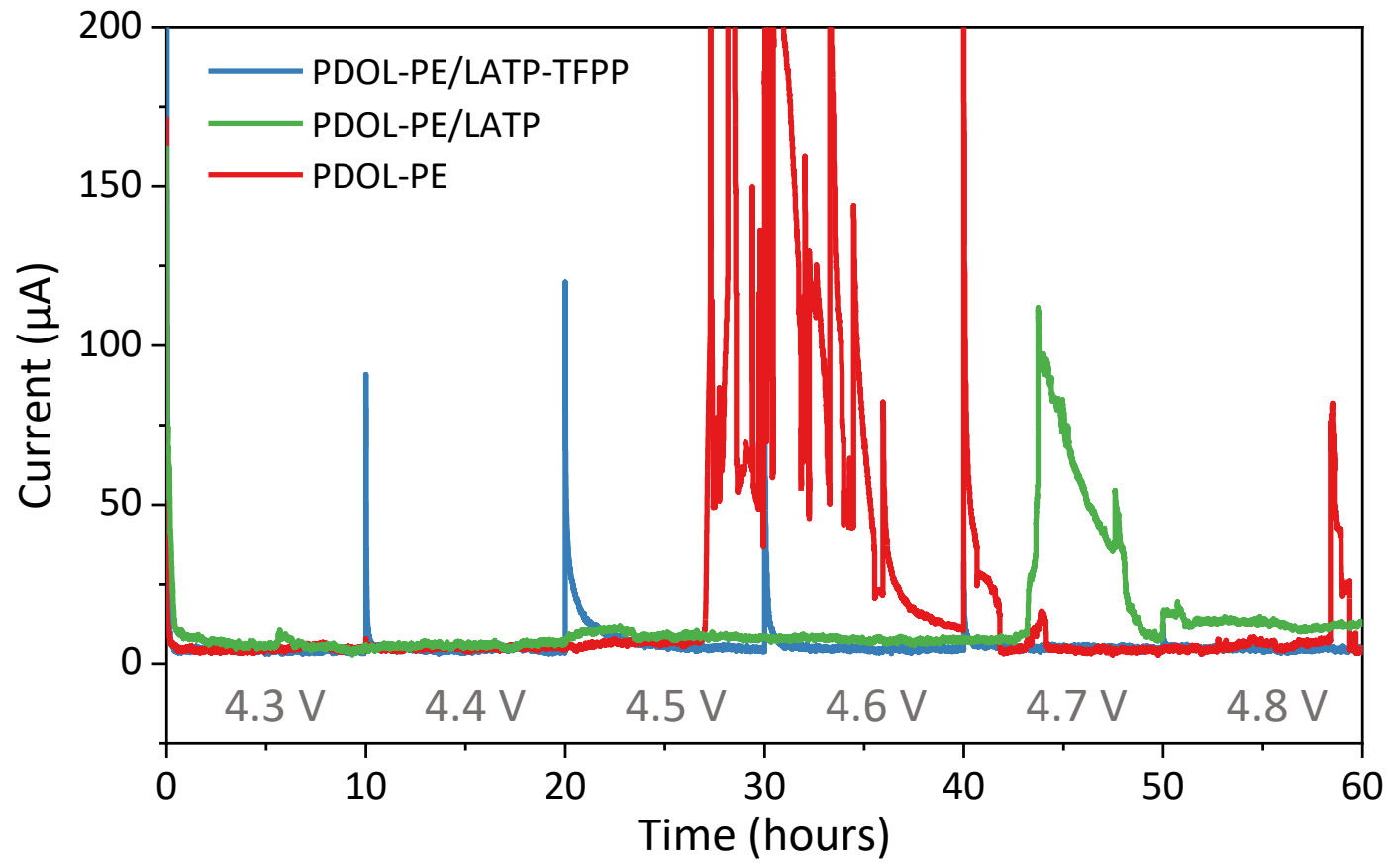

Supplementary Fig. 12 | High-voltage stability of the SPEs. Electrochemical floating tests of

NCM811|Li cells assembled with PDOL-PE, PDOL-PE/LATP and PDOL-PE/LATP/TFPP SPEs.

4. There are several typos in the manuscript. For instance, in Equation 1, aera should be corrected to area. The authors should carefully check the entire text to eliminate similar errors.

Response: *We sincerely thank the reviewer for the careful reading of our manuscript and for pointing out this oversight. We apologize for the typographical error in Equation 1. We have corrected "aera" to "area" in Equation 1 as suggested. Furthermore, we have conducted a thorough proofreading of the entire manuscript to identify and eliminate any remaining typographical or grammatical errors, ensuring the text now meets the high standards of publication.*

5. The current evaluation relies on Li/Li symmetric cells, which utilize an infinite Li that can mask irreversible Li loss. To rigorously assess the anode reversibility and the specific efficacy of the $Mg(TFSI)_2$ additive in suppressing dead lithium formation, the cycling performance of Li/Cu cells are highly recommended.

Response: *We sincerely thank the reviewer for highlighting the inherent limitations of Li/Li symmetric cells. We fully acknowledge that the excess lithium reservoir in such configurations can indeed mask irreversible lithium loss, and we agree that Li|Cu asymmetric cell testing is indispensable for a rigorous and transparent assessment of the true anode reversibility.*

Following the reviewer's suggestion, we assembled Li|Cu cells with four comparative electrolyte systems and tested them under the stringent conditions of 0.5 mA cm^{-2} and 5 mAh cm^{-2}. The results are presented in Supplementary Fig. 20 and summarized below: The PDOL-PLMT (dual-additive) system demonstrated exceptional reversibility, achieving a high Average Coulombic Efficiency (ACE) of 99.03%. Crucially, the PDOL-PE/LATP-$Mg(TFSI)_2$ sample exhibited nearly identical performance to the full PLMT system. This comparative result provides definitive evidence that the $Mg(TFSI)_2$ additive is the dominant factor governing anode stability. The in-situ formation of a lithiophilic Li-Mg alloy interface regulates uniform Li deposition, enabling high efficiency even under deep cycling (5 mAh cm^{-2}) conditions.

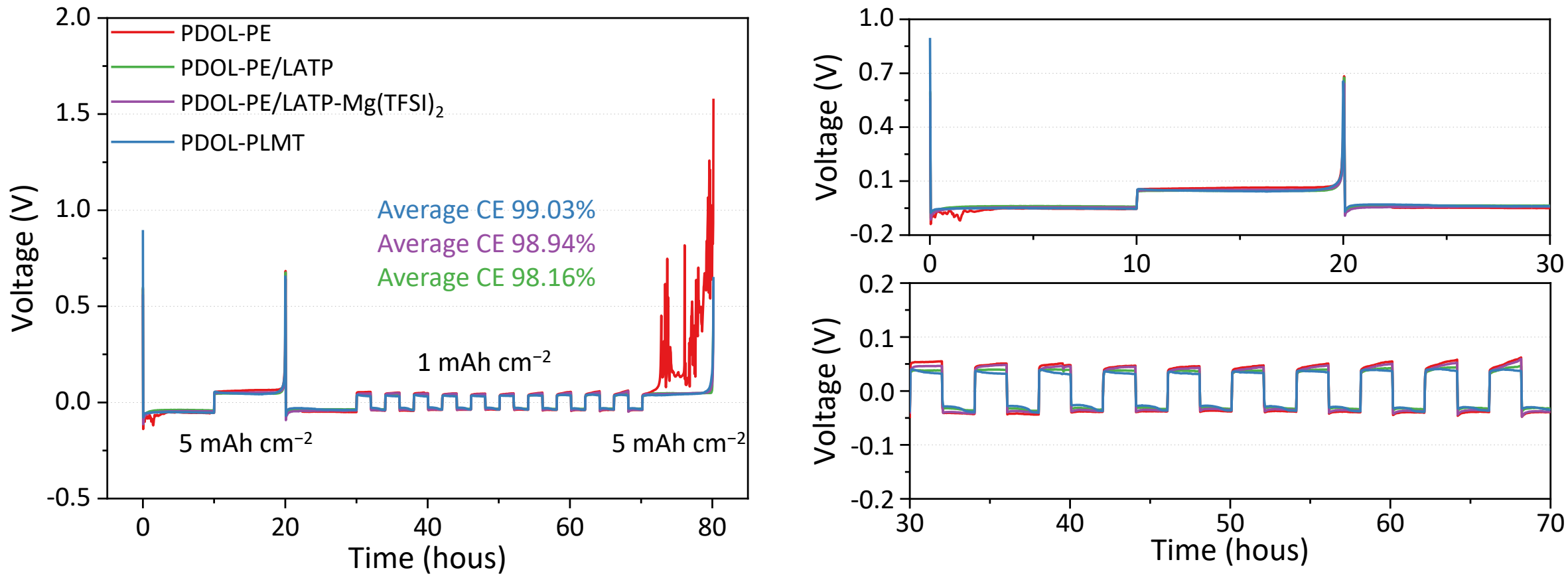

Supplementary Fig. 20 | Electrochemical reversibility of lithium plating and stripping. Comparative Li plating/stripping stability of Li|Cu cells with the four indicated SPEs (PDOL-PE, PDOL-PE/LATP, PDOL-PE/LATP-$Mg(TFSI)_2$, and PDOL-PLMT).

In sharp contrast, the PDOL-PE failed to support such high-capacity cycling. As shown in the voltage profiles (Supplementary Fig. 20), the PDOL-PE cell exhibited erratic voltage oscillations during the initial plating stages, indicative of uneven deposition and potential dendrite nucleation. During the stripping step after approximately 70 hours, the voltage polarization increased sharply (rapid impedance buildup). We have included the Li|Cu half-cell data in the Supplementary Information and added the corresponding discussion to the revised manuscript (page 22, line 352).

6. The authors should conduct Molecular Dynamics simulations to determine whether the additives participate in Li^+ coordination.

Response: *We sincerely thank the reviewer for this highly constructive suggestion. We fully agree that understanding the solvation structure at the molecular level is essential to elucidate whether the additives interfere with the intrinsic bulk ion transport. To address this, we have conducted comprehensive Molecular Dynamics (MD) simulations to compare the local coordination environments of the baseline PDOL-LATP electrolyte and the optimized dual-additive PDOL-PLMT electrolyte. The newly obtained Radial Distribution Functions (RDFs) and corresponding Coordination Numbers (CNs) are now provided in revised Supplementary Fig. 19.*

1. Non-participation of Additives in the Li^+ Solvation Sheath

Our structural analysis explicitly confirms that neither the TFPP molecule nor the Mg^{2+} cation actively participates in the primary Li^+ coordination sphere. The RDF peaks for Li^+-TFPP and Li^+-

Mg^{2+} are absent, and their corresponding CNs are effectively zero. This lack of coordination is thermodynamically expected: Mg^{2+} naturally repels Li^+ due to strong electrostatic repulsion, and the bulky, fluorinated TFPP molecule exhibits much weaker nucleophilicity toward Li^+ compared to the abundant ether oxygens in the PDOL backbone.

2. Preservation of the Intrinsic Li^+ Transport Pathways

Crucially, the introduction of the dual additives does not perturb the primary solvation environment of the polymer matrix. The RDF and CN profiles for the interactions between Li^+ and the polymer backbone (Li^+-PDOL), as well as between Li^+ and the primary anion (Li^+-$TFSI^-$), remain nearly identical in both the PDOL-LATP and PDOL-PLMT systems.

These MD simulations provide definitive theoretical proof that TFPP and $Mg(TFSI)_2$ do not trap Li^+ or alter its conduction mechanism within the bulk electrolyte. Instead, their functional roles are strictly confined to the electrode interfaces (forming the CEI and Li-Mg alloy, respectively). This perfectly explains why the PDOL-PLMT system can achieve dual interfacial stabilization while simultaneously maintaining the unhindered, high-speed Li^+ transport required for superior rate capability.

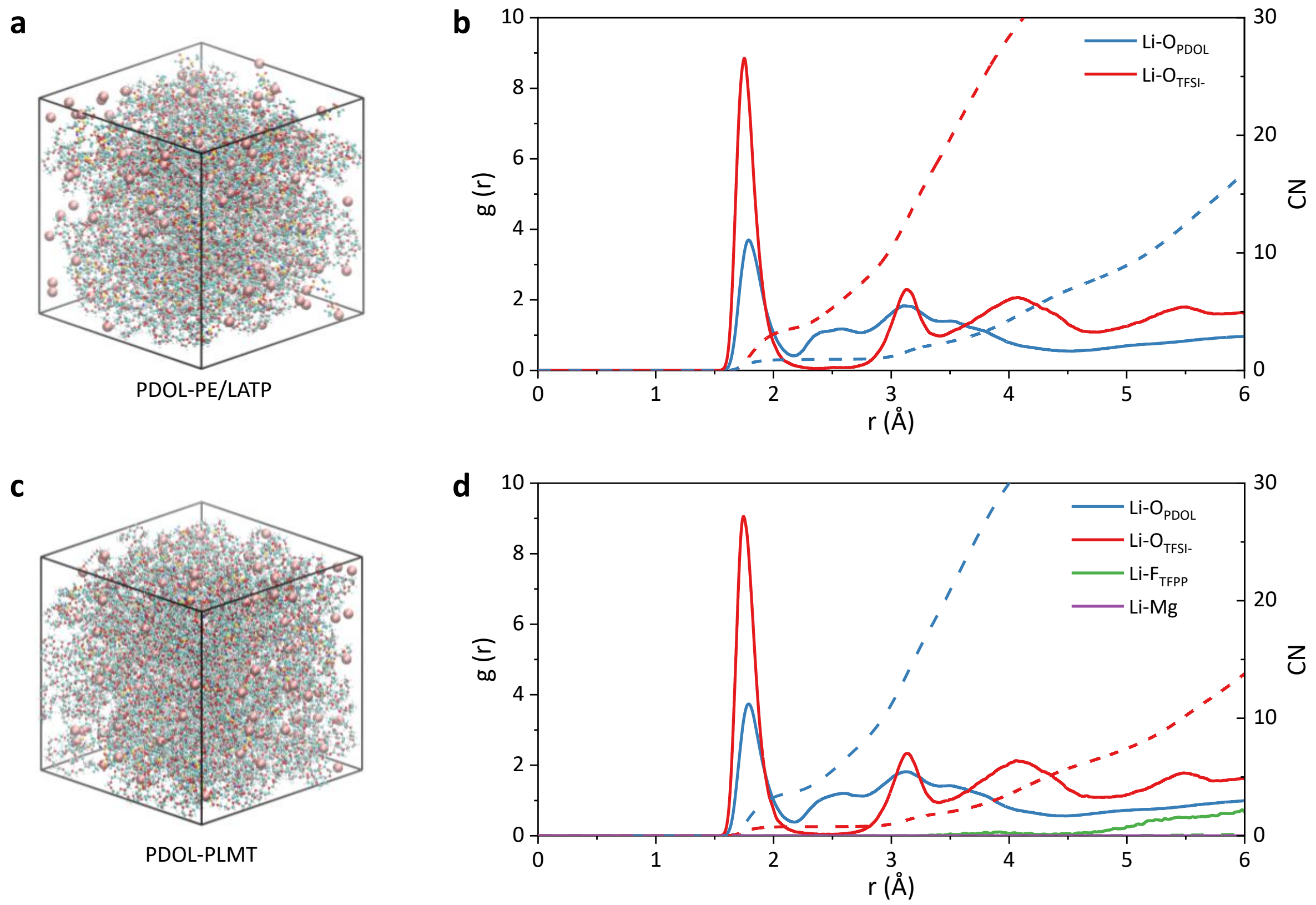

Supplementary Fig. 19 | Molecular dynamics (MD) simulations of the Li^+ coordination

environments. a MD snapshots (**b**) RDF and CN of PDOL-PE/LATP electrolyte. **c** MD snapshots (**d**) RDF and CN of PDOL-PLMT electrolyte.

7. The TFPP additive causes a significant irreversible capacity loss (43 mAh g^{-1}), leading to a low initial Coulombic efficiency (< 75%), which is concerning for practical cells. The authors must explicitly state the N/P ratio of the 1.2 Ah pouch cells and critically discuss whether this high Li consumption is viable for lean-Li battery designs.

Response: *We sincerely appreciate the reviewer's critical assessment regarding the initial coulombic efficiency (ICE) and its implications for practical lean-Li cell design. We fully acknowledge that an irreversible capacity loss of 43 mAh g^{-1} (resulting in <75% ICE) is a significant consumption of the lithium inventory.*

However, thermodynamically, this initial capacity consumption represents a necessary interfacial activation cost to establish a robust, long-term passivation layer. The 'excess' charge capacity originates entirely from the sacrificial oxidative decomposition of the TFPP additive, leading to a highly dense, F-rich CEI, and is not indicative of NCM811 structural degradation. Specifically, we would like elaborate the configuration design in response to the viability concerns with the following points.

1. Origin of the Excess Charge Capacity

The observed "excess" charge capacity (~ 43 mAh g^{-1}) originates primarily from the sacrificial oxidative decomposition of the TFPP additive, rather than the over-delithiation or structural degradation of the NCM811 cathode. The TFPP additive undergoes sacrificial oxidation to form a dense, F-rich Cathode Electrolyte Interphase (CEI). Simultaneously, the balanced reduction at the anode (in the presence of Mg salt) promotes a highly compact Li-Mg alloy. This "densification" process consumes more charge initially compared to forming a porous, organic-rich layer (PDOL-PE), but it effectively terminates further electrolyte decomposition.

2. N/P Ratio and Lithium Inventory Buffer

For practical cell design, we have explicitly stated that our 1.2 Ah pouch cells utilize an N/P ratio of 2.06 (using a 100 μm Li foil). This ratio provides an adequate buffer to accommodate the initial passivation cost without starving the cell of active lithium for subsequent cycling. More importantly, this strategic trade-off yields exceptional steady-state stability. And the "passivation

cost" is strictly limited to the initial cycles.

3. Strategic Trade-off Between ICE and Steady-State CE

The viability of this design is validated by the long-term cycling performance. Once the TFPP-derived CEI is established, the cell transitions to a highly stable cycling regime. As shown in Fig. 6c and d, the PDOL-PLMT pouch cells exhibit a superior average Coulombic efficiency of >99.5% over long-term cycling. In contrast, PDOL-PE pouch cells with higher ICE but unstable interfaces suffer from continuous, parasitic Li consumption (low average CE), leading to faster failure. Therefore, in practical SLMB applications, sacrificing a fraction of the initial lithium inventory to halt continuous parasitic reactions ultimately maximizes the operational lifespan and safety of the cell.

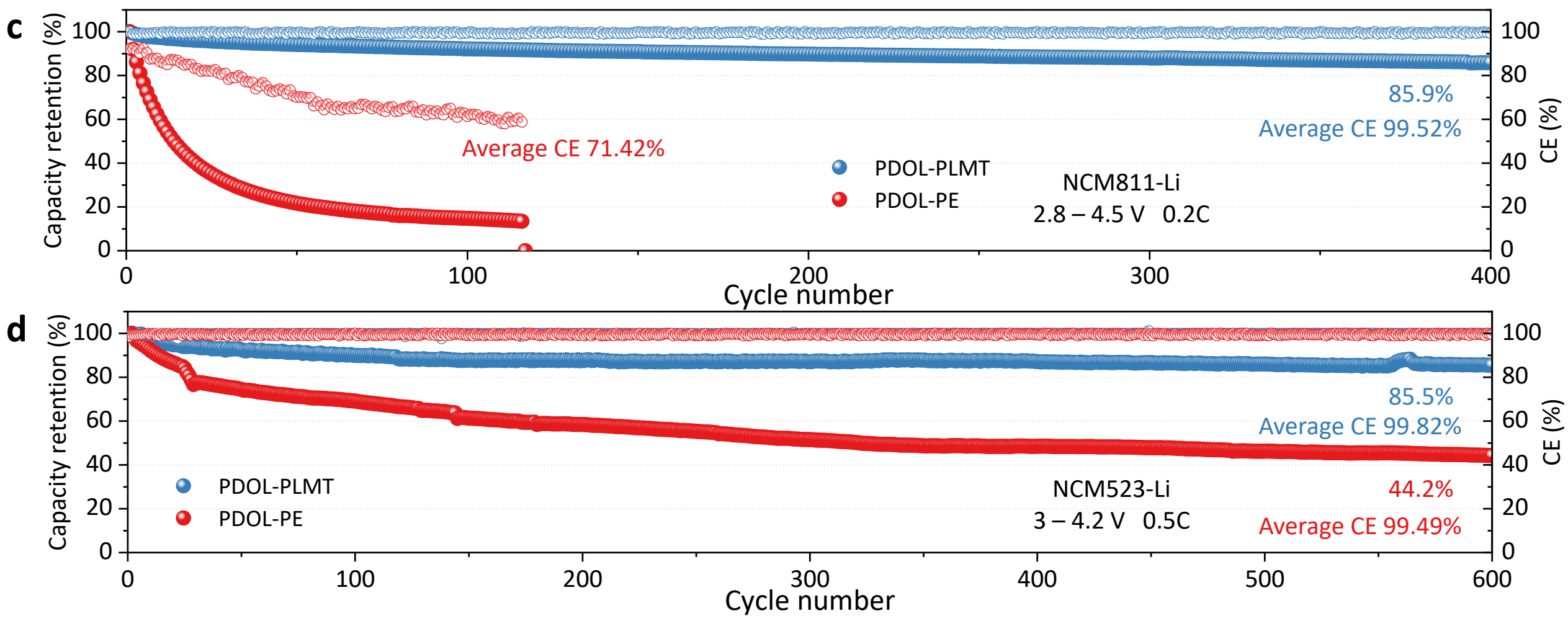

Fig. 6 | c Room-temperature cycling stability of NCM811|Li pouch cells with PDOL-PE and PDOL-PLMT SPEs within 2.8–4.5 V. **d** Room-temperature cycling performance of NCM523|Li pouch cells with PDOL-PE and PDOL-PLMT SPEs within 3.0–4.2 V.

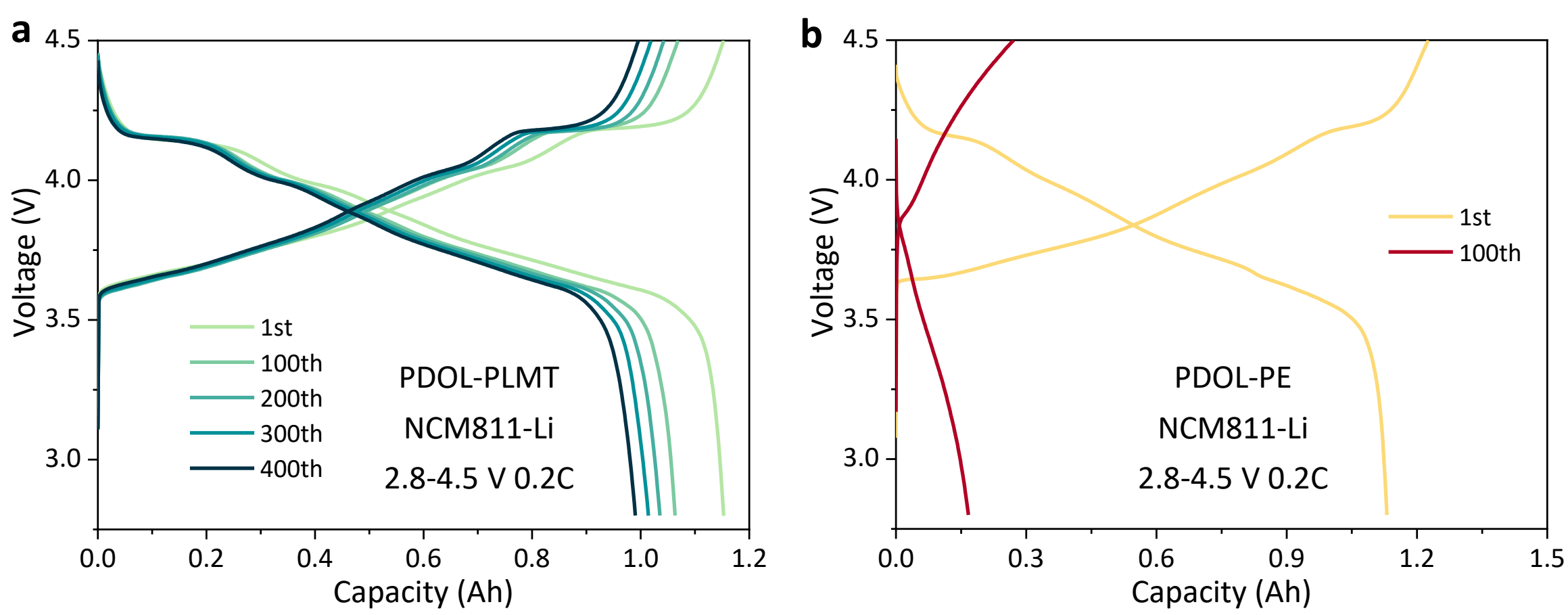

Supplementary Fig. 37 | Electrochemical performance of the 1.2 Ah practical-scale pouch cells

at 4.5 V. Charge-discharge profiles of 1.2 Ah (**a**) NCM811|PDOL-PLMT|Li and (**b**) NCM811|PDOL-PE|Li pouch cells cycled between 2.8 – 4.5 V.

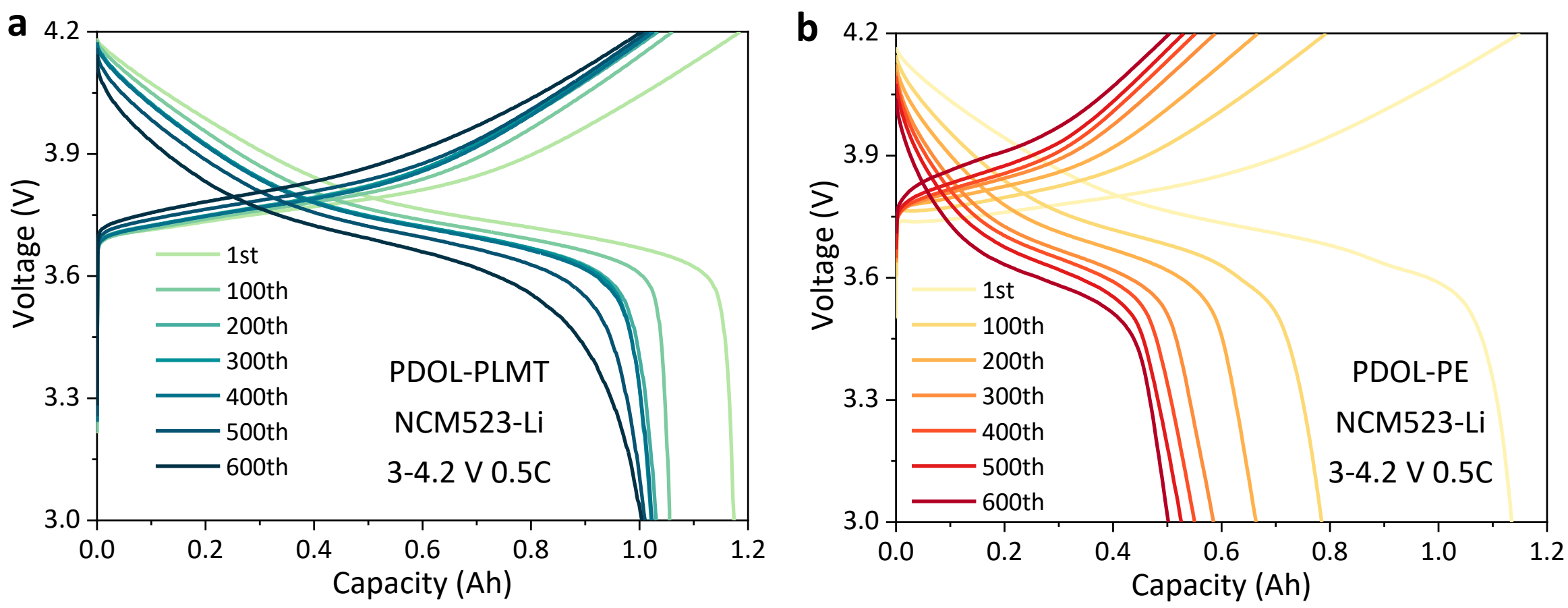

Supplementary Fig. 38 | Demonstration of the electrolyte universality in 1.2 Ah NCM523-based pouch cells. Charge-discharge profiles of (**a**) NCM523|PDOL-PLMT|Li and (**b**) NCM523|PDOL-PE|Li pouch cells cycled between 3 – 4.2 V.

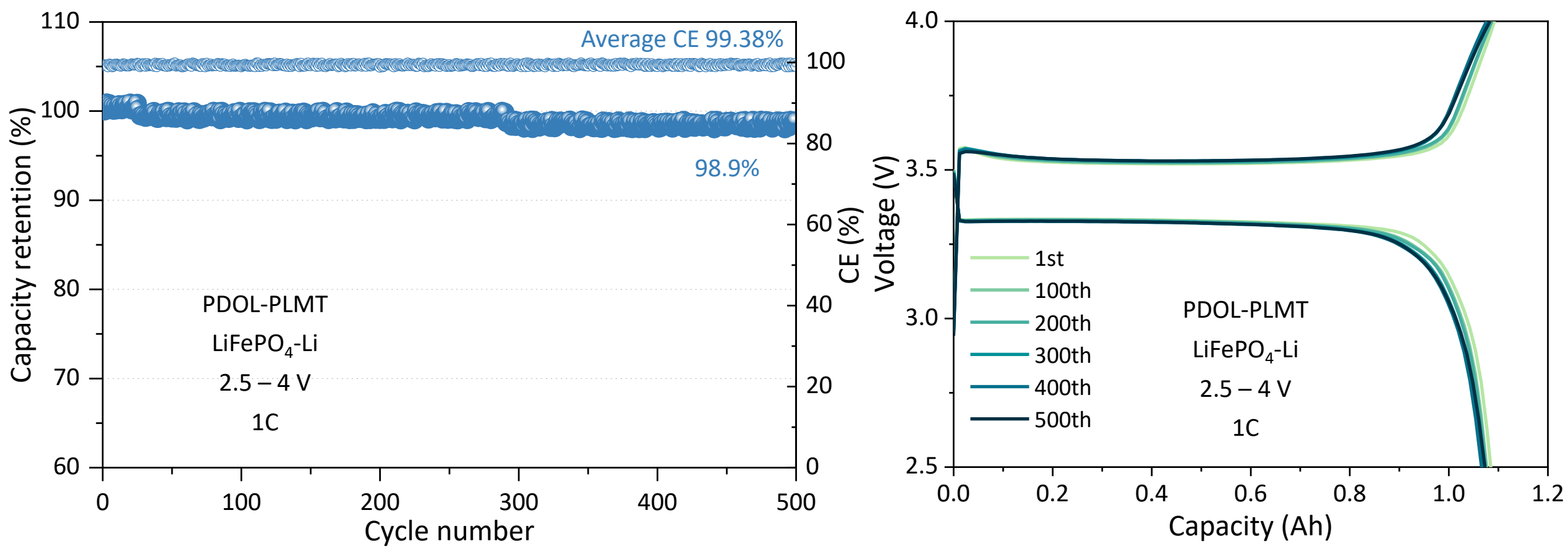

Supplementary Fig. 39 | Cycling performance and Charge-discharge profiles of the LiFePO4| PDOL-PLMT |Li pouch cell at 25 °C.

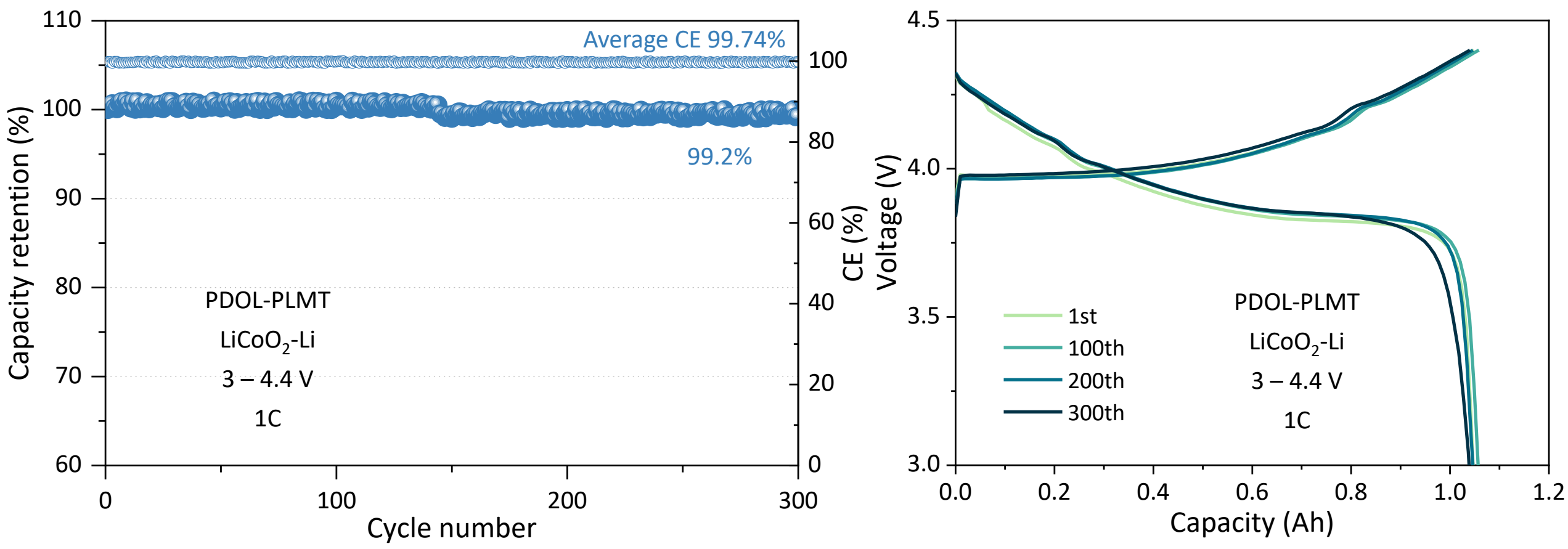

Supplementary Fig. 40 | Cycling performance and Charge-discharge profiles of the $LiCoO_2$|PDOL-PLMT|Li pouch cell at 25 °C.

8. Figure S10 and Line 239 indicate a low initial Coulombic efficiency due to TFPP decomposition (43 mAh g^{-1} loss). However, the pouch cell cycling data (e.g., Figure 5c) does not seem to reflect such a severe capacity loss in the first cycle, which may be due to the undisclosed formation step. Please define the formation protocol (C-rate, voltage range, cycles) used for the pouch cells in Methods.

Response: *We sincerely appreciate the reviewer's keen observation regarding the initial capacity loss and the specific cycling data presentation. We agree that the decomposition of TFPP induces an irreversible capacity loss (~ 43 mAh g^{-1}) during the initial SEI/CEI construction, which is a necessary trade-off for the subsequent long-term stability.*

We respectfully clarify that the cycling performance presented in Fig. 6 (the previous Figure 5) reflects the cell behavior after the completion of the activation and formation process. The initial irreversible capacity loss mentioned by the reviewer occurred during the specific "Formation Protocol" (described below) and was therefore not plotted in the long-term cycling figures. This is a standard practice for Ah-scale pouch cells to ensure the reported cycle life reflects the stable operation phase.

In response to the reviewer's request, we have detailed the specific formation procedures (C-rate, voltage range, and degassing steps) in the Experimental Section of the revised manuscript (page 34, line 554). The protocol is designed to ensure complete wetting, controlled polymerization, and gas removal: After electrolyte injection and pre-sealing, the pouch cells were kept at 45 °C for 48 hours. This step ensures the complete infiltration of the precursor into the electrode pores and the completion of the in-situ polymerization reaction prior to electrochemical charging. We adopted a multi-step formation strategy. During this stage, a uniform stack pressure of 0.5 MPa was applied to the fixture to ensure dense interface formation.

1) NCM811|Li pouch cells:

Constant current (CC) charge at 0.05 C to 3.8 V, rest for 10 min.

CC charge at 0.1 C to 4.5 V, followed by constant voltage (CV) holding until current drops below 0.02 C.

Rest for 10 min, then CC discharge at 0.1 C to 2.8 V.

2) **NCM523|Li pouch cells:**

   Similar protocol with a cutoff voltage of 4.2 V (0.05 C to 3.8 V → 0.1 C/CV to 4.2 V → Discharge to 3.0 V).

3) **LFP|Li pouch cells:**

   Protocol adapted for LFP (0.05 C to 3.3 V → 0.1 C/CV to 4.0 V → Discharge to 2.5 V).

4) **LCO|Li pouch cells:**

   Protocol adapted for high-voltage LCO (0.05 C to 3.8 V → 0.1 C/CV to 4.4 V → Discharge to 3.0 V).

Critically, after the formation cycles, the cells were transferred to a Ar glovebox for degassing (gas bag removal) and secondary sealing. This step effectively removes any gaseous byproducts generated from the sacrificial oxidation of TFPP, preventing cell swelling and ensuring compact assembly. Subsequent long-term cycling tests were conducted under a controlled stack pressure of 0.2 MPa to accommodate volume expansion while maintaining interface contact.

General Response to the Concerns from the Editor and Reviewers' comments

We sincerely thank the editorial team and the reviewers for their positive feedback and for recommending the acceptance of our manuscript. We are deeply grateful for your rigorous guidance throughout the entire review process, which has fundamentally elevated the quality of our work.

In this final revision, we have carefully addressed the remaining editorial requests and the final comment from Reviewer #3. Please find our point-by-point responses below.

Point-by-point responses to reviewer's comments

Response to Reviewer #1

Reviewer #1: The authors revised the manuscript carefully, I recommend acceptation of this revised manuscript.

Response: *We sincerely thank the reviewer for recognizing the careful revisions we have made and for recommending the acceptance of our manuscript. We are deeply grateful for your time, rigorous evaluation, and highly constructive feedback throughout the review process, which have significantly elevated the quality of our work.*

Response to Reviewer #2

Reviewer #2: The authors have addressed my questions. I support its publication in this version.

Response: *We thank the reviewer for thoroughly reviewing our revised manuscript and for supporting its publication. Your insightful questions and comments during the previous rounds were invaluable in helping us clarify our mechanistic interpretations and strengthen the overall evidence chain of our study. We deeply appreciate your time and expertise.*

Response to Reviewer #3

Reviewer #3: The authors have addressed most of my comments satisfactorily. However, I noticed that several figures (such as Supplementary Figure 28-31) in the Supplementary Information are neither cited in the main text nor accompanied by descriptive text within the SI document itself. The authors should check all the Figures and revise the text to direct the readers to these supplemental results where relevant.

Response: *We thank the reviewer for their meticulous reading and for pointing out this oversight. We sincerely apologize for the missing citations and figure descriptions.*

Following your highly constructive suggestion, we have conducted a comprehensive audit of all figures in the Supplementary Information. We have now explicitly cited Supplementary Figures 27–31 in the relevant sections of the main text to smoothly direct the readers (page 25, line 397, the highlight page). Furthermore, we have added detailed descriptive text and captions within the SI document to ensure these results are properly contextualized.

For instance, the newly added descriptions for Figures 27–31 in the revised SI document are as follows:

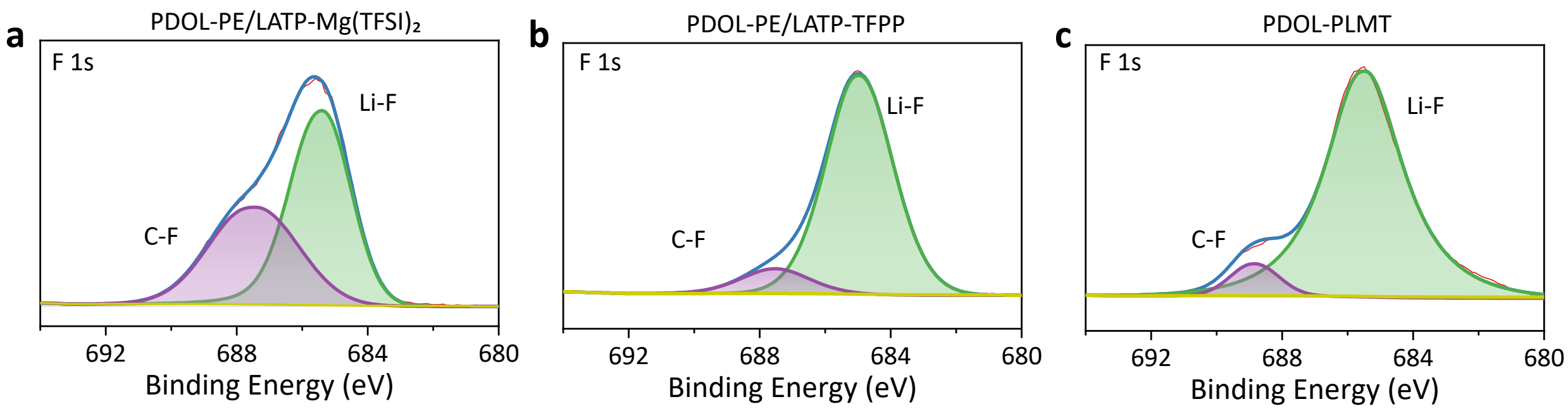

Supplementary Fig. 1 | Surface chemistry and SEI composition analysis on the Li metal anodes. High-resolution F 1s XPS spectra of the Li metal anode surfaces harvested from Li||NCM811 coin cells after 100 cycles (2.8–4.5 V, 20 mA g^{-1}, 25 °C), employing (**a**) PDOL-PE/LATP-$Mg(TFSI)_2$, (**b**) PDOL-PE/LATP-TFPP, and (**c**) PDOL-PLMT.

Comparative F 1s XPS analysis of the cycled Li anodes (Supplementary Fig. 27) reveals uniformly high LiF intensities across the single- and dual-additive systems. This confirms that the LiF-rich SEI is primarily dictated by the decomposition of the $TFSI^-$ anions, independent of TFPP. Consequently, this explicitly decouples the distinct additive functions, validating that the superior high-voltage stability conferred by TFPP strictly originates from its robust construction of the CEI.

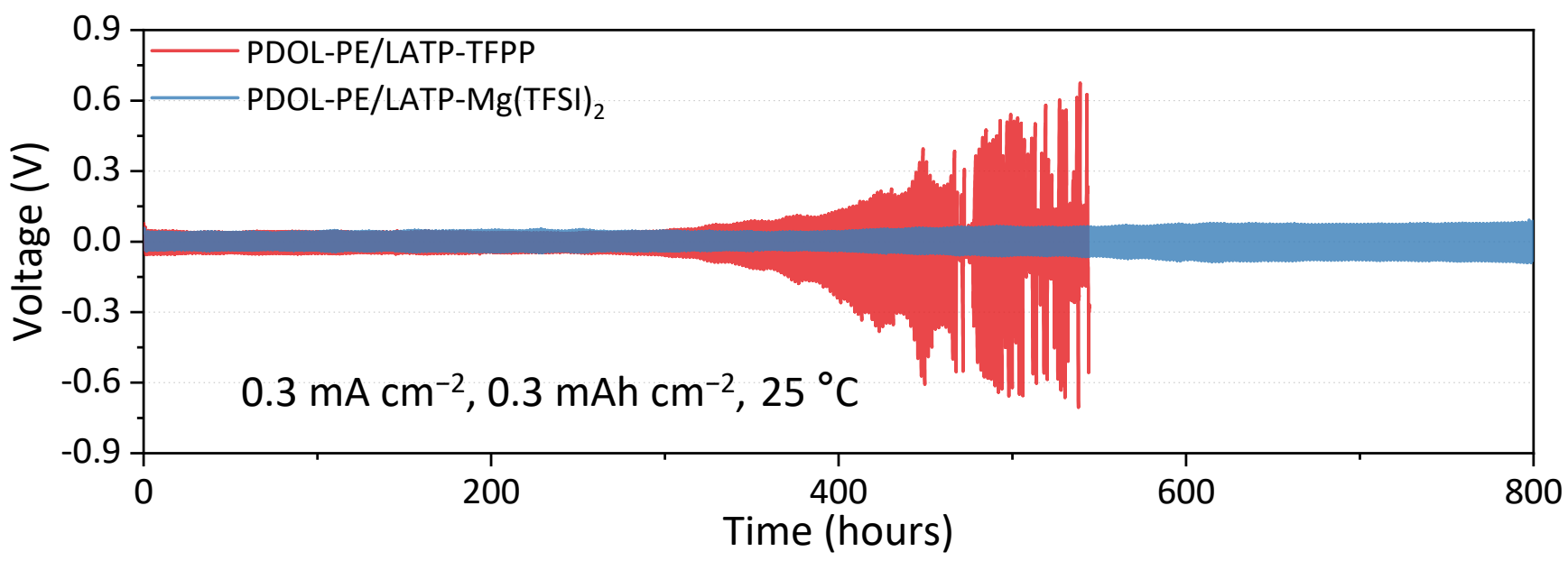

Supplementary Fig. 28| Long-term interfacial stability and lithium cycling endurance. Galvanostatic Li plating/stripping profiles of the Li|Li symmetric cells employing the PDOL-PE/LATP-TFPP and PDOL-PE/LATP-$Mg(TFSI)_2$ electrolytes.

To explicitly decouple the interfacial roles of the additives and evaluate lithium plating/stripping stability, Li|Li symmetric cells were systematically evaluated (Supplementary Fig. 28). The control electrolyte formulated exclusively with the PDOL-PE/LATP-TFPP succumbed to soft short-circuits within 500 hours, indicative of uncontrolled dendritic penetration. In stark contrast, the incorporation of $Mg(TFSI)_2$ enabled remarkably stable voltage profiles for over 800 hours. This comparative electrochemical evidence unambiguously dictates that the in situ formed Li–Mg alloy interphase is the dominant factor in regulating homogeneous Li deposition and fundamentally preventing dendrite-induced failure.

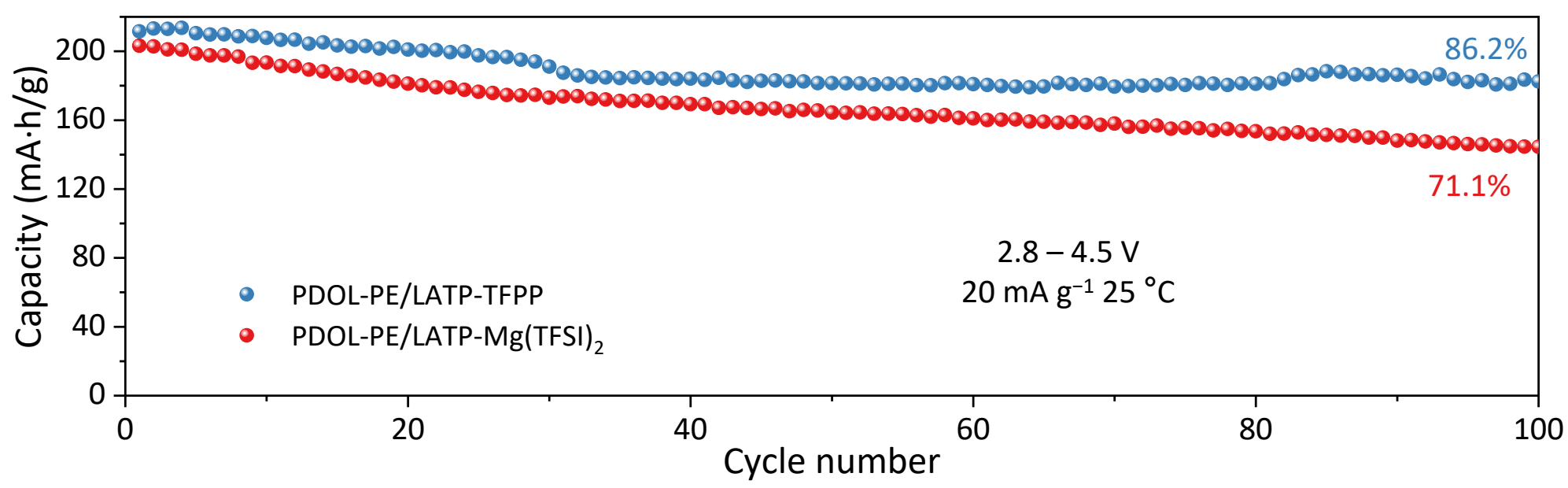

Supplementary Fig. 29| High-voltage cycling endurance and capacity retention. Long-term cycling performance of Li||NCM811 cells using PDOL-PE/LATP-TFPP and PDOL-PE/LATP-$Mg(TFSI)_2$ electrolytes at 4.5V (25 °C).

Evaluating full-cell performance under an aggressive 4.5 V cut-off explicitly isolates the cathode-stabilizing efficacy of TFPP (Supplementary Fig. 29). The $Mg(TFSI)_2$-only control exhibited severe degradation, with capacity retention plummeting to 71.1% after 100 cycles. In sharp contrast,

the isolated addition of TFPP effectively mitigated this decay, delivering an 86.2% retention that closely approaches the fully integrated dual-additive system. This comparative evaluation conclusively establishes that the TFPP-derived CEI is essential for protecting the highly reactive NCM811 cathode under severe high-voltage stress.

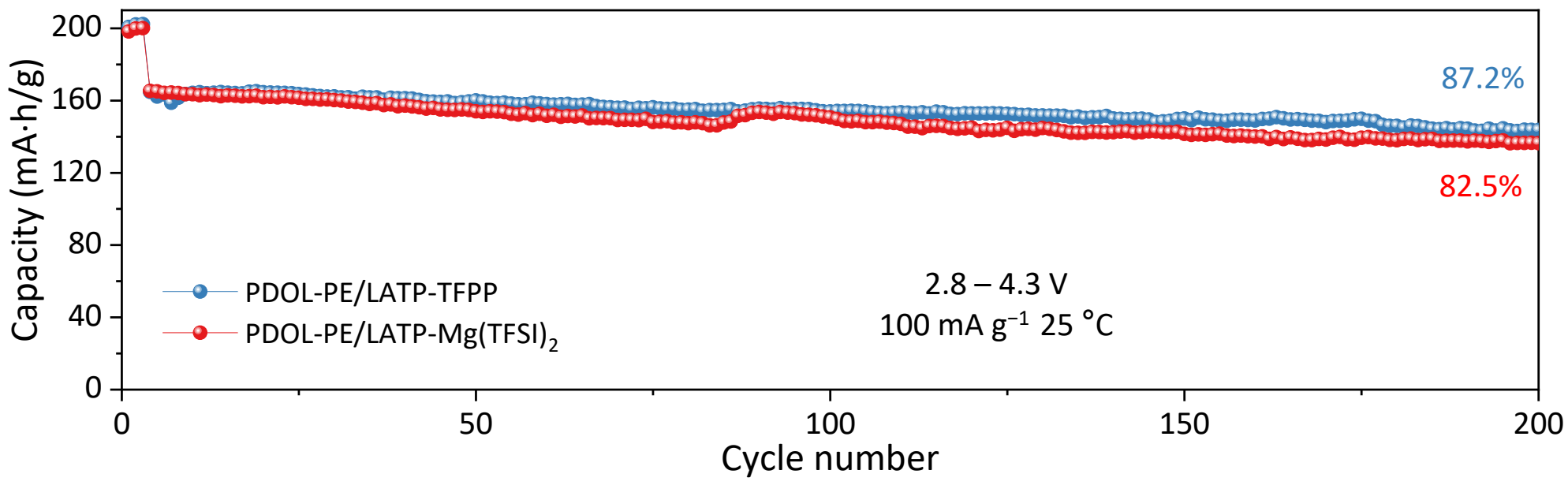

Supplementary Fig. 30 | Comparative evaluation of the long-term cycling endurance. Long-term cycling performance of Li||NCM811 coin-cells using PDOL-PE/LATP-TFPP and PDOL-PE/LATP-$Mg(TFSI)_2$ electrolytes at 100 mA g^{-1} (2.8–4.3 V, 25 °C).

To further delineate the distinct interfacial contributions under continuous electrochemical stress, long-term cycling was evaluated at 0.5C (Supplementary Fig. 30). This comparative analysis highlights the indispensable role of TFPP in securing long-term reversibility. Specifically, the control system relying solely on the $Mg(TFSI)_2$ anodic regulator exhibited cumulative capacity fade, yielding an 82.5% retention after 200 cycles. In distinct contrast, the isolated addition of TFPP effectively suppressed this degradation, achieving a significantly superior retention of 87.2%. This quantitative performance gap unambiguously corroborates that the TFPP-derived CEI provides essential shielding for the cathode, sustaining its structural integrity throughout extended operation.

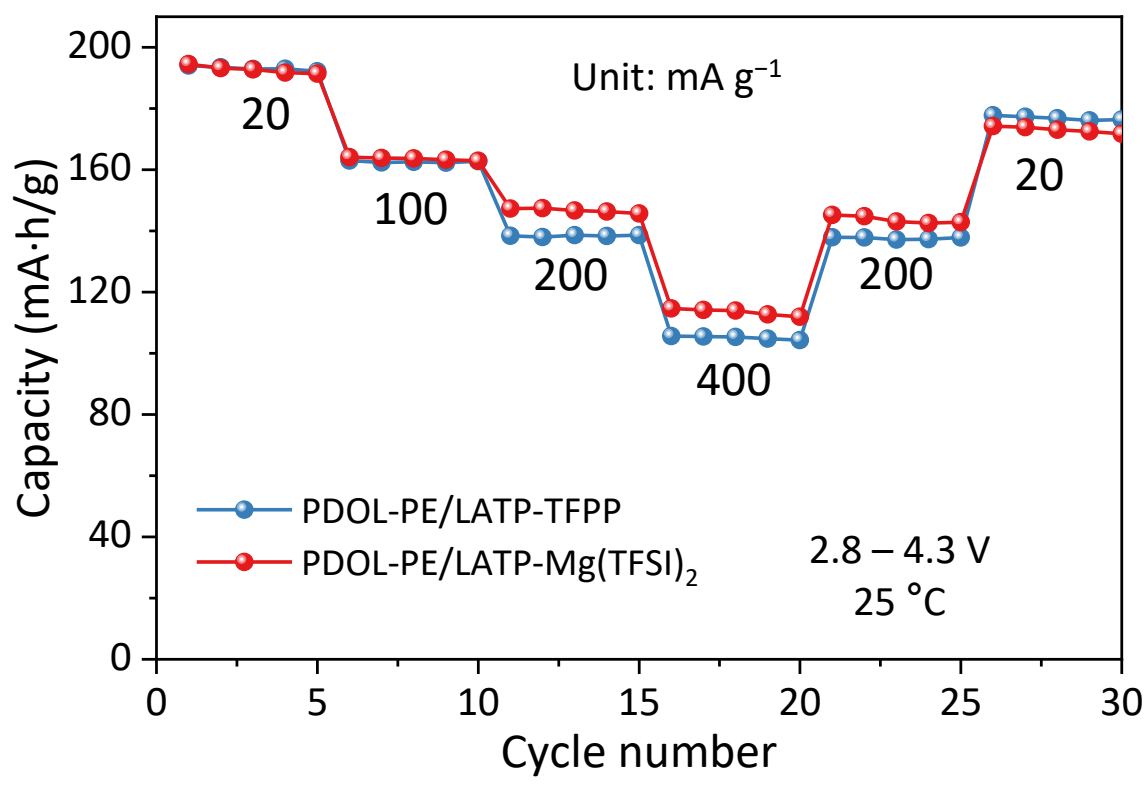

Supplementary Fig. 31| Rate capability and electrochemical kinetic response at 25 °C.

Comparative rate performance of the Li||NCM811 cells employing PDOL-PE/LATP-TFPP and PDOL-PE/LATP-$Mg(TFSI)_2$ electrolytes from 20 to 400 mA g^{-1}.

Complementary rate capability tests (Supplementary Fig. 31) precisely isolate the kinetic advantages imparted by the anodic regulator. As the applied current density incrementally increases, systems formulated with $Mg(TFSI)_2$ demonstrate profoundly superior high-rate kinetics compared to their Mg-free counterparts. This exceptional rate performance stems directly from the in situ construction of the lithiophilic Li–Mg alloy-rich SEI. By establishing a fast-ion conducting interfacial network that drastically minimizes the Li^+ diffusion barrier, the Mg-regulated interphase fundamentally facilitates rapid charge transfer, ensuring robust capacity delivery even under stringent high-rate conditions.
